# Two-Step Azidoalkenylation of Terminal Alkenes Using Iodomethyl Sulfones

**DOI:** 10.3390/molecules24224184

**Published:** 2019-11-18

**Authors:** Nicolas Millius, Guillaume Lapointe, Philippe Renaud

**Affiliations:** Department of Chemistry and Biochemistry, University of Bern, Freiestrasse 3, CH-3012 Bern, Switzerland; Nicolas.Millius@dsm.com (N.M.); guillaume.lapointe@novartis.com (G.L.)

**Keywords:** radical reaction, azidoalkylation, carboazidation, sulfones, azides, Julia–Kocienski olefination

## Abstract

The radical azidoalkylation of alkenes that was initially developed with α-iodoesters and α-iodoketones was extended to other activated iodomethyl derivatives. By using iodomethyl aryl sulfones, the preparation of γ-azidosulfones was easily achieved. Facile conversion of these azidosulfones to homoallylic azides using a Julia–Kocienski olefination reaction is reported, making the whole process equivalent to the azidoalkenylation of terminal alkenes.

## 1. Introduction

Organic alkyl azides are highly versatile compounds for synthesis [1,2,3,4]. They are unreactive towards a broad range of reaction conditions but, under dedicated conditions, they may become nitrene [5] and aminyl radical precursors [6,7,8,9,10,11] as well as suitable substrates for Schmidt reaction [12,13,14], aza-Wittig [15] reaction, and 1,3-dipolar cycloaddition [16,17].

**Scheme 1:**
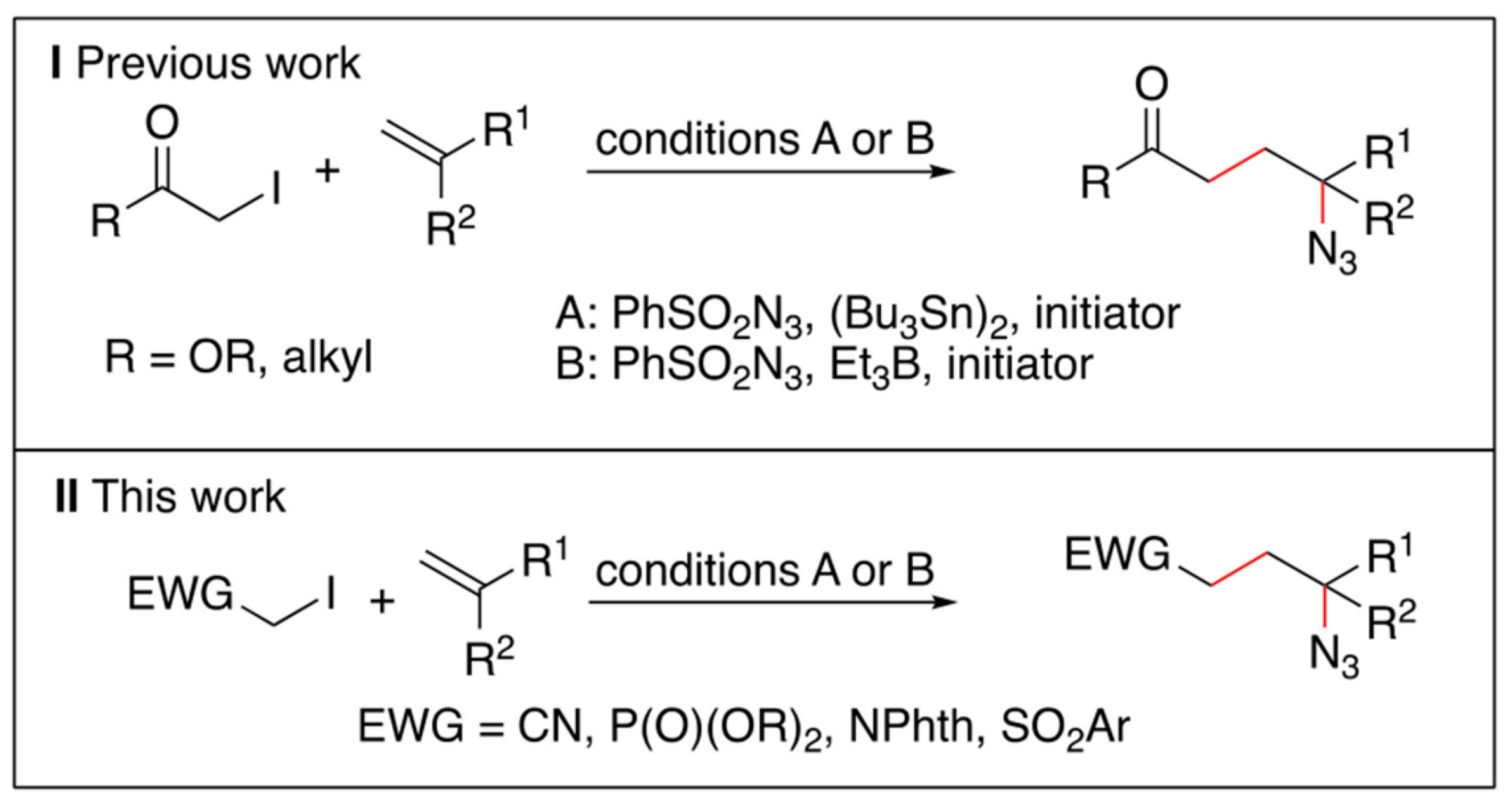
The radical carboazidation reaction.

They are commonly prepared via nucleophilic substitution of halides and related electrophiles using inorganic azides [18]. For tertiary alkyl azides, the nucleophilic substitution approach is often difficult, and free radical processes have proven to be a very convenient alternative. Radical azidation reactions are run under mild conditions, and they are compatible with a broad range of functional groups [19,20,21]. The carboazidation of alkenes represents a particularly attractive method to transform terminal alkenes into functionalized alkyl azides [22]. It is performed under chain transfer conditions and has been employed as a key step in several alkaloids syntheses [20,23,24,25,26,27,28,29]. Alternatively, carboazidation using under transition metal catalysis has also been reported [30,31,32,33,34]. Except for one reaction involving CCl_3_Br [22], the reaction has mainly been used with α-iodoesters and α-iodoketones (Scheme 1I) under ditin [35,36] or triethylborane [37,38] mediation. More recently, a very efficient desulfitative approach was reported starting from α-azidosulfonyl esters [26]. This approach is the best in terms of atom economy and efficiency, but it is less convenient to test the applicability of the method with a broad range of substituted radicals since every starting azide has to be prepared separately. The iodide approach remains very attractive in terms of availability of the starting material (the starting iodide and the azidating agents are either commercially available or easily prepared), and it can be easily used to introduce of variety of functional groups. Here, we report the extension of the carboazidation process for the preparation of azido-nitriles, -phosphonates, -phthalimides, and sulfones according to Scheme 1II. The reaction with sulfones is particularly attractive since it allows one to prepare homoallylic azides.

## 2. Results

**Scheme 2:**
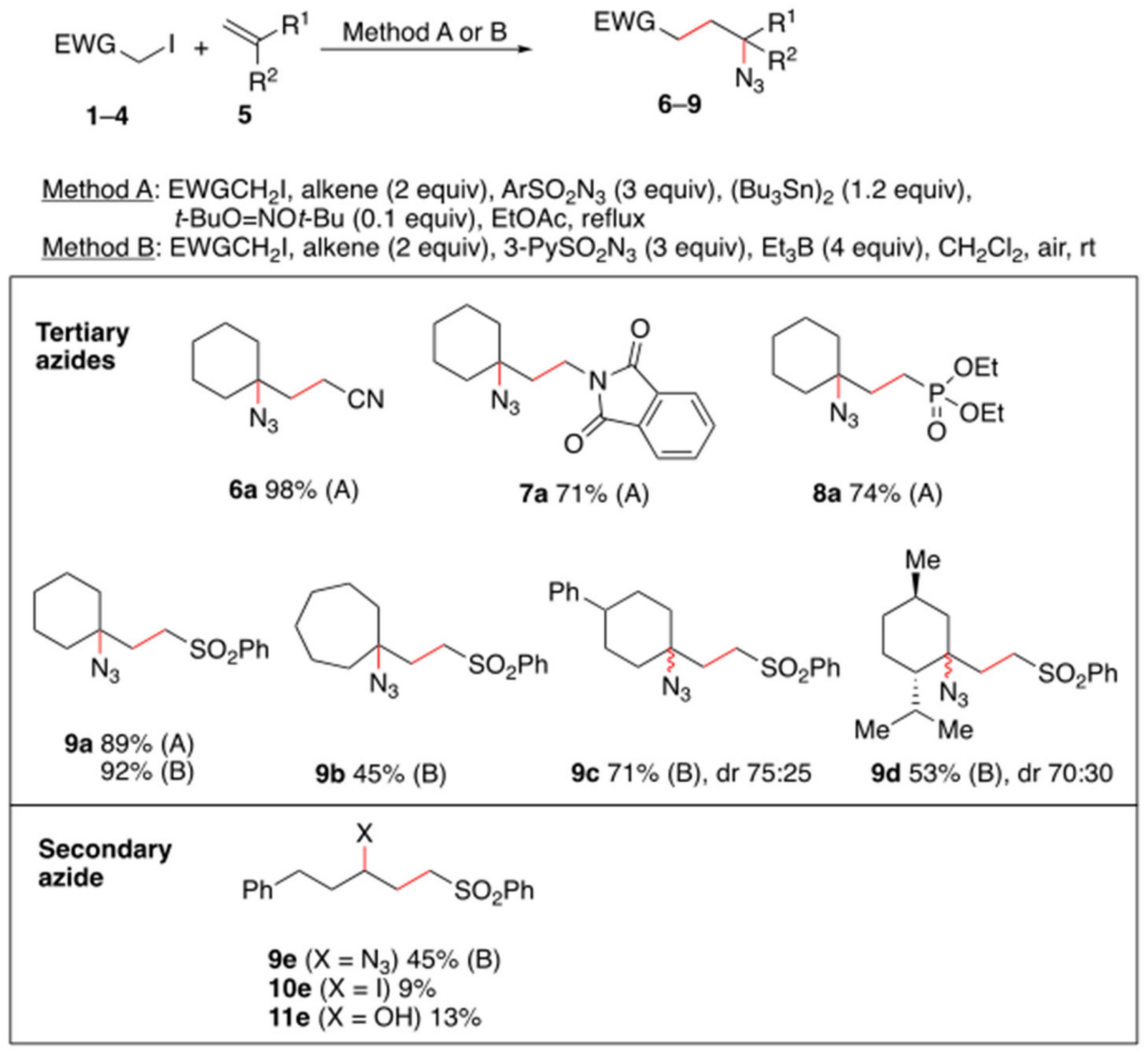
Radical carboazidation with cyano-, phthalimido-, diethoxylphosphonyl-, and benzenesulfonyl-substituted radicals.

Iodoacetonitrile **1**, *N*-iodomethylphthalimide **2**, diethyl iodomethanephosphonate **3**, and iodomethyl phenyl sulfones **4** are either commercially available or easily prepared (see supporting information). They were tested for the carboazidation of terminal alkenes **5** under ditin (A) or triethylborane (B) conditions (Scheme 2). Under ditin-mediated conditions A, reactions of **1**–**4** with methylenecyclohexane **5a** worked fine and provided the desired tertiary azide **6a**–**9a** in good yields. Azidonitrile **6a** is a potential precursor for 1,4-diamines, and azidophthalimide **7a** is a bis-protected 1,3-diamine. γ-Azidophosphonates such as **8a** are interesting precursors of γ-aminophosphonic acids, a well-established class of biologically active compounds [39]. Finally, the rich chemistry of sulfones renders γ-azidosulfones such as **9a** as potential precursors for a broad range of functionalized amines. The reaction of iodomethylsulfone **4** with **5a** mediated by Et_3_B (method B) provided the azidosulfones **9a** in an increased 92% yield. Sulfone **4** was also employed for the carboazidation of methylenecycloheptene **5b** and the two substituted methylenecyclohexanes **5c** and **5d** as well as the monosubstituted terminal alkene **5e** under conditions B. The tertiary azides **9b**–**9d** were obtained in moderate to good yields, and the level of stereoselectivity observed for **9c** and **9d** (2–3:1) corresponded to expectations [40]. The secondary azidosulfone **9e** was obtained in 45% yield under conditions B. The crude product was contaminated with the iodide **10e** (9%) and the alcohol **11e** (13%). The alcohol **13e** presumably resulted from a sulfone assisted hydrolysis of the iodide **10e**, but reaction of the intermediate radical with oxygen could not be excluded. When the reaction was run at a higher temperature according to method A, no azide **9e** was obtained, and the iodine atom transfer product **10e** (34% yield) was the only isolated product.

**Scheme 3:**
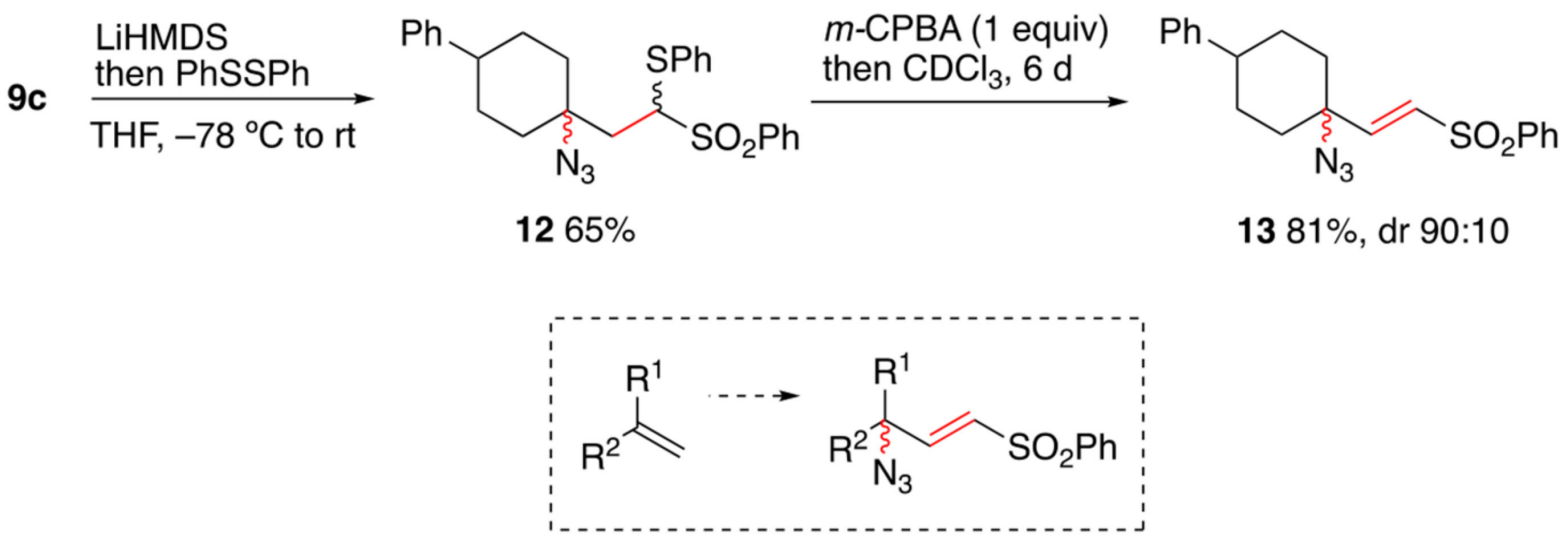
Preparation of unsaturated γ-azido vinyl sulfone **13** from the azidosulfone **9c**.

To illustrate the utility of γ-azidosulfones, compound **9c** was sulfurized to **12** by treatment with lithium hexamethyldisilazane (LiHMDS) and diphenyl disulfide (PhSSPh). The sulfide **12** was easily converted to the unsaturated γ-azido vinyl sulfone **13**, an attractive and versatile building block for synthesis, upon oxidation to the sulfoxide and standing in CDCl_3_ (Scheme 3). The whole reaction sequence allowed us to convert a terminal 2,2-disubsituted alkene into a tertiary 1-sufonylated allylic azide. Attempts to convert **12** into a β-azido ester upon treatment successively with *meta*-chloroperbenzoic acid (*m*-CPBA) and trifluoroacetic acid (TFA) to promote a Pummerer rearrangement according to a procedure reported by Barton and co-workers failed to give the desired product [41].

**Scheme 4:**
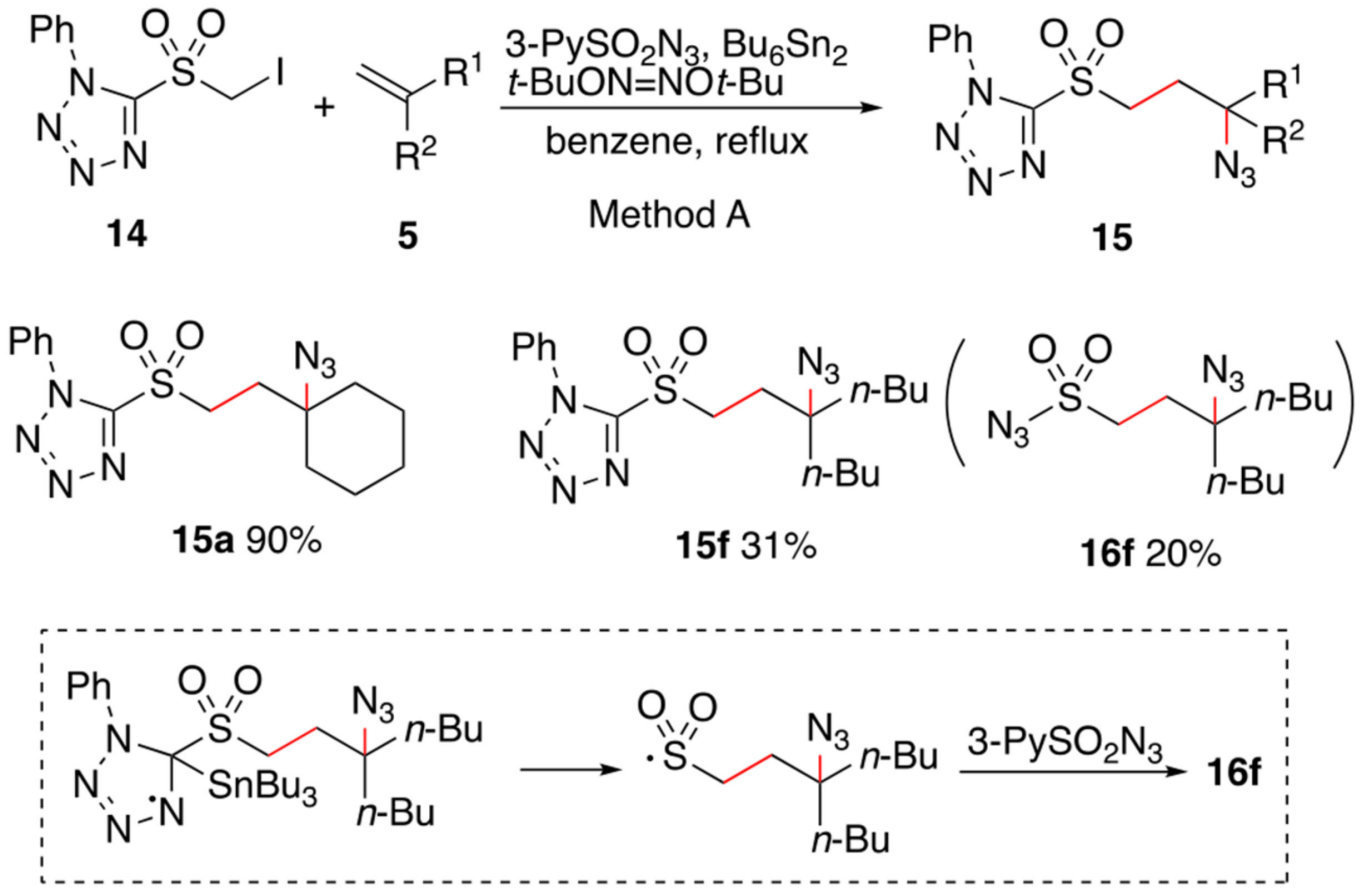
Tin mediated azidoalkylation with 1-phenyl-1*H*-tetrazole-5-yl iodomethyl sulfone **14.**

The carboazidation with sulfones also offers a potential approach for the preparation of homoallylic azides [42] by taking advantage of the Julia–Kocienski olefination process [43,44]. For this purpose, 1-phenyl-1*H*-tetrazole-5-yl iodomethyl sulfone **14** was prepared from the commercially available 1-phenyl-1*H*-tetrazole-5-thiol [45,46]. Carboazidation was then tested with methylene cyclohexene **5a** and 2-butyl-1-hexene **5f** using the ditin procedure (Scheme 4). With **5a**, the tertiary azide **15a** was obtained in high yield. The reaction with 2-butyl-1-hexene **5f** proved to be more challenging. The desired azide **15f** was isolated in 31% yield together with a side product identified as being **16f** in 20% yield. Compound **16f** most likely resulted from the ipso attack of a tin radical to the 1*H*-tetrazole-5-yl sulfone followed by reaction of the primary alkanesulfonyl radical with 3-pyridinesulfonyl azide. A related intermolecular ipso substitution was recently reported by Kamijo and co-workers [47].

**Scheme 5:**
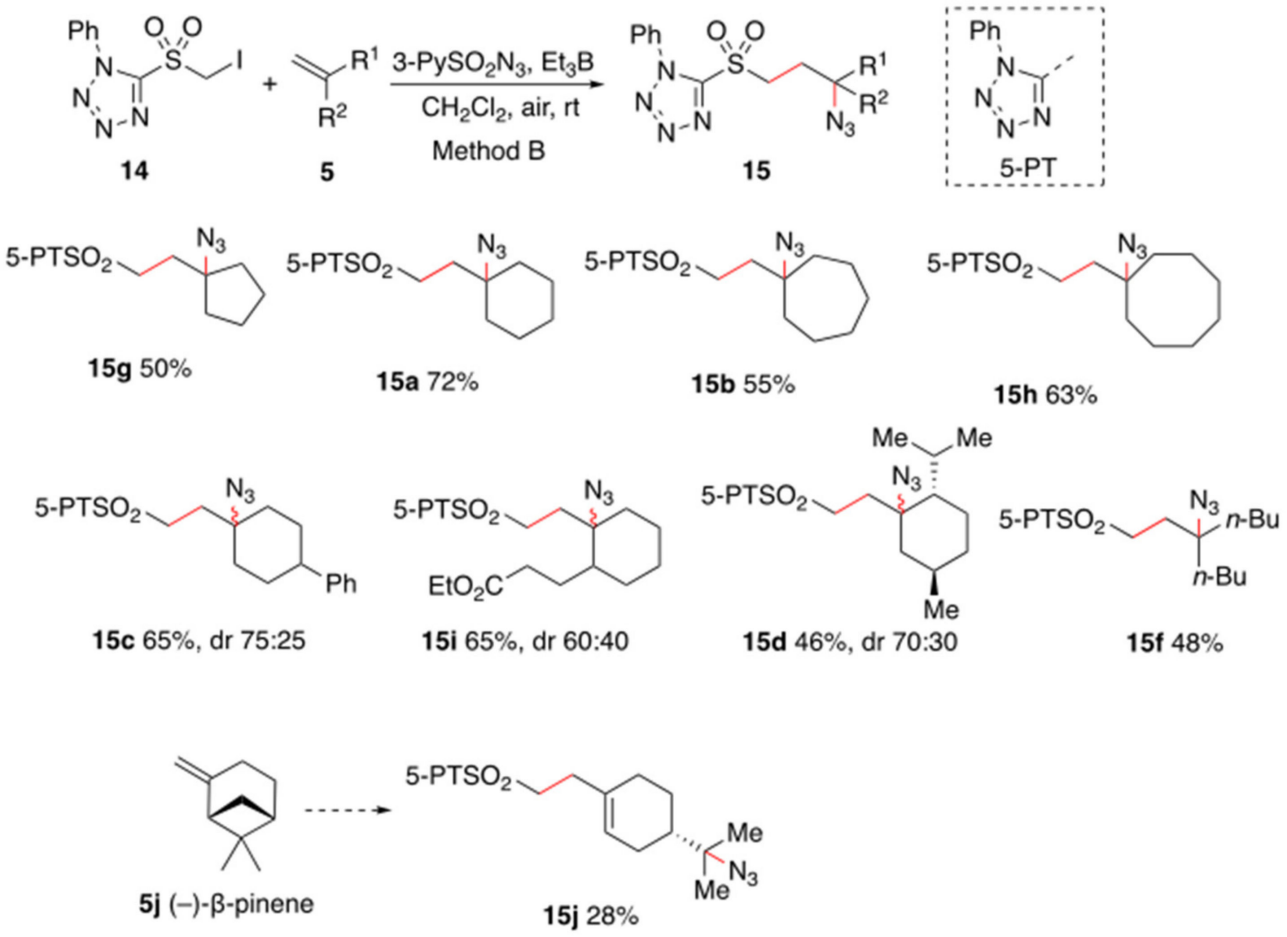

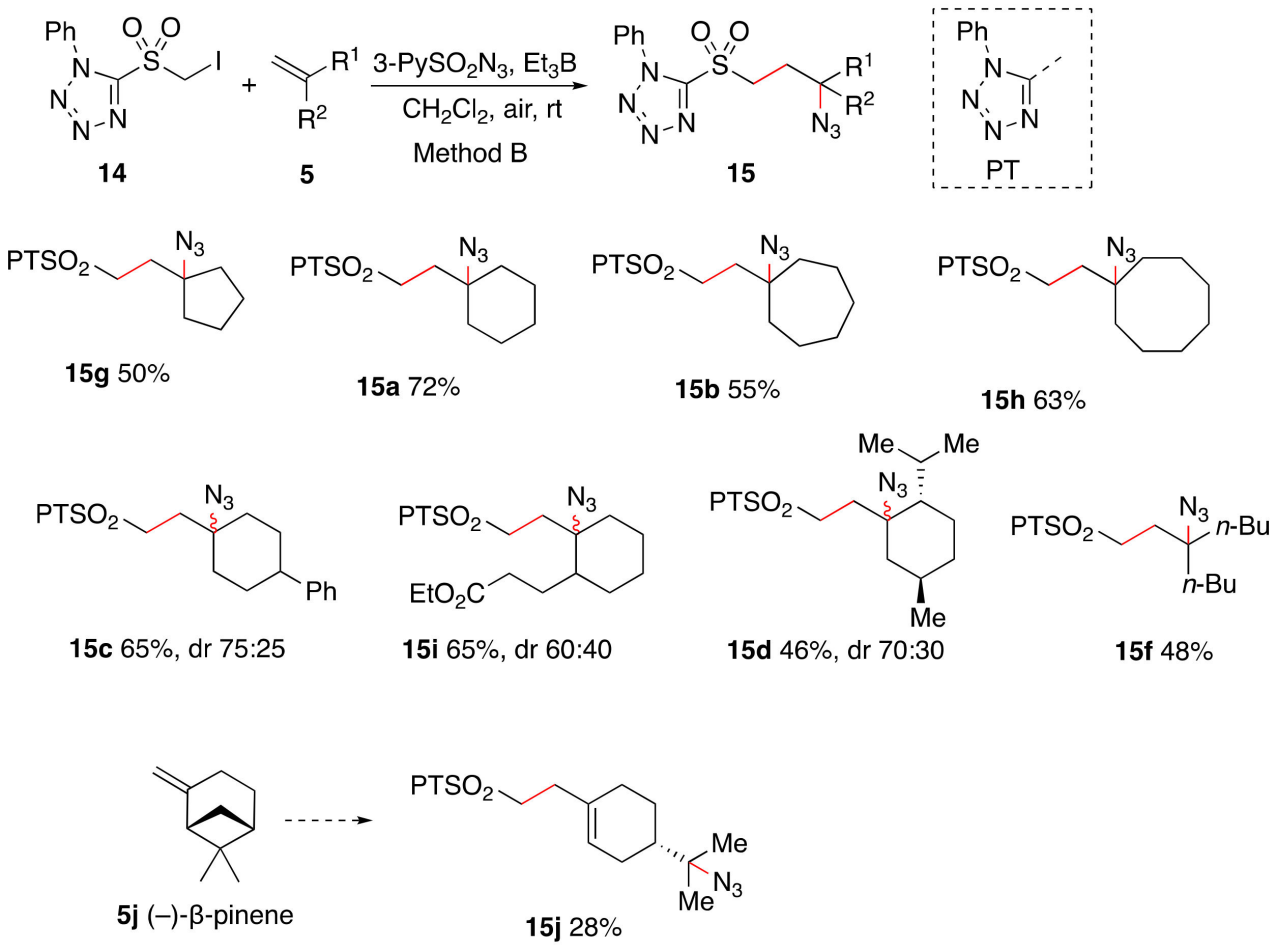
Et_3_B mediated azidoalkylation with 1-phenyl-1*H*-tetrazole-5-yl iodomethyl sulfone **14.**

Following this observation, all carboazidation reactions involving **14** and different alkenes **5** were using the Et_3_B method B. Results are summarized in Scheme 5, and moderate to good yields were observed for the formation of γ-azidosulfones **15** with a broad range of 2,2-substituted alkenes. No side product resulting from an ipso substitution at the tetrazole could be detected in those reactions. The radical nature of the process was demonstrated by formation of the ring-opening reaction product **15j** from (–)-β-pinene.

**Scheme 6:**
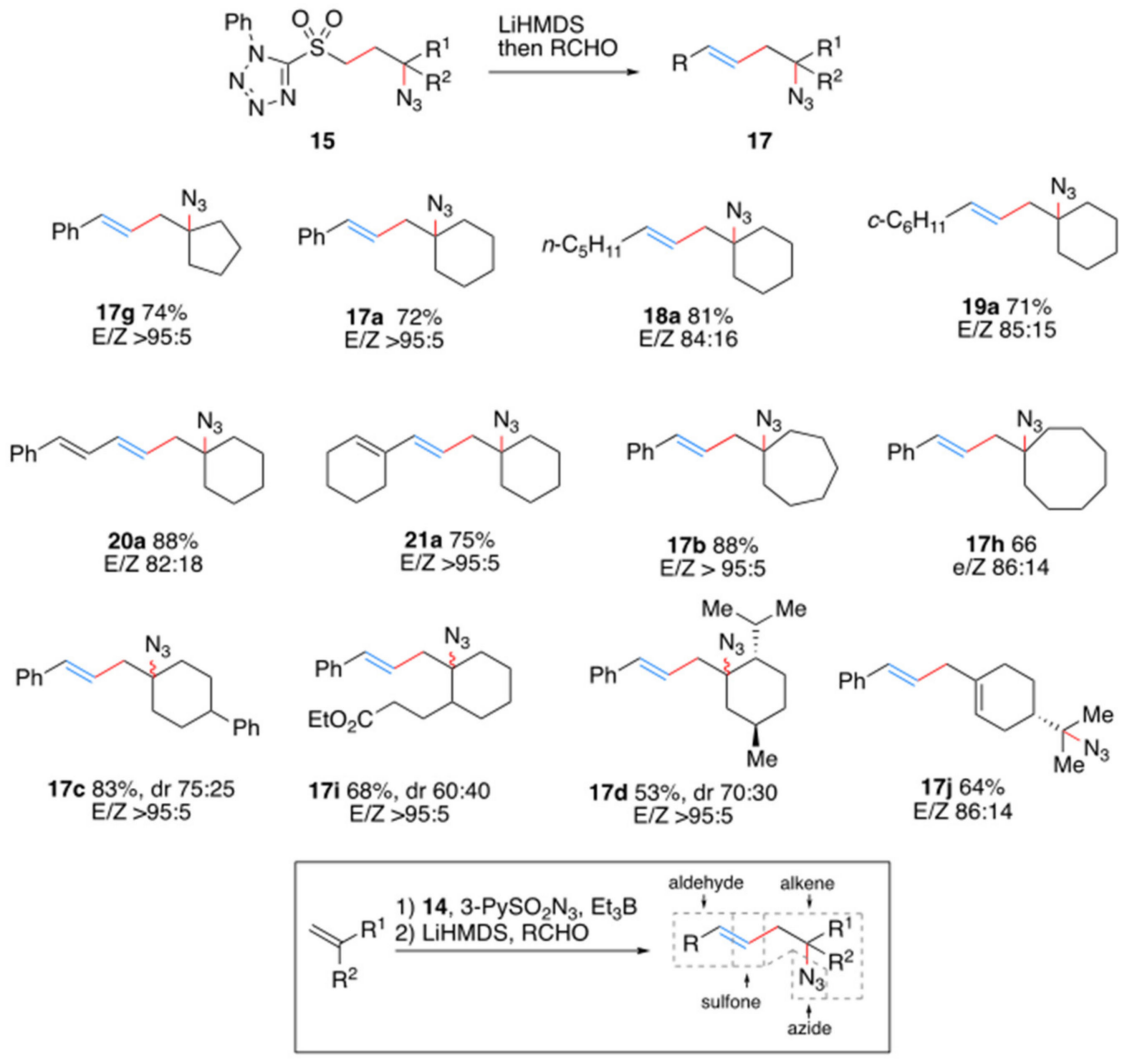
Julia–Kocienski olefination of γ-azidosulfones **15** with aldehydes, a formal 4-component azidovinylation of alkenes.

Finally, the 1-phenyl-1*H*-tetrazole sulfones **15** were submitted to the Julia–Kocienski olefination. Deprotonation of the sulfones **15** with LiHMDS followed by treatment with aldehydes afforded the homoallylic tertiary azides **17**–**21**. Moderate to good yields and high *E* selectivity were obtained with aromatic (**17**), aliphatic (**18**, **19**), and α,β-unaturated (**20**, **21**) aldehydes (Scheme 6). Interestingly, the homoallylic tertiary azides **17**–**21** were found to be stable and easily purified by column chromatography on silicagel.

## 3. Experimental Procedures

### 3.1. General Methods

All glassware was oven-dried at 160 °C and assembled hot or flame dried under vacuum, and allowed to cool under a nitrogen atmosphere. Unless otherwise stated, all the reactions were performed under a nitrogen atmosphere. For flash chromatography (FC) silica gel P60 (40–63 μm) (Silicycle, Basel, Switzerland) was used. Thin layer chromatography (TLC) was performed on silica gel F-254 plates (Silicycle, Basel, Switzerland) visualisation under UV (254 nm) or by staining. Staining solutions: (1) KMnO_4_ (1.5 g), K_2_CO_3_ (10 g) and NaOH 10% (1.25 mL) in H_2_O (200 mL); (2) ammonium molybdate tetrahydrate (50 g), CeSO_4_ (2 g) and conc. H_2_SO_4_ (100 mL) in H_2_O (900 mL); (3) *p*-anisaldehyde (3.7 mL), acetic acid (1.5 mL) and conc. H_2_SO_4_ (5 mL) in EtOH (135 mL). ^1^H and ^13^C NMR spectra were recorded on a Bruker Advance 300 (^1^H: 300.18 MHz, ^13^C: 75.48 MHz) (Bruker BioSpin AG, Fällanden, Switzerland). Chemical shifts (d) were reported in parts per million (ppm) with the residue solvent peak used as internal standard (CHCl_3_: d = 7.26 ppm, C_6_H_6_: d = 7.16 ppm and THF: d = 1.72 ppm for ^1^H NMR spectra and CHCl_3_: d = 77.00 ppm, C_6_H_6_: d = 128.00 ppm and THF: d = 67.21 ppm for ^13^C NMR spectra). Multiplicities were abbreviated as follows: s (singlet), d (doublet), t (triplet), q (quadruplet), m (multiplet) and br (broad). Coupling constants (*J*), are reported in Hz. ^13^C NMR measurements were run using a proton-decoupled pulse sequence. The number of carbon atoms for each signal is indicated only when more than one. High-resolution mass spectrometry (HRMS) analyses were measured on an Applied Biosystems Sciex QSTAR Pulsar (hybrid quadrupole time-of-flight mass spectrometer using electrospray ionisation (ESI) (Sciex, Baden, Switzelrand). Low resolution mass-spectrometry (LRMS) analyses were performed Finnigan Trace GC-MS (Thermo Scientific, Schlieren, Switzerland) (EI mode at 70 eV); GC column: Optima Delta 3 0.25 μm, 20 m, 0.25 mm (Macherey-Nagel, Oensingen, Switzerland). The infrared measurements were performed on a Jasco FTIR-460 Plus spectrometer equipped with a Specac MKII Golden Gate Single Reflection Diamond ATR System and are reported in wave numbers (cm^−1^). All reagents were obtained from commercial sources and used without further purification, unless otherwise mentioned. All reactions solvents (distilled THF, distilled Et_2_O, distilled dichloromethane, commercial toluene and benzene) were filtered over columns of activated alumina under a positive pressure of argon. Solvents for flash chromatography and extractions were of technical grade and were distilled prior to use. Hexamethyldisilazane (HMDS) was fractionally distilled under a nitrogen atmosphere before use. 1,2-Dichloroethane (DCE) was distilled over CaH_2_ under a nitrogen atmosphere.

### 3.2. General Procedures

**Hexabutylditin-mediated carboazidation (procedure A)**

Di-tert-butyl hyponitrite (DTBHN) [48] (0.1 equiv) was added in one portion to a solution of alkene (2–4 equiv), iodomethyl derivative (1 equiv), (Bu_3_Sn)_2_ (1.2 equiv), and ArSO_2_N_3_ [49] (3 equiv.) in benzene (0.5 M). The solution was stirred at 70 °C for 3 h. The crude mixture was directly purified by flash chromatography (FC) using KF/silica [50].

**Et_3_B-mediated carboazidation (procedure B)**

A 1 M solution of Et_3_B (3–4 equiv) was added at room temperature (rt) over 2 h via syringe pump to an open flask and then charged with a vigorously stirred mixture of alkene (2–4 equiv), iodomethyl derivative (1 equiv), and 3-PySO_2_N_3_ [49] (3 equiv) in solvent (0.66 M). Caution: the needle should be immersed into the reaction mixture in order to avoid direct contact of Et_3_B drops with air. The reaction vessel should be protected from direct light exposure by aluminum foil. After 1 h stirring, H_2_O and CH_2_Cl_2_ were added, and the layers were separated. The aqueous layer was extracted with CH_2_Cl_2_ (3×). The combined organic layers were washed with brine and dried over Na_2_SO_4_. The solvent was removed under reduced pressure, and the crude product was purified by FC.

**Julia–Kocienski olefination**

The phenyltetrazole sulfone derivative (1 equiv) was dissolved/diluted in THF (0.15 M) and cooled to −78 °C. A freshly prepared LiHMDS solution in THF (1.5 equiv) was added slowly and stirred for a further 30 min at −78 °C. Aldehyde (2 equiv) was added neat and stirred for a further 3 h at −78 °C. The reaction mixture was allowed to reach rt and was further stirred at rt overnight. H_2_O and Et_2_O were added to the reaction suspension, and the layers were separated. The aqueous phase was extracted with Et_2_O (3×). The combined organic layers were washed with brine and dried over Na_2_SO_4_. The solvent was removed under reduced pressure, and the crude product was purified by FC.

5-((2-(1-Azidocyclohexyl)ethyl)sulfonyl)-1-phenyl-1*H*-tetrazole (**15a**)

According to the procedure A from di-*tert*-butylhyponitrite (17 mg, 0.10 mmol), methylenecyclohexane **5a** (0.24 mL, 2.00 mmol), 5-((iodomethyl)sulfonyl)-1-phenyl-1*H*-tetrazole **14** (350 mg, 1.00 mmol), hexabutylditin (0.61 mL, 1.20 mmol), and 3-PySO_2_N_3_ (552 mg, 3.00 mmol) in benzene (2.0 mL). The crude mixture was directly purified by FC using KF/silica gel (cyclohexane/EtOAc, 95:5) to afford **15a** (325 mg, 90%).

According to the procedure B from a 1 M solution of Et_3_B in CH_2_Cl_2_ (4.00 mL, 4.00 mmol), methylenecyclohexane **5a** (0.24 mL, 2.00 mmol), 5-((iodomethyl)sulfonyl)-1-phenyl-1*H*-tetrazole **14**, (350 mg, 1.00 mmol), 3-PySO_2_N_3_ (552 mg, 3.00 mmol), and CH_2_Cl_2_ (0.50 mL). Purification by FC (cyclohexane/EtOAc, 95:5) afforded **15a** (260 mg, 72%). The NMR spectra of some compounds are in the Supplementary Materials.

Colorless crystals: m.p. 90.9–93.6 °C. ^1^H NMR (300 MHz, CDCl_3_): δ = 7.76–7.72 (m, 2H), 7.69–7.62 (m, 3H), 3.90–3.84 (m, 2H), 2.24–2.18 (m, 2H), 1.81–1.73 (m, 2H), 1.69–1.29 (m, 8H). ^13^C NMR (75 MHz, CDCl_3_): *δ* = 153.31, 132.96, 131.50, 129.75 (2C), 125.00 (2C), 62.45, 51.61, 34.41 (2C), 31.72, 25.07, 21.96 (2C). IR (neat): 2933, 2856, 2098, 1497, 1337, 1253, 1150. HRMS (ESI): calcd. for [M + H]^+^: C_15_H_20_N_7_O_2_S calcd 362.1394; found: 362.1400.

(3-(1-Azidocyclohexyl)prop-1-en-1-yl)benzene (**17a**)

According to the Julia–Kocienski procedure from **15a** (260 mg, 0.72 mmol), LiHMDS in THF (1.66 mL, 1.08 mmol), benzaldehyde (0.15 mL, 1.44 mmol), and THF (3.00 mL). Purification by FC (cyclohexane/EtOAc, 98:2) afforded the alkene **17a** as an inseparable mixture of isomers (141 mg, *E*/*Z* > 95:5, 81%). Colorless oil.

(*E*)-**17a** (major): ^1^H NMR (300 MHz, CDCl_3_): δ = 7.39–7.20 (m, 5H), 6.47 (d, *J* = 15.8 Hz, 1H), 6.24 (dt, *J* = 15.8, 7.4 Hz, 1H), 2.46 (dd, *J* = 7.4, 1.2 Hz, 2H), 1.72 (d, *J* = 13.1 Hz, 2H), 1.65–1.39 (m, 7H), 1.32–1.21 (m, 1H). ^13^C NMR (75 MHz, CDCl_3_): = 137.24, 133.74, 128.51 (2C), 127.29, 126.17 (2C), 124.36, 64.22, 43.93, 34.52 (2C), 25.33, 22.07 (2C).

Characteristic signals for (Z)-**17a** (minor): ^1^H NMR (300 MHz, CDCl_3_): δ = 2.55 (d, *J* = 5.8 Hz, 2H). IR (neat): 3027, 2931, 2858, 2096, 1495, 1447, 1254, 1138, 1102, 1029. EI-MS m/z (%): M–N_2_: 213.3 (21), 198.3 (7), 170.3 (20), 156.3 (16), 128.3 (10), 117.3 (100), 115.3 (73), 96.3 (63), 91.3 (40), 69.3 (34), 55.3 (39). HRMS (ESI): calcd. for [M + H]^+^: C_15_H_20_N_3_: 242.1652; found: 242.1655.

## 4. Conclusions

In conclusion, we demonstrated that the azidoalkylation of terminal alkenes is not limited to α-iodoester and α-iodoketones. The reaction also works well with nitriles, phosphonates, phthalimides, and aryl sulfones. This last class of compounds is particularly interesting in terms of potential synthetic applications. This point was illustrated by the preparation of homoallylic azides by merging the azidoalkylation process with a Julia–Kocienski olefination reaction. Recently, 1-phenyl-1*H*-tetrazole sulfones have also been shown to be privileged substrates for reductive cross-coupling processes, opening new opportunities for further functionalization [51,52].

## Supplementary Materials

Detailed experimental procedures and NMR spectra of all compounds are available online at https://www.mdpi.com/1420-3049/24/22/4184/s1.
