# Supplementary material for: Two-Step Azidoalkenylation of Terminal Alkenes Using Iodomethyl Sulfones
Source: Molecules. 2019 Nov 18;24(22):4184. doi: 10.3390/molecules24224184 (PMC6891591; doi:10.3390/molecules24224184)
Supplement: Supplementary file 1 [file molecules-24-04184-s001.pdf]

Supporting Information

Two-step Azidoalkenylation of Terminal Alkenes using Iodomethyl Sulfones

Nicolas Millius, Guillaume Lapointe, Philippe Renaud*

University of Bern, Department of Chemistry and Biochemistry, Freiestrasse 3, CH-3012 Bern, Switzerland;

*Correspondence: philippe.renaud@dcb.unibe.ch

General Information

All glassware was oven-dried at 160 °C and assembled hot or flame dried under vacuum, and allowed to cool under a nitrogen atmosphere. Unless otherwise stated, all the reactions were performed under a nitrogen atmosphere. For flash chromatography (FC) silica gel P60 (40-63 μm, *Silicycle*) was used. Thin layer chromatography (TLC) was performed on *Silicycle* F-254 plates; visualisation under UV (254 nm) or by staining. Staining solutions: 1) $KMnO_4$ (1.5 g), K_2CO_3 (10 g) and NaOH 10% (1.25 mL) in H_2O (200 mL); 2) ammonium molybdate tetrahydrate (50 g), $CeSO_4$ (2 g) and conc. H_2SO_4 (100 mL) in H_2O (900 mL); 3) *p*-anisaldehyde (3.7 mL), acetic acid (1.5 mL) and conc. H_2SO_4 (5 mL) in EtOH (135 mL).

Instrumentation

^1H and ^{13}C NMR spectra were recorded on a Bruker Advance 300 (^1H: 300.18 MHz, ^{13}C: 75.48 MHz). Chemical shifts (δ) were reported in parts per million (ppm) with the residue solvent peak used as internal standard ($CHCl_3$: $\delta = 7.26$ ppm, C_6H_6: $\delta = 7.16$ ppm and THF: $\delta = 1.72$ ppm for ^1H NMR spectra and $CHCl_3$: $\delta = 77.00$ ppm, C_6H_6: $\delta =$ 128.00 ppm and THF: $\delta = 67.21$ ppm for ^{13}C NMR spectra). Multiplicities were abbreviated as follows: s (singlet), d (doublet), t (triplet), q (quadruplet), m (multiplet) and br (broad). Coupling constants (*J*), are reported in Hz. ^{13}C NMR measurements were run using a proton-decoupled pulse sequence. The number of carbon atoms for each signal is indicated only when more than one. HRMS analyses were measured on an *Applied Biosystems* Sciex QSTAR Pulsar (hybrid quadrupol time-of-flight mass spectrometer) using electrospray ionisation (ESI). LRMS analyses were taken from GC-MS (*Finnigan* Trace MS, EI mode at 70 eV; GC column: Macherey-Nagel Optima Delta 3-0.25 μm, 20m, 0.25 mm) analysis. The infrared measurements were performed on a *Jasco* FT-IR-460 Plus spectrometer equipped with a Specac MKII Golden Gate Single Reflection Diamond ATR System and are reported in wave numbers (cm^{-1}).

Materials and Methods

All reagents were obtained from commercial sources and used without further purification, unless otherwise mentioned. All reactions solvents (distilled THF, distilled Et_2O, distilled dichloromethane, commercial toluene and benzene) were filtered over columns of activated alumina under a positive pressure of argon. Solvents for flash chromatography and extractions were of technical grade and were distilled prior to use. Hexamethyldisilazane (HMDS) was fractionally distilled under a nitrogen atmosphere before use. 1,2-Dichloroethane (DCE) was distilled over CaH_2 under a nitrogen atmosphere.

Silica with KF: 10 w/w% KF was grinded to a powder, blended with the silica and used as stationary phase for the flash chromatography (FC). This KF/silica separation system was used to have a better separation when tin compounds were used.[1]

Solid loading for flash chromatography (FC): the crude product was suspended in silica and the required solvent for the mobile phase. The solvent was removed under reduced pressure and the remaining solid was loaded on the column.

Preparation of Et_3B solutions: Commercial pure Et_3B ($\geq$ 95%) was dissolved in the degassed solvent of choice.

General Procedure A (tin-mediated carboazidation):

DTBHN[2] (0.10 equiv.) was added in one portion to a solution of alkene (2.00 to 4.00 equiv.), iodomethyl derivative (1.00 equiv.), $(Bu_3Sn)_2$ (1.20 equiv.) and 3-$PySO_2N_3$[3] (3.00 equiv.) in benzene (0.5 M). The solution was stirred at 70 °C for 3 h. The crude mixture was directly purified by FC using KF/silica.

General Procedure B (Et_3B-mediated carboazidation):

A 1 M solution of Et_3B (3.00 to 4.00 equiv.) was added at rt over 2 h *via* syringe pump to an open flask, charged with a vigorously stirred mixture of alkene (2.00 to 4.00 equiv.), iodomethyl derivative (1.00 equiv.) and 3-$PySO_2N_3$[3] (3.00 equiv.) in solvent

(0.66 M). *Caution: the needle should be immersed into the reaction mixture in order to avoid a direct contact of Et_3B drops with air. The reaction vessel should be protected from direct light exposure by aluminium foil.* After 1 h of extra stirring, H_2O and DCM were added and the layers were separated. The aqueous layer was extracted with DCM (3 ×). The combined organic layers were washed with brine and dried over Na_2SO_4. The solvent was removed under reduced pressure and the crude product was purified by FC.

General Procedure C (Wittig olefination):

$MePPh_3Br$ (1.20 to 2.00 equiv.) was suspended in Et_2O or THF. *t*-BuONa (1.20 to 2.00 equiv.) was slowly added and the stirring maintained for 1 h at rt. The ketone (1.00 equiv.) was dissolved/diluted in Et_2O or THF, added to the reaction suspension and stirred at rt overnight. H_2O was added and the phases were separated. The aqueous phase was extracted with Et_2O (3 ×). The combined organic phases were washed with brine and dried over Na_2SO_4. The solvent was removed under reduced pressure. The crude product was purified by FC with solid loading.

General procedure D (Julia/Kocienski olefination):

The phenyltetrazole sulfone derivative (1.00 equiv.) was dissolved/diluted in THF (0.15 M) and cooled to –78 °C. A freshly prepared LiHMDS solution in THF (1.50 equiv.) was added slowly and stirred for further 30 min at –78 °C. Aldehyde (2.00 equiv.) was added neat and stirred for further 3 h at –78 °C. The reaction mixture was allowed to reach rt and further stirred at rt overnight. H_2O and Et_2O were added to the reaction suspension and the layers were separated. The aqueous phase was extracted with Et_2O (3 ×). The combined organic layers were washed with brine and dried over Na_2SO_4. The solvent was removed under reduced pressure. The crude product was purified by FC.

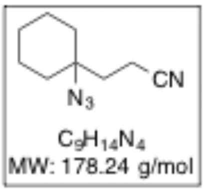

3-(1-Azidocyclohexyl)propanenitrile (**6a**). According to the general procedure A with di-*tert*-butylhyponitrite[2] (17 mg, 0.1 mmol), methylenecyclohexane **5a** (0.25 mL, 2.00 mmol), 3-$PySO_2N_3$[3] (552 mg, 3.00 mmol), hexabutyldistannane (0.61 mL, 1.20 mmol) and iodoacetonitrile **1** (167 mg, 1.00 mmol) in EtOAc (2.0 mL). The crude mixture was directly purified by flash column chromatography (*n*-pentane/Et_2O, 90:10) using 10% KF/ 90% silica gel as stationary phase to afford **6a** (158 mg, 89%). Yellow oil: ^1H NMR (300 MHz, $CDCl_3$): δ = 2.43 (t, *J* = 7.8 Hz, 2H), 1.92 (t, *J* = 7.8 Hz, 2H), 1.75 - 1.65 (m, 2H), 1.64 - 1.51 (m, 5H), 1.48 - 1.28 (m, 3H). ^{13}C NMR (75 MHz, $CDCl_3$): δ = 119.56, 62.62, 35.26, 34.15 (2C), 25.13, 21.96 (2C), 11.68. IR (neat): 2933, 2860, 2095, 1449, 1253. HRMS (ESI): calcd. for $M+H^+$: $C_9H_{15}N_4$ 179.1291; found 179.1296.

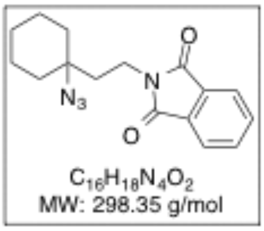

2-(2-(1-Azidocyclohexyl)ethyl)phtalimide (**7a**). According to the general procedure A with di-*tert*-butylhyponitrite[2] (17 mg, 0.1 mmol), methylenecyclohexane **5a** (0.25 mL, 2.00 mmol), $PhSO_2N_3$[4] (550 mg, 3.00 mmol), hexabutyldistannane (0.61 mL, 1.20 mmol) and iodomethylphthalimide **2**[5] (280 mg, 1.00 mmol) in EtOAc (2.0 mL). The crude mixture was directly purified by flash column chromatography (*n*-pentane/Et_2O, 90:10) using 10% KF/ 90% silica gel as stationary phase to afford **7a** (213 mg, 71%). Colourless crystals: M. p.: 59.0 - 59.8 °C. ^1H NMR (300 MHz, $CDCl_3$): δ = 7.85 (dd, *J* = 5.5, 3.0 Hz, 2H), 7.71 (dd, *J* = 5.5, 3.0 Hz, 2H), 3.81 (dd, *J* = 8.0, 5.4 Hz, 2H), 1.92 (dd, *J* = 8.0, 5.4 Hz, 2H), 1.82 - 1.71 (m, 2H), 1.65 - 1.43 (m, 7H), 1.38 - 1.25 (m, 1H). ^{13}C NMR (75 MHz, $CDCl_3$): δ= 168.04 (2C), 133.87 (2C), 132.11 (2C), 123.15 (2C), 62.73, 37.45, 34.36 (2C), 33.08, 25.26, 21.98 (2C). IR (neat): 2932, 2860, 2098, 1710, 1399, 1373, 1257. HRMS (ESI): calcd. for $M+Na^+$: $C_{16}H_{18}N_4O_2Na$: 321.1322; found: 321.1327.

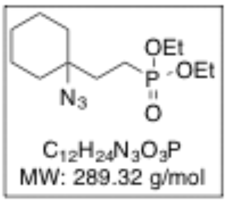

Diethyl (2-(1-azidocyclohexyl)ethyl)phosphonate (**8a**). According to the general procedure A with di-*tert*-butylhyponitrite[2] (17 mg, 0.1 mmol), methylenecyclohexane **5a** (0.25 mL, 2.00 mmol), $PhSO_2N_3$[4] (550 mg, 3.00 mmol), hexabutyldistannane (0.61 mL, 1.20 mmol)

and iodomethylphosphonate **3** (280 mg, 1.00 mmol) in EtOAc (2.0 mL). The crude mixture was directly purified by flash column chromatography (cyclohexane/EtOAc, 50:50) using 10% KF/ 90% silica gel as stationary phase to afford **8a** (215 mg, 74%). Yellow oil: ^{1}H NMR (300 MHz, $CDCl_3$): δ = 4.21 - 4.00 (m, 4H), 1.87 - 1.77 (m, 3H), 1.74 - 1.49 (m, 8H), 1.42 - 1.24 (m, 3H), 1.33 (t, J = 7.1 Hz, 6H). ^{13}C NMR (75 MHz, $CDCl_3$): δ = 63.27 (d, J = 17.2 Hz), 61.53 (d, J = 6.5 Hz, 2C), 34.06 (2C), 32.30 (d, J = 4.0 Hz), 25.18, 21.89 (2C), 19.75 (d, J = 142.52 Hz), 16.29 (d, J = 6.0 Hz, 2C). ^{31}P NMR: (121 MHz, H_3PO_4 in $CDCl_3$): δ = 32.21. IR (neat): 2979, 2933, 2861, 2097, 1253, 1055, 1019, 954. HRMS (ESI): calcd. for M+Na$^+$: $C_{12}H_{24}N_3O_3NaP$: calcd 312.1447; found: 312.1452.

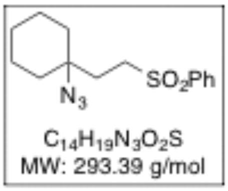

((2-(1-Azidocyclohexyl)ethyl)sulfonyl)benzene (**9a**). Tin-mediated Carboazidation: According to the general procedure A with di-*tert*-butylhyponitrite[2] (17 mg, 0.1 mmol), methylenecyclohexane **5a** (0.25 mL, 2.00 mmol), 3-$PySO_2N_3$[3] (552 mg, 3.00 mmol), hexabutyldistannane (0.61 mL, 1.20 mmol) and ((iodomethyl)sulfonyl)benzene **4**[6] (282 mg, 1.00 mmol) in EtOAc (2.0 mL). The solution was warmed to 70 °C for 3 h and the crude mixture was directly purified by flash column chromatography (cyclohexane/EtOAc, 90:10) using 10% KF/ 90% silica gel as stationary phase to afford **9a** (261 mg, 89%). Et_3B-mediated Carboazidation: According to the general procedure B with a 1 M solution of Et_3B in EtOH (4.00 mL, 4.00 mmol), methylenecyclohexane **5a** (0.25 mL, 2.00 mmol), 3-$PySO_2N_3$[3] (552 mg, 3.00 mmol) and ((iodomethyl)sulfonyl)benzene **4**[6] (282 mg, 1.00 mmol) in EtOH/H_2O (1:1, 1.0 mL). Purification by FC (cyclohexane/EtOAc, 90:10) to afford **9a** (282 mg, 92%). Colourless oil: ^{1}H NMR (300 MHz, $CDCl_3$): δ = 7.93 (d, J = 7.3 Hz, 2H), 7.68 (tt, J = 7.3, 1.4 Hz, 1H), 7.59 (tt, J = 7.3, 1.4 Hz, 2H), 3.22 - 3.14 (m, 2H), 1.98 - 1.90 (m, 2H), 1.68 - 1.46 (m, 7H), 1.42 - 1.24 (m, 3H). ^{13}C NMR (75 MHz, $CDCl_3$) δ = 138.78, 133.76, 129.25 (2C), 127.85 (2C), 62.45, 51.18, 34.29 (2C), 31.89, 24.98, 21.81 (2C). IR (neat): 2932, 2859, 2096, 1446, 1300, 1252, 1146, 1086. HRMS (ESI): calcd. for M+Na$^+$: $C_{14}H_{19}N_3O_2NaS$: 316.1090; found: 316.1098.

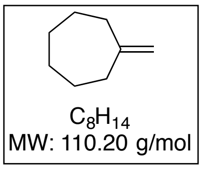

Methylenecycloheptane (**5b**). According to the general procedure C with cycloheptanone (5.89 g, 50.00 mmol), $MePPh_3Br$ (21.43 g, 60.00 mmol), *t*-BuONa (5.77 g, 60.00 mmol) and Et_2O (320 mL). Purification by FC with solid loading (*n*-pentane, 100%). Colourless liquid **5b** (2.80 g, 51%, yield corrected from solvent residue). The spectral data were in accordance with the literature.[7] ^1H NMR (300 MHz, $CDCl_3$): δ = 4.68 (d, J = 0.9 Hz, 2H), 2.30 - 2.26 (m, 4H), 1.59 - 1.51 (m, 8H). ^{13}C NMR (75 MHz, $CDCl_3$): δ = 152.27, 110.26, 36.18 (2C), 29.47 (2C), 28.40 (2C).

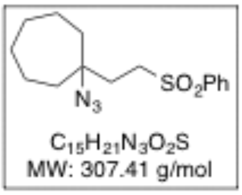

1-Azido-1-(2-(phenylsulfonyl)ethyl)cycloheptane (**9b**). According to the general procedure B with a 1 M solution of Et_3B in CH_2Cl_2 (4.00 mL, 4.00 mmol), methylenecycloheptane **5b** (220 mg, 2.00 mmol), 3-$PySO_2N_3$[3] (552 mg, 3.00 mmol) and ((iodomethyl)sulfonyl)benzene **4**[6] (282 mg, 1.00 mmol) in CH_2Cl_2 (1.0 mL). Purification by FC (*n*-pentane/Et_2O, 70:30) to afford **9b** (139 mg, 45%). Colourless crystals: M. p.: 42.6 – 45.3 °C. ^1H NMR (300 MHz, $CDCl_3$): δ = 7.94 – 7.91 (m, 2H), 7.68 (tt, J = 7.4 Hz, 1.4 Hz, 1H), 7.59 (tt, J = 7.4 Hz, 1,4 Hz, 2H), 3.17 (m, 2H), 1.92 (m, 2H), 1.63 – 1.36 (m, 12H). ^{13}C NMR (75 MHz, $CDCl_3$): δ = 139.00, 133.82, 129.39 (2C), 127.98 (2C), 66.04, 51.76, 38.08 (2C), 32.80, 29.29 (2C), 22.41 (2C). IR (neat): 2935, 2861, 2095, 1446, 1305, 1258, 1149, 1087, 740, 699. HRMS (ESI): calcd. for $M+Na^+$: $C_{15}H_{21}O_2N_3NaS$: 330.1247; found: 330.1248.

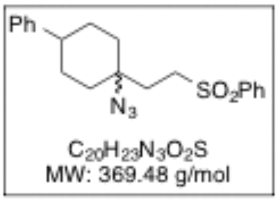

4-Azido-4-(2-(phenylsulfonyl)ethyl)cyclohexyl)benzene (**9c**). According to the general procedure B with a 1 M solution of Et_3B in CH_2Cl_2 (12.00 mL, 12.00 mmol), (4-methylenecyclohexyl)benzene **5c**[8] (1.04 g, 6.00 mmol), 3-$PySO_2N_3$[3] (1.66 g, 9.00 mmol)) and ((iodomethyl)sulfonyl)benzene **4**[6] (846 mg, 3.00 mmol) in CH_2Cl_2 (1.5 mL). Purification by FC (*n*-pentane/Et_2O/CH_2Cl_2, 60:30:10) to afford **9c** as a mixture of diastereomers (831 mg, *cis*/*trans* 75:25, 71%). White solid: M. p. = 149.7 - 153.2 °C. **9c-major**: ^1H NMR (300 MHz, $CDCl_3$): δ = 7.95 (m, 2H), 7.70

(t, J = 7.4 Hz, 1H), 7.61 (t, J = 7.4 Hz, 2H), 7.32 - 7.26 (m, 3H), 7.21 - 7.15 (m, 2H), 3.27 - 3.21 (m, 2H), 2.52 - 2.43 (m, 1H), 2.04 - 1.98 (m, 2H), 1.90 - 1.64 (m, 6H), 1.50 - 1.40 (m, 2H). ^{13}C NMR (75 MHz, $CDCl_3$): δ = 145.95, 138.91, 133.91, 129.45 (2C), 128.45 (2C), 128.01 (2C), 126.71 (2C), 126.32, 61.93, 51.40, 43.30, 34.78 (2C), 33.72, 29.25 (2C). Characteristic signals for **9c-minor**: ^{1}H NMR (300 MHz, $CDCl_3$): δ = 3.32 - 3.15 (m, 2H), 2.11 - 2.07 (m, 2H). ^{13}C NMR (75 MHz, $CDCl_3$): δ = 145.09, 138.99, 133.85, 128.52 (2C), 126.76, 126.69 (2C), 62.03, 51.35, 42.94, 34.23 (2C), 33.63, 29.77 (2C). IR (neat): 2932, 2860, 2092, 1446, 1306, 1258, 1149, 1087. HRMS (ESI): calcd. for M+Na$^+$: $C_{20}H_{23}O_2N_3NaS$: 392.1403; found: 392.1400.

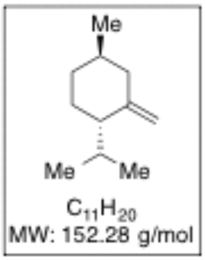

(1S,4R)-1-Isopropyl-4-methyl-2-methylenecyclohexane (**5d**). According to the general procedure C with (–)-menthone (1.71 g, 10.00 mmol), $MePPh_3Br$ (7.15 g, 20.00 mmol), *t*-BuONa (1.92 g, 20.00 mmol) and Et_2O (32 mL). Purification by FC with solid loading (cyclohexane/EtOAc, 95:5). Colourless liquid (1*S*,4*R*)-1-Isopropyl-4-methyl-2-methylenecyclohexane **5d** (1.12 g, 74%). The spectral data were in accordance with the literature.[9] Colourless liquid: ^{1}H NMR (300 MHz, $CDCl_3$): δ = 4.70 (s, 1H), 4.59 (s, 1H), 2.29 (dd, J = 12.4, 3.7 Hz, 1H), 1.96 (dq, J = 13.2, 6.6 Hz, 1H), 1.80 - 1.73 (m, 2H), 1.68 -1.63 (m, 2H), 1.61 - 1.53 (m, 1H), 1.23 - 1.02 (m, 2H), 0.93 - 0.88 (m, 9H). ^{13}C NMR (75 MHz, $CDCl_3$): δ = 151.13, 106.12, 49.42, 44.41, 34.00, 33.38, 27.10, 27.09, 22.09, 21.36, 19.01.

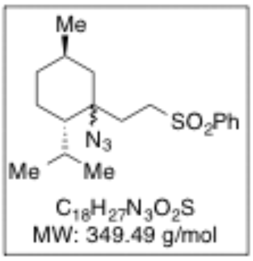

((2-((2S,5R)-1-Azido-2-isopropyl-5-methylcyclohexyl)ethyl)sulfonyl)benzene (**9d**).). According to the general procedure B with a 1 M solution of Et_3B in CH_2Cl_2 (4.00 mL, 4.00 mmol), (1*S*,4*R*)-1-Isopropyl-4-methyl-2-methylenecyclohexane **5d** (305 mg, 2.00 mmol), $3\text{-}PySO_2N_3$[3] (552 mg, 3.00 mmol)) and ((iodomethyl)sulfonyl)benzene **4**[6] (282 mg, 1.00 mmol) in CH_2Cl_2 (0.5 mL). Purification by FC (cyclohexane/EtOAc, 95:5) to afford **9d** (187 mg, dr 7:3,

53%). Colourless crystals: M. p. = 97.9 - 98.0 °C. **9d-major**: ^{1}H NMR (300 MHz, $CDCl_3$): δ = 7.96 - 7.90 (m, 2H), 7.70 - 7.56 (m, 3H), 3.22 - 2.92 (m, 2H), 2.12 (dd, J = 11.6, 5.4 Hz, 1H), 2.04 - 1.88 (m, 2H), 1.79 - 1.65 (m, 4H), 1.46 - 1.32 (m, 2H), 1.27 - 1.07 (m, 2H), 0.96 (d, J = 6.9 Hz, 3H), 0.89 (d, J = 6.9 Hz, 3H), 0.82 - 0.78 (m, 3H). ^{13}C NMR (75 MHz, $CDCl_3$): δ = 139.00, 133.79, 129.33 (2C), 128.02 (2C), 67.19, 51.54, 49.71, 42.25, 34.41, 28.90, 26.14, 23.97, 23.88, 23.72, 22.08, 19.40. Characteristic signals for **9d-minor**: ^{1}H NMR (300 MHz, $CDCl_3$): δ = 1.99 - 1.78 (m, 2H). ^{13}C NMR (75 MHz, $CDCl_3$): δ = 133.96, 129.45 (2C), 128.08 (2C), 67.15, 48.36, 34.73, 28.23, 25.56, 22.12, 19.43, 17.34. IR (neat): 2954, 2930, 2868, 2096, 1447, 1303, 1258, 1148, 1087. HRMS (ESI): calcd. for M–H$^+$: $C_{18}H_{26}O_2N_3S$: 348.1740; found: 348.1739.

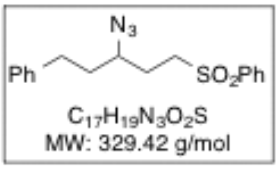

(3-Azido-5-(phenylsulfonyl)pentyl)benzene (**9e**). According to the general procedure B with a 1 M solution of Et_3B in CH_2Cl_2 (4.00 mL, 4.00 mmol), 4-phenyl-1-butene **5e** (0.30 mL, 2.00 mmol), 3-$PySO_2N_3$[3] (552 mg, 3.00 mmol)) and ((iodomethyl)sulfonyl)benzene **4**[6] (282 mg, 1.00 mmol) in CH_2Cl_2 (0.5 mL). Purification by FC (cyclohexane/EtOAc, 95:5) to afford **9e** (145 mg, 45%), **10e** (38 mg, 9%) and **11e** (40 mg, 13%). **9e** colourless oil: ^{1}H NMR (300 MHz, $CDCl_3$): δ = 7.91 (d, J = 7.4 Hz, 2H), 7.68 (tt, J = 7.4, 1.3 Hz, 1H), 7.58 (t, J = 7.4 Hz, 2H), 7.34 - 7.13 (m, 5H), 3.46 - 3.34 (m, 1H), 3.31 - 3.08 (m, 2H), 2.84 - 2.59 (m, 2H), 2.13 - 1.98 (m, 1H), 1.92 - 1.76 (m, 3H). ^{13}C NMR (75 MHz, $CDCl_3$): δ = 140.36, 138.95, 133.86, 129.39 (2C), 128.49 (2C), 128.29 (2C), 127.93 (2C), 126.27, 60.23, 52.94, 36.00, 32.00, 27.52. IR (neat): 2927, 2099, 1446, 1307, 1149, 1087. HRMS (ESI): calcd. for M+Na$^+$: $C_{17}H_{19}N_3O_2SNa$: 352.1090; found: 352.1080.

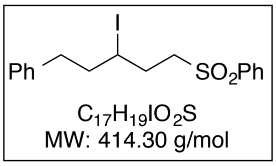

(3-Iodo-5-(phenylsulfonyl)benzene (**10e**). Yellowish oil. ^{1}H NMR (300 MHz, $CDCl_3$): δ = 7.96 - 7.94 (m, 2H), 7.74 - 7.71 (m, 1H), 7.65 - 7.61 (m, 2H), 7.37 - 7.20 (m, 5H), 4.05 - 3.99 (m, 1H), 3.46 - 3.38 (m, 1H), 3.28 - 3.20 (m, 1H), 2.99 - 2.87 (m, 1H), 2.99 - 2.87 (m, 1H), 2.29 - 2.16 (m, 3H), 2.03 - 1.94 (m, 1H). ^{13}C NMR (75 MHz, $CDCl_3$): δ = 140.03, 138.98, 133.90, 129.40 (2C), 128.58 (2C), 128.45 (2C), 127.96 (2C), 126.33, 56.48, 42.00, 35.30, 33.85, 33.23. IR (neat): 2920, 2855, 1446, 1306, 1147, 1086. HRMS (ESI): calcd. for

M+H+: $C_{17}H_{20}IO_2S$: 415.0223; found: 415.0215.

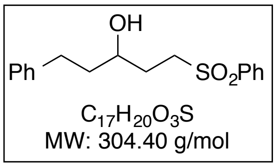

1-Phenyl-5-(phenylsulfonyl)pentane-3-ol (**11e**). Amorphous white solid. ^{1}H NMR (300 MHz, $CDCl_3$): δ = 7.91 - 7.89 (m, 2H), 7.68 - 7.64 (m, 1H), 7.59 - 7.55 (m, 2H), 7.30 - 7.15 (m, 5H), 3.73 (br, 1H), 3.34 - 3.17 (m, 2H), 2.79 - 2.62 (m, 2H), 2.02 - 1.94 (m, 1H), 1.87 - 1.73 (m, 4H). ^{13}C NMR (75 MHz, $CDCl_3$): δ = 141.31, 139.12, 133.72, 129.31 (2C), 128.51 (2C), 128.31 (2C), 127.97 (2C), 126.04, 69.31, 53.04, 39.03, 31.86, 30.09. IR (neat): 3515, 2926, 2861, 1602, 1496, 1446, 1304, 1146, 1086. HRMS (ESI): calcd. for M+H+: $C_{17}H_{21}O_3S$: 305.1206; found: 305.1206.

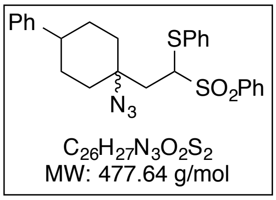

(2-(1-Azido-4-phenylcyclohexyl)-1-(phenylsulfonyl)ethyl)(phenyl)sulfane (**12**). The sulfone **9c** (369 mg, 1.00 mmol) was dissolved in THF (5.0 mL) and cooled to –78 °C. A freshly prepared solution of LiHMDS in THF (4.22 mL, 2.00 mmol) was added and stirred for 30 min at –78 °C. $(PhS)_2$ (436 mg, 2.00 mmol) was dissolved in THF (4.0 mL) and added to the reaction mixture, and stirred for 2 h at –78 °C. The reaction mixture was allowed to reach rt. H_2O (1.00 mL) and a saturated solution of NH_4Cl (15 mL) were added. The layers were separated. The aqueous phase was extracted with EtOAc (3 × 30 mL). The combined organic phases were washed with brine (20 mL) and dried over Na_2SO_4. The solvent was removed under reduced pressure. The crude product was purified by FC (DCM/Et_2O/*n*-pentane, 1:2:7) to afford the sulfide **12** as a mixture of diastereomers (310 mg, *cis*/*trans* 75:25, 65%). Yellowish oil: **12-major:** ^{1}H NMR (300 MHz, $CDCl_3$): δ = 7.91 (d, J = 7.5 Hz, 2H), 7.60 (t, J = 7.5 Hz, 1H), 7.48 (t, J = 7.5 Hz, 2H), 7.37 - 7.33 (m, 2H), 7.29 (d, J = 6.7 Hz, 3H), 7.29 - 7.19 (m, 5H), 4.43 (dd, J = 8.6, 1.3 Hz, 1H), 2.56 (dd, J = 15.3, 1.0 Hz, 1H), 2.53 - 2.45 (m, 1H), 2.06 - 1.98 (m, 2H), 1.92 (dd, 13.4, 2.7 Hz, 1H), 1.81 - 1,76 (m, 2H), 1.70 - 1.46 (m, 4H). ^{13}C NMR (75 MHz, $CDCl_3$): δ = 146.00, 136.60, 133.96, 132.99, 131,97 (2C), 129.68 (2C), 129.10 (2C), 128.94 (2C), 128.41 (2C), 128.22, 126.70 (2C), 126.25, 68.09, 62.52, 43.17, 39.93, 35.49, 34.85, 29.29, 29.25. Characteristic signals

for **12-minor**: ^{1}H NMR (300 MHz, $CDCl_3$): δ = 2.29 - 2.15 (m, 2H). ^{13}C NMR (75 MHz, $CDCl_3$): δ = 145.24, 136.67, 133.92, 133.28, 131.47 (2C), 129.68 (2C), 129.10 (2C), 128.94 (2C), 128.01 (2C), 126.34, 68.59, 62.57, 42.82, 34.33, 33.47, 30.04, 29.59. IR (neat): 3073, 3033, 2929, 2847, 2092, 1582, 1494, 1477, 1446, 1305, 1252, 1145, 1082, 1024. HRMS (ESI): calcd. for $M+Na^+$: $C_{26}H_{27}O_2N_3NaS_2$: 500.1437; found: 500.1437.

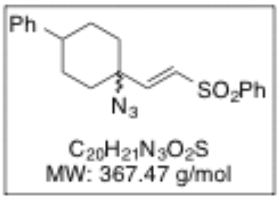

(E)-(4-Azido-4-(2-(phenylsulfonyl)vinyl)cyclohexyl)benzene (**13**). The sulfide **12** (239 mg, 0.50 mmol) was diluted in CH_2Cl_2 (6.0 mL) and cooled to –30 °C and *m*CPBA (108 mg, 0.48 mmol) was added. The reaction solution was stirred at –30 °C for 2 h. The stirring was continued at rt overnight. A saturated solution of Na_2CO_3 (20 mL) and CH_2Cl_2 (25 mL) were added. The layers were separated and the aqueous layer was extracted with CH_2Cl_2 (3 × 25 mL). The combined organic layers were washed with brine (80 mL) and dried over Na_2SO_4. The solvent was removed under reduced pressure. The crude product was dissolved in $CDCl_3$ (4.0 mL) and stirred at rt for 6 d. The solvent was removed under reduced pressure and the residue was purified by FC (*n*-pentane/Et_2O, 90:10) to afford **13** (149 mg, dr 9:1, 81%). Colourless liquid: **13-major:** ^{1}H NMR (300 MHz, $CDCl_3$): δ = 7.91 (dd, *J* = 7.3, 1.6 Hz, 2H), 7.66 (t, *J* = 7.3 Hz, 1H), 7.75 (t, *J* = 7.3 Hz, 2H), 7.34 - 7.29 (m, 2H), 7.23 - 7.19 (m, 3H), 6.96 (d, *J* = 15.2 Hz, 1H), 6.56 (d, *J* = 15.2 Hz, 1H), 2.57 - 2.47 (m, 1H), 1.93 (d, *J* = 1.6 Hz, 2H), 1.86 - 1.77 (m, 4H), 1.73 - 1.63 (m, 2H). ^{13}C NMR (75 MHz, $CDCl_3$): δ = 147.57, 145.71, 140.03, 133.69, 130.52, 129.44 (2C), 128.51 (2C), 127.74 (2C), 126.71 (2C), 126.44, 63.02, 42.85, 34.12 (2C), 28.76 (2C). Characteristic signals for **13-minor**: ^{1}H NMR (300 MHz, $CDCl_3$): δ = 6.64 (d, *J* = 15.2 Hz, 1H). IR (neat): 3058, 3030, 2030, 2857, 2096, 1446, 1325, 1307, 1252, 1148, 1086. HRMS (ESI): calcd. for $M–N_2$: $C_{20}H_{22}O_2NS$: 340.1366; found: 340.1363.

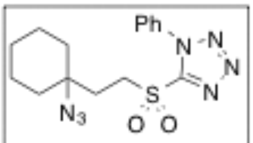

$C_{15}H_{19}N_7O_2S$
MW: 361.42 g/mol

5-((2-(1-Azidocyclohexyl)ethyl)sulfonyl)-1-phenyl-1H-tetrazole (**15a**). Tin-mediated method: According to the general method A with di-*tert*-butylhyponitrite[2] (17 mg, 0.1 mmol) was added in one portion to a solution of methylenecyclohexane **5a** (0.24 mL, 2.00 mmol), 5-((iodomethyl)sulfonyl)-1-phenyl-1*H*-tetrazole **14**[10] (350 mg, 1.00 mmol), hexabutylditin (0.61 mL, 1.20 mmol) and 3-$PySO_2N_3$[3] (552 mg, 3.00 mmol) in benzene (2.0 mL). The crude mixture was directly purified by FC using KF/silica (cyclohexane/EtOAc, 95:5) to afford **15a** (325 mg, 90%). Et_3B-mediated method: According to the general method B with 1 M solution of Et_3B in DCM (4.00 mL, 4.00 mmol), methylenecyclohexane **5a** (0.24 mL, 2.00 mmol), 5-((iodomethyl)sulfonyl)-1-phenyl-1*H*-tetrazole **14**[10]. (350 mg, 1.00 mmol), 3-$PySO_2N_3$[3] (552 mg, 3.00 mmol) and DCM (0.50 mL). Purification by FC (cyclohexane/EtOAc, 95:5) afforded **15a** (260 mg, 72%). Colourless crystals: M. p.: 90.9 - 93.6 °C. ^1H NMR (300 MHz, $CDCl_3$): δ = 7.76 - 7.72 (m, 2H), 7.69 - 7.62 (m, 3H), 3.90 - 3.84 (m, 2H), 2.24 - 2.18 (m, 2H), 1.81 - 1.73 (m, 2H), 1.69 - 1.29 (m, 8H). ^{13}C NMR (75 MHz, $CDCl_3$): δ = 153.31, 132.96, 131.50, 129.75 (2C), 125.00 (2C), 62.45, 51.61, 34.41 (2C), 31.72, 25.07, 21.96 (2C). IR (neat): 2933, 2856, 2098, 1497, 1337, 1253, 1150. HRMS (ESI): calcd. for $M+H^+$: $C_{15}H_{20}N_7O_2S$ calcd 362.1394; found: 362.1400.

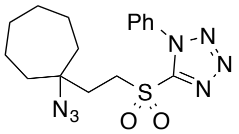

$C_{16}H_{21}N_7O_2S$
MW: 375.45 g/mol

5-((2-(1-Azidocycloheptyl)ethyl)sulfonyl)-1-phenyl-1H-tetrazole (**15b**). According to the general procedure B with a 1 M solution of Et_3B in DCM (4.00 mL, 4.00 mmol), methylenecyclopentane **5b** (220 mg, 2.00 mmol), 5-((iodomethyl)sulfonyl)-1-phenyl-1*H*-tetrazole **14**[10] (350 mg, 1.00 mmol), 3-$PySO_2N_3$[3] (552 mg, 3.0 mmol) and DCM (0.50 mL). Purification by FC (cyclohexane/EtOAc, 95:5) afforded **15b** (206 mg, 55%). Amorphous grey solid: ^1H NMR (300 MHz, $CDCl_3$): δ = 7.72 - 7.69 (m, 2H), 7.65 - 7.59 (m, 3H), 3.85 - 3.80 (m, 2H), 2.17 - 2.12 (m, 2H), 1.94 - 1.85 (m, 2H), 1.75 - 1.47 (m, 10H). ^{13}C NMR (75 MHz, $CDCl_3$): δ = 153.34, 132.98, 131.50, 129.74 (2C), 125.01 (2C), 65.93, 52.02, 38.07 (2C), 32.62, 29.29 (2C), 22.45 (2C). IR (neat): 2928, 2858, 2092, 1498, 1463, 1337, 1255, 1152, 1014. HRMS (ESI): calcd. for $M+H^+$: $C_{16}H_{22}O_2N_7S$: 376.1550; found: 376.1559.

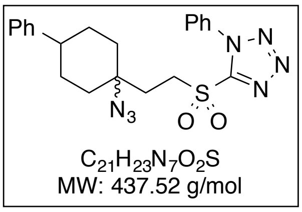

5-((2-(1-Azido-4-phenylcyclohexyl)ethyl)sulfonyl)-1-phenyl-1H-tetrazole (**15c**). According to the general procedure B with 1 M solution of Et_3B in DCM (4.00 mL, 4.00 mmol), (4-methylenecyclohexyl)benzene **5c**[8] (344 mg, 2.00 mmol), 5-((iodomethyl)sulfonyl)-1-phenyl-1*H*-tetrazole **14**[10] (350 mg, 1.00 mmol), 3-$PySO_2N_3$[4] (552 mg, 3.00 mmol) and DCM (0.50 mL). Purification by FC (cyclohexane/EtOAc, 90:10) afforded **15c** as an inseparable mixture of diastereomers (284 mg, dr 7.5:2.5, 65%). Amorphous solid. **15c-major:** ^{1}H NMR (300 MHz, $CDCl_3$): δ = 7.73 - 7.70 (m, 2H), 7.65 - 7.61 (m, 3H), 7.35 - 7.19 (m, 5H), 3.94 - 3.89 (m, 2H), 2.58 - 2.48 (m, 1H), 2.27 - 2.22 (m, 2H), 1.99 - 1.92 (m, 2H), 1.87 - 1.69 (m, 4H), 1.82 - 1.52 (m, 2H). ^{13}C NMR (75 MHz, $CDCl_3$): δ = 153.30, 145.86, 132.97, 131.55, 129.80 (2C), 128.50 (2C), 126.74 (2C), 126.38, 124.99 (2C), 61.86, 51.65, 43.28, 34.75 (2C), 33.69, 29.26 (2C). Characteristic signals for **15c-minor**: ^{1}H NMR (300 MHz, $CDCl_3$): δ = 3.86 - 3.81 (m, 2H), 2.36 - 2.30 (m, 2H). ^{13}C NMR (75 MHz, $CDCl_3$): δ = 128.59, 61.12, 51.77, 42.99, 34.28, 29.82. IR (neat): 3083, 3061, 3027, 2928, 2862, 2094, 1596, 1496, 1451, 1338, 1309, 1254, 1174, 1154, 1014. HRMS (ESI): calcd. for M+H$^+$: $C_{21}H_{24}O_2N_7S$: 438.1707; found: 438.1712.

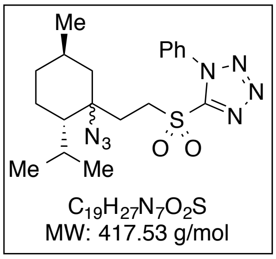

5-((2-((2R,5S)-1-Azido-2-isopropyl-5-methylcyclohexyl)ethyl)sulfonyl)-1-phenyl-1H-tetrazole (**15d**). According to the general procedure B with 1 M solution of Et_3B in DCM (4.00 mL, 4.00 mmol), (1*S*,4*R*)-1-Isopropyl-4-methyl-2-methylenecyclohexane **5d** (304 mg, 2.00 mmol), 5-((iodomethyl)sulfonyl)-1-phenyl-1*H*-tetrazole **14**[10] (350 mg, 1.00 mmol), 3-$PySO_2N_3$[4] (552 mg, 3.00 mmol) and DCM (0.50 mL). Purification by FC (cyclohexane/EtOAc, 98:2) afforded **15d** as an inseparable mixture of diastereomers (192 mg, dr 7:3, 46%). Colourless oil. ^{1}H NMR (300 MHz, $CDCl_3$): δ = 7.23 - 7.68 (m, 2H for the 2 diast.), 7.64 - 7.57 (m, 3H for the 2 diast.), 3.83 - 3.62 (m, 2H for the 2 diast.), 2.37 - 2.31 (m, 1H for the major), 2.28 - 2.16 (m, 2H for the minor), 2.09 - 1.97 (m, 2H for the 2 diast.), 1.87 - 1.72 (m, 3H for major, 2H for the minor), 1.64 - 1.39 (m, 3H for the 2 diast.), 1.28

- 1.09 (m, 2H for the 2 diast.), 1.02 (d, *J* = 6.9 Hz, 3H for the major), 0.97 (d, *J* = 6.9 Hz, 3H for the major), 0.95 (d, *J* = 6.1 Hz, 3H for the minor), 0.92 - 0.86 (m, 6H for the minor), 0.83 (d, *J* = 6.9 Hz, 3H for the major). **15d-major**: ^{13}C NMR (75 MHz, $CDCl_3$): δ = 153.46, 133.00, 131.49, 129.73 (2C), 125.06 (2C), 67.15, 51.94, 49.70, 42.34, 34.42, 29.98, 26.25, 23.96, 23.90, 23.49, 22.07, 19.57. **15d-minor**: ^{13}C NMR (75 MHz, $CDCl_3$): δ = 153.42, 132.94, 131.56, 129.79 (2C), 125.00 (2C), 65.55, 52.05, 48.61, 43.29, 34.68, 30.82, 28.33, 25.73, 23.84, 22.13, 21.34, 17.43. IR (neat): 2954, 2926, 2869, 2097, 1497, 1458, 1340, 1257, 1152. HRMS (ESI): calcd. for $M+H^+$: $C_{19}H_{28}O_2N_7S$: 418.2020; found: 418.2012.

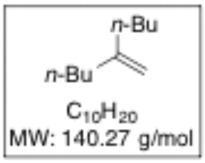

5-Methylenenonane (**5f**). According to the general procedure C with nonane-5-one (2.60 mL, 15.00 mmol), $MePPh_3Br$ (6.43 g, 18.00 mmol), *t*-BuONa (1.73 g, 18.00 mmol) and Et_2O (100 mL). Purification by FC with solid loading (*n*-pentane, 100%) afforded **5f** (1.45 g, 69%). The spectral data were in accordance with the literature.[11] Colourless liquid: ^{1}H NMR (300 MHz, $CDCl_3$): δ = 4.69 - 4.68 (m, 2H), 2.00 (t, *J* = 7.4 Hz, 4H), 1.46 - 1.25 (m, 8H), 0.91 (t, *J* = 7.1 Hz, 6H). ^{13}C NMR (75 MHz, $CDCl_3$): δ = 150.40, 108.31, 35.79 (2C), 30.05 (2C), 22.50 (2C), 13.98 (2C).

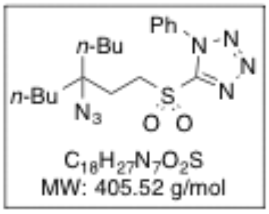

5-((3-Azido-3-butylheptyl)sulfonyl)-1-phenyl-1H-tetrazole (**15f**). Tin-mediated method: According to the general method A with di-*tert*-butylhyponitrite[2] (17 mg, 0.1 mmol) 5-methylenenonane **5f** (0.280 mg, 2.00 mmol), 5-((iodomethyl)sulfonyl)-1-phenyl-1*H*-tetrazole **14**[10] (350 mg, 1.00 mmol), hexabutylditin (0.61 mL, 1.20 mmol) and 3-$PySO_2N_3$[3] (552 mg, 3.00 mmol) in benzene (2.0 mL). The crude mixture was directly purified by FC using KF/silica (*n*-pentane/Et_2O, 95:5) to afford **15f** (126 mg, 31%) and **16f** (61 mg, 20%). Et_3B-mediated method: According to the general procedure B with 1 M solution of Et_3B in DCM (4.00 mL, 4.00 mmol), 5-((iodomethyl)sulfonyl)-1-phenyl-1*H*-tetrazole **14**[10] (350 mg, 1.00 mmol), 5-methylenenonane **5f** (280 mg, 2.00 mmol) and 3-$PySO_2N_3$[4] (552 mg, 3.00 mmol) in DCM (0.50 mL). The crude product was

purified by FC (*n*-pentane/Et_2O, 95:5) to afford the azide **15f** (195 mg, 48%). Colourless oil: ^1H NMR (300 MHz, $CDCl_3$): δ = 7.71 - 7.67 (m, 2H), 7.64 - 7.57 (m, 3H), 3.79 - 3.74 (m, 2H), 2.12 - 2.07 (m, 2H), 1.68 - 1.51 (m, 4H), 1.41 - 1.26 (m, 8H), 0.94 (t, *J* = 6.9 Hz, 6H). ^{13}C NMR (75 MHz, $CDCl_3$): δ = 153.28, 132.94, 131.51, 129.75 (2C), 124.99 (2C), 65.02, 51.89, 35.98 (2C), 29.31, 25.56 (2C), 22.91 (2C), 13.92 (2C). IR (neat): 2957, 2934, 2871, 2097, 1497, 1457, 1338, 1254, 1152. HRMS (ESI): calcd. for $M+H^+$: $C_{18}H_{28}O_2N_7S$: 406.2020; found: 406.2020.

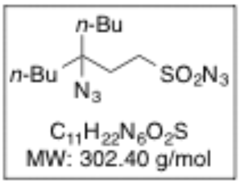

3-Azido-3-butylheptane-1-sulfonyl azide (**16f**). Colourless crystals: ^1H NMR (300 MHz, $CDCl_3$): δ = 3.37 - 3.31 (m, 2H), 2.04 - 1.98 (m, 2H), 1.66 - 1.48 (m, 4H), 1.39 - 1.25 (m, 8H), 0.94 (t, *J* = 6.4 Hz, 6H). ^{13}C NMR (75 MHz, $CDCl_3$): δ = 64.90, 51.39, 36.03 (2C), 30.40, 25.64 (2C), 22.91 (2C), 13.90 (2C). IR (neat): 2957, 2935, 2872, 2133, 2097, 1457, 1369, 1255, 1196, 1159. HRMS (ESI): calcd. for $M–H^+$: $C_{11}H_{21}O_2N_6S$: 301.1452; found: 301.1455.

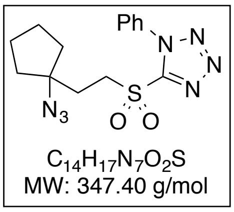

5-((2-(1-Azidocyclopentyl)ethyl)sulfonyl)-1-phenyl-1H-tetrazole (**15g**). According to the general procedure B with 1 M solution of Et_3B in DCM (4.00 mL, 4.00 mmol), methylenecyclopentane **5g** (0.21 mL, 2.00 mmol), 5-((iodomethyl)sulfonyl)-1-phenyl-1*H*-tetrazole **14**[10] (350 mg, 1.00 mmol), 3-$PySO_2N_3$[3] (552 mg, 3.00 mmol) and DCM (0.50 mL). Purification by FC (cyclohexane/EtOAc, 90:10) afforded **15g** (172 mg, 50%). Colourless crystals: M. p. = 84.4 - 85.3 °C. ^1H NMR (300 MHz, $CDCl_3$): δ = 7.71 - 7.67 (m, 2H), 7.64 - 7.58 (m, 3H), 3.89 - 3.84 (m, 2H), 2.27 - 2.21 (m, 2H), 1.97 - 1.89 (m, 2H), 1.87 - 1.63 (m, 6H). ^{13}C NMR (75 MHz, $CDCl_3$): δ = 153.33, 132.98, 131.52, 129.77 (2C), 125.01 (2C), 71.38, 52.94, 37.02 (2C), 31.88, 23.86 (2C). IR (neat): 2960, 2871, 2098, 1497, 1455, 1337, 1250, 1151, 1101, 1075, 1045, 1014. HRMS (ESI): calcd. for $M+H^+$: $C_{14}H_{18}O_2N_7S$: 348.1237; found: 348.1243.

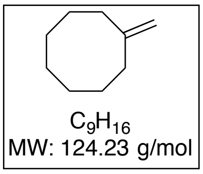

Methylenecyclooctane.(**5h**) According to the general procedure C with cyclooctanone (6.31 g, 50.00 mmol), $MePPh_3Br$ (21.40 g, 60.00 mmol), *t*-BuONa (5.77 g, 60.00 mmol) and Et_2O (320 mL). Purification by FC with solid loading (*n*-pentane, 100%). Colourless liquid (4.65g, 75%, yield corrected from solvent residue). The spectral data were in accordance with the literature.[12] ^1H NMR (300 MHz, $CDCl_3$): δ = 4.76 (s, 2H), 2.22 - 2.18 (m, 4H), 1.69 - 1.61 (m, 4H), 1.54 - 1.49 (m, 6H). ^{13}C NMR (75 MHz, $CDCl_3$): δ = 152.37, 110.81, 35.29 (2C), 27.57 (2C), 26.81 (2C), 25.97.

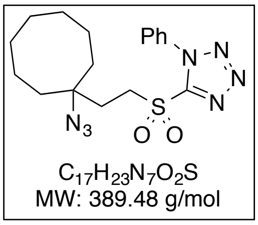

5-((2-(1-Azidocyclooctyl)ethyl)sulfonyl)-1-phenyl-1H-tetrazole (**15h**). According to the general procedure B with 1 M solution of Et_3B in DCM (4.00 mL, 4.00 mmol), methylenecyclooctane **5h** (248 mg, 2.00 mmol), 5-((iodomethyl)sulfonyl)-1-phenyl-1*H*-tetrazole **14**[10] (350 mg, 1.00 mmol), 3-$PySO_2N_3$[3] (552 mg, 3.00 mmol) and DCM (0.50 mL). Purification by FC (cyclohexane/EtOAc, 90:10) afforded **15h** (245 mg, 63%). Colourless oil: ^1H NMR (300 MHz, $CDCl_3$): δ = 7.71 - 7.67 (m, 2H), 7.64 - 7.59 (m, 3H), 3.84 - 3.78 (m, 2H), 2.16 - 2.10 (m, 2H), 2.03 - 1.94 (m, 2H), 1.70 - 1.59 (m, 8H), 1.50 - 1.44 (m, 4H). ^{13}C NMR (75 MHz, $CDCl_3$): δ = 153.32, 132.96, 131.47, 129.72 (2C), 125.00 (2C), 65.90, 51.98, 33.25 (2C), 31.18, 27.90 (2C), 24.80, 22.23 (2C). IR (neat): 2922, 2854, 2102, 1595, 1497, 1478, 1447, 1338, 1250, 1152, 1072, 1049, 1014. HRMS (ESI): calcd. for $M+H^{+}$: $C_{17}H_{24}O_2N_7S$: 390.1707; found: 390.1705.

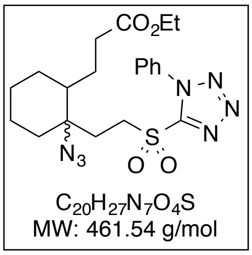

Ethyl 3-(2-azido-2-(2-((1-phenyl-1H-tetrazol-5-yl)sulfonyl)ethyl)cyclohexyl)propanoate (**15i**). According to the general procedure B with 1 M solution of Et_3B in DCM (4.00 mL, 4.00 mmol), ethyl 3-(2-methylenecyclohexyl)propanoate **5i**[13] (393 mg, 2.00 mmol), 5-((iodomethyl)sulfonyl)-1-phenyl-1*H*-tetrazole **14**[10] (350 mg, 1.00 mmol), 3-$PySO_2N_3$[3] (552 mg, 3.00 mmol) and DCM (0.50 mL). Purification by FC (cyclohexane/EtOAc, 85:15) afforded **15i** as an inseparable mixture of diastereomers (300 mg, dr 6:4, 65%). Yellowish oil. **15i-major:** ^1H NMR (300 MHz,

$CDCl_3$): δ = 7.66 - 7.63 (m, 2H), 7.57 - 7.51 (m, 3H), 4.11 - 4.01 (m, 2H), 3.76 - 3.65 (m, 1H), 2.43 - 2.09 (m, 4H), 1.99 - 1.76 (m, 4H), 1.68 - 1.43 (m, 5H), 1.39 - 1.24 (m, 3H), 1.22 - 1.16 (m, 3H). ^{13}C NMR (75 MHz, $CDCl_3$): δ = 172.83, 153.16, 132.79, 131.25, 129.49 (2C), 124.90 (2C), 65.55, 60.28, 51.35, 41.72, 32.27, 31.95, 30.96, 26.01, 24.09, 23.54, 21.94, 13.99. Characteristic signals for **15i-minor**: ^{13}C NMR (75 MHz, $CDCl_3$): δ = 173.11, 153.07, 132.81, 129.47 (2C), 24.94 (2C), 65.09, 60.20, 51.47, 42.92, 32.93, 30.05, 26.66, 24.19, 23.95, 23.64, 21.49. IR (neat): 2937, 2862, 2096, 1729, 1595, 1497, 1458, 1339, 1259, 1179, 1152, 1036, 1014. HRMS (ESI): calcd. for M+Na$^+$: $C_{20}H_{27}O_4N_7NaS$: 484.1737; found: 484.1738.

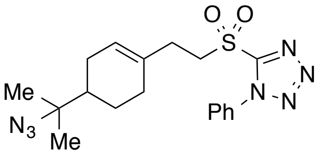

$C_{18}H_{23}N_7O_2S$
401.49 g/mol

5-((2-(4-(2-Azidopropan-2-yl)cyclohex-1-en-1-yl)ethyl)sulfonyl)-1-phenyl-1H-tetrazole (**15j**). According to the general procedure B with 1 M solution of Et_3B in DCM (4.00 mL, 4.00 mmol), (–)-β-pinene **5j** (0.33 mL, 2.00 mmol), 5-((iodomethyl)sulfonyl)-1-phenyl-1*H*-tetrazole **14**[10] (350 mg, 1.00 mmol), 3-$PySO_2N_3$[3] (552 mg, 3.00 mmol) and DCM (0.50 mL). Purification by FC (cyclohexane/EtOAc, 85:15) afforded **15j** (112 mg, 28%). Colourless liquid: ^{1}H NMR (300 MHz, $CDCl_3$): δ = 7.70 - 7.66 (m, 2H), 7.64 - 7.58 (m, 3H), 5.53 (d, J = 3.3 Hz, 1H), 3.82 (td, J = 7.2, 3.3 Hz, 2H), 2.61 - 2.56 (m, 2H), 2.11 - 2.04 (m, 3H), 1.93 - 1.76 (m, 2H), 1.57 - 1.47 (m, 1H), 1.34 – 1.22 (m 1H), 1.27 (s, 3H), 1.24 (s, 3H). ^{13}C NMR (75 MHz, $CDCl_3$): δ = 153.47, 133.04, 132.62, 131.46, 129.71 (2C), 125.05 (2C), 123.68, 63.84, 54.64, 43.16, 29.41, 28.83, 26.70, 23.82, 23.73, 23.13. IR (neat): 2972, 2922, 2840, 2099, 1497, 1343, 1266, 1248, 1151. HRMS (ESI): calcd. for M+H$^+$: $C_{18}H_{24}O_2N_7S$: 402.1707; found: 402.1701.

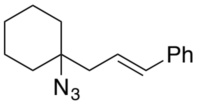

$C_{15}H_{19}N_3$
MW: 241.34 g/mol

(3-(1-Azidocyclohexyl)prop-1-en-1-yl)benzene (**17a**). According to the general procedure D with the tetrazole **15a** (260 mg, 0.72 mmol), LiHMDS in THF (1.66 mL, 1.08 mmol), benzaldehyde (0.15 mL, 1.44 mmol) and THF (3.00 mL). Purification by FC (cyclohexane/EtOAc, 98:2) afforded the alkene **17a** as an inseparable mixture of isomers (141 mg, *E*/*Z* > 95:5, 81%). Colourless oil. **17a-major:** ^{1}H NMR (300 MHz, $CDCl_3$): δ = 7.39 - 7.20 (m,

5H), 6.47 (d, J = 15.8 Hz, 1H), 6.24 (dt, J = 15.8, 7.4 Hz, 1H), 2.46 (dd, J = 7.4, 1.2 Hz, 2H), 1.72 (d, J = 13.1 Hz, 2H), 1.65 - 1.39 (m, 7H), 1.32 - 1.21 (m, 1H). ^{13}C NMR (75 MHz, $CDCl_3$): δ = 137.24, 133.74, 128.51 (2C), 127.29, 126.17 (2C), 124.36, 64.22, 43.93, 34.52 (2C), 25.33, 22.07 (2C). Characteristic signals for **17a-minor**: ^{1}H NMR (300 MHz, $CDCl_3$): δ = 2.55 (d, J = 5.8 Hz, 2H). IR (neat): 3027, 2931, 2858, 2096, 1495, 1447, 1254, 1138, 1102, 1029. EI-MS m/z (%): M–N_2: 213.3 (21), 198.3 (7), 170.3 (20), 156.3 (16), 128.3 (10), 117.3 (100), 115.3 (73), 96.3 (63), 91.3 (40), 69.3 (34), 55.3 (39). HRMS (ESI): calcd. for M+H$^+$: $C_{15}H_{20}N_3$: 242.1652; found: 242.1655.

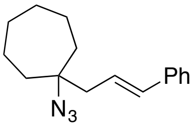

$C_{16}H_{21}N_3$
MW: 255.37 g/mol

1-Azido-1-cinnamylcycloheptane (**17b**). According to the general procedure D with the tetrazole **15b** (180 mg, 0.48 mmol), LiHMDS in THF (1.44 mL, 0.72 mmol), benzaldehyde (0.10 mL, 0.96 mmol) and THF (3.00 mL). Purification by FC (cyclohexane/EtOAc, 99:1) afforded the alkene **17b** (107 mg, *E*/*Z* > 95:5, 88%). Colourless liquid. **17b-major:** ^{1}H NMR (300 MHz, $CDCl_3$): δ = 7.39 - 7.29 (m, 4H), 7.25 - 7.19 (m, 1H), 6.47 (d, J = 15.8 Hz, 1H), 6.24 (dt, J = 15.8, 7.3 Hz, 1H), 2.46 (dd, J = 7.3 Hz, 1.2 Hz, 2H), 1.85 - 1.77 (m, 2H), 1.73 - 1.46 (m, 10H). ^{13}C NMR (75 MHz, $CDCl_3$): δ = 137.27, 133.70, 128.52 (2C), 127.29, 126.17 (2C), 124.81, 67.73, 44.74, 38.17 (2C), 29.34 (2C), 22.48 (2C). Characteristic signals for **17b-minor**: ^{1}H NMR (300 MHz, $CDCl_3$): δ = 2.41 - 2.38 (m, 2H). ^{13}C NMR (75 MHz, $CDCl_3$): δ = 128.42 (2C), 126.00 (2C), 37.92 (2C), 29.71 (2C), 22.16 (2C). IR (neat): 3085, 1057, 3030, 2925, 2855, 2088, 1598, 1496, 1459, 1447, 1255. MS m/z (%): M–N_2: 227.3 (45), 184.2 (18), 117.2 (36), 115.2 (43), 110.2 (100), 91.2 (37), 69.2 (22). HRMS (ESI): calcd. for M+H$^+$: $C_{16}H_{22}N_3$: 256.1808; found: 256.1811.

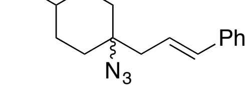

$C_{21}H_{23}N_3$
MW: 317.44 g/mol

(E)-(4-Azido-4-cinnamylcyclohexyl)benzene (**17c**). According to the general procedure D with the tetrazole **15c** (779 mg, 1.78 mmol), LiHMDS in THF (4.96 mL, 3.23 mmol), benzaldehyde (0.44 mL, 4.31 mmol) and THF (9.00 mL). Purification by FC (*n*-pentane/Et_2O, 95:5) afforded the alkene **17c** as an inseparable mixture of

diastereomers (471 mg, dr 75:25, only *E*, 83%). Colourless liquid. **17c-major:** ^{1}H NMR (300 MHz, $CDCl_3$): δ = 7.43 - 7.17 (m, 10H), 6.51 (d, *J* = 15.7 Hz, 1H), 6.33 - 6.23 (m, 1H), 2.53 (dd, *J* = 7.5 Hz, 0.9 Hz, 2H), 2.52 - 2.40 (m, 1H), 1.94 (dd, *J* = 14.1, 2.7 Hz, 2H), 1.82 - 1.75 (m, 4H), 1.61 - 1.51 (m, 2H). ^{13}C NMR (75 MHz, $CDCl_3$): δ = 146.52, 137.15, 134.11, 128.56 (2C), 128.40 (2C), 127.41, 126.80 (2C), 126.21 (2C), 126.16, 124.07, 63.60, 45.09, 43.54, 34.77 (2C), 29.51 (2C). Characteristic signals for **17c-minor**: ^{1}H NMR (300 MHz, $CDCl_3$): δ = 2.60 (dd, *J* = 7.3, 1.0 Hz, 2H). ^{13}C NMR (75 MHz, $CDCl_3$): δ = 145.76, 137.20, 133.61, 128.58 (2C), 127.35, 126.73 (2C), 126.04, 124.42, 42.86, 38.38, 34.40 (2C), 29.92 (2C). IR (neat): 3024, 2930, 2856, 2091, 1495, 1448, 1259. EI-MS m/z (%): M–N_2: 289.3 (100), 274.3 (14), 260.3 (7), 233.3 (7), 212.3 (13), 198.3 (25), 184.3 (36), 170.3 (61), 131.3 (31), 117.3 (78), 94.3 (70), 91.3 (63), 77.3 (9). HRMS (ESI): calcd. for M+H$^+$: $C_{21}H_{24}N_3$: 318.1965; found: 318.1966.

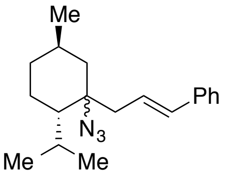

$C_{19}H_{27}N_3$
MW: 297.45 g/mol

((E)-3-((2R,5S)-1-Azido-2-isopropyl-5-methylcyclohexyl)prop-1-en-1-yl)benzene **(17d)**. According to the general procedure D with tetrazole **15d** (133 mg, 0.32 mmol), LiHMDS in THF (1.29 mL, 0.48 mmol), benzaldehyde (0.06 mL, 0.64 mmol) and THF (3.00 mL). Purification by FC (cyclohexane/EtOAc, 99:1) afforded the alkene **17d** (50 mg, dr 7:3, only *E*, 53%). Colourless liquid. ^{1}H NMR (300 MHz, $CDCl_3$): δ = 7.29 - 7.19 (m, 4H for 2 diast.), 7.16 - 7.09 (m, 1H for 2 diast.), 6.39 (d, *J* = 16.0 Hz, 1H for minor), 6.34 (d, *J* = 16.4 Hz, 1H for major), 6.15 - 5.98 (m, 1H for 2 diast.), 2.68 - 2.41 (m, 1H for major), 2.36 - 2.23 (m, 1H for major, 2H for minor), 2.08 - 1.98 (m, 1H for 2 diast.), 1.91 - 1.85 (m, 1H for major), 1.77 - 1.57 (m, 2H for major, 3H for minor), 1.53 - 1.39 (m, 1H for 2 diast.), 1.38 - 1.32 (m, 1H for 2 diast.), 1.30 - 1.01 (m, 2H for 2 diast.), 0.97 - 0.92 (m, 3H for 2 diast.), 0.83 - 0.76 (m, 7H for 2 diast.). **17d-major:** ^{13}C NMR (75 MHz, $CDCl_3$): δ = 137.47, 133.12, 128.51 (2C), 127.17, 126.12 (2C), 125.24, 68.76, 50.03, 42.62, 34.96, 34.65, 28.80, 26.13, 24.25, 23.74, 22.20, 19.34. **17d-minor:** ^{13}C NMR (75 MHz, $CDCl_3$): δ = 137.26, 134.12, 128.58 (2C), 127.40, 126.15 (2C), 124.34, 67.20, 47.68, 43.60, 41.98, 34.88, 28.27, 25.64, 23.71, 22.20, 21.39, 17.48. IR (neat): 3026, 2954, 2926, 2868, 2849, 2092, 1457, 1389, 1368, 1256, 1164. MS m/z (%): M–N_2: 269.3 (11), 254.3 (19), 227.3 (20), 228.3 (36), 211.3 (23),

178.3 (7), 155.2 (11), 152.3 (22), 136.3 (24), 117.2 (100), 115.2 (53), 107.2 (15), 91.2 (31), 69.2 (9), 55.3 (12). HRMS (ESI): calcd. for M+H+: $C_{19}H_{28}N_3$: 298.2278; found: 298.2283.

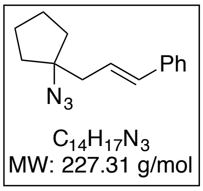

(E)-(3-(1-Azidocyclopentyl)prop-1-en-1-yl)benzene (**17g**). According to the general procedure D with the tetrazole **15g** (166 mg, 0.48 mmol), LiHMDS in THF (1.43 mL, 0.72 mmol), benzaldehyde (0.10 mL, 0.96 mmol) and THF (3.00 mL). Purification by FC (cyclohexane/EtOAc, 99:1) afforded the alkene **17g** (80 mg, *E*/*Z* > 95:5 , 74%). Colourless liquid. **17g-major:** ^{1}H NMR (330 MHz, $CDCl_3$): δ = 7.40 - 7.21 (m, 5H), 6.50 (d, *J* = 15.8 Hz, 1H), 6.25 (dt, *J* = 15.8, 7.3 Hz, 1H), 2.65 (dd, *J* = 7.3, 1.2 Hz, 2H), 1.88 - 1.64 (m, 8H). ^{13}C NMR (75 MHz, $CDCl_3$): δ = 137.26, 133.41, 128.51 (2C), 127.30, 126.18 (2C), 125.26, 73.09, 42.47, 36.55 (2C), 23.79 (2C). Characteristic signals for **17g-minor**: ^{1}H NMR (300 MHz, $CDCl_3$): δ = 2.51 (dd, *J* = 7.3, 1.2 Hz, 2H). ^{13}C NMR (75 MHz, $CDCl_3$): δ = 131.91, 128.42 (2C), 127.04, 126.88, 126.03 (2C), 67.95, 40.05, 36.05 (2C), 25.60 (2C). IR (neat): 3057, 3027, 2959, 2871, 2092, 1497, 1448, 1255, 1073. MS m/z (%): M–N_2: 199.2 (19), 118.2 (18), 117.2 (100), 115.2 (72), 91.2 (43), 65.2 (17), 55.2 (79). HRMS (ESI): calcd. for M+H+: $C_{14}H_{18}N_3$: 228.1495; found: 228.1502.

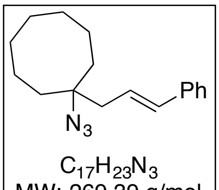

1-Azido-1-cinnamylcyclooctane (**17h**). According to the general procedure D with the tetrazole **15h** (161 mg, 0.41 mmol), LiHMDS in THF (1.38 mL, 0.62 mmol), benzaldehyde (0.09 mL, 0.83 mmol) and THF (3.00 mL). Purification by FC (cyclohexane/EtOAc, 99:1) afforded the alkene **17h** (73 mg, *E*/*Z* > 95:5, 66%). Colourless liquid. **17h-major:** ^{1}H NMR (300 MHz, $CDCl_3$): δ = 7.39 - 7.29 (m, 4H), 7.25 - 7.19 (m, 1H), 6.47 (d, *J* = 15.8 Hz, 1H), 6.25 (dt, *J* = 15.8, 7.3 Hz, 1H), 2.45 (dd, *J* = 7.3, 1.1 Hz, 2H), 1.90 - 1.82 (m, 2H), 1.72 - 1.47 (m, 12H). ^{13}C NMR (75 MHz, $CDCl_3$): δ = 137.30, 133.56, 128.52 (2C), 127.28, 126.18 (2C), 124.87, 67.77, 43.29, 33.39 (2C), 28.13 (2C), 24.77, 22.27 (2C). Characteristic signals for **17h-minor**: ^{1}H NMR (300 MHz, $CDCl_3$): δ = 5.96 (t, *J* = 7.4

Hz, 1H), 2.40 (d, J = 7.4 Hz, 2H). ^{13}C NMR (75 MHz, $CDCl_3$): δ = 124.98, 67.95, 39.66 (2C), 33.30 (2C), 28.03 (2C). IR (neat): 3026, 2924, 2854, 2092, 1496, 1472, 1447, 1254, 1119, 1072. MS m/z (%): M–N_2: 241.3 (34), 212.2 (9), 198.2 (32), 170.2 (8), 150.2 (21), 124.2 (100), 115.2 (41), 91.2 (36), 82.2 (19). HRMS (ESI): calcd. for M+H$^+$: $C_{17}H_{24}N_3$: 270.1965; found: 270.1966.

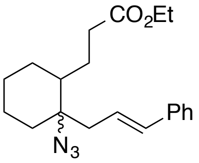

$C_{20}H_{27}N_3O_2$
MW: 341.46 g/mol

(E)-Ethyl 3-(2-azido-2-cinnamylcyclohexyl)propanoate (**17i**). According to the general procedure C with the tetrazole **15i** (260 mg, 0.56 mmol), LiHMDS in THF (1.52 mL, 0.84 mmol), benzaldehyde (0.11 mL, 1.12 mmol) and THF (3.00 mL). Purification by FC (cyclohexane/EtOAc, 95:5) afforded the alkene **17i** as an inseparable mixture of diastereomers (126 mg, dr 6:4, *E/Z* >95:5, 66%). Colourless liquid. **17i-mix:** ^{1}H NMR (300 MHz, $CDCl_3$): δ = 7.38 (dd, J = 7.4, 1.5 Hz, 2H), 7.31 (td, J = 7.4, 2.4 Hz, 2H), 7.25 - 7.20 (m, 1H), 6.50 (dd, J = 15.8, 7.6, 1H), 6.28 - 6.16 (m, 1H), 4.19 - 4.10 (m, 1H), 2.65 (ddd, J = 21.3, 14.2, 7.6, 1H), 2.50 - 2.17 (m, 3H), 2.10 - 2.00 (m, 1H), 1.96 - 1.86 (m, 1H), 1.84 - 1.77 (m, 1H), 1.74 - 1.33 (m, 8H), 1.30 - 1.17 (m, 4H). **17i-major**: ^{13}C NMR (75 MHz, $CDCl_3$): δ = 173.41, 137.28, 133.50, 128.47 (2C), 127.21, 126.11 (2C), 124.46, 67.13, 60.37, 42.24, 41.60, 32.63, 31.88, 26.59, 24.56, 24.47, 22.11, 14.18. **17i-minor**: ^{13}C NMR (75 MHz, $CDCl_3$): δ = 173.59, 137.16, 134.22, 128.51 (2C), 127.35, 126.15 (2C), 123.99, 66.63, 60.26, 43.57, 35.55, 33.79, 32.66, 27.07, 25.10, 23.91, 21.76, 14.20. IR (neat): 2979, 2931, 2859, 2091, 1732, 1599, 1496, 1447, 1369, 1256, 1174, 1115, 1032. MS m/z (%): M–N_2: 313.2 (18), 212.2 (31), 156.2 (13), 150.2 (15), 122.2 (32), 117.2 (100), 115.2 (47), 91.2 (29), 81.2 (7). HRMS (ESI): calcd. for M+H$^+$: $C_{20}H_{28}O_2N_3$: 342.2176; found: 342.2177.

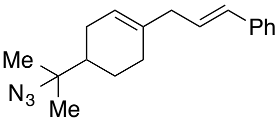

$C_{18}H_{23}N_3$
MW: 281.40 g/mol

(3-(4-(2-Azidopropan-2-yl)cyclohex-1-en-1-yl)prop-1-en-1-yl)benzene (**17j**). According to the general procedure D with the tetrazole **15j** (112 mg, 0.28 mmol), LiHMDS in THF (1.26 mL, 0.42 mmol), benzaldehyde (0.06 mL, 0.56 mmol) and THF (3.00 mL). Purification by FC (cyclohexane/EtOAc, 99:1) to afford the alkene **17j** (50

mg, *E*/*Z* 85:15, 64%). Colourless liquid. **17j-major**: ^{1}H NMR (300 MHz, $CDCl_3$): δ = 7.26 - 7.07 (m, 5H), 6.29 (d, *J* = 15.8 Hz, 1H), 6.14 - 6.04 (m, 1H), 5.38 (s, 1H), 2.73 (d, *J* = 6.7 Hz, 2H), 2.02 - 1.95 (m, 4H), 1.80 - 1.72 (m, 2H), 1.54 - 1.42 (m, 1H), 1.20 - 1.11 (m, 6H). ^{13}C NMR (75 MHz, $CDCl_3$): δ = 137.64, 136.49, 131.09, 128.46 (2C), 128.31, 126.95, 126.02 (2C), 121.17, 64.09, 43.56, 40.90, 29.27, 26.83, 24.15, 23.79, 23.06. Characteristic signals for **17j-minor**: ^{1}H NMR (300 MHz, $CDCl_3$): δ = 6.41 (d, *J* = 12.6 Hz, 1H), 5.76 (t, *J* = 7.6 Hz, 1H), 3.04 (d, *J* = 18.4 Hz, 1H), 2.85 - 2.80 (m, 1H). IR (neat): 3030, 2972, 2922, 2890, 2841, 2095, 1495, 1448, 1388, 1369, 1262, 1135. MS m/z (%): M–N_2: 253.3 (42), 238.3 (50), 182.3 (17), 167.2 (21), 136.3 (100), 115.2 (80), 91.2 (85), 71.2 (41), 56.2 (90). HRMS (ESI): M–N_2: calcd. for $C_{18}H_{24}N$: 254.1903; found: 254.1895.

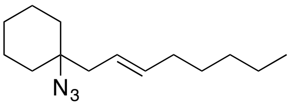

$C_{14}H_{25}N_3$
MW: 235.38 g/mol

(E)-1-Azido-1-(oct-2-en-1-yl)cyclohexane (**18a**). According to the general procedure D with the tetrazole **15a** (250 mg, 0.69 mmol), LiHMDS in THF (1.64 mL, 1.04 mmol), hexanal (0.17 mL, 1.38 mmol) and THF (3.00 mL). Purification by FC (*n*-pentane/Et_2O, 99:1) to afford the alkene **18a** (131 mg, *E*/*Z* 84:16, 81%). Colourless liquid. **18a-major**: ^{1}H NMR (300 MHz, $CDCl_3$): δ = 5.57 - 5.38 (m, 2H), 2.23 (d, *J* = 6.7 Hz, 2H), 2.02 (dd, *J* = 13.6, 6.7 Hz, 2H), 1.67 - 1.49 (m, 7H), 1.40 - 1.16 (m, 9H), 0.88 (t, *J* = 6.7 Hz, 3H). ^{13}C NMR (75 MHz, $CDCl_3$): δ = 135.19, 123.71, 63.99, 43.64, 34.40 (2C), 32.62, 31.38, 29.00, 25.42, 22.49, 22.09 (2C), 14.04. Characteristic signals for **18a-minor**: ^{1}H NMR (300 MHz, $CDCl_3$): δ = 2.30 (d, *J* = 7.4 Hz, 2H). ^{13}C NMR (75 MHz, $CDCl_3$): δ = 22.13, 22.55, 27.40, 29.24, 31.38, 34.38, 64.29, 123.00, 133.73. IR (neat): 2930, 2857, 2098, 1448, 1258, 971. HRMS (ESI): calcd. for M+H$^+$: $C_{14}H_{26}N_3$: 236.2121; found: 236.2129.

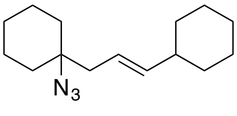

$C_{15}H_{25}N_3$
MW: 247.39 g/mol

(E)-1-Azido-1-(3-cyclohexylallyl)cyclohexane (**19a**). According to the general procedure D with the tetrazole **15a** (255 mg, 0.71 mmol), LiHMDS in THF (1.65 mL, 1.07 mmol), cyclohexanecarboxaldehyde (0.17 mL, 1.42 mmol) and THF (3.00

mL). Purification by FC (*n*-pentane/Et_2O, 99:1) to afford the alkene **119a** (124 mg, *E*/*Z* 85:15, 71%). Colourless liquid. **19a-major**: ^{1}H NMR (300 MHz, $CDCl_3$): δ = 5.52 - 5.28 (m, 2H), 2.22 (d, *J* = 6.5 Hz, 2H), 2.01 - 1.90 (m, 1H), 1.73 - 1.48 (m, 12H), 1.41 - 1.01 (m, 8H). ^{13}C NMR (75 MHz, $CDCl_3$): δ = 141.05, 121.14, 63.97, 43.88, 40.78, 34.42 (2C), 32.97 (2C), 26.20, 26.03 (2C), 25.43, 22.09 (2C). Characteristic signals for **19a-minor**: ^{1}H NMR (300 MHz, $CDCl_3$): δ = 2.31 (d, *J* = 6.1 Hz, 2H). ^{13}C NMR (75 MHz, $CDCl_3$): δ = 139.50, 120.97, 64.12, 36.47, 34.37, 33.14, 25.89, 25.40, 22.14. IR (neat): 2923, 2850, 2096, 1447, 1256, 1147. HRMS (ESI): calcd. for M+H$^+$: $C_{15}H_{26}N_3$: 248.2121; found: 248.2125.

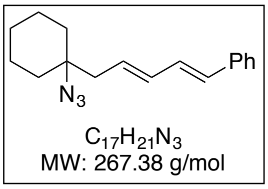

((1E,3E)-5-(1-Azidocyclohexyl)penta-1,3-dien-1-yl)benzene **(20a).** According to the general procedure D with the tetrazole **15a** (213 mg, 0.59 mmol), LiHMDS in THF (2.14 mL, 1.86 mmol), *trans*-cinnamaldehyde (0.32 mL, 2.54 mmol) and THF (6.00 mL). Purification by FC (*n*-pentane/Et_2O, 95:5) to afford the alkene **20a** (139 mg, *E*/*Z* 82:18, 88%). Colourless liquid. **20a-major**: ^{1}H NMR (300 MHz, $CDCl_3$): δ = 7.44 - 7.19 (m, 5H), 6.78 (dd, *J* = 15.6, 10.4 Hz, 1H), 6.50 (d, *J* = 15.6 Hz, 1H), 6.29 (dd, *J* = 15.1 10.4 Hz, 1H), 5.48 (dt, *J* = 15.1, 7.6 Hz, 1H), 2.40 (d, *J* = 7.6 Hz, 2H), 1.78 - 1.47 (m, 7H), 1.48 - 1.35 (m, 2H), 1.33 - 1.17 (m, 1H). ^{13}C NMR (75 MHz, $CDCl_3$): δ = 137.26, 134.30, 131.34, 128.65, 128.59, 128.47 (2C), 127.27, 126.17 (2C), 64.09, 43.65, 34.39 (2C), 25.24, 21.99 (2C). Characteristic signals for **20a-minor**: ^{1}H NMR (300 MHz, $CDCl_3$): δ = 7.05 (dd, *J* = 16.4, 11.2 Hz, 1H), 6.60 (d, *J* = 15.5 Hz, 1H), 5.59 (dd, *J* = 16.4, 8.0 Hz, 1H), 2.54 (dd, *J* = 8.0, 1.2 Hz, 2H). ^{13}C NMR (75 MHz, $CDCl_3$): δ = 133.48, 132.01, 128.51, 127.53, 127.37, 126.37, 125.78, 123.64, 67.83, 64.24, 60.22, 38.58, 14.09. IR (neat): 3021, 2930, 2855, 2096, 1594, 1496, 1447, 1256, 1147, 1071. EI-MS m/z (%): M–N_2: 239.4 (7), 224.4 (6), 210.4 (3), 196.3 (5), 183.3 (8), 148.4 (100), 128.3 (10), 120.3 (11), 106.3 (18), 93.3 (26), 91.3 (38), 77.3 (7), 65.3 (11). HRMS (ESI): calcd. for M+H$^+$: $C_{17}H_{22}N_3$: 268.1808; found: 268.1809.

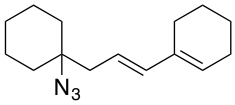

$C_{15}H_{23}N_3$
MW: 245.37 g/mol

(E)-1-(3-(1-Azidocyclohexyl)prop-1-en-1-yl)cyclohex-1-ene (**21a**). According to the general procedure D with the tetrazole **15a** (220 mg, 0.61 mmol), LiHMDS in THF (1.56 mL, 0.92 mmol), cyclohex-1-en-1-carboxaldehyde (0.14 mL, 1.22 mmol) and THF (3.00 mL). Purification by FC (*n*-pentane, 100%) to afford the alkene **21a** (112 mg, *E*/*Z* > 95:5, 75%). Colourless liquid. **21a-major**: ^{1}H NMR (300 MHz, $CDCl_3$): δ = 6.09 (d, J = 15.1 Hz, 1H), 5.69 (br, 1H), 5.54 (dt, J = 15.1, 7.5 Hz, 1H), 2.32 (d, J = 7.5 Hz, 2H), 2.13 (d, J = 6.2 Hz, 4H), 1.69 - 1.49 (m, 11H), 1.42 - 1.33 (m, 2H), 1.30 - 1.17 (m, 1H). ^{13}C NMR (75 MHz, $CDCl_3$): δ = 137.41, 135.48, 128.52, 119.77, 64.26, 43.89, 34.46 (2C), 25.78, 25.38, 24.59, 22.55, 22.48, 22.09 (2C). Characteristic signals for **21a-minor**: ^{1}H NMR (300 MHz, $CDCl_3$): δ = 5.91 (d, J = 11.4 Hz, 1H), 5.39 (dt, J = 11.8, 7.4 Hz, 1H), 2.51 (dd, J = 7.4, 1.7 Hz, 2H). ^{13}C NMR (75 MHz, $CDCl_3$): δ = 134.84, 127.55, 122.51, 29.06, 25.54, 25.43, 22.83, 22.13. IR (neat): 3025, 2928, 2857, 2096, 1447, 1432, 1254, 1137. EI-MS m/z (%): M–N_2: 216.2 (10), 174.2 (18), 121.2 (53), 96.2 (61), 79.2 (72), 55.2 (79), 41.3 (100). HRMS (ESI): calcd. for M+H$^+$: $C_{15}H_{24}N_3$: 246.1965; found: 246.1966.

References

[1] D. C. Harrowven, I. L. Guy, *Chem. Commun.* **2004**, 1968.

[2] G. D. Mendenhall, *Tetrahedron Lett.* **1983**, *24*, 451.

[3] P. Panchaud, P. Renaud, *Adv. Synth. Catal.* **2004**, *346*, 925.

[4] C. Ollivier, P. Renaud, *J. Am. Chem. Soc.* **2001**, *123*, 4717.

[5] G. W. Pucher, T. B. Johnson, *J. Am. Chem. Soc.* **1922**, *44*, 817.

[6] A. Jończyk, T. Pytlewski, *Synthesis* **1978**, 883.

[7] G. A. Olah, V. P. Reddy, G. K. Surya Prakash, *Synthesis* **1991**, 29.

[8] S. Cren, P. Schär, P. Renaud, K. Schenk, *J. Org. Chem.* **2009**, *74*, 2942.

[9] J. Duran, D. Oliver, A. Polo, J. Real, J. Benet-Buchholz, X. Fontrodona, *Tetrahedron: Asymetry* **2003**, *14*, 2529.

[10] (a) M. Lebrun, P. Le Marquand, *J. Org. Chem.* **2006**, *71*, 2009; (b) X. Wang, E. J. Bowman, B. J. Bowman, J. A. Porco, *Angew. Chem. Int. Ed.* **2004**, *43*, 3601.

[11] R. W. Alder, P. R. Allen, K. R. Anderson, C. P. Butts, E. Khosravi, A. Martín, C. M. Maunder, A. G. Orpen, C. B. St Pourçain, *J. Chem. Soc., Perkin Trans. 2* **1998**, 2083.

[12] J. Barluenga, J. L. Fernandez-Simon, J. M. Concellon, M. Yus, *J. Chem. Soc., Perkin Trans. 1* **1988**, 3339.

[13] K. Weidner, A. Giroult, P. Panchaud, P. Renaud, *J. Am. Chem. Soc.* **2010**, *132*, 17511.

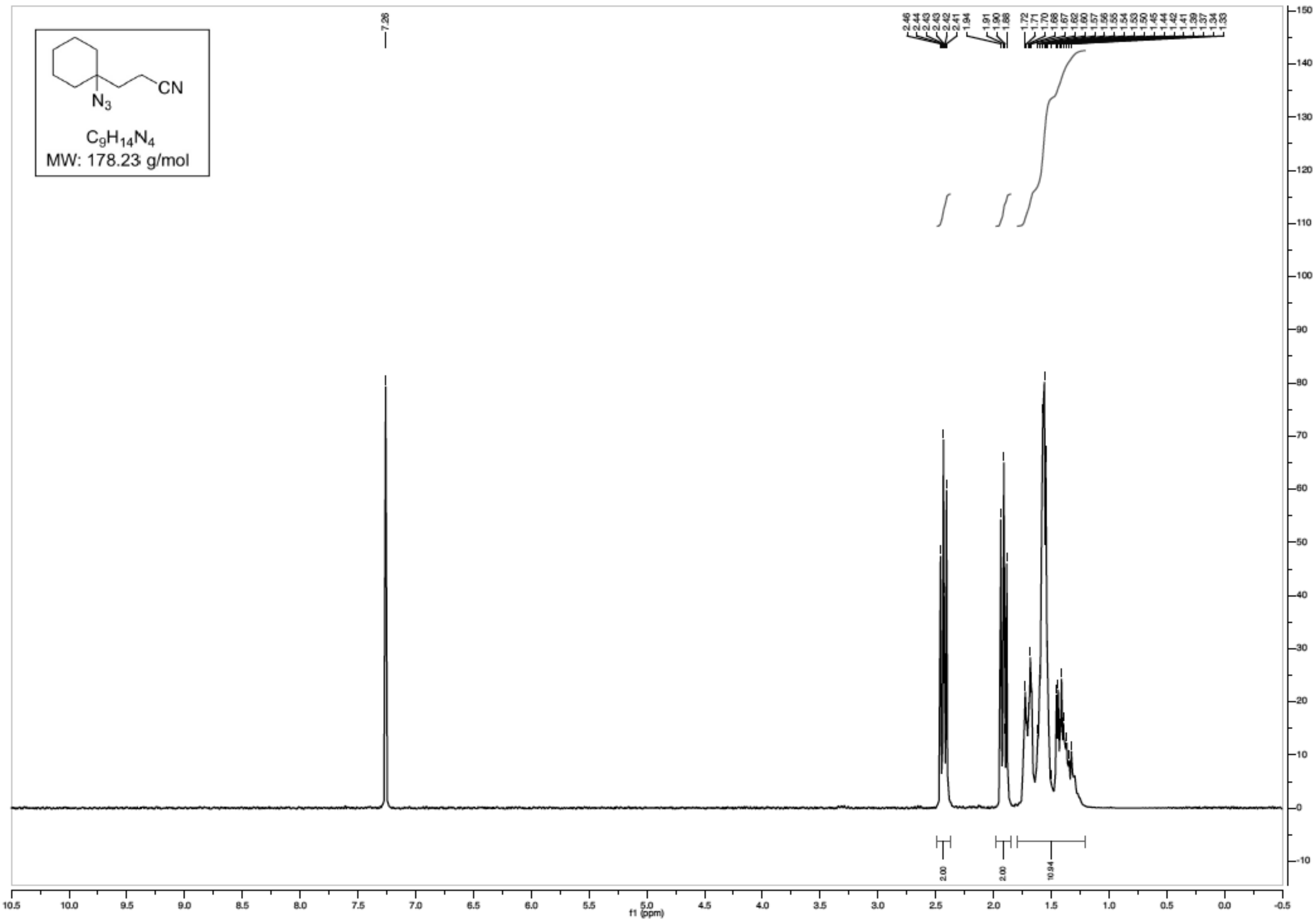
CN
N_3
$C_9H_{14}N_4$
MW: 178.23 g/mol
7.26
2.46
2.44
2.43
2.43
2.42
2.41
1.94
1.91
1.90
1.88
1.72
1.71
1.70
1.68
1.67
1.62
1.60
1.57
1.56
1.55
1.54
1.53
1.50
1.45
1.44
1.42
1.41
1.39
1.37
1.34
1.33
2.00
2.00
10.94
f1 (ppm)

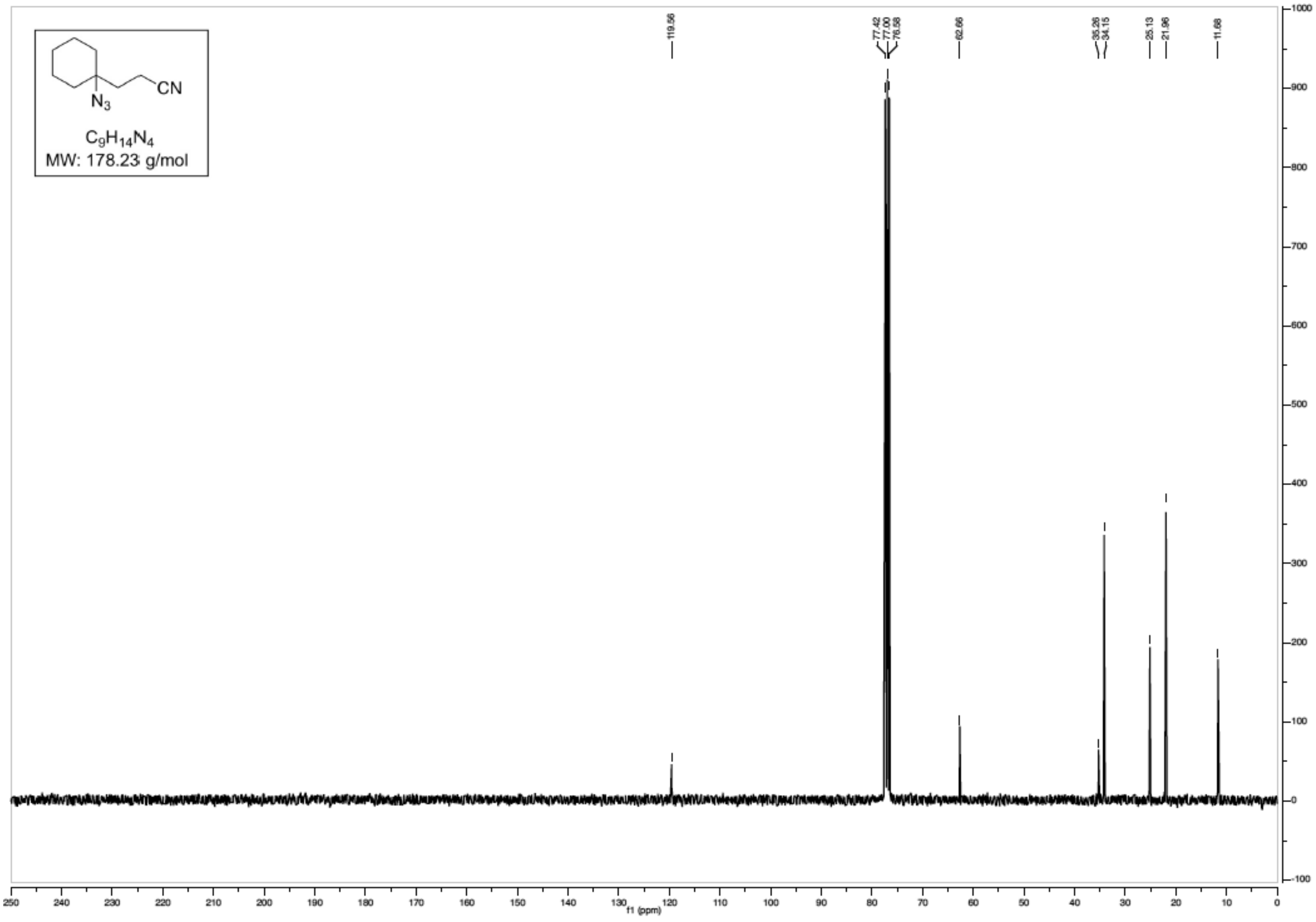

119.56
77.42
77.00
76.58
62.66
35.26
34.15
25.13
21.96
11.68
N3
CN
C9H14N4
MW: 178.23 g/mol
f1 (ppm)

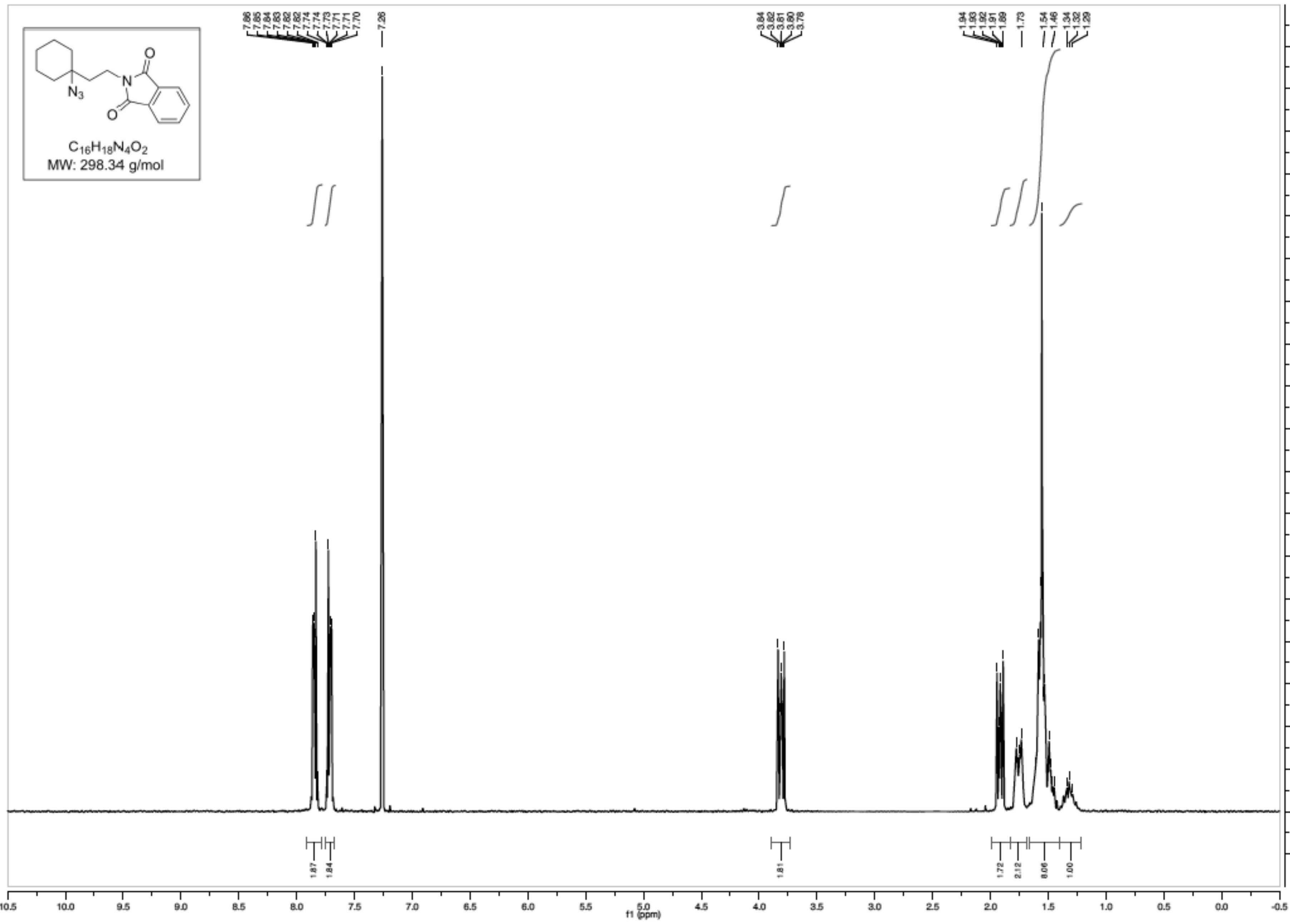

$C_{16}H_{18}N_4O_2$
MW: 298.34 g/mol
N3
O
N
7.86 7.85 7.84 7.83 7.82 7.82 7.74 7.74 7.73 7.71 7.71 7.70
7.26
3.84 3.82 3.81 3.80 3.78
1.94 1.93 1.92 1.91 1.89
1.73
1.54 1.46 1.34 1.32 1.29
1.87
1.84
1.81
1.72
2.12
8.06
1.00
f1 (ppm)

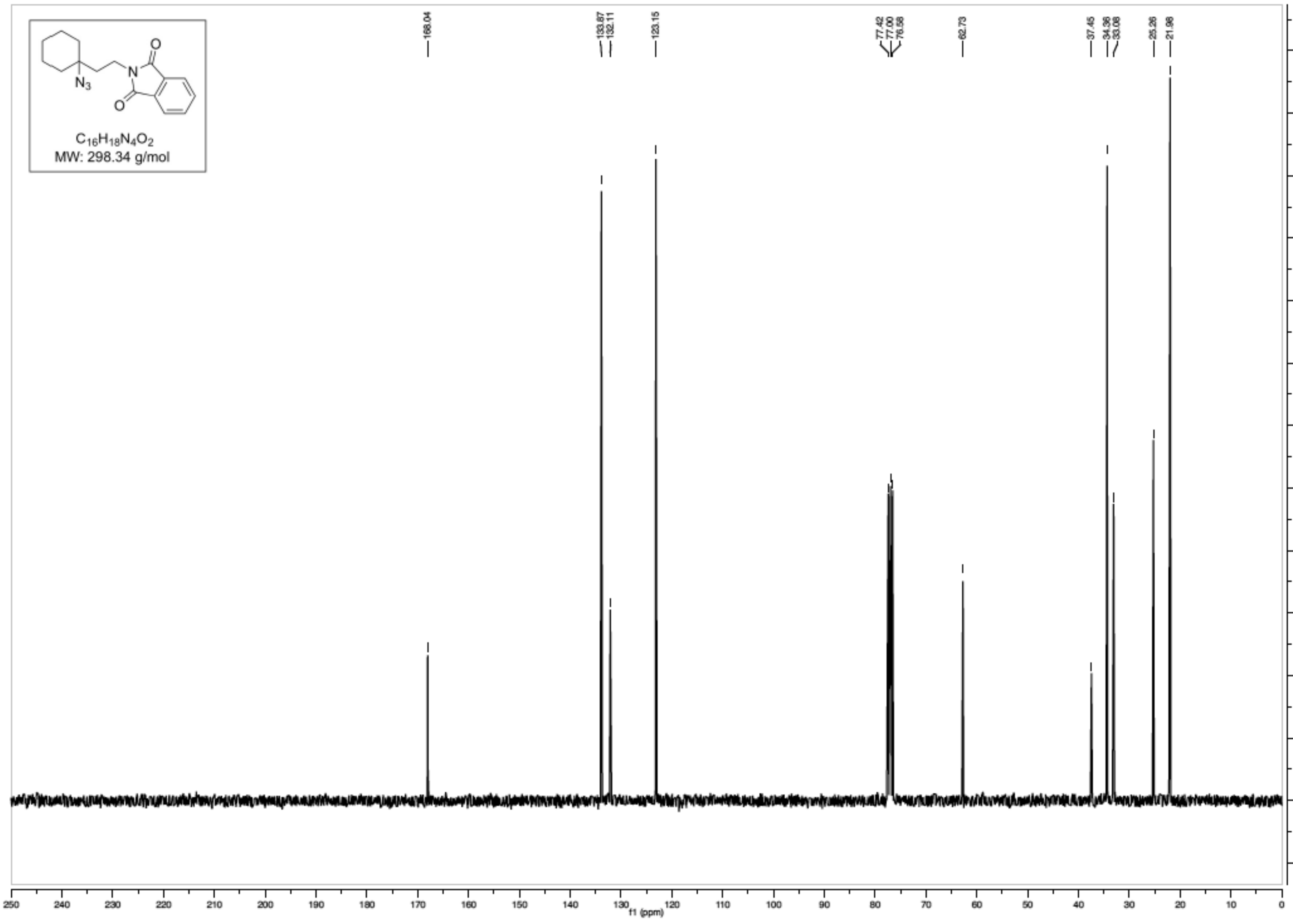
C16H18N4O2
MW: 298.34 g/mol
N3
168.04
133.87
132.11
123.15
77.42
77.00
76.58
62.73
37.45
34.36
33.08
25.26
21.98
f1 (ppm)

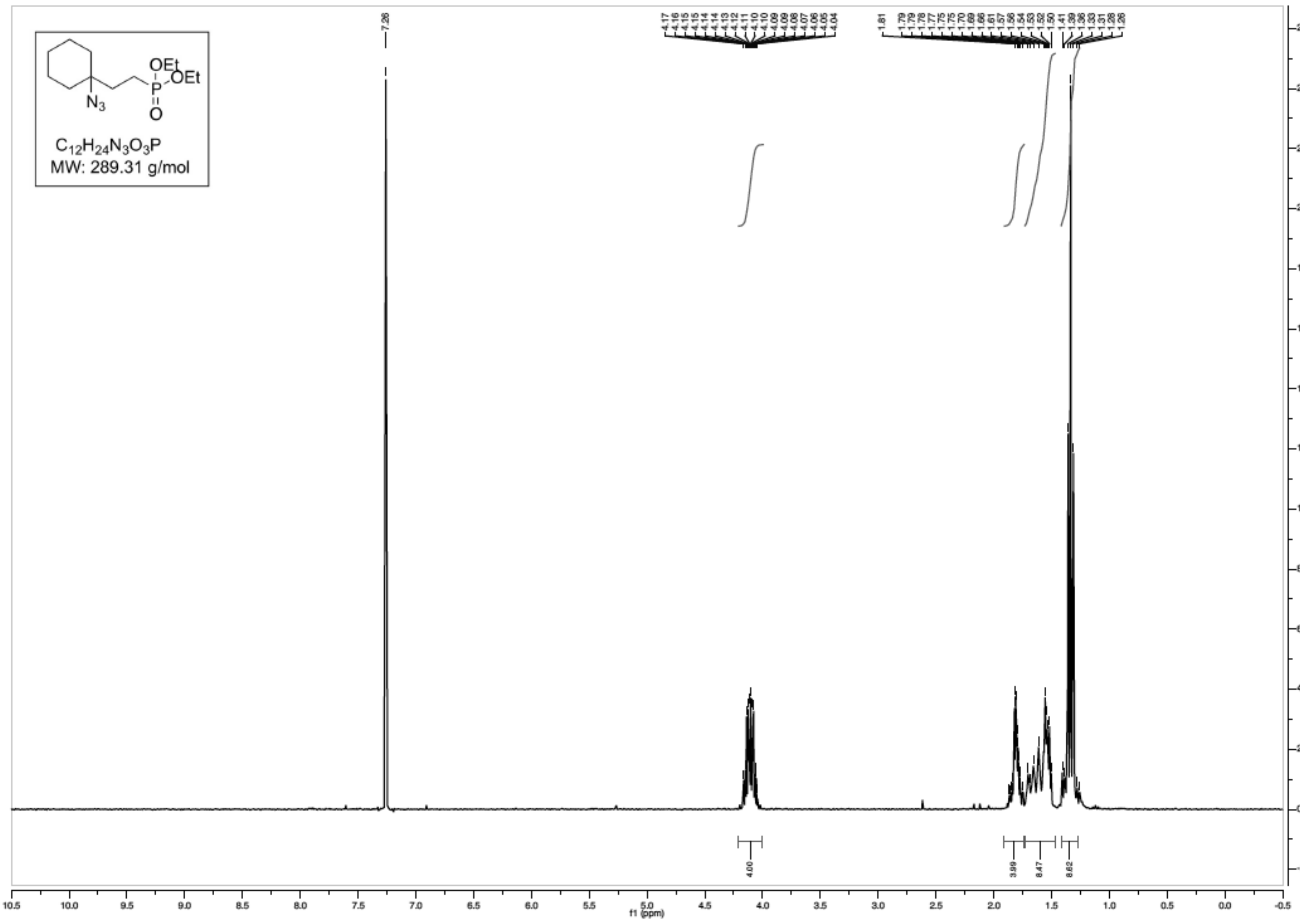
OEt
OEt
P
O
N3
C12H24N3O3P
MW: 289.31 g/mol
7.26
4.17 4.16 4.15 4.15 4.14 4.14 4.13 4.12 4.11 4.10 4.10 4.09 4.09 4.08 4.07 4.06 4.05 4.04
1.81 1.79 1.79 1.78 1.77 1.75 1.75 1.70 1.69 1.66 1.61 1.57 1.56 1.54 1.53 1.52 1.50 1.41 1.39 1.36 1.33 1.31 1.28 1.26
4.00
3.99
8.47
8.62
f1 (ppm)

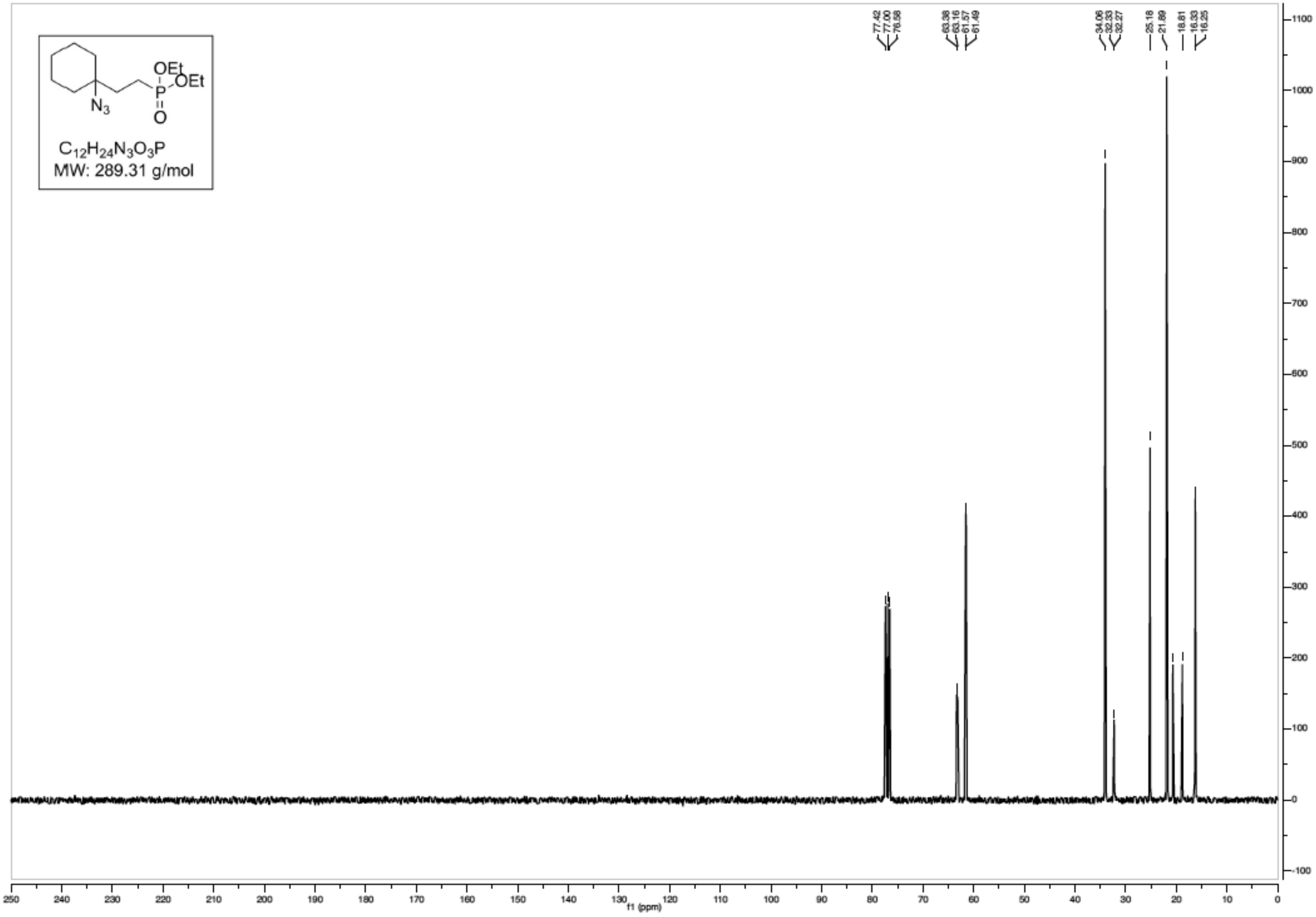
77.42
77.00
76.58
63.38
63.16
61.57
61.49
34.06
32.33
32.27
25.18
21.89
18.81
16.33
16.25
OEt
OEt
P
O
N3
C12H24N3O3P
MW: 289.31 g/mol
f1 (ppm)

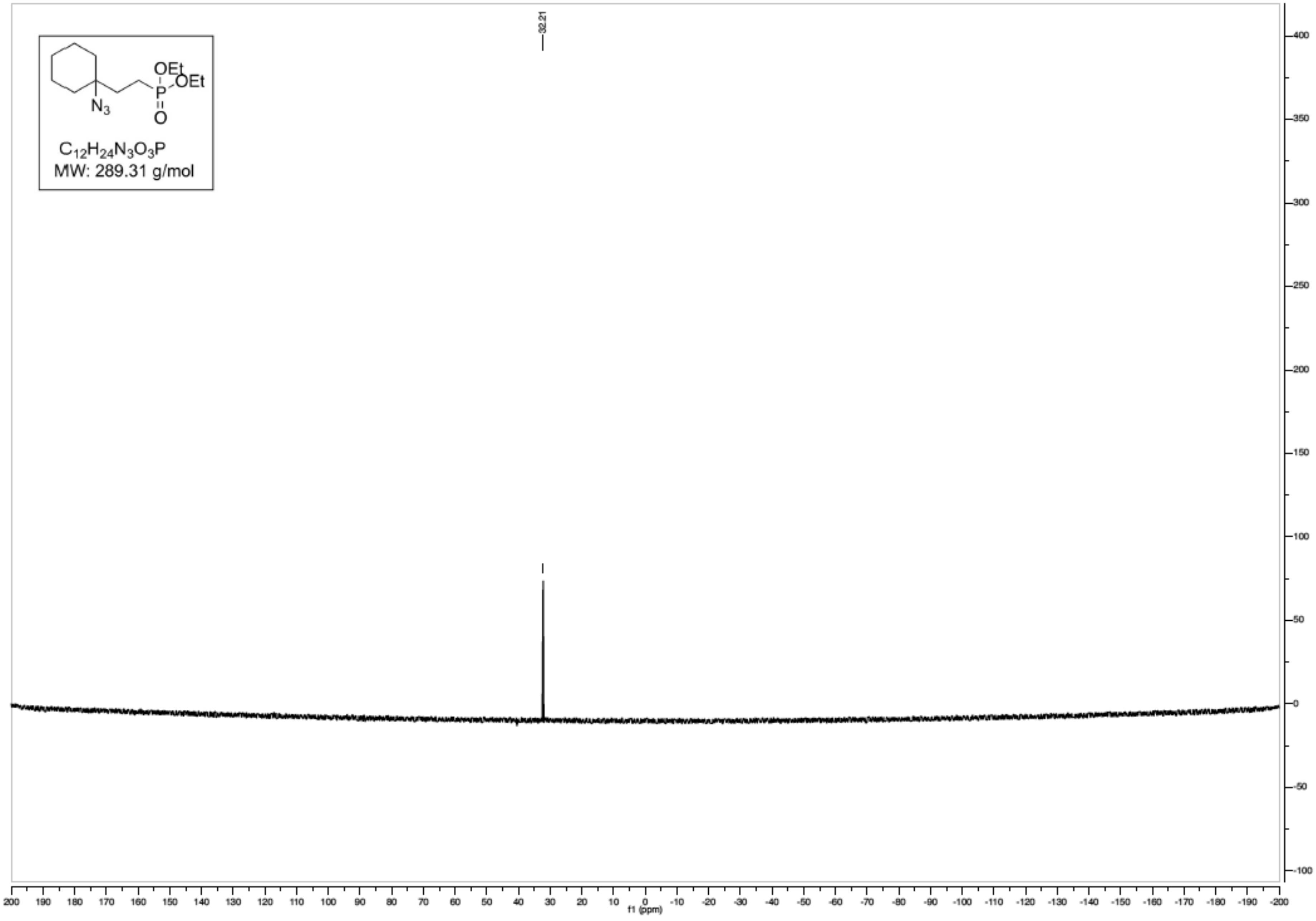

32.21
OEt
OEt
P
O
N3
C12H24N3O3P
MW: 289.31 g/mol
f1 (ppm)

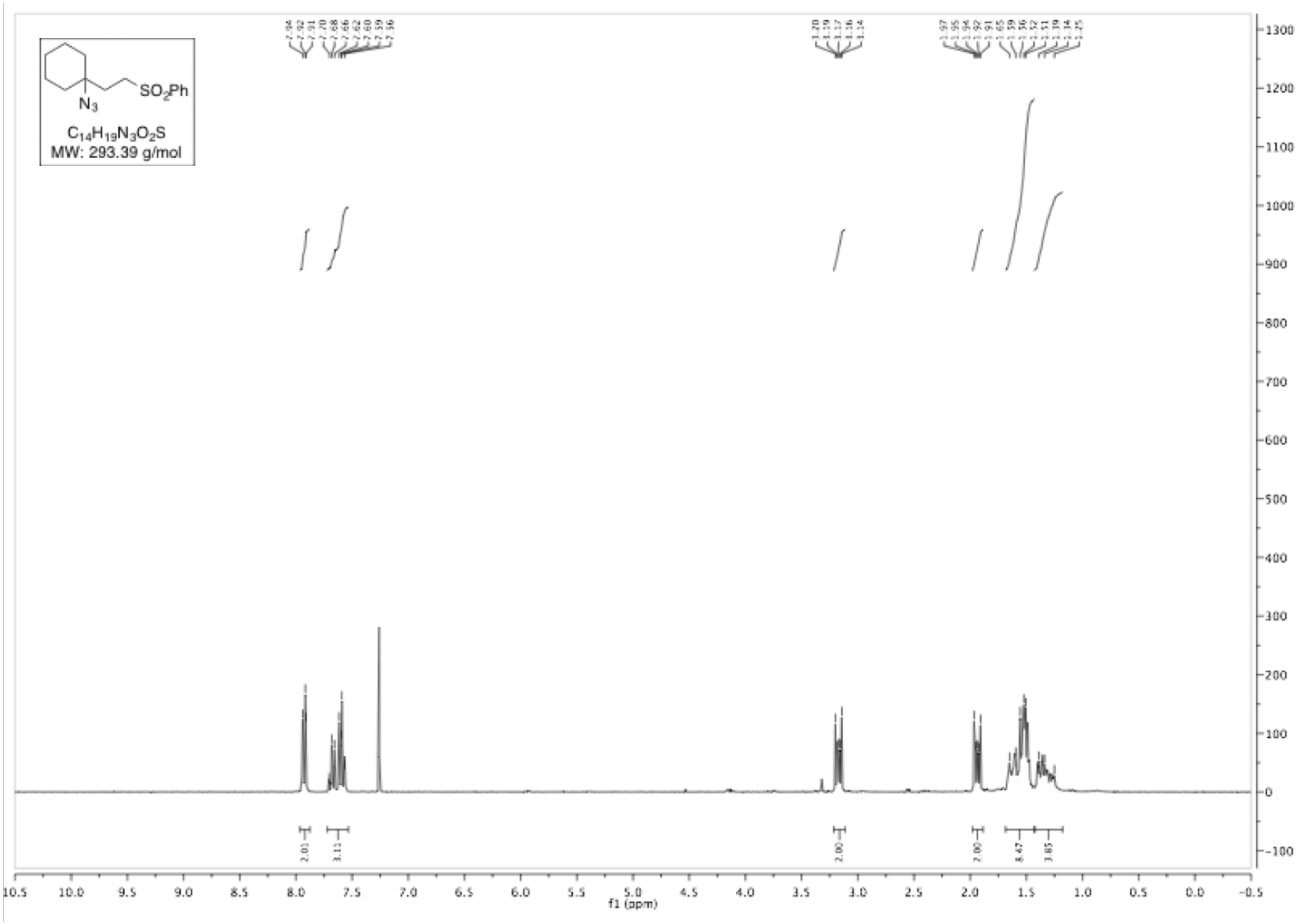
SO2Ph
N3
C14H19N3O2S
MW: 293.39 g/mol
7.94
7.92
7.91
7.70
7.68
7.66
7.62
7.60
7.59
7.56
3.20
3.19
3.17
3.16
3.14
1.97
1.95
1.94
1.92
1.91
1.65
1.59
1.56
1.52
1.51
1.39
1.34
1.25
2.01
3.11
2.00
2.00
8.47
3.85
f1 (ppm)

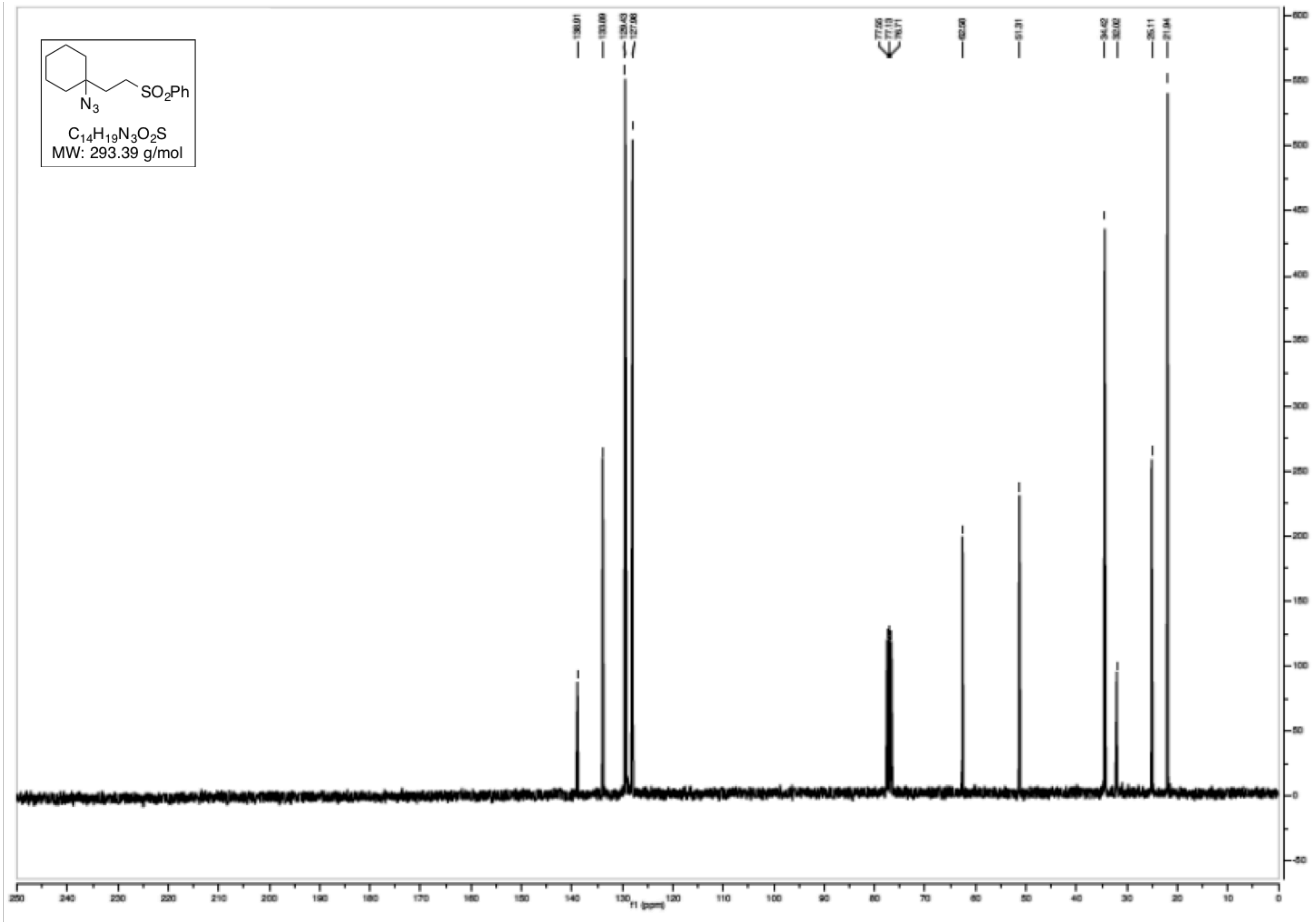
138.91
133.89
129.43
127.98
77.55
77.13
76.71
62.58
51.31
34.42
32.02
25.11
21.94
SO2Ph
N3
C14H19N3O2S
MW: 293.39 g/mol
f1 (ppm)

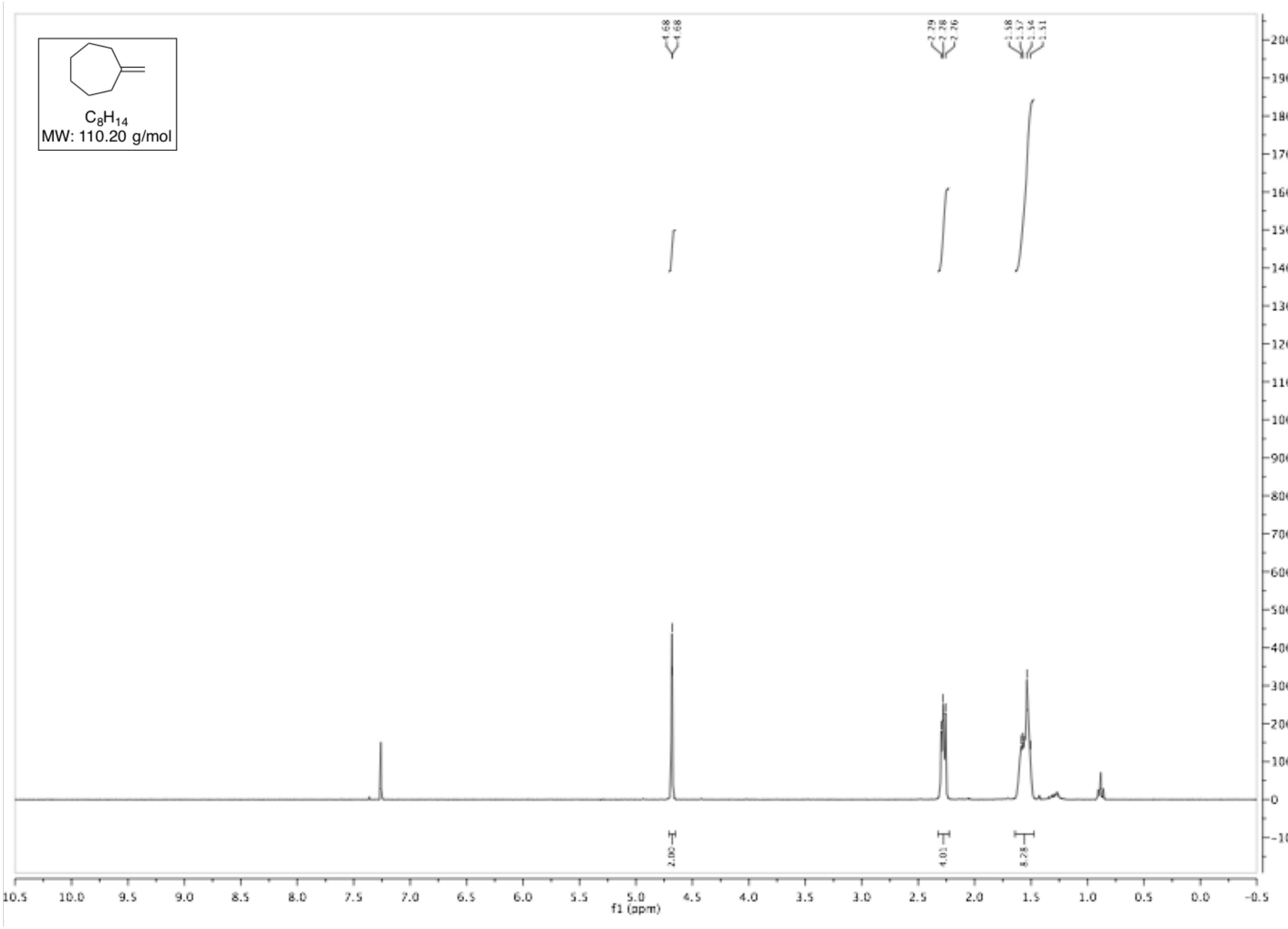

C_8H_{14}
MW: 110.20 g/mol
4.68
4.68
2.29
2.28
2.26
1.58
1.57
1.54
1.51
2.00
4.01
8.28
f1 (ppm)

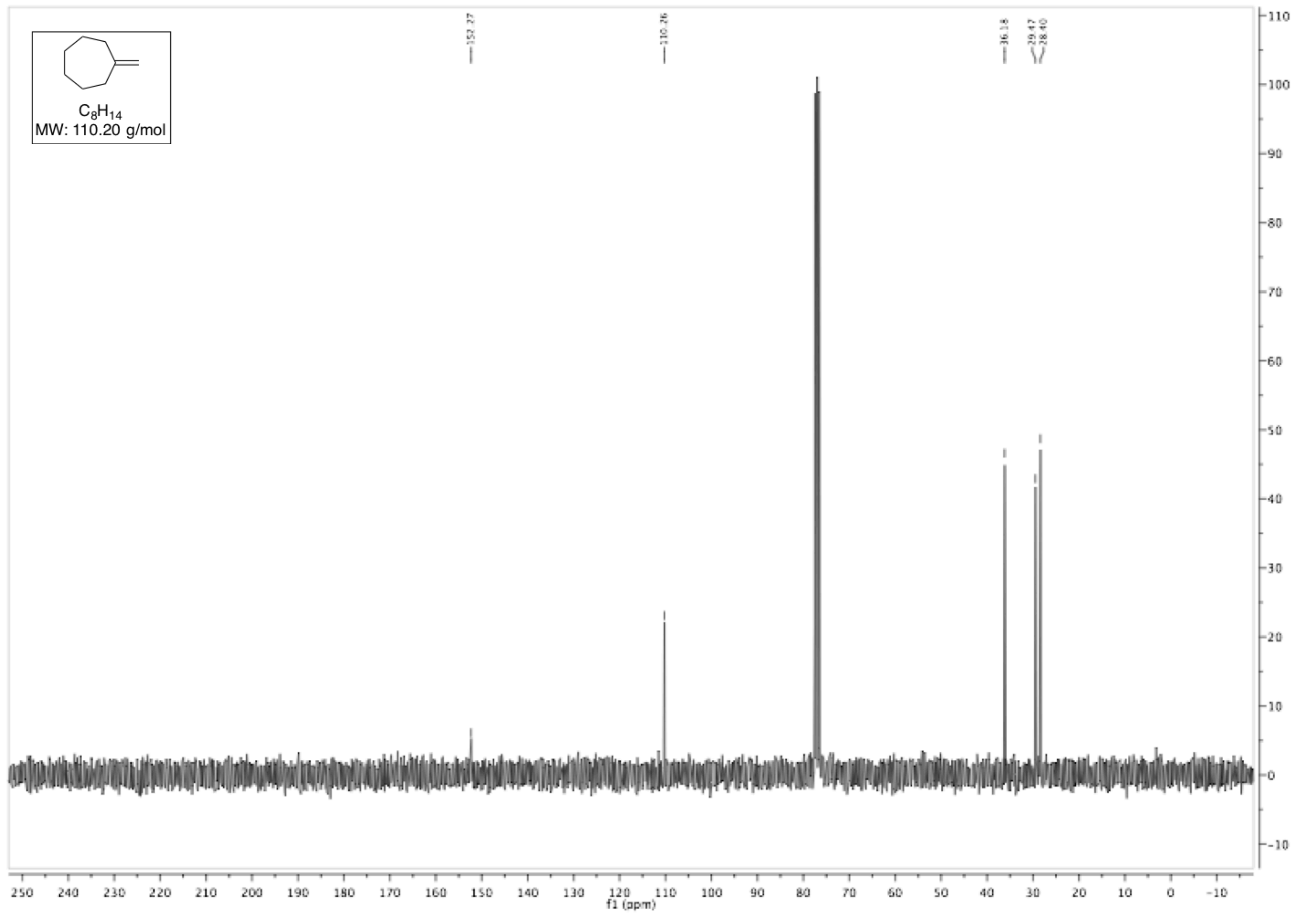
C8H14
MW: 110.20 g/mol
152.27
110.26
36.18
29.47
28.40
f1 (ppm)

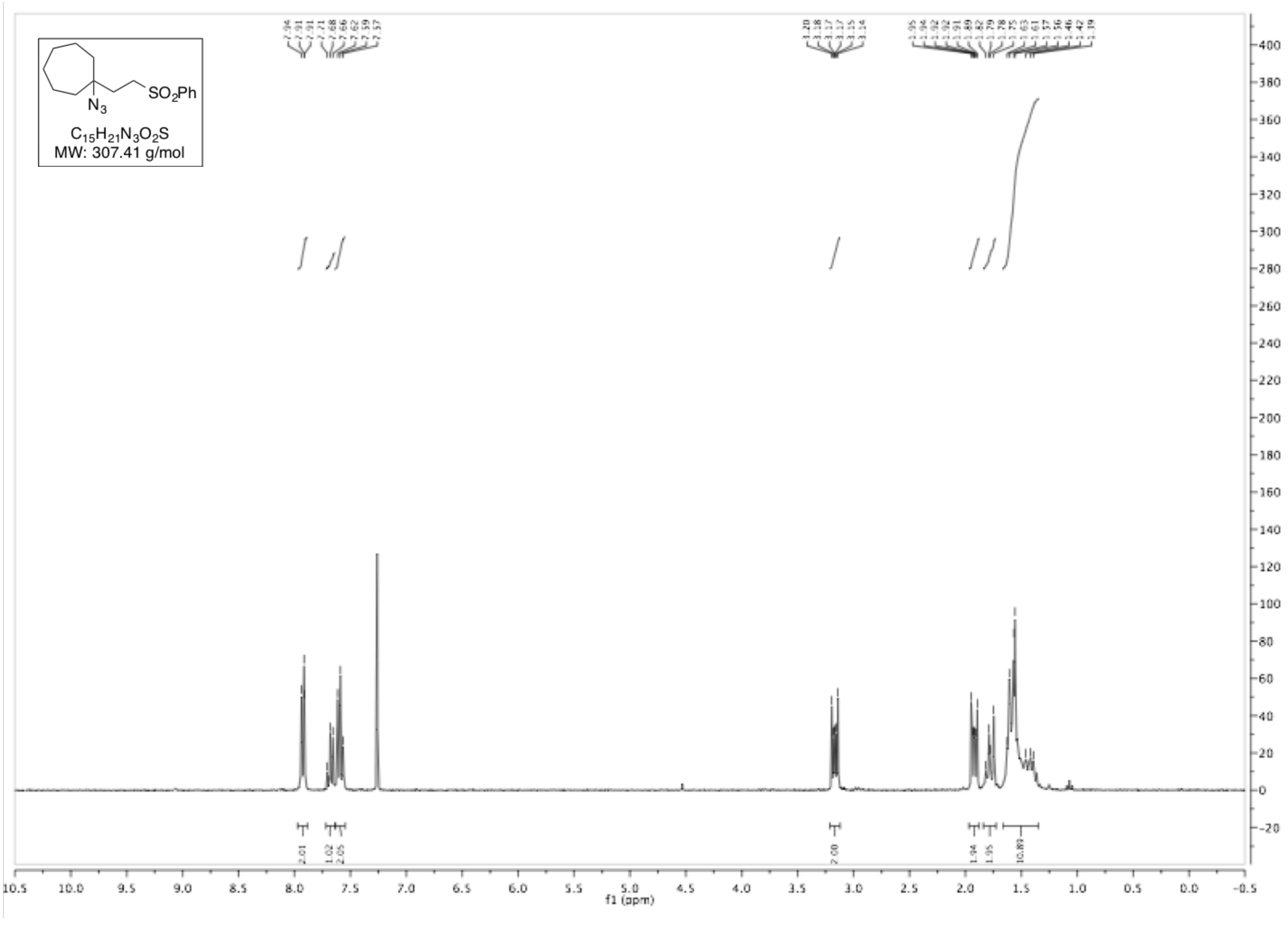
SO2Ph
N3
C15H21N3O2S
MW: 307.41 g/mol
7.94 7.91 7.91 7.71 7.68 7.66 7.62 7.59 7.57
3.20 3.18 3.17 3.17 3.15 3.14
1.95 1.94 1.92 1.92 1.91 1.89 1.82 1.79 1.78 1.75 1.63 1.61 1.57 1.56 1.46 1.42 1.39
2.01 1.02 2.05 2.00 1.94 1.95 10.89
f1 (ppm)

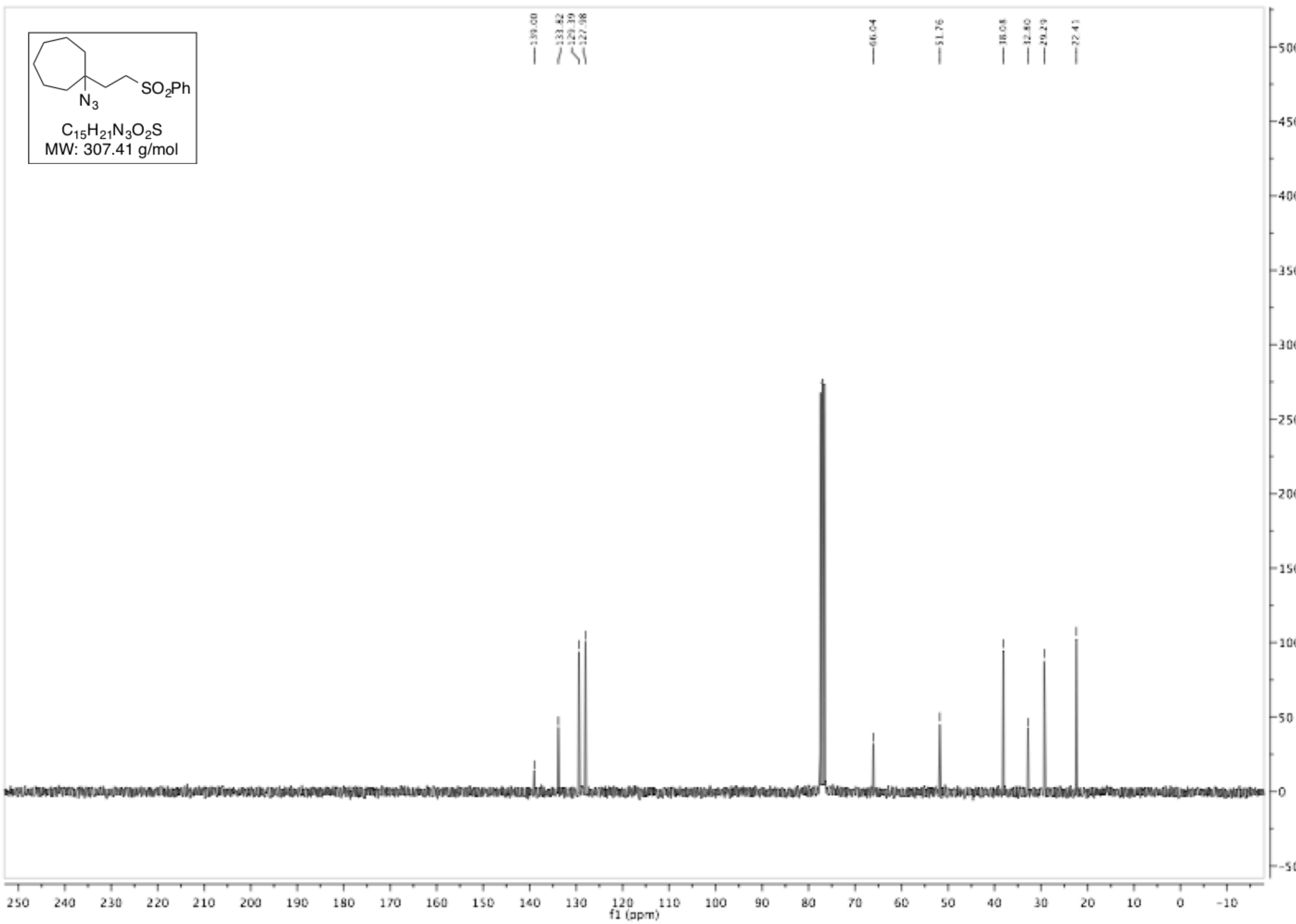
SO2Ph
N3
C15H21N3O2S
MW: 307.41 g/mol
139.00
133.82
129.39
127.98
66.04
51.76
38.08
32.80
29.29
22.41
f1 (ppm)

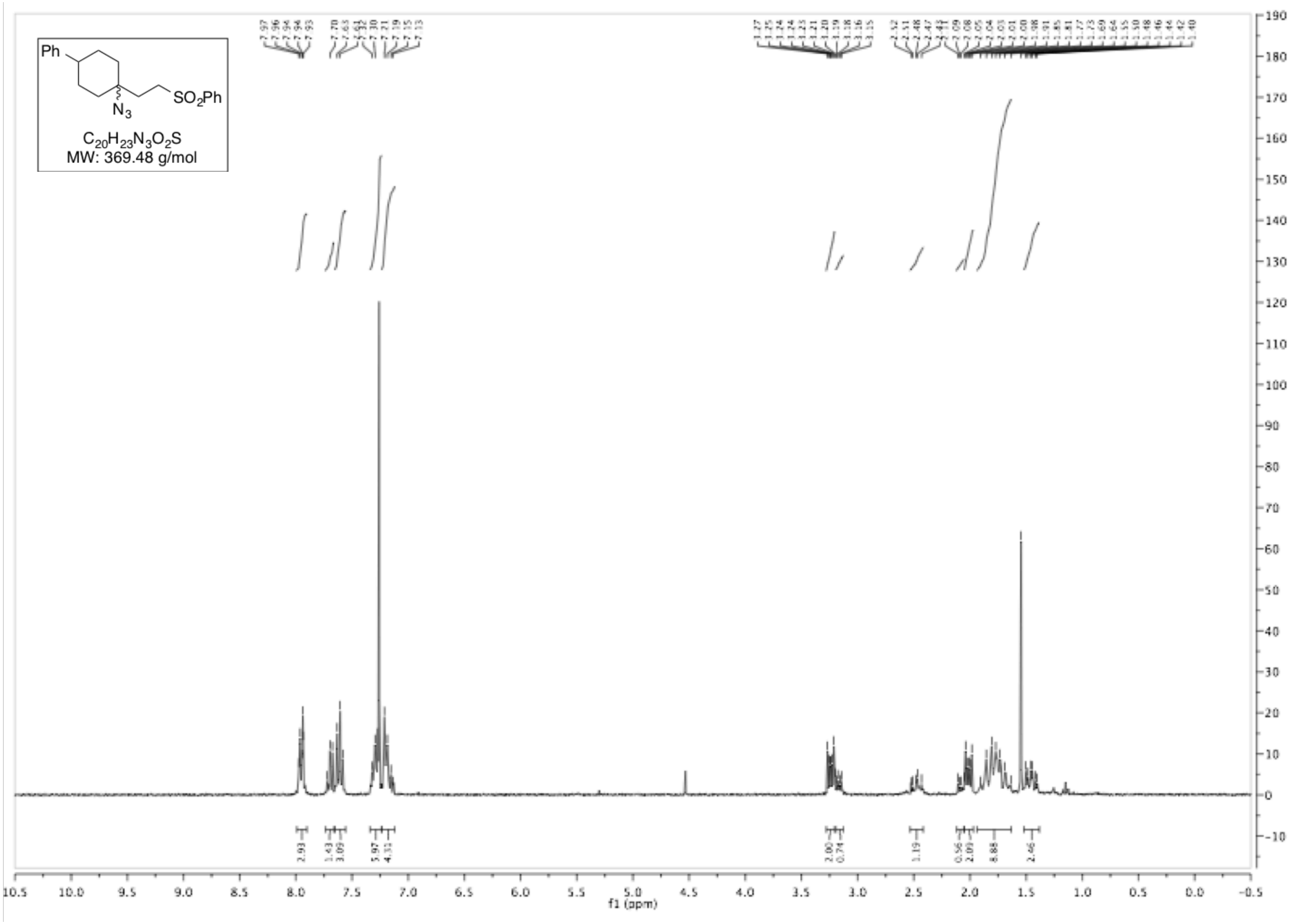
Ph
N3
SO2Ph
C20H23N3O2S
MW: 369.48 g/mol
f1 (ppm)

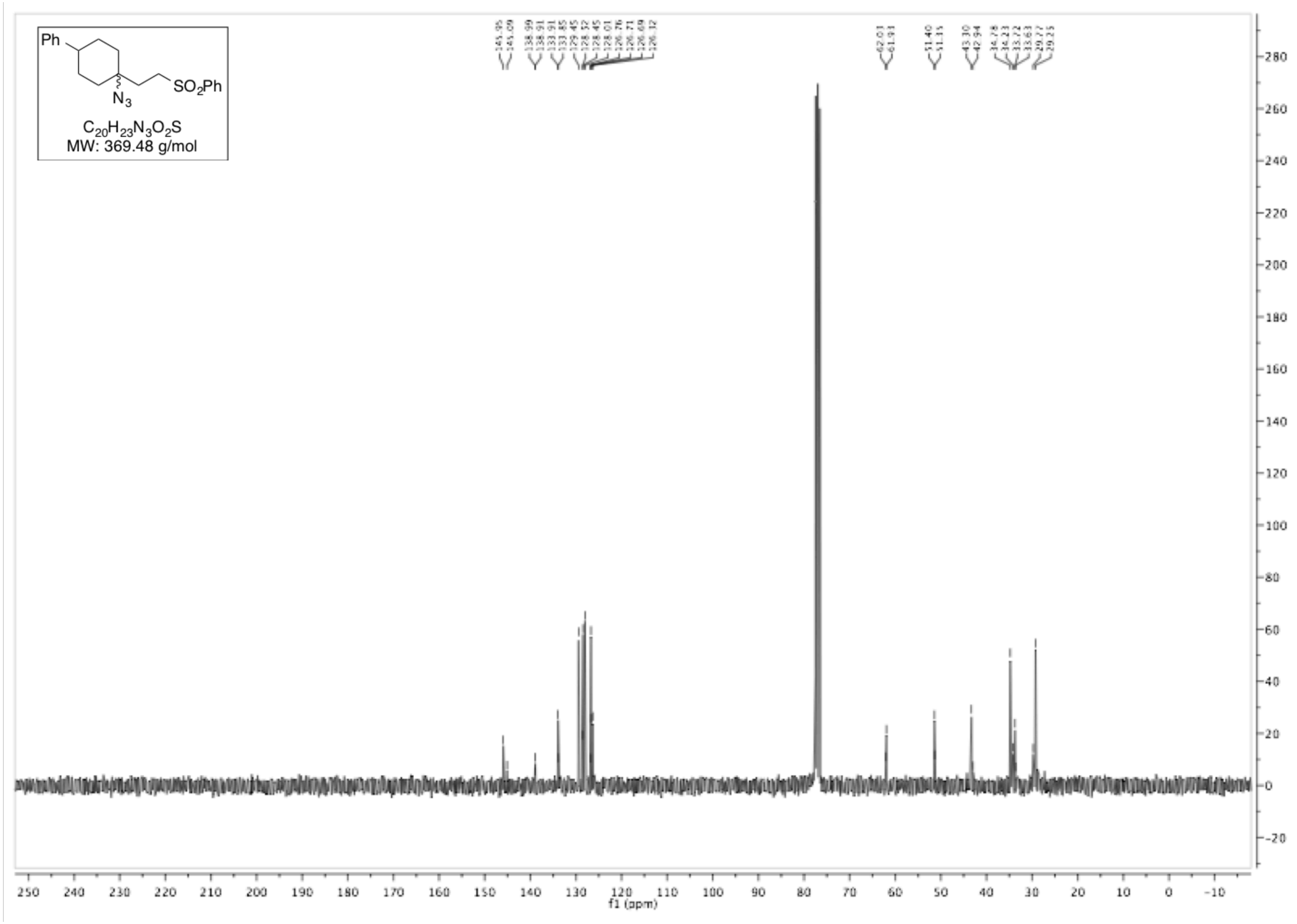
Ph
N3
SO2Ph
C20H23N3O2S
MW: 369.48 g/mol
145.95
145.09
138.99
138.91
133.91
133.85
129.45
128.52
128.45
128.01
126.76
126.71
126.69
126.32
62.01
61.93
51.40
51.35
43.30
42.94
34.78
34.23
33.72
33.63
29.77
29.25
f1 (ppm)

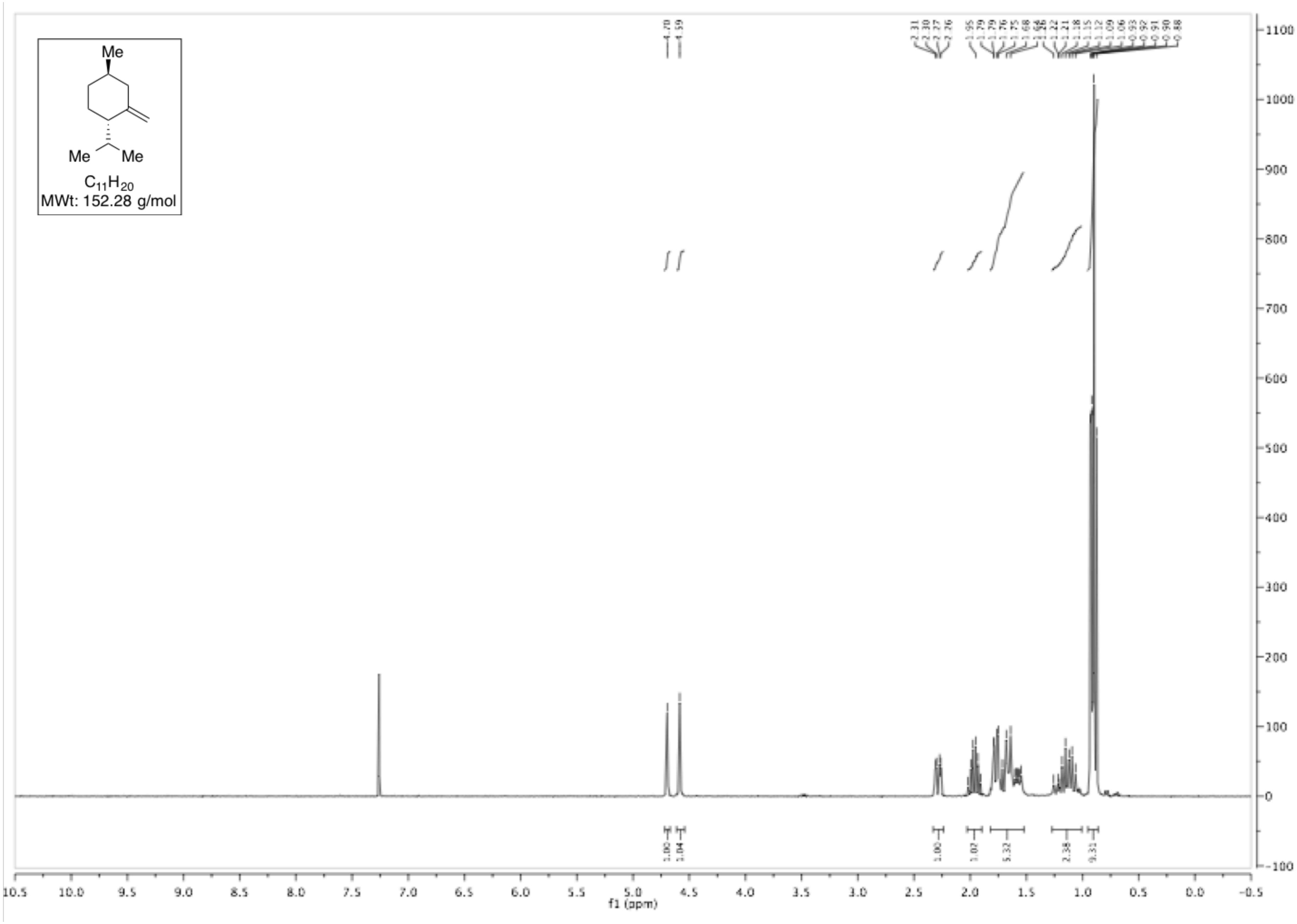
Me
Me
Me
$C_{11}H_{20}$
MWt: 152.28 g/mol
4.70
4.59
2.31
2.30
2.27
2.26
1.95
1.79
1.79
1.76
1.75
1.68
1.00
1.04
1.02
5.32
2.38
9.31
f1 (ppm)

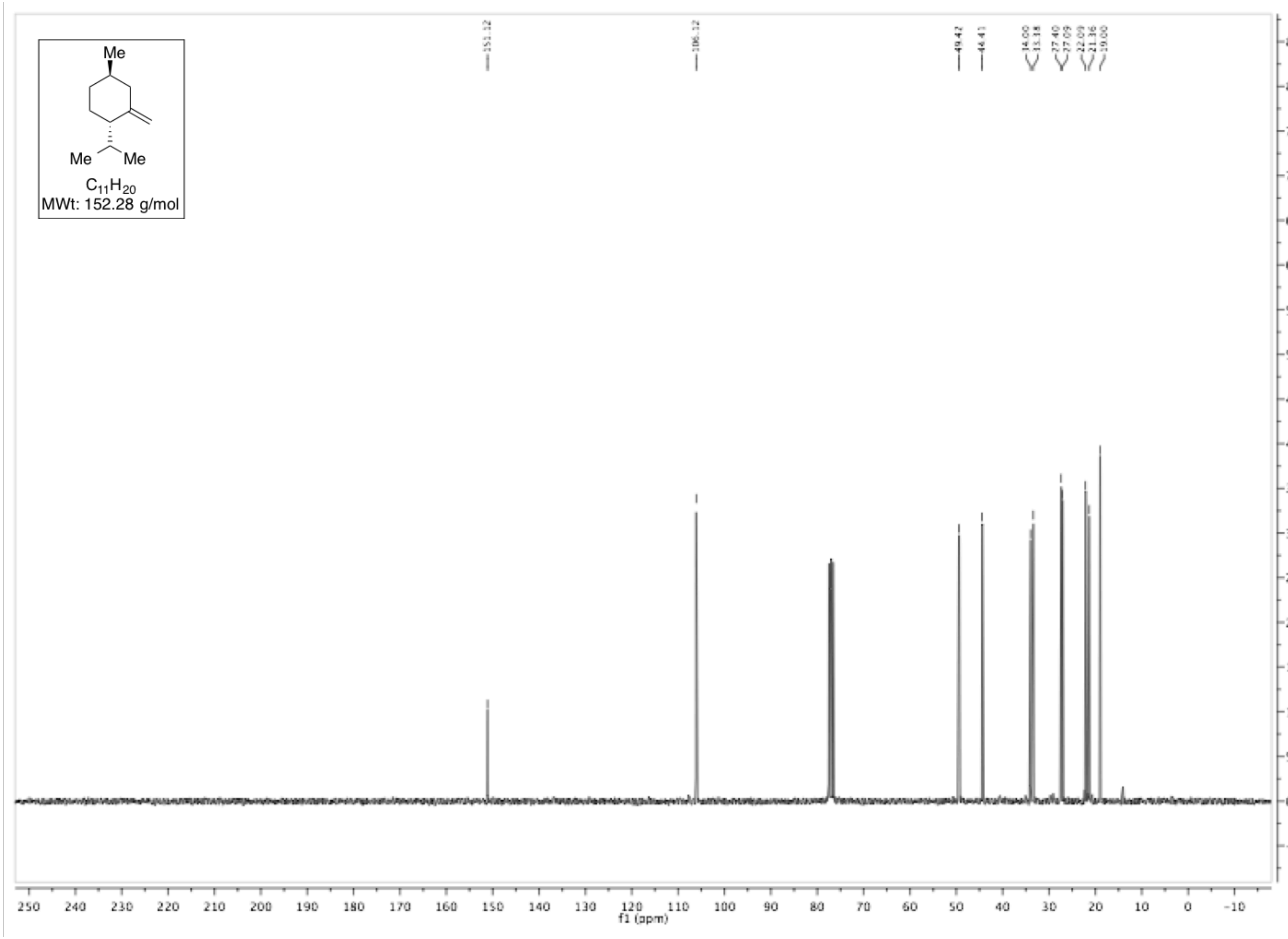
151.12
106.12
49.42
44.41
34.00
33.18
27.40
27.09
22.09
21.36
19.00
Me
Me
Me
$C_{11}H_{20}$
MWt: 152.28 g/mol
850
800
750
700
650
600
550
500
450
400
350
300
250
200
150
100
50
0
-50
250 240 230 220 210 200 190 180 170 160 150 140 130 120 110 100 90 80 70 60 50 40 30 20 10 0 -10
f1 (ppm)

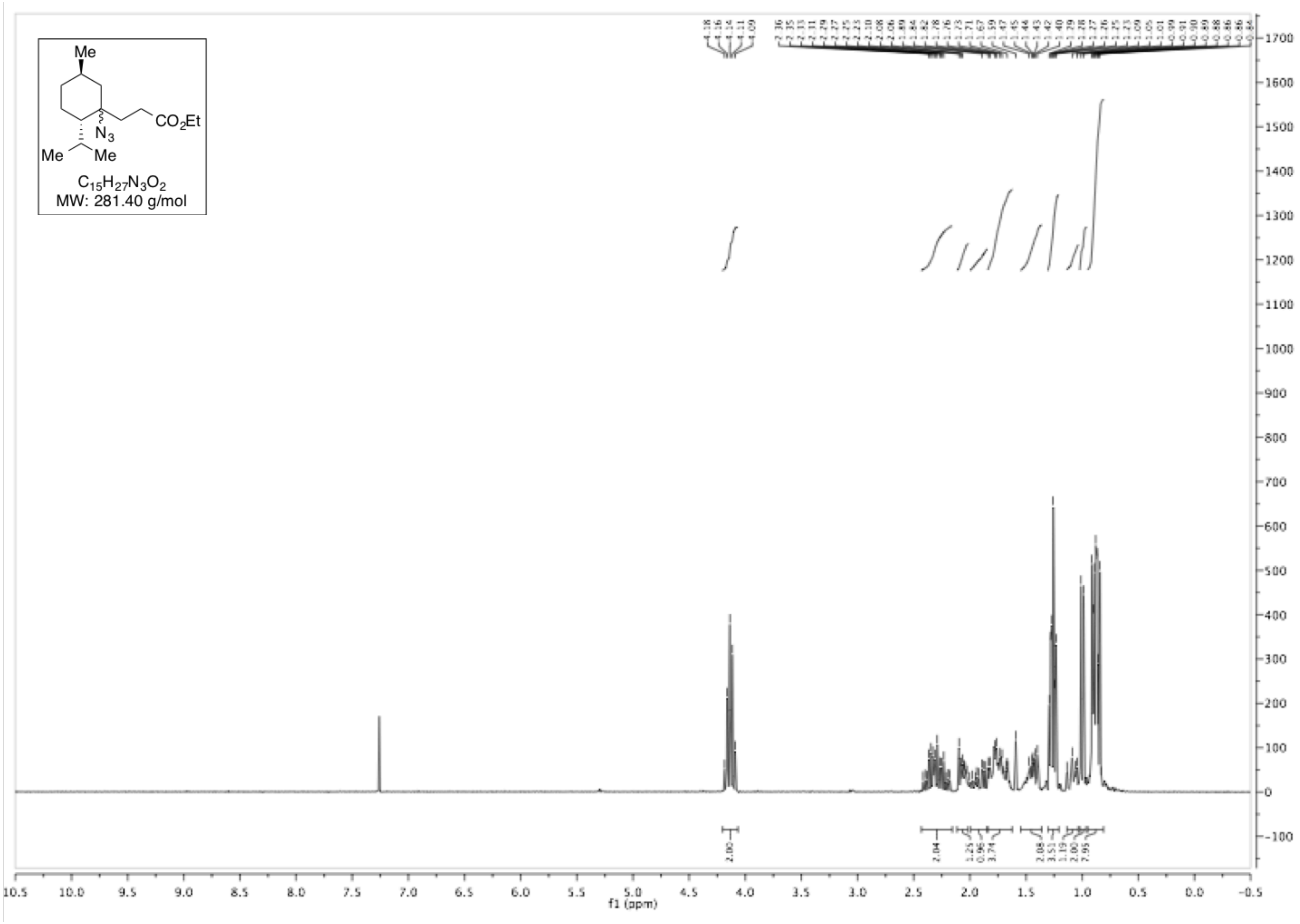
Me
CO2Et
N3
Me
Me
C15H27N3O2
MW: 281.40 g/mol
f1 (ppm)

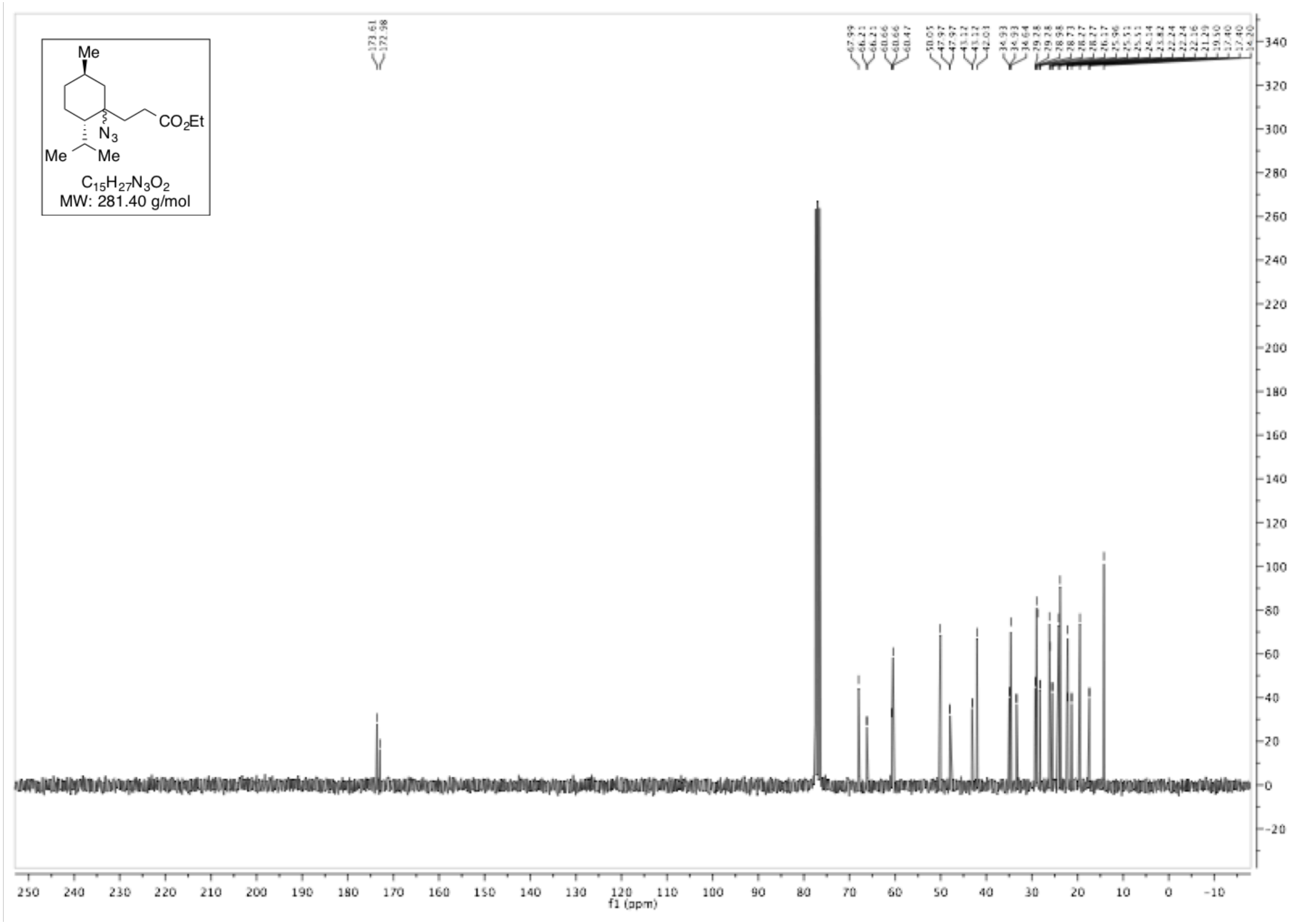

Me
CO2Et
N3
Me
Me
C15H27N3O2
MW: 281.40 g/mol
173.61
172.98
67.99
66.21
66.21
60.66
60.66
60.47
50.05
47.97
47.97
43.12
43.12
42.01
34.93
34.93
34.64
29.28
29.28
28.98
28.73
28.27
28.27
26.17
25.96
25.51
25.51
24.14
23.82
22.24
22.24
22.16
21.29
19.50
17.40
17.40
14.20
f1 (ppm)

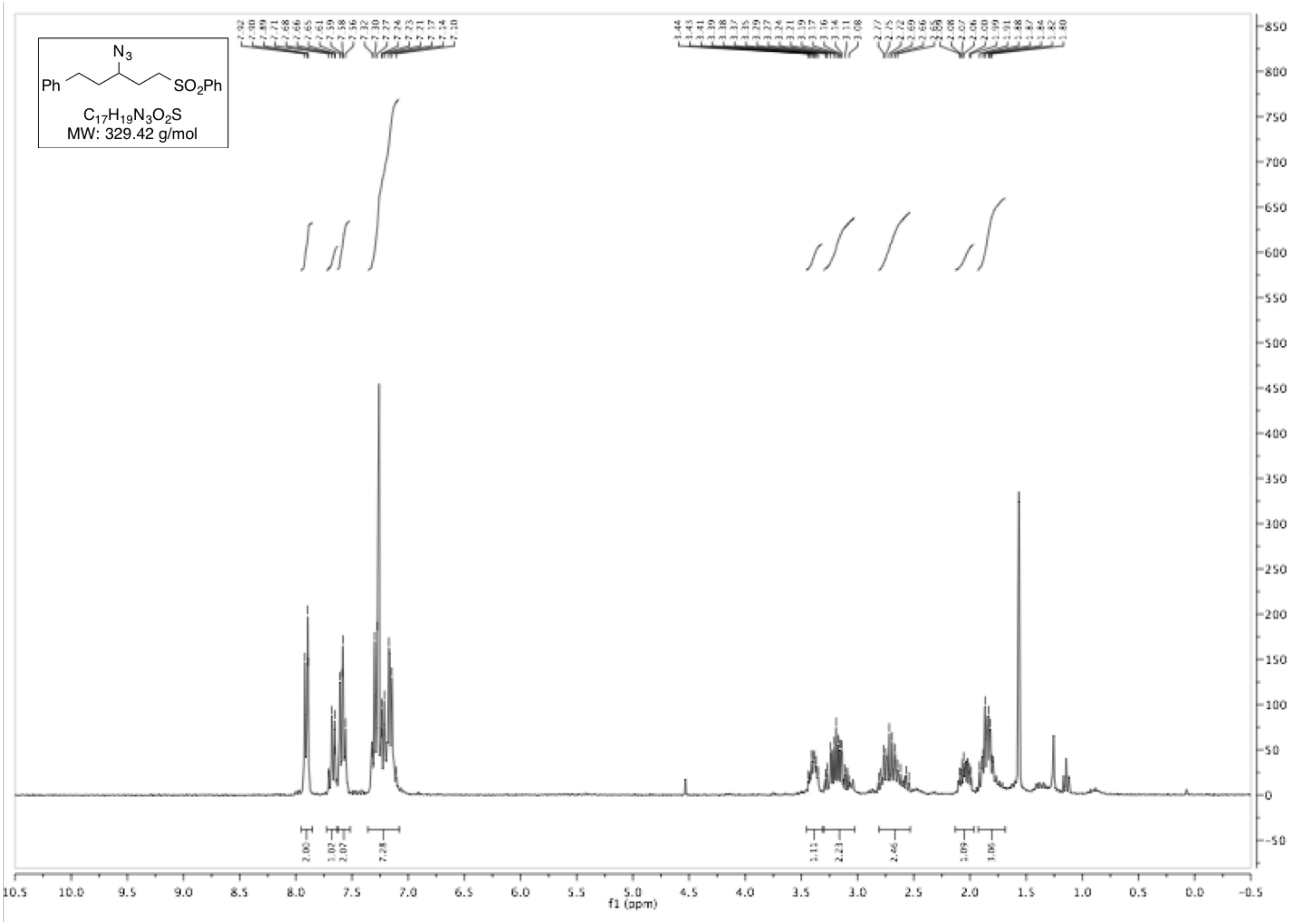
N3
Ph
SO2Ph
C17H19N3O2S
MW: 329.42 g/mol
2.00
1.02
2.07
7.28
1.11
2.23
2.46
1.09
3.06
f1 (ppm)

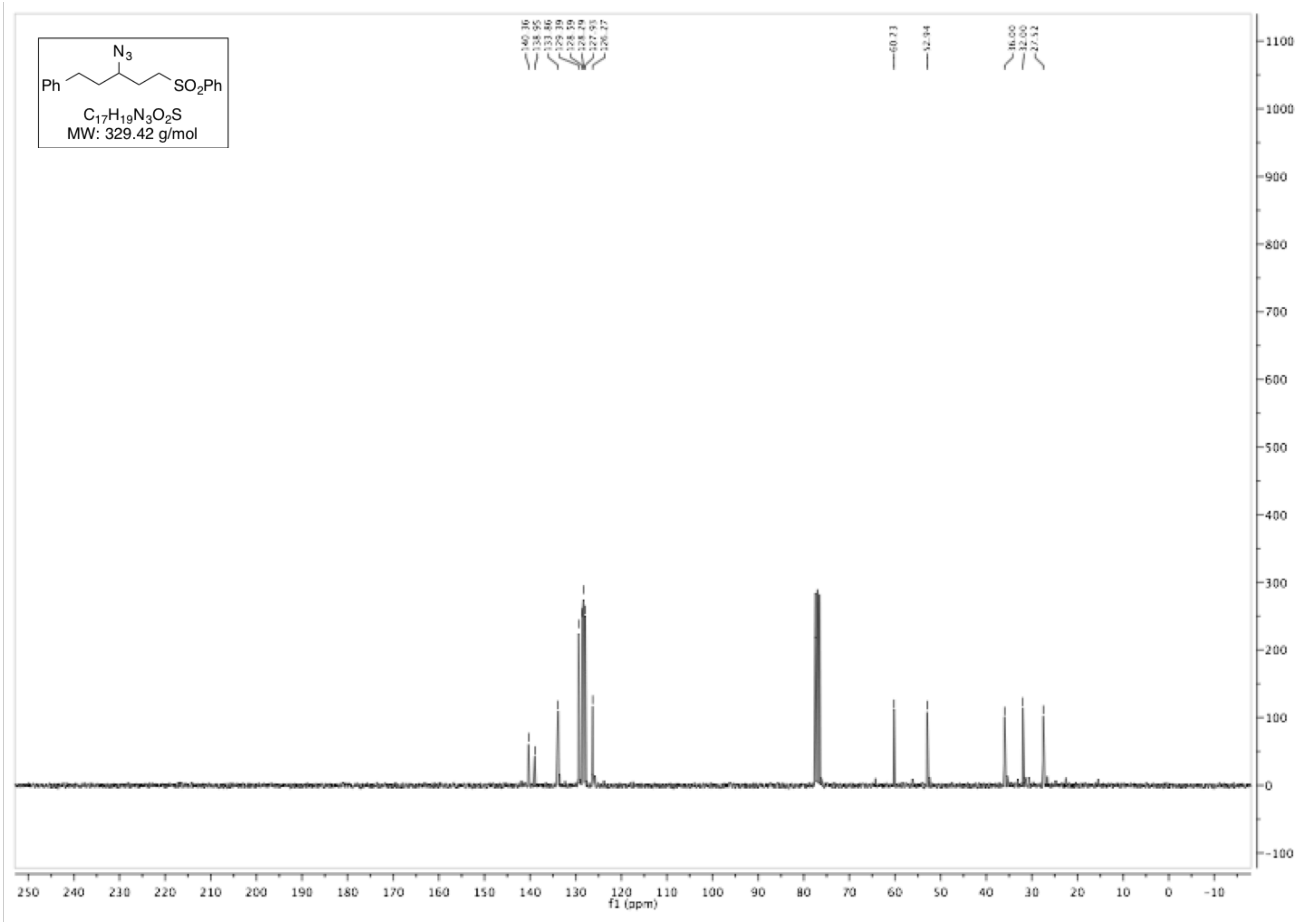
N3
Ph
SO2Ph
C17H19N3O2S
MW: 329.42 g/mol
140.36
138.95
133.86
129.39
128.59
128.29
127.93
126.27
60.23
52.94
36.00
32.00
27.52
f1 (ppm)

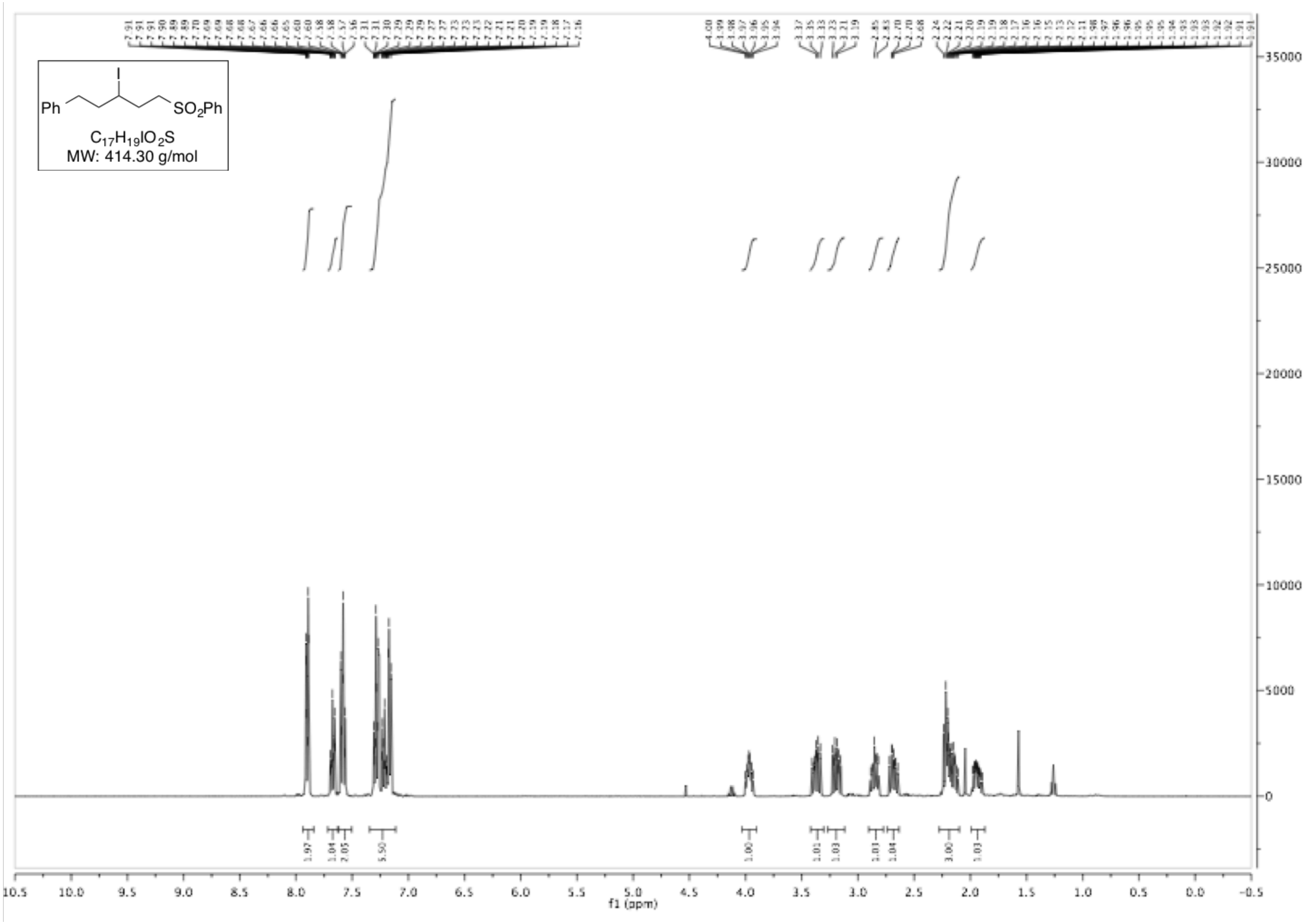

Ph
I
SO2Ph
C17H19IO2S
MW: 414.30 g/mol
f1 (ppm)

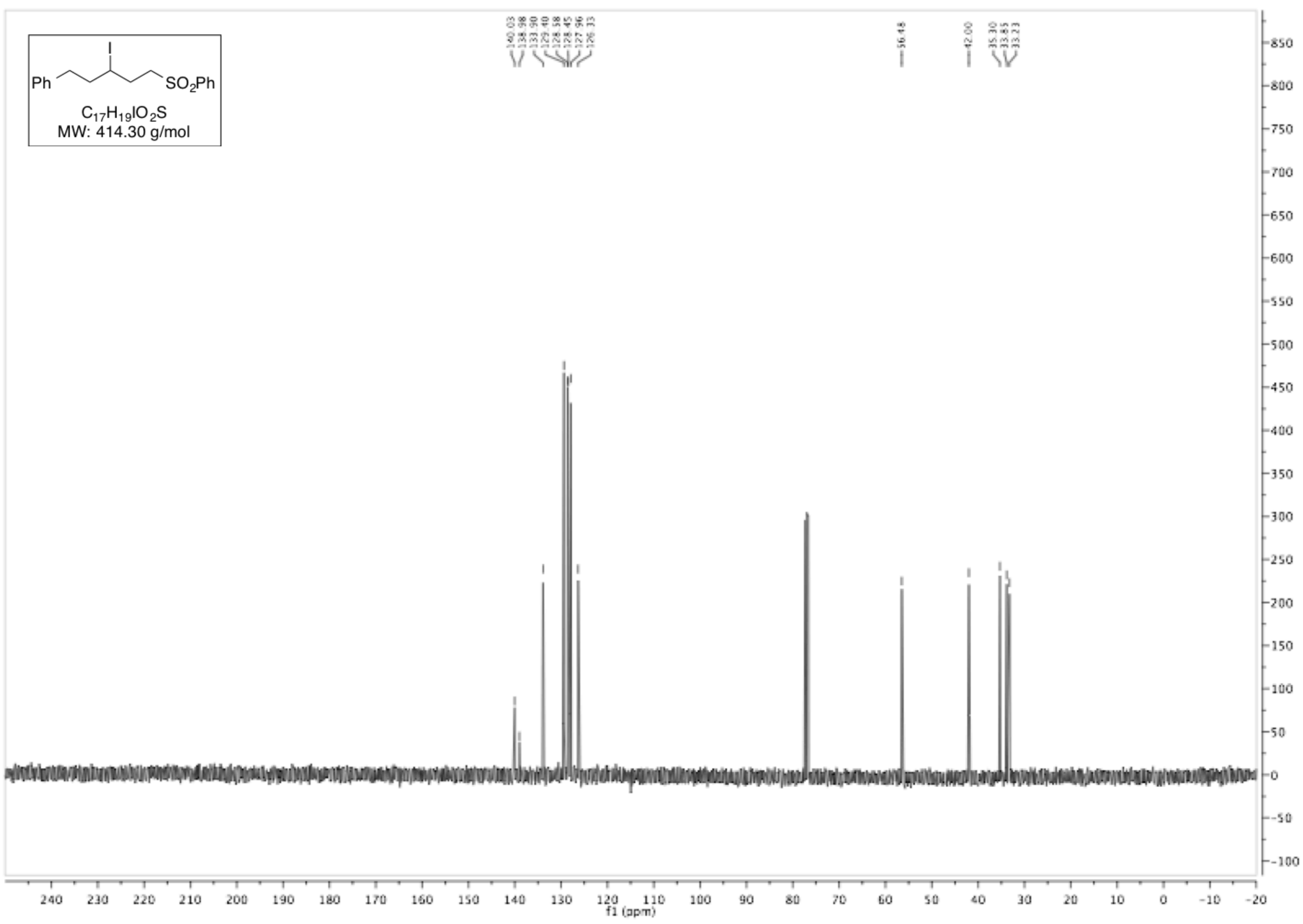
Ph
SO2Ph
I
C17H19IO2S
MW: 414.30 g/mol
140.03
138.98
133.90
129.40
128.58
128.45
127.96
126.33
56.48
42.00
35.30
33.85
33.23
f1 (ppm)

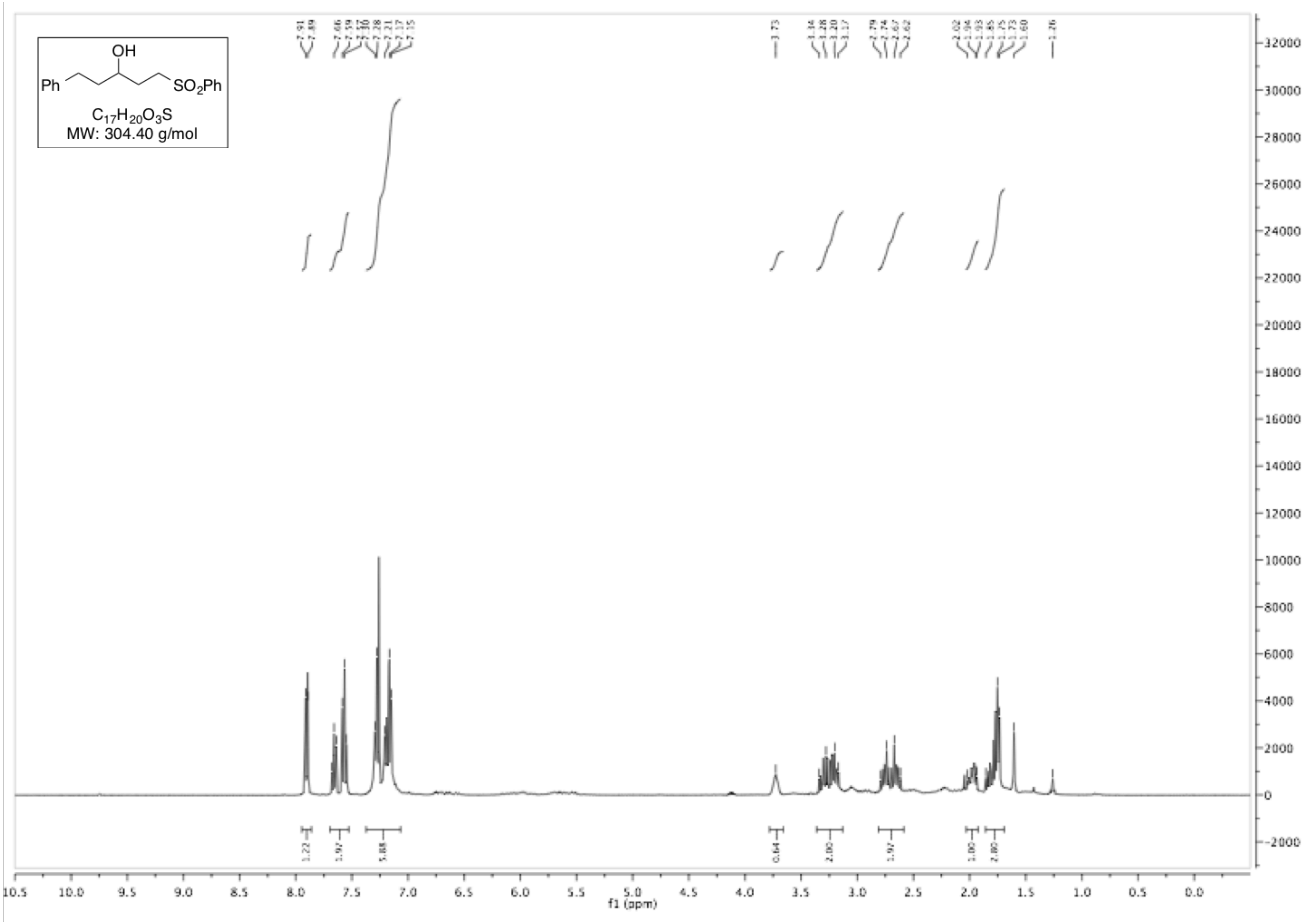

OH
Ph
SO2Ph
C17H20O3S
MW: 304.40 g/mol
7.91
7.89
7.66
7.59
7.57
7.30
7.28
7.21
7.17
7.15
3.73
3.34
3.28
3.20
3.17
2.79
2.74
2.67
2.62
2.02
1.94
1.93
1.85
1.75
1.73
1.60
1.26
1.22
1.97
5.88
0.64
2.00
1.97
1.00
2.80
f1 (ppm)

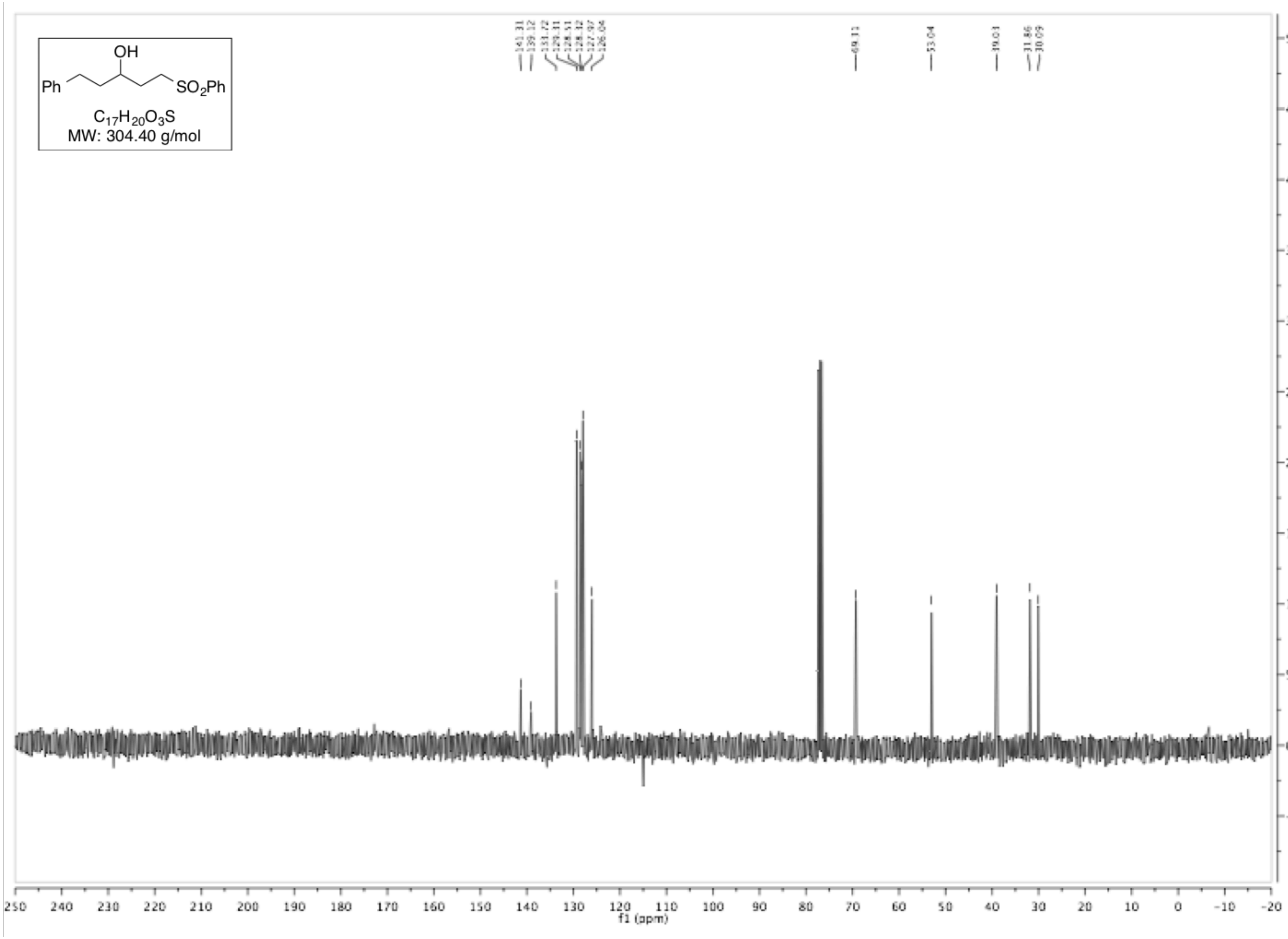

Ph
OH
SO2Ph
C17H20O3S
MW: 304.40 g/mol
141.31
139.12
133.72
129.31
128.51
128.32
127.97
126.04
69.11
53.04
39.03
31.86
30.09
f1 (ppm)

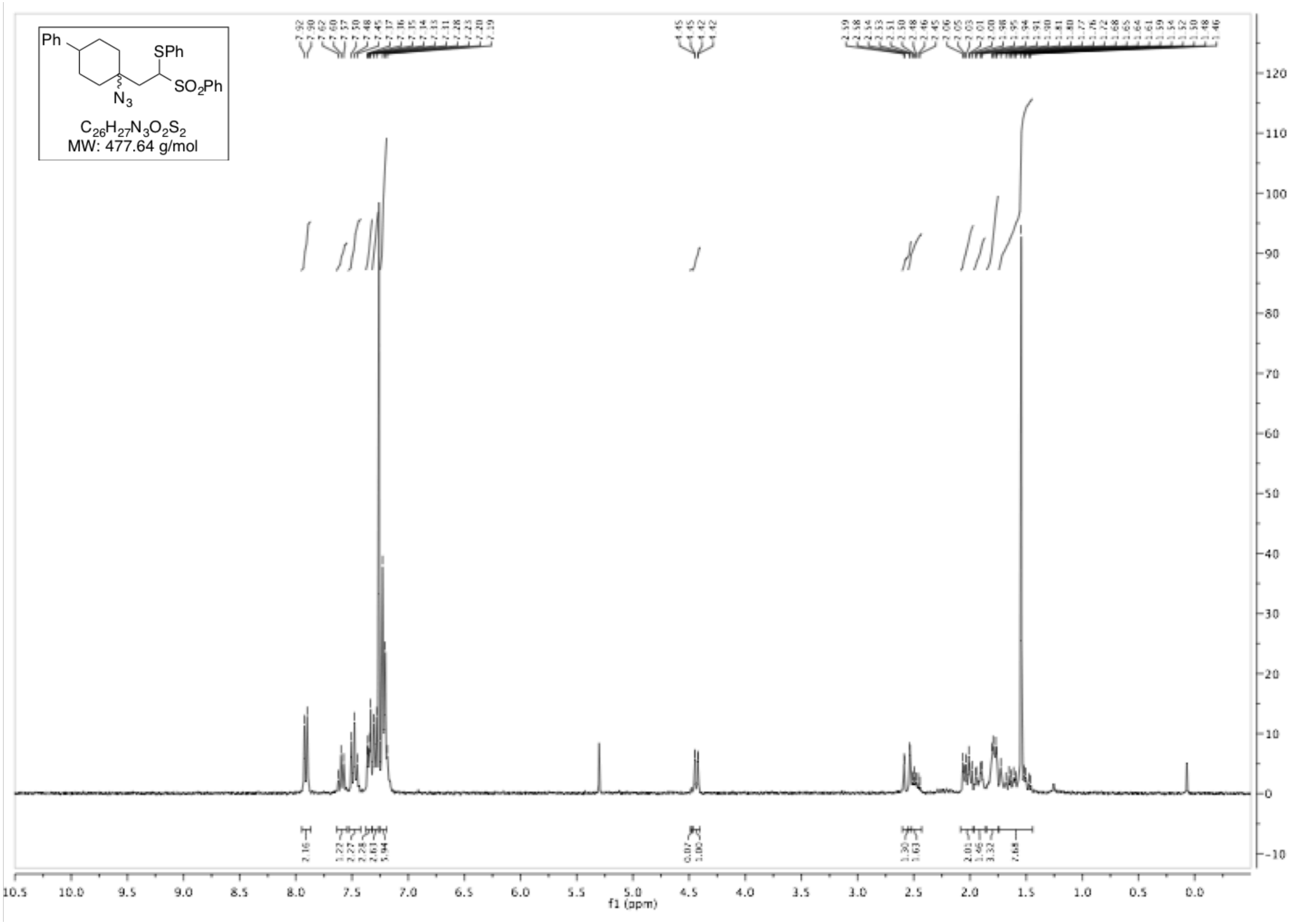

Ph
SPh
SO2Ph
N3
C26H27N3O2S2
MW: 477.64 g/mol
f1 (ppm)

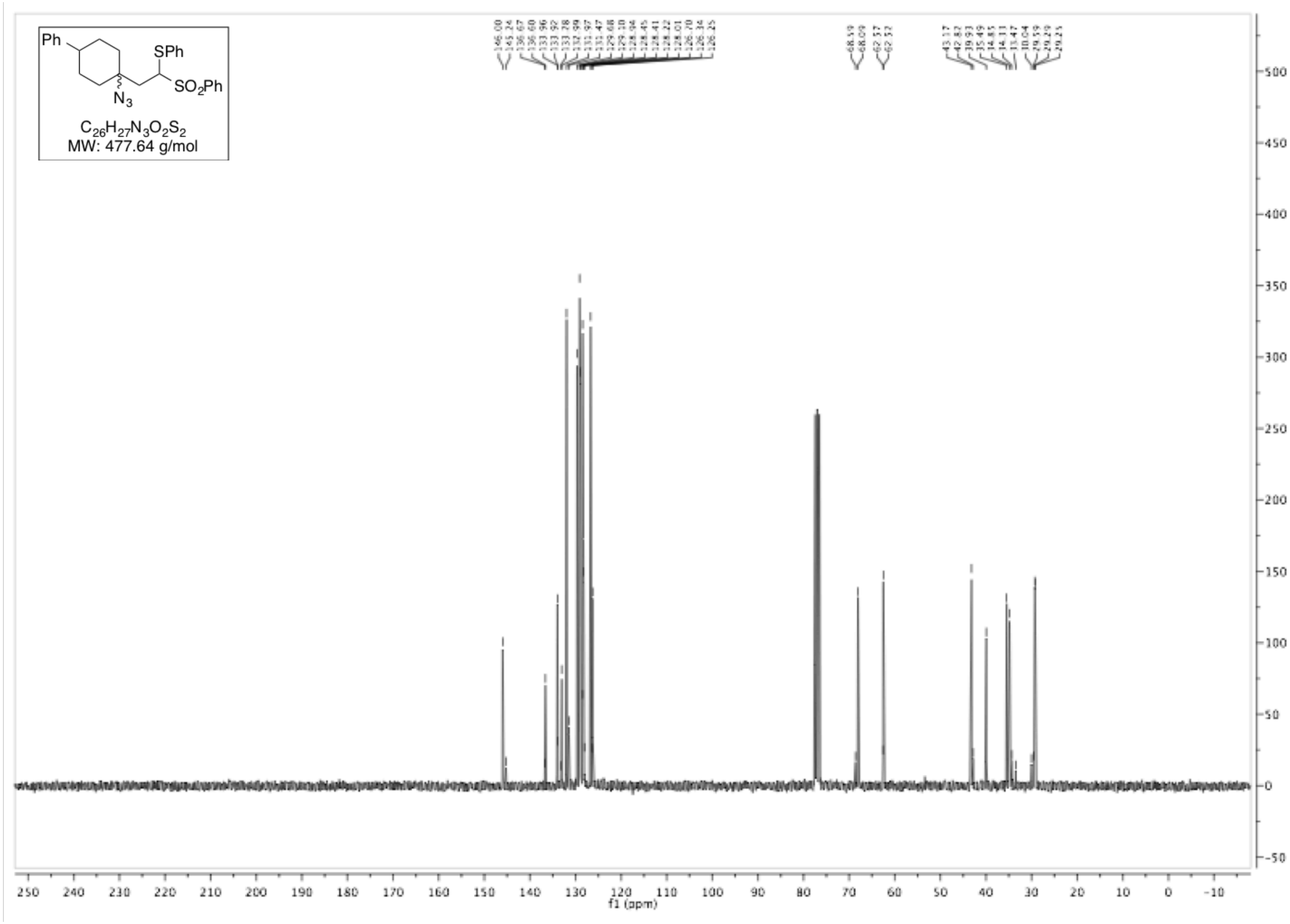
Ph
SPh
SO2Ph
N3
C26H27N3O2S2
MW: 477.64 g/mol
146.00
145.24
136.67
136.60
133.96
133.92
133.28
132.99
131.97
131.47
129.68
129.10
128.94
128.45
128.41
128.22
128.01
126.70
126.34
126.25
68.59
68.09
62.57
62.52
43.17
42.82
39.93
35.49
34.85
34.11
33.47
30.04
29.59
29.29
29.25
f1 (ppm)

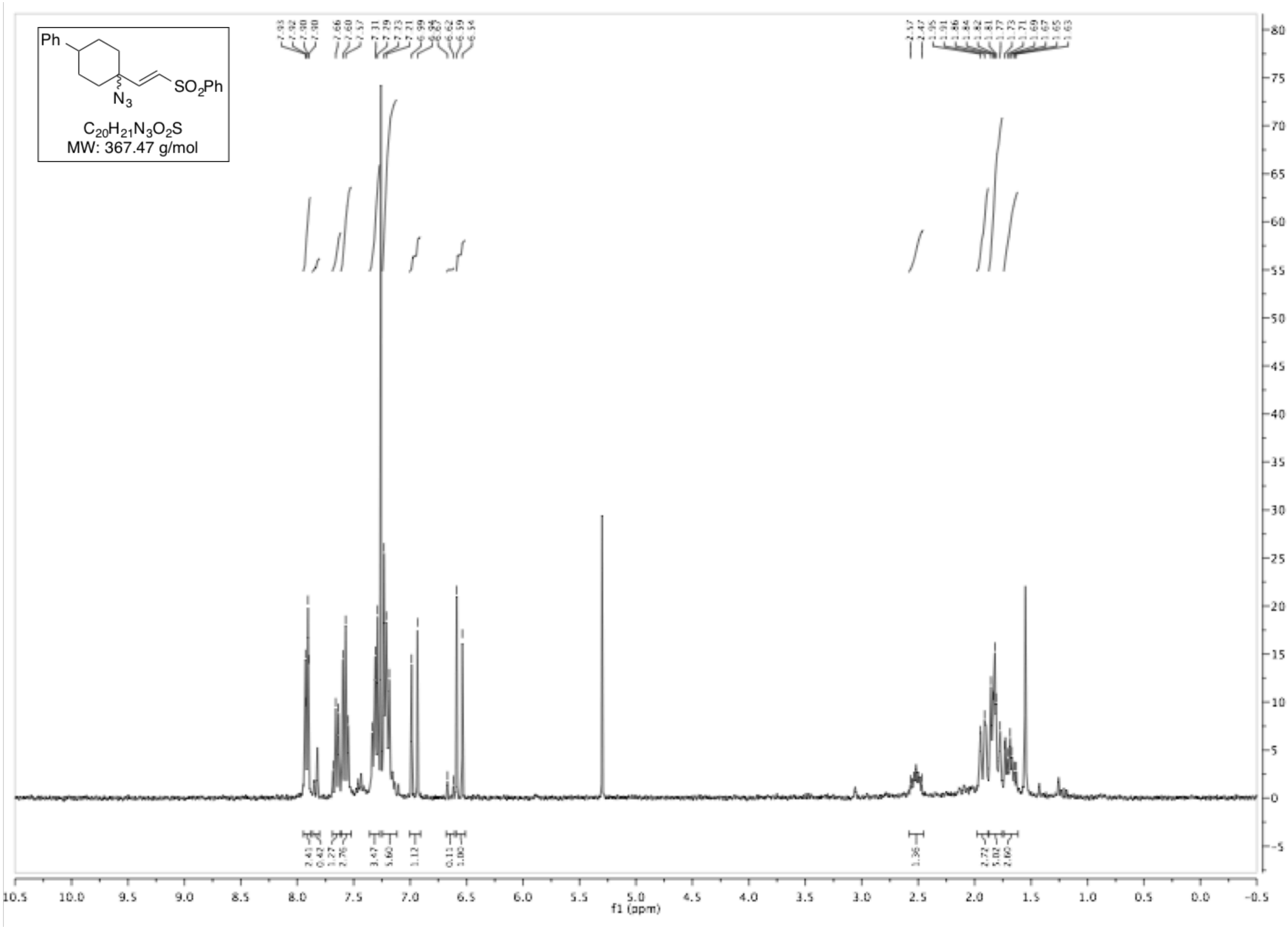
Ph
SO_2Ph
N_3
$C_{20}H_{21}N_3O_2S$
MW: 367.47 g/mol
f1 (ppm)

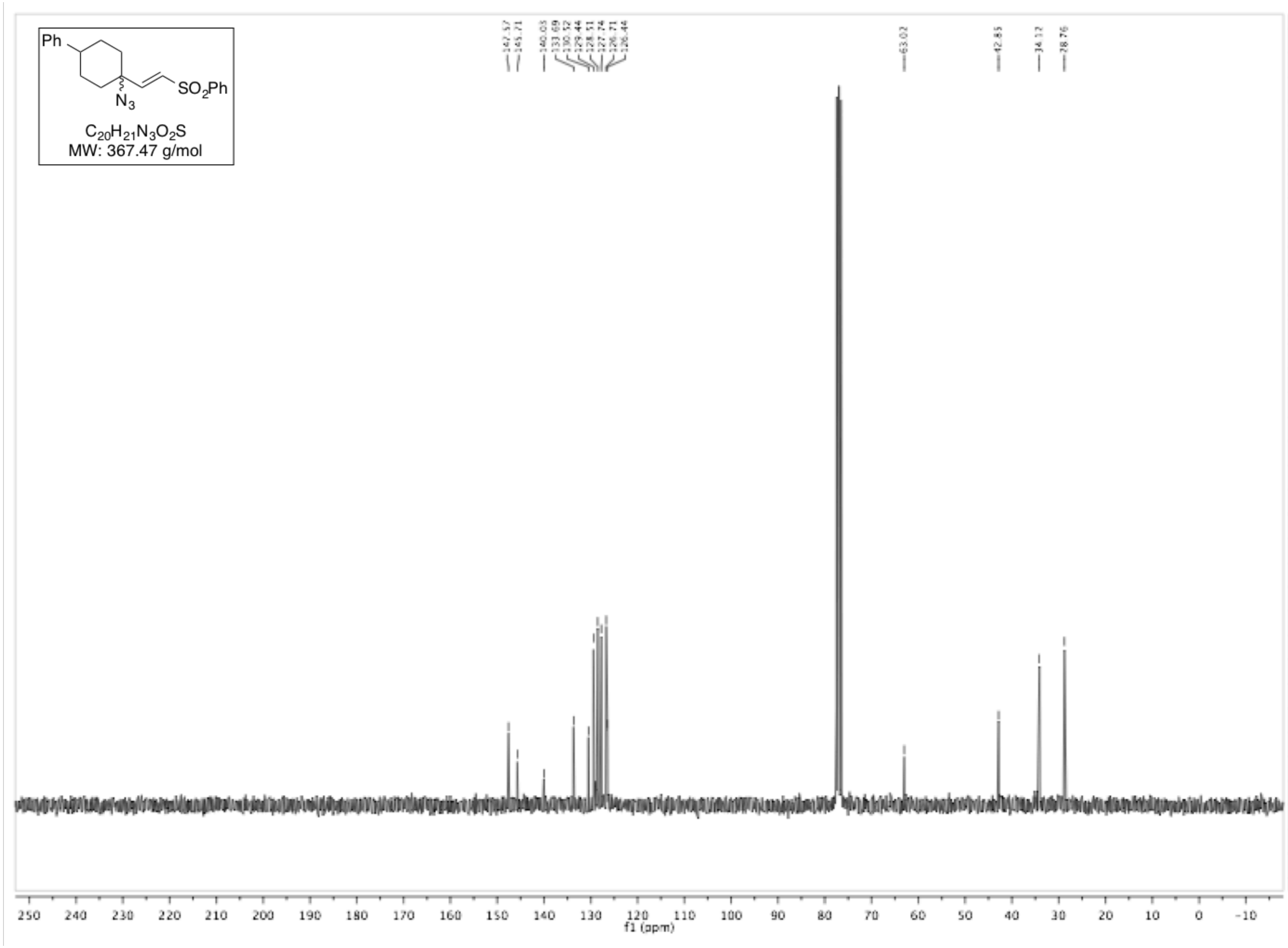
Ph
N3
SO2Ph
C20H21N3O2S
MW: 367.47 g/mol
147.57
145.71
140.03
133.69
130.52
129.44
128.51
127.74
126.71
126.44
63.02
42.85
34.12
28.76
f1 (ppm)

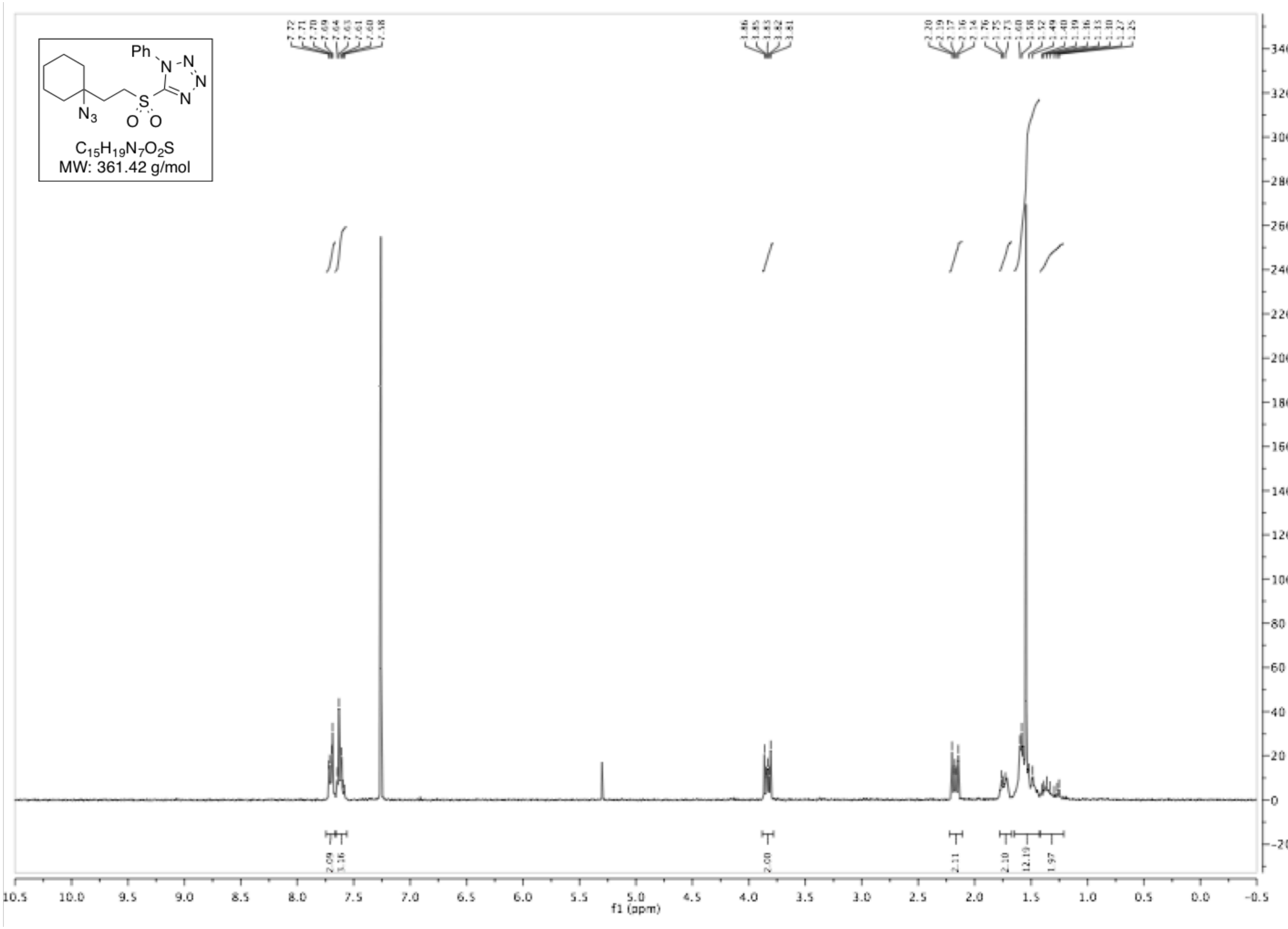
Ph
N
N
N
N
S
O O
N3
C15H19N7O2S
MW: 361.42 g/mol
f1 (ppm)

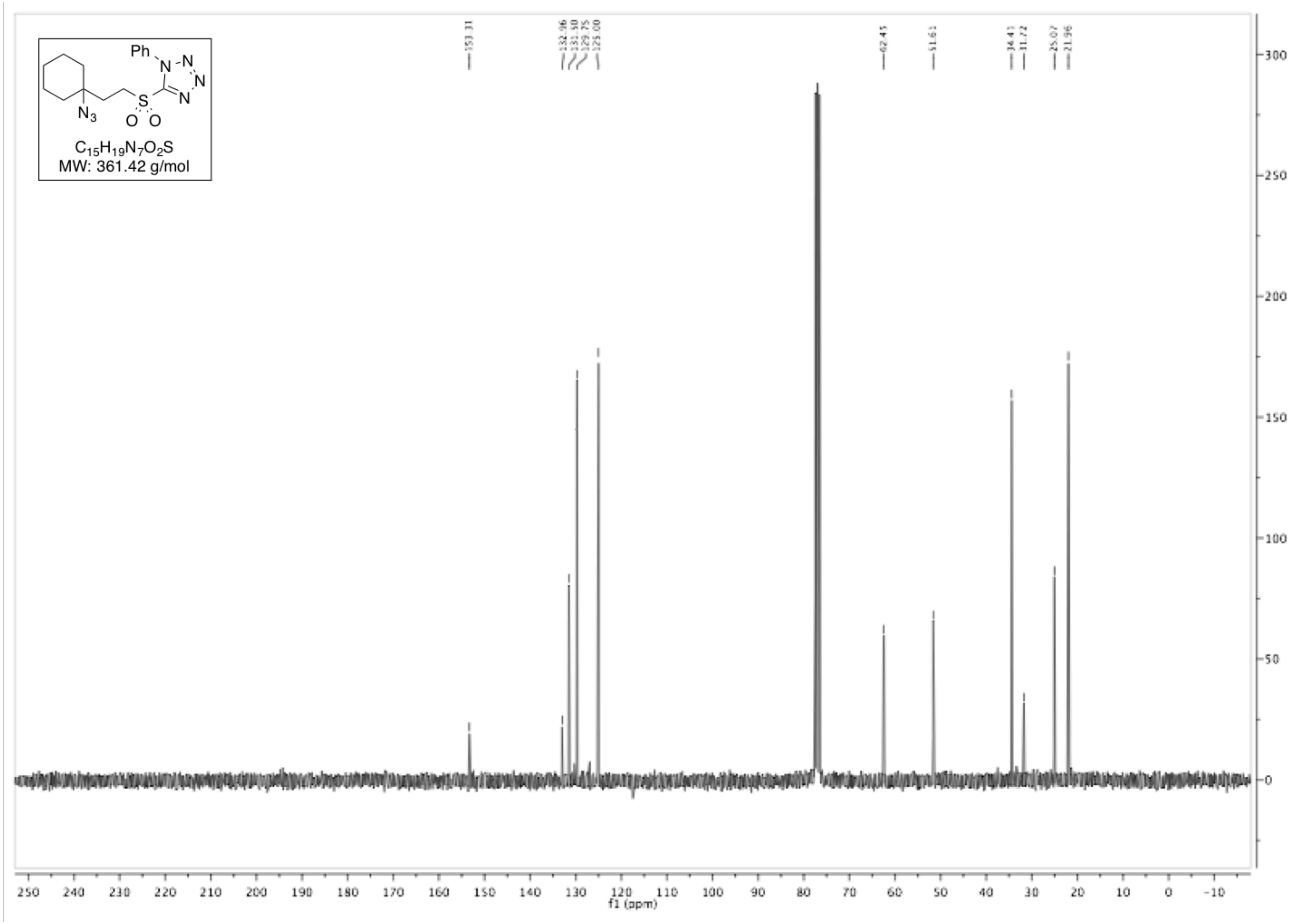
Ph
N
N
N
N
S
O
O
N3
C15H19N7O2S
MW: 361.42 g/mol
153.31
132.96
131.50
129.75
125.00
62.45
51.61
34.41
31.72
25.07
21.96
f1 (ppm)

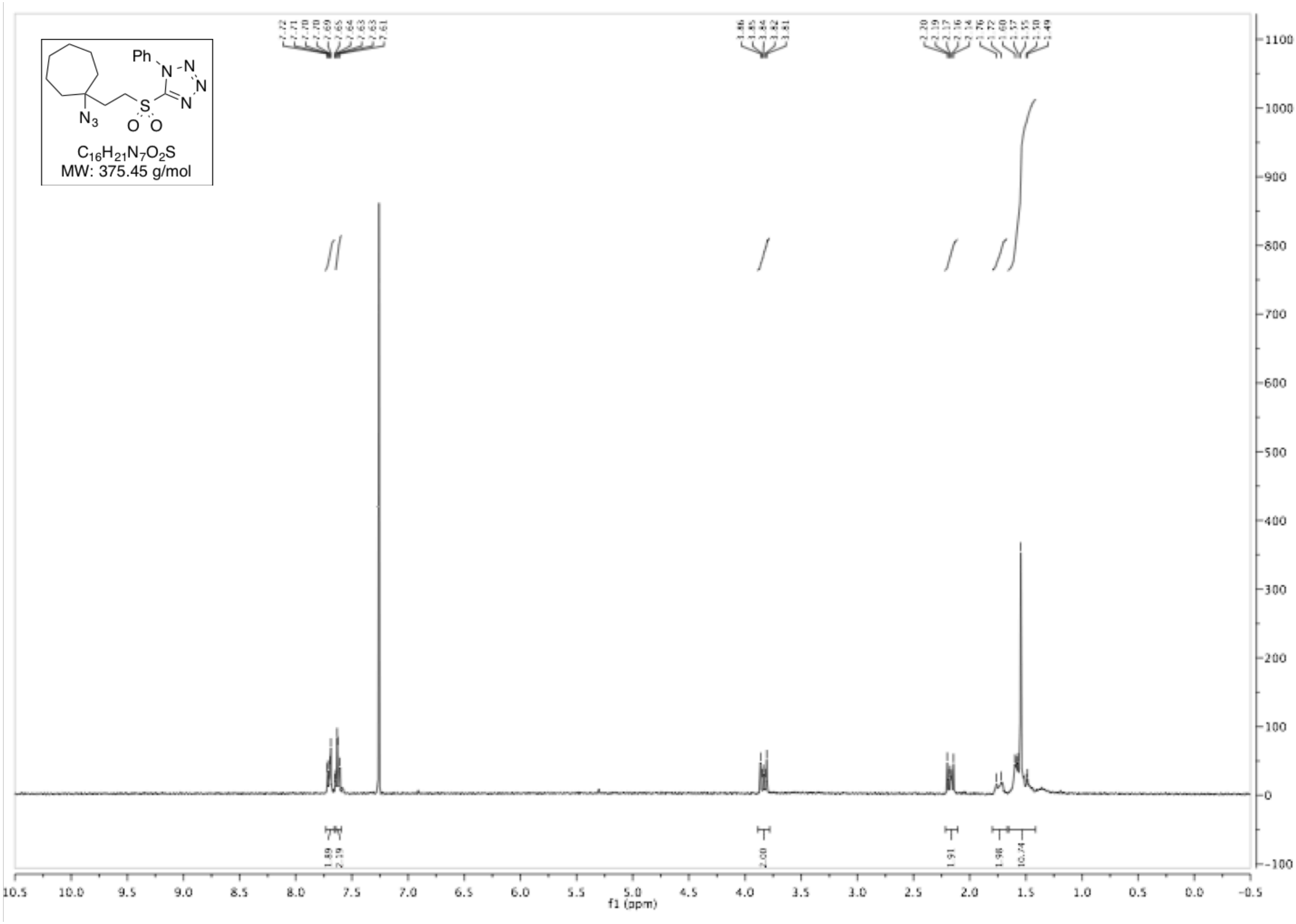
Ph
N
N
N
N
N3
S
O O
C16H21N7O2S
MW: 375.45 g/mol
7.72
7.71
7.70
7.70
7.69
7.65
7.64
7.63
7.63
7.61
3.86
3.85
3.84
3.82
3.81
2.20
2.19
2.17
2.16
2.14
1.76
1.72
1.60
1.57
1.55
1.50
1.49
1.89
2.19
2.00
1.91
1.98
10.74
f1 (ppm)

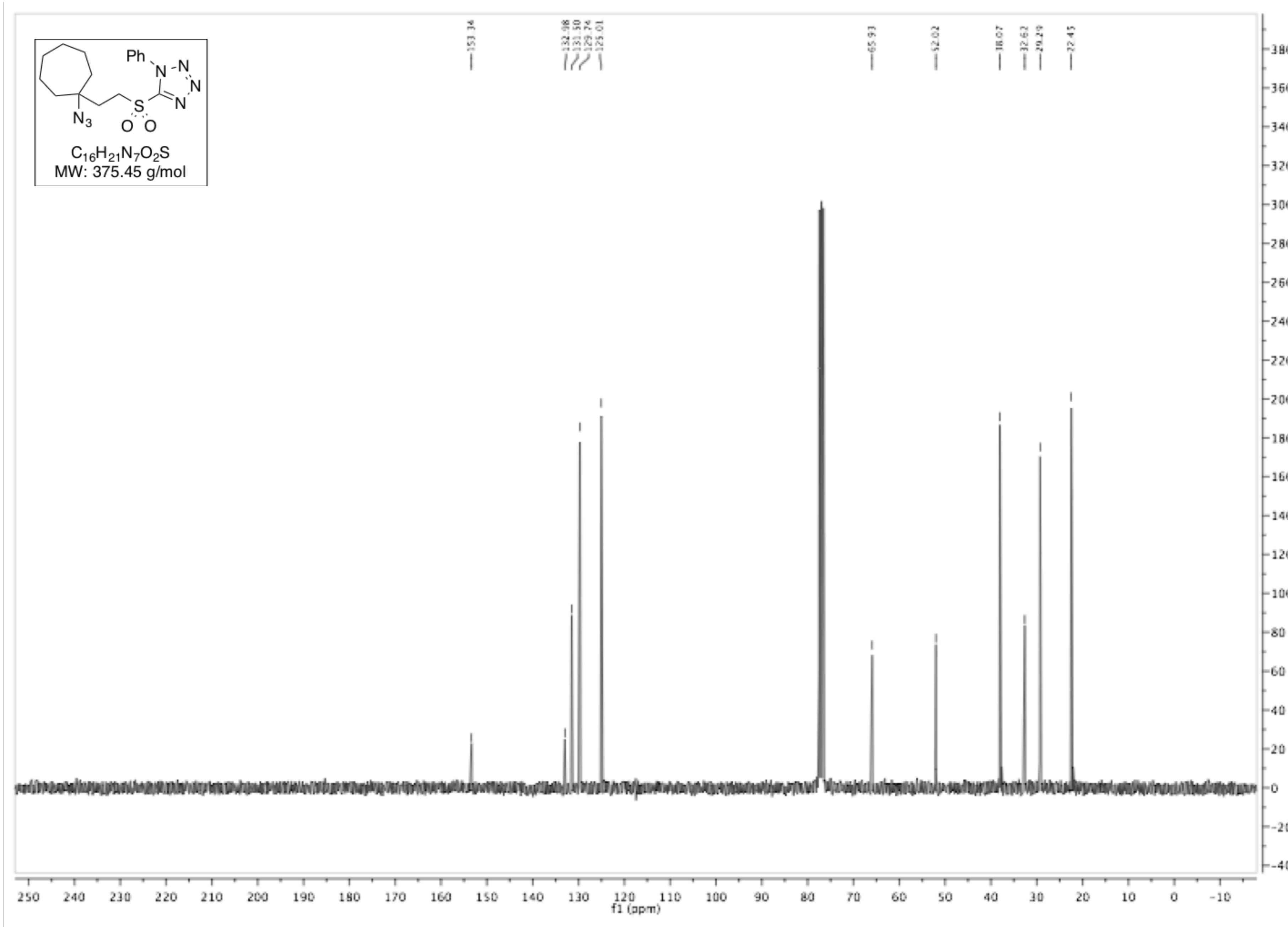

Ph
N
N
N
N
S
O
O
N3
C16H21N7O2S
MW: 375.45 g/mol
153.34
132.98
131.50
129.74
125.01
65.93
52.02
38.07
32.62
29.29
22.45
f1 (ppm)

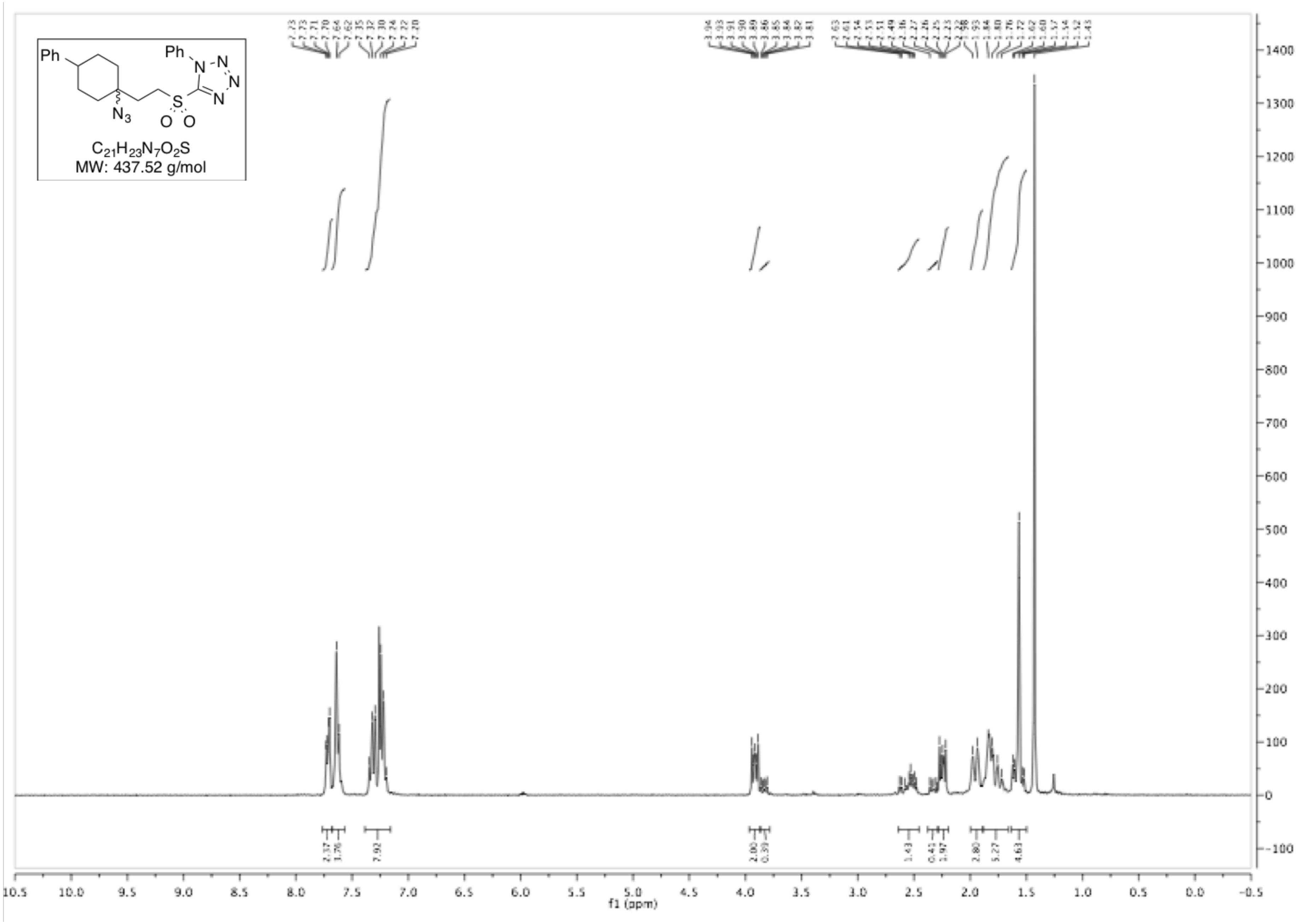
Ph
Ph
N
N
N
N
N3
S
O O
C21H23N7O2S
MW: 437.52 g/mol
2.37
1.76
7.92
2.00
0.39
1.43
0.41
1.97
2.80
5.27
4.63
f1 (ppm)

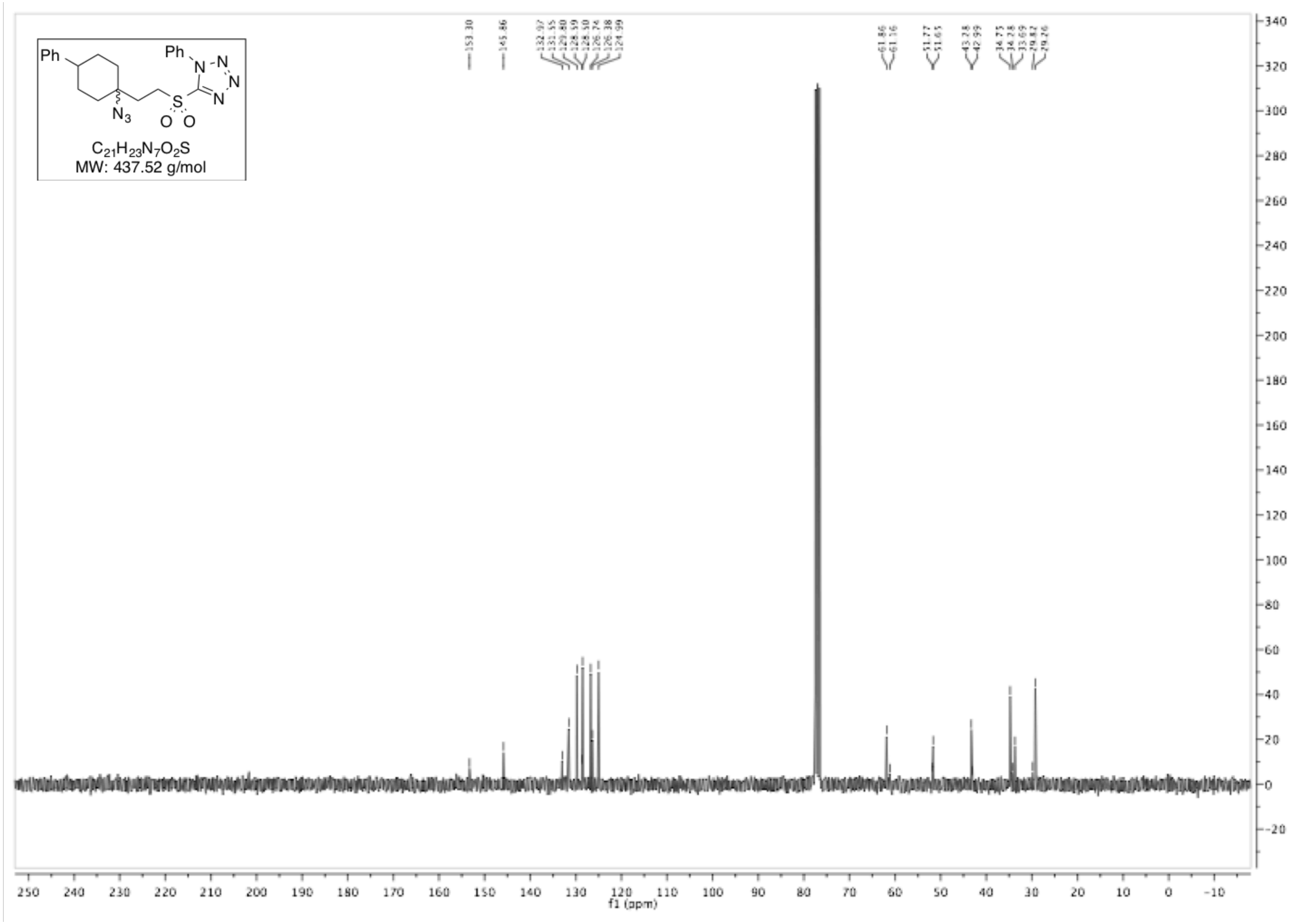
Ph
Ph
N
N
N
N
S
O O
N3
C21H23N7O2S
MW: 437.52 g/mol
153.30
145.86
132.97
131.55
129.80
128.59
128.50
126.74
126.38
124.99
61.86
61.16
51.77
51.65
43.28
42.99
34.75
34.28
33.69
29.82
29.26
f1 (ppm)

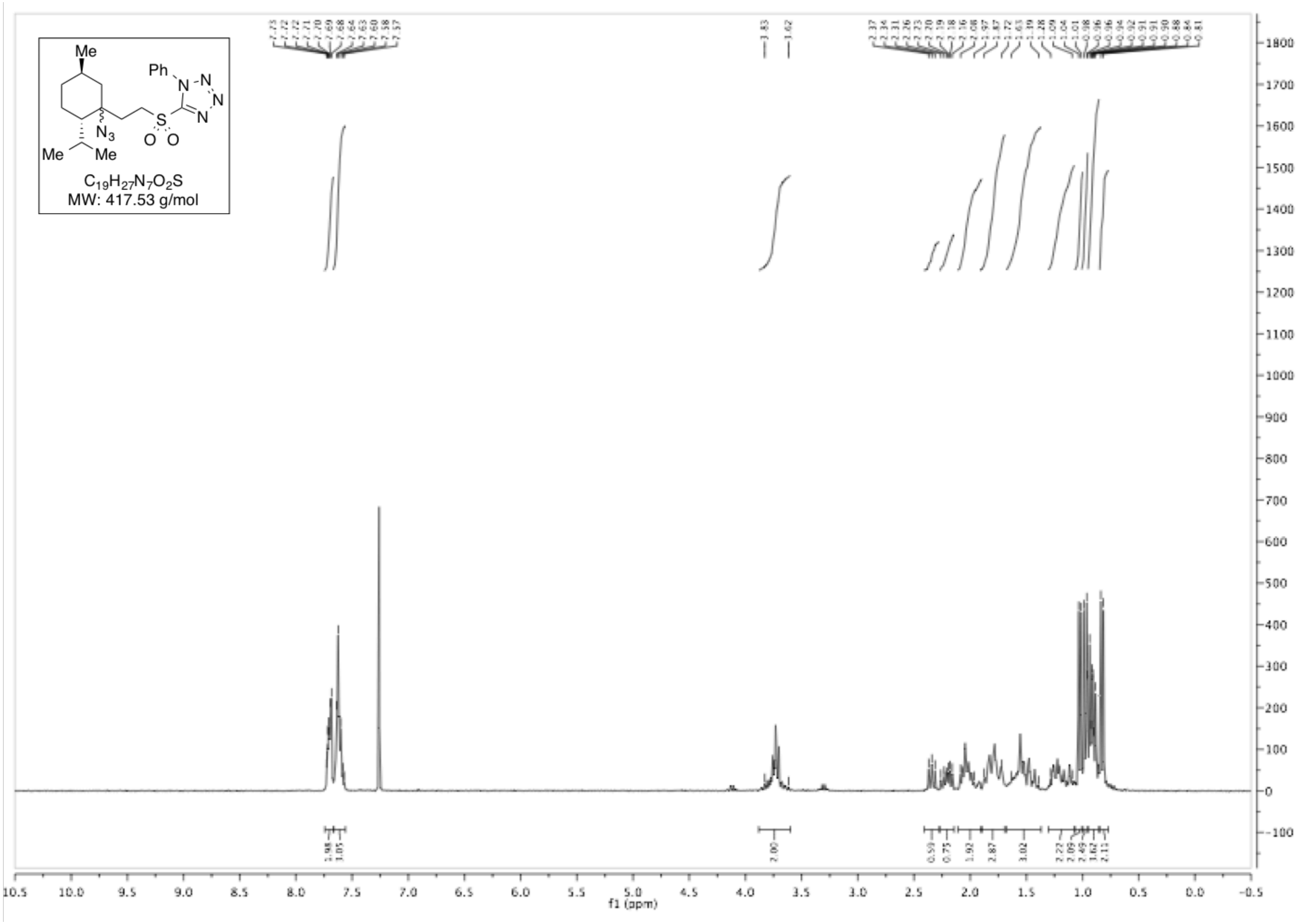
Me
Ph
N
N
N
N
S
O
O
N3
Me
Me
C19H27N7O2S
MW: 417.53 g/mol
f1 (ppm)

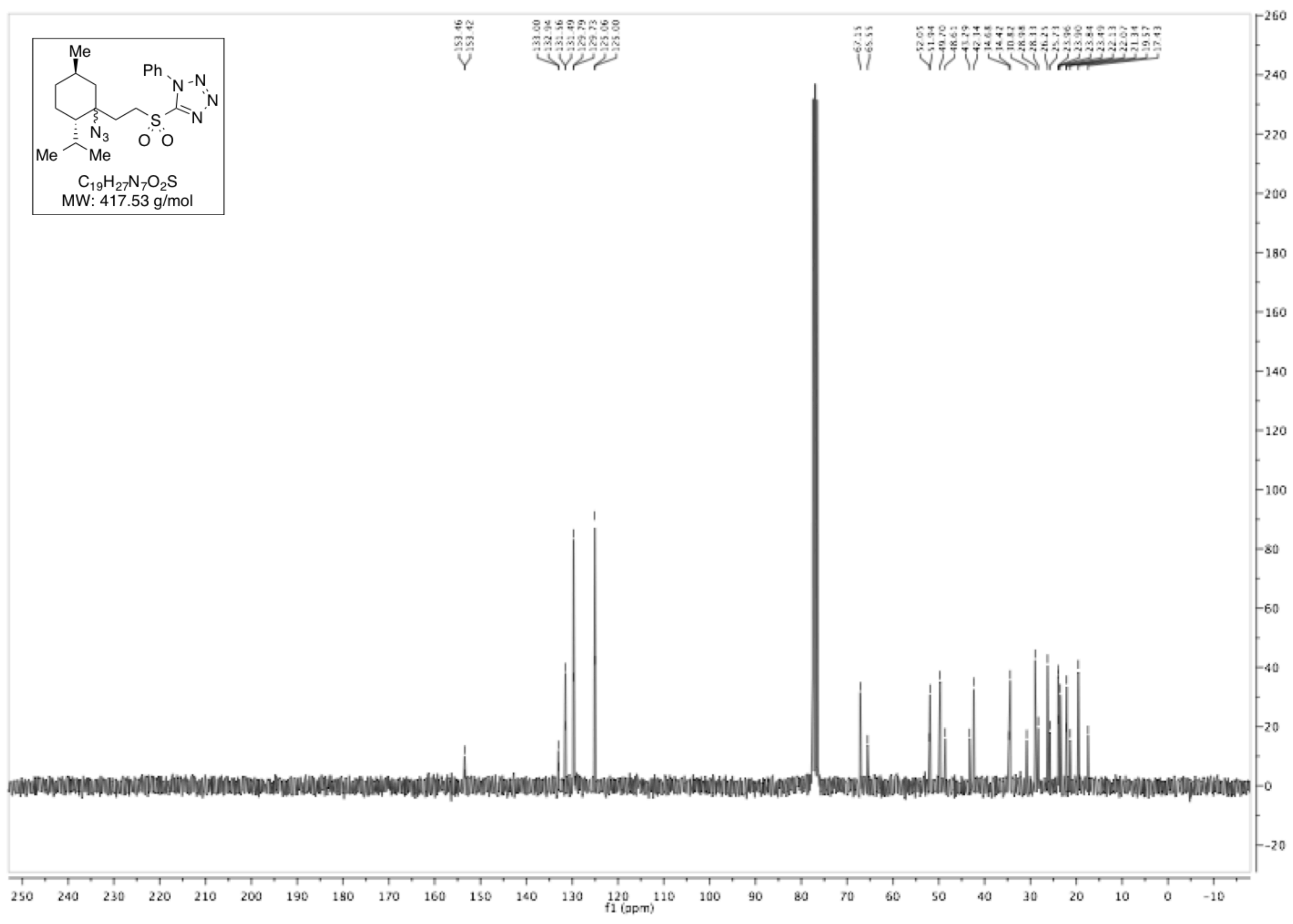

Me
Ph
N
N
N
N
S
O O
N$_3$
Me Me
$C_{19}H_{27}N_7O_2S$
MW: 417.53 g/mol
f1 (ppm)

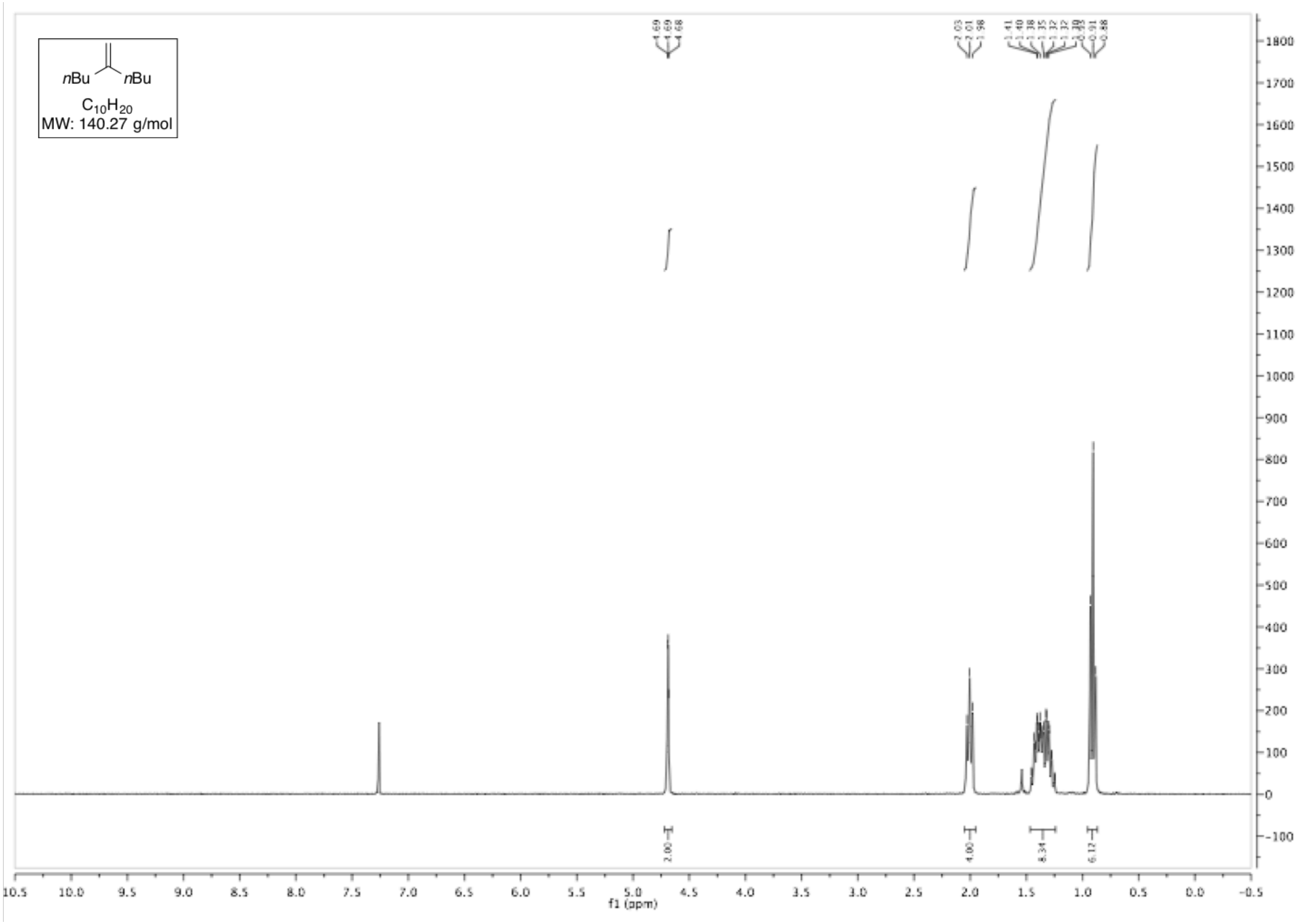
nBu
nBu
$C_{10}H_{20}$
MW: 140.27 g/mol
4.69
4.69
4.68
2.03
2.01
1.98
1.41
1.40
1.38
1.35
1.32
1.32
0.91
0.88
2.00
4.00
8.34
6.12
f1 (ppm)

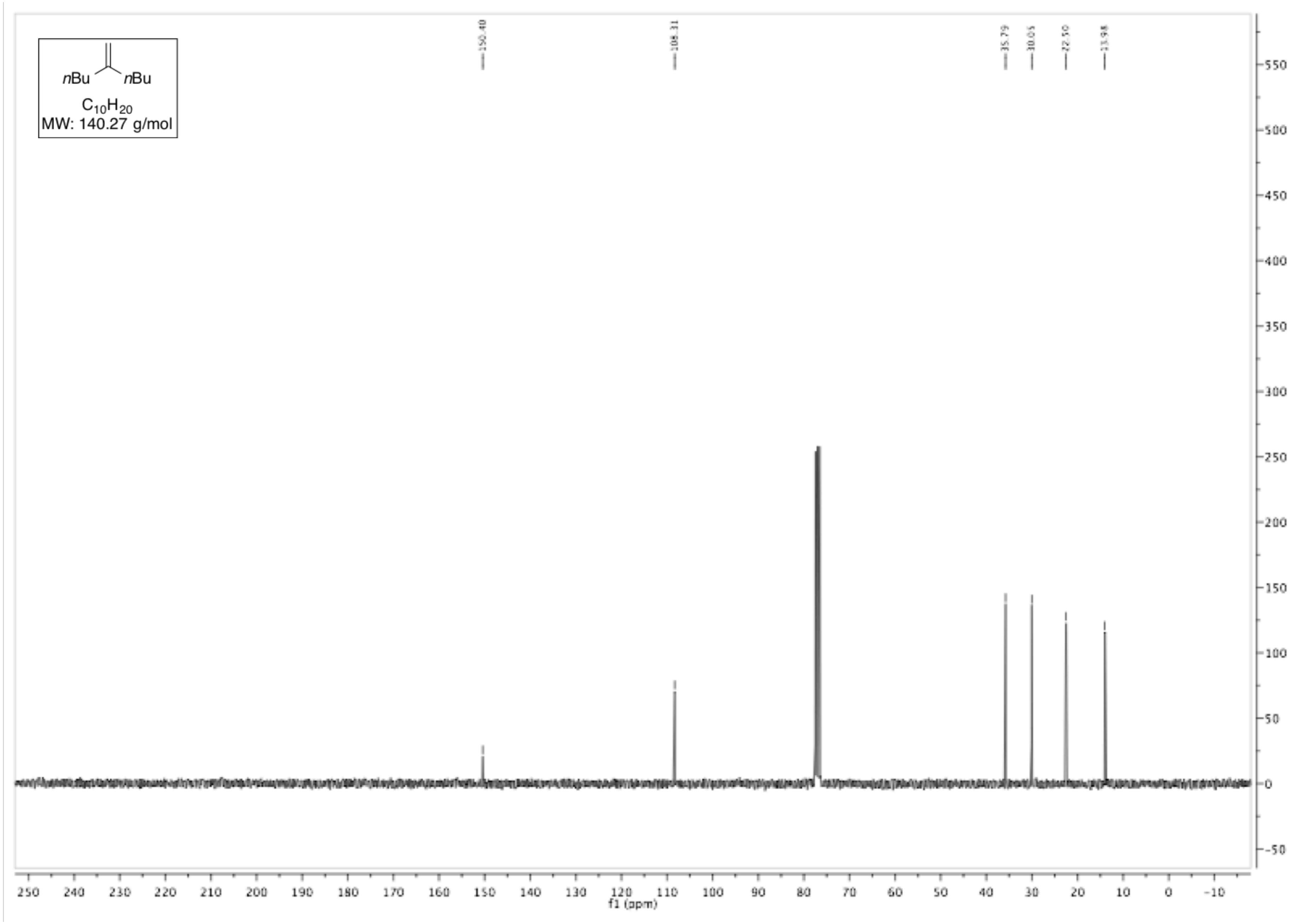
nBu
nBu
$C_{10}H_{20}$
MW: 140.27 g/mol
150.40
108.31
35.79
30.05
22.50
13.98
f1 (ppm)

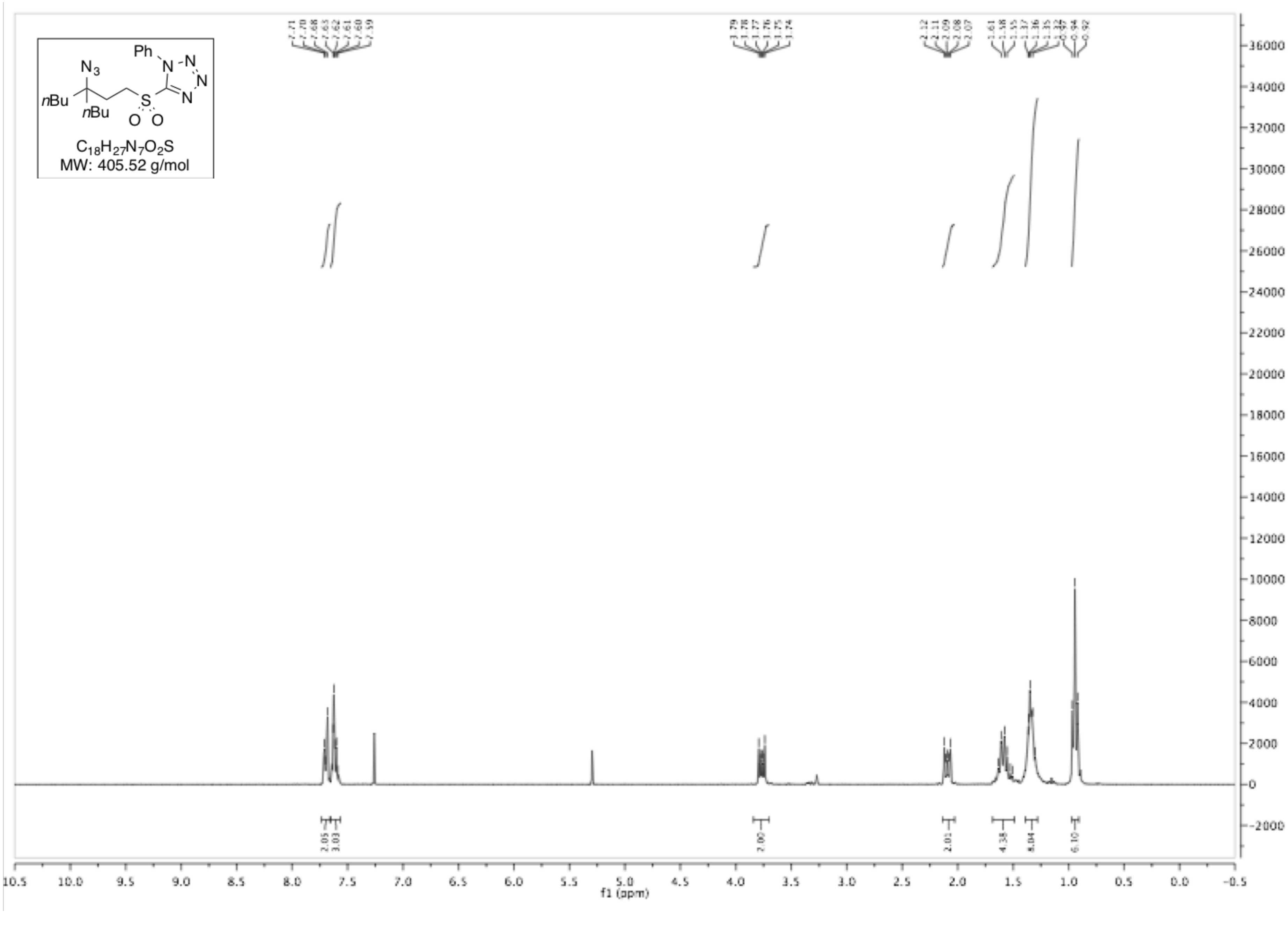
Ph
N3
nBu
nBu
S
O O
N
C18H27N7O2S
MW: 405.52 g/mol
7.71
7.70
7.68
7.63
7.62
7.61
7.60
7.59
3.79
3.78
3.77
3.76
3.75
3.74
2.12
2.11
2.09
2.08
2.07
1.61
1.58
1.55
1.37
1.36
1.35
0.94
0.92
2.05
3.03
2.00
2.01
4.38
8.04
6.10
f1 (ppm)
10.5
10.0
9.5
9.0
8.5
8.0
7.5
7.0
6.5
6.0
5.5
5.0
4.5
4.0
3.5
3.0
2.5
2.0
1.5
1.0
0.5
0.0
-0.5
36000
34000
32000
30000
28000
26000
24000
22000
20000
18000
16000
14000
12000
10000
8000
6000
4000
2000
0
-2000

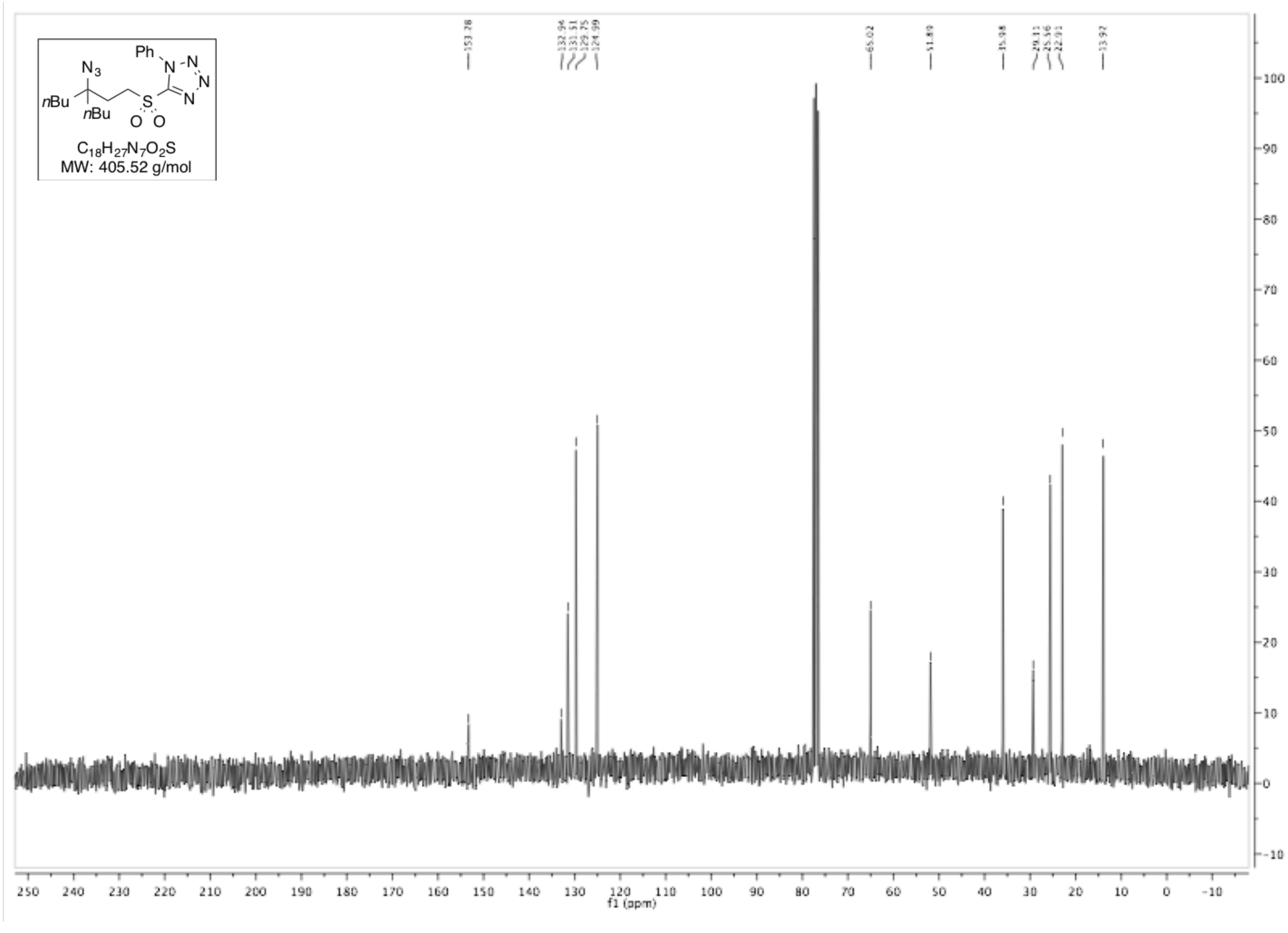

Ph
N
N
N
N
N3
nBu
nBu
S
O O
C18H27N7O2S
MW: 405.52 g/mol
153.28
132.94
131.51
129.75
124.99
65.02
51.89
35.98
29.11
25.56
22.91
13.92
f1 (ppm)

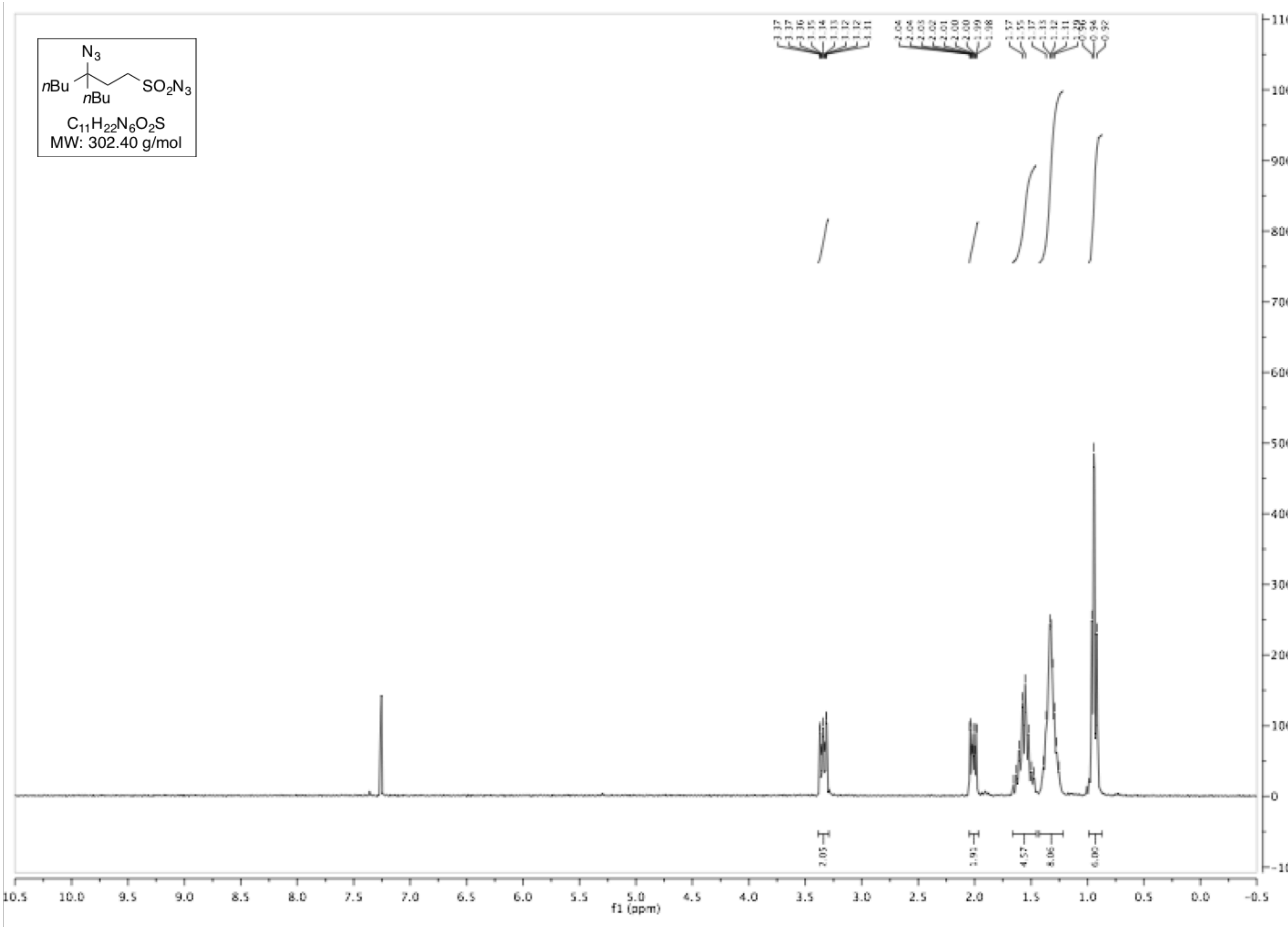
N3
nBu
nBu
SO2N3
C11H22N6O2S
MW: 302.40 g/mol
3.37 3.37 3.36 3.35 3.34 3.33 3.32 3.32 3.31
2.04 2.04 2.03 2.02 2.01 2.00 2.00 1.99 1.98
1.57 1.55 1.37 1.33 1.32 1.31 0.96 0.94 0.92
2.05
1.93
4.57
8.06
6.00
f1 (ppm)

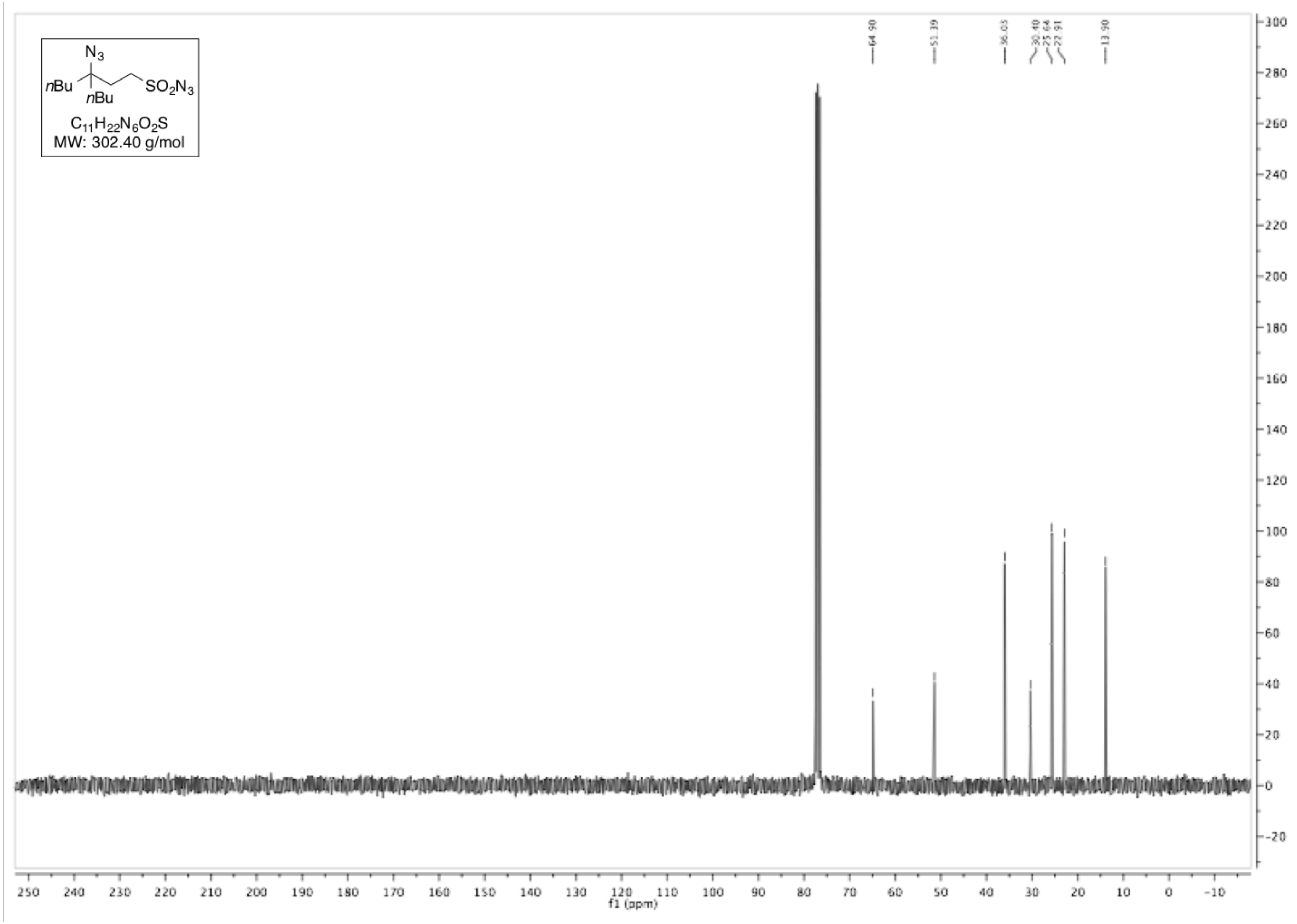
N3
nBu
nBu
SO2N3
C11H22N6O2S
MW: 302.40 g/mol
64.90
51.39
36.03
30.40
25.64
22.91
13.90
f1 (ppm)

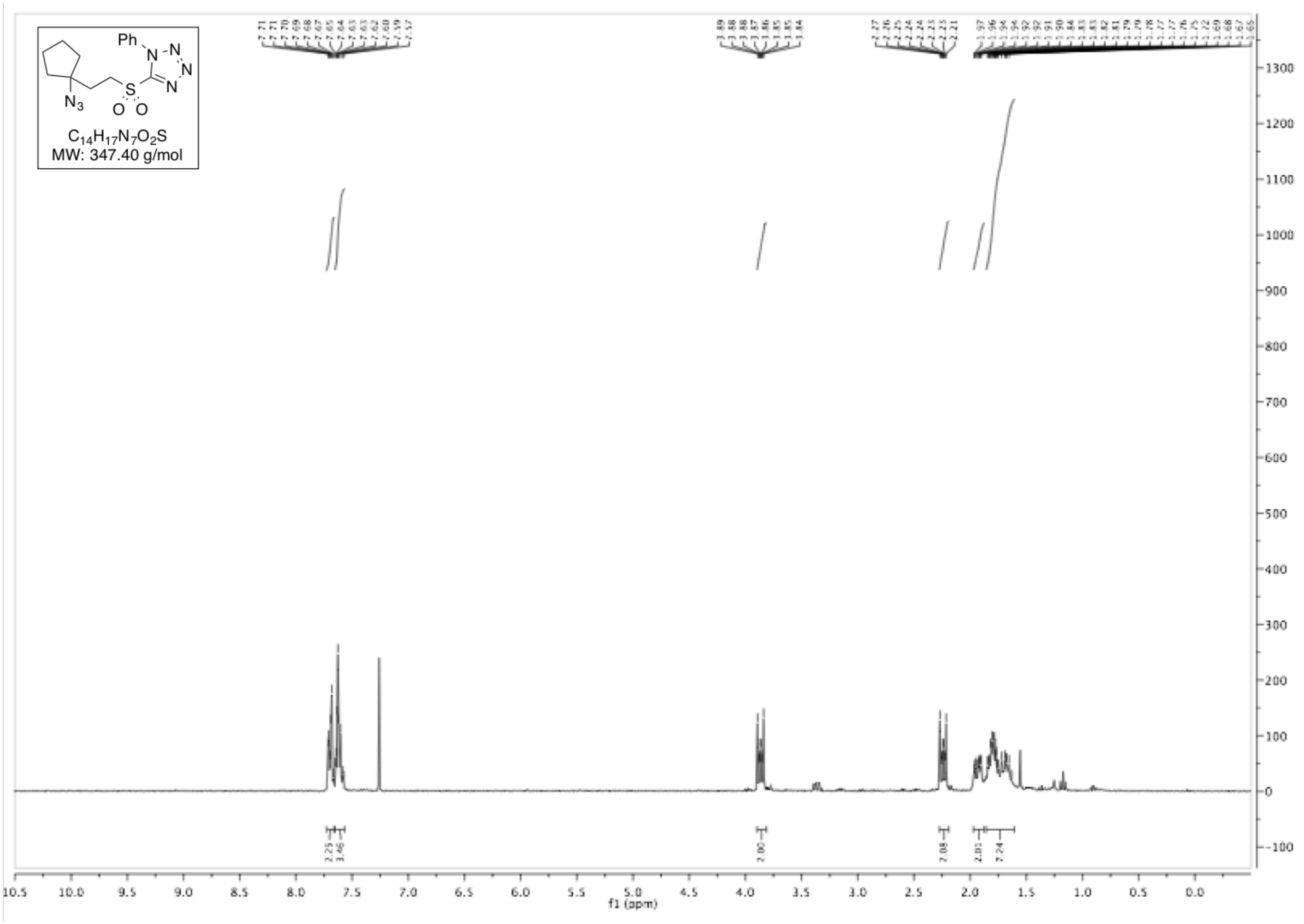

Ph
N
N
N
N
S
O O
N3
C14H17N7O2S
MW: 347.40 g/mol
f1 (ppm)

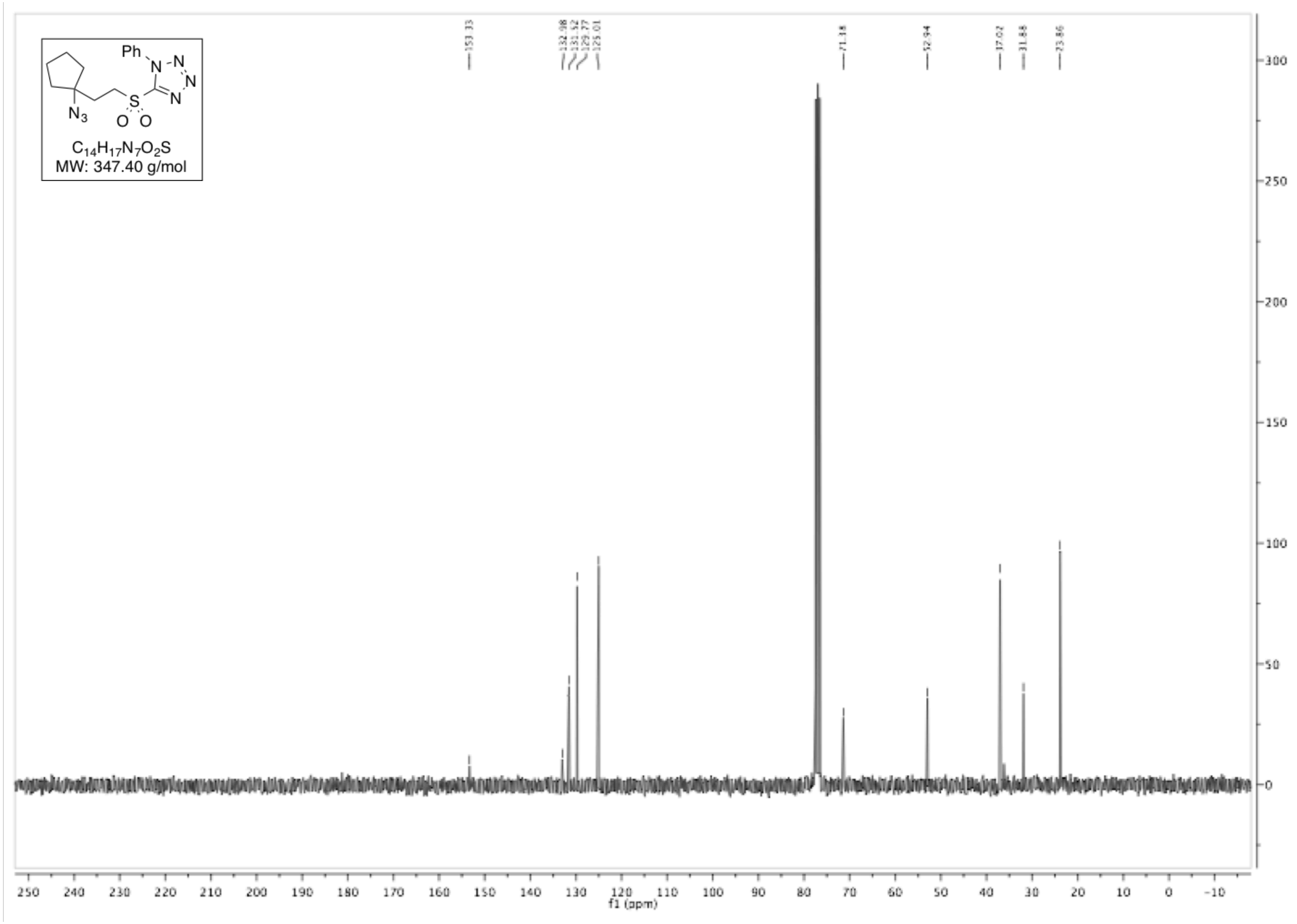

Ph
N
N
N
N
S
O O
N3
C14H17N7O2S
MW: 347.40 g/mol
153.33
132.98
131.52
129.77
125.01
71.38
52.94
37.02
31.88
23.86
f1 (ppm)

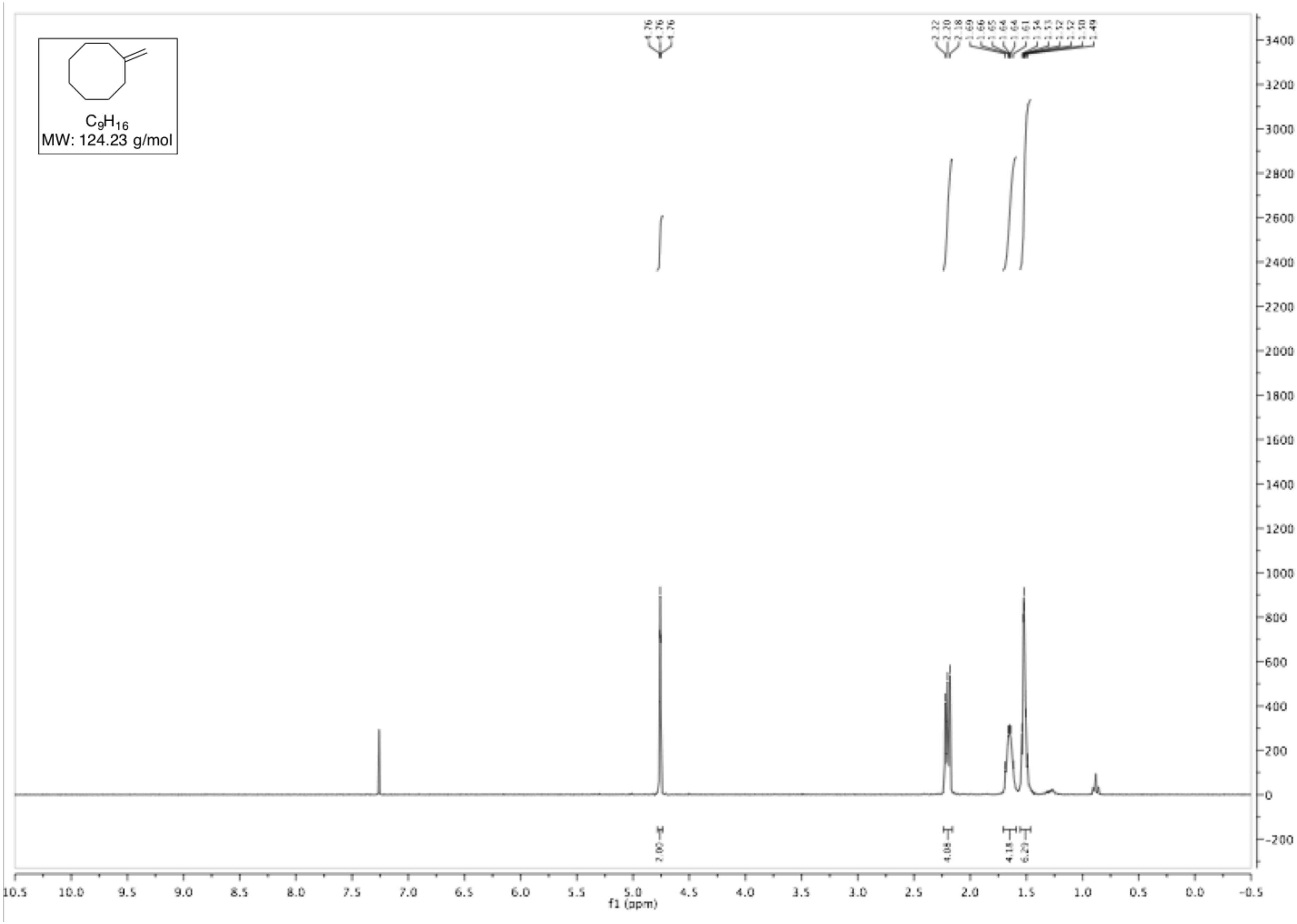

C$_9$H$_{16}$
MW: 124.23 g/mol
4.76
4.76
4.76
2.22
2.20
2.18
1.69
1.66
1.65
1.64
1.64
1.61
1.54
1.53
1.52
1.52
1.50
1.49
2.00
4.08
4.18
6.29
f1 (ppm)

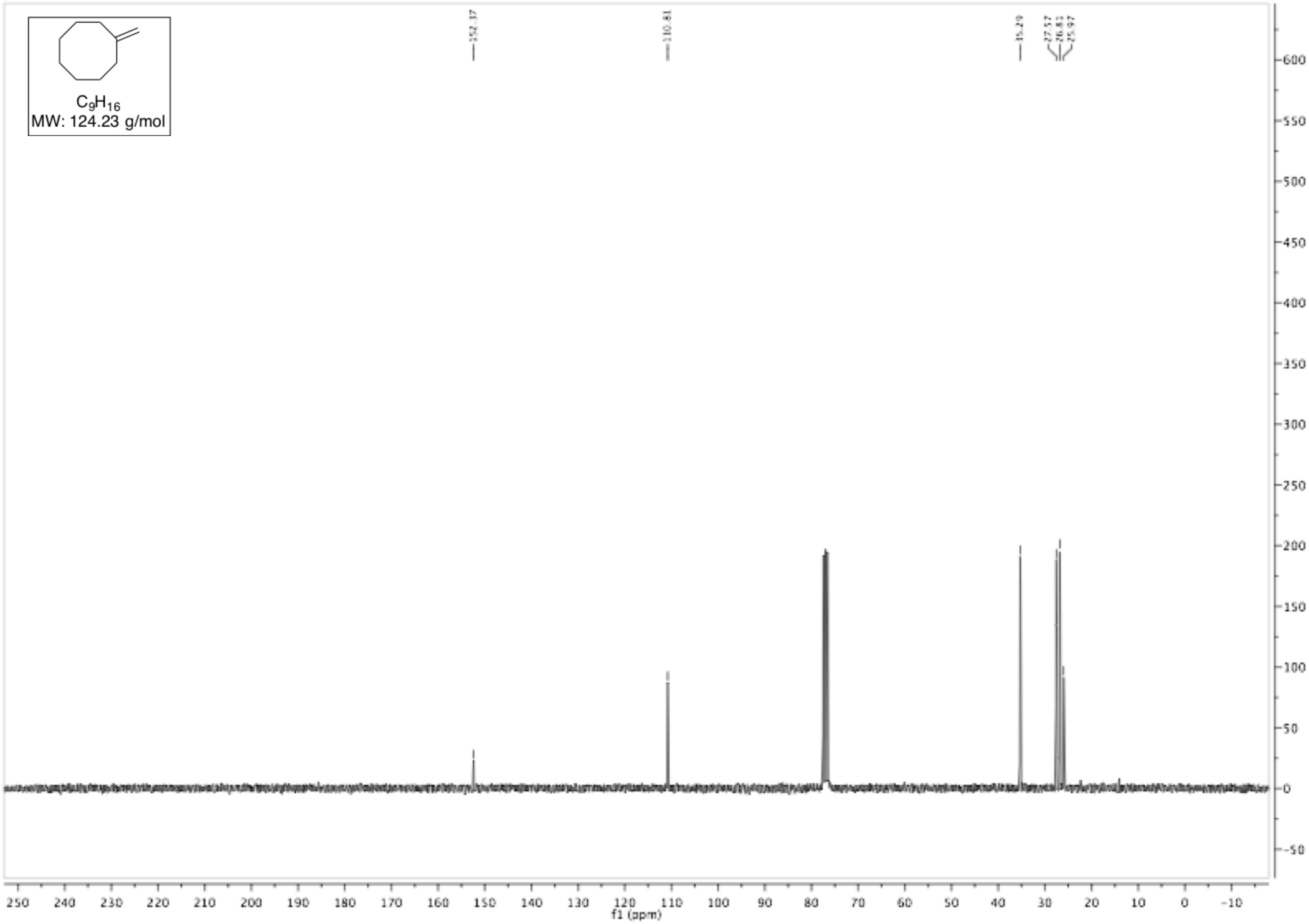

C9H16
MW: 124.23 g/mol
152.37
110.81
35.29
27.57
26.81
25.97
f1 (ppm)

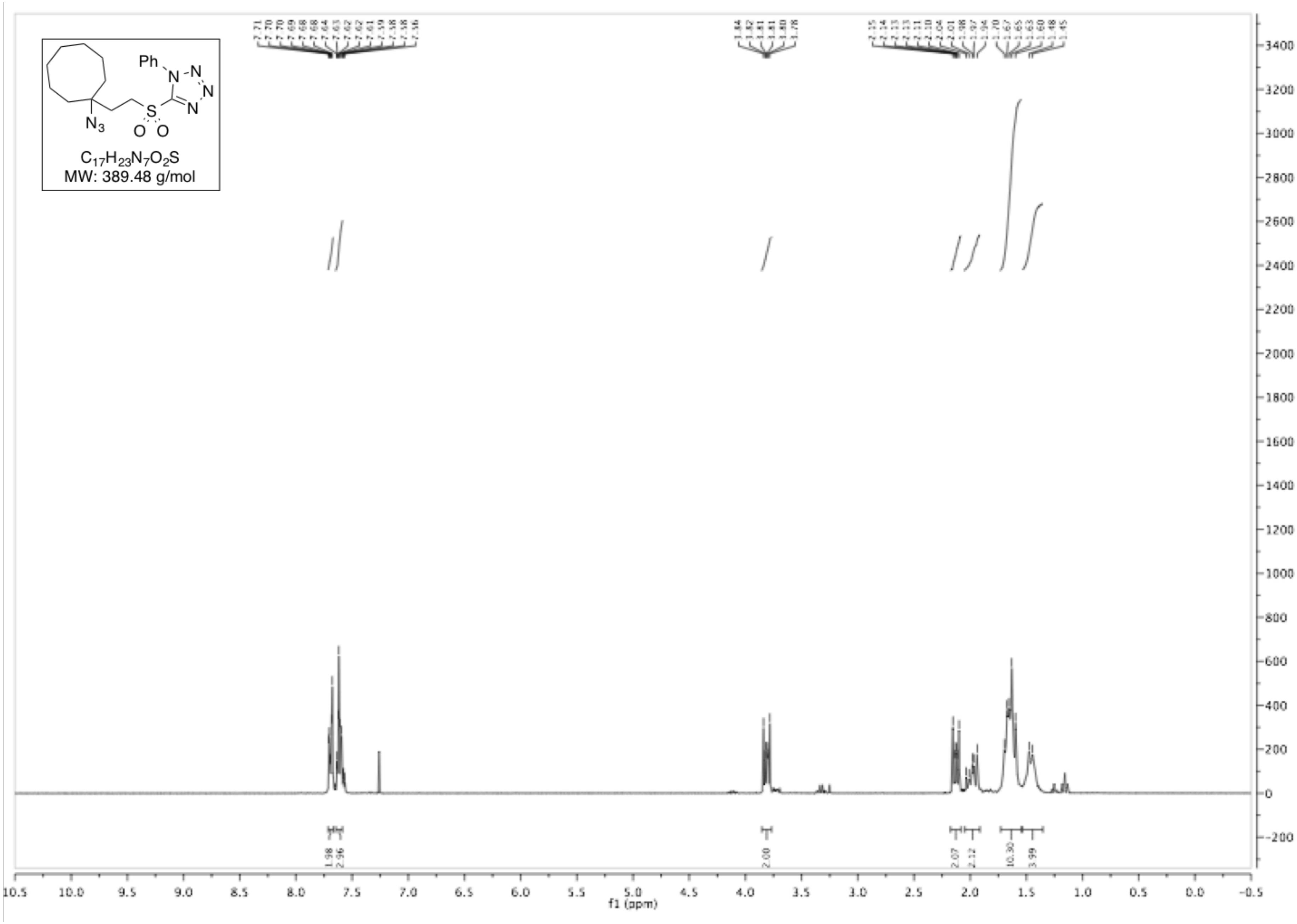
Ph
N
N
N
N
S
O O
N3
C17H23N7O2S
MW: 389.48 g/mol
f1 (ppm)

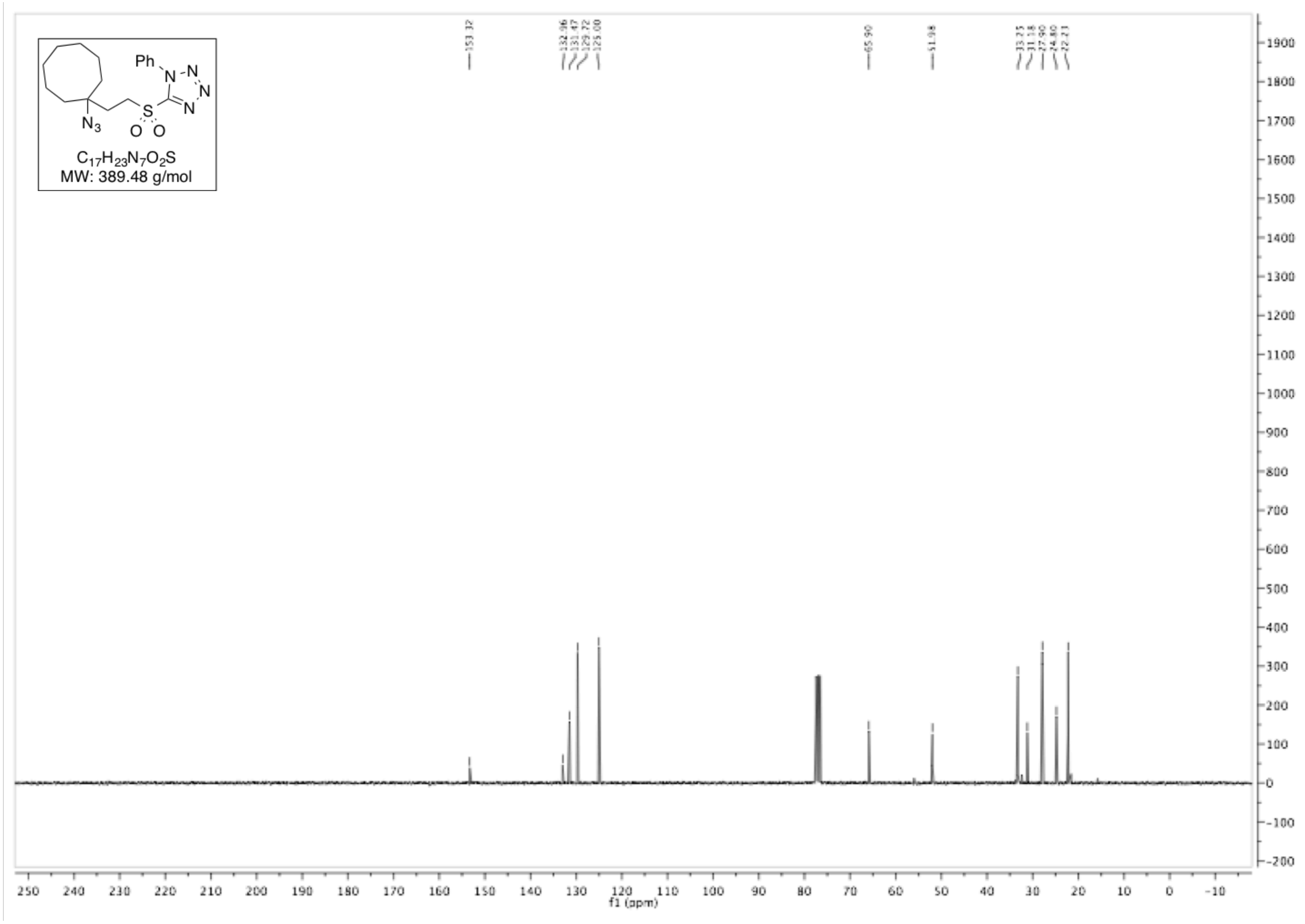

Ph
N
N
N
N
S
O
O
N3
C17H23N7O2S
MW: 389.48 g/mol
153.32
132.96
131.47
129.72
125.00
65.90
51.98
33.25
31.18
27.90
24.80
22.21
f1 (ppm)

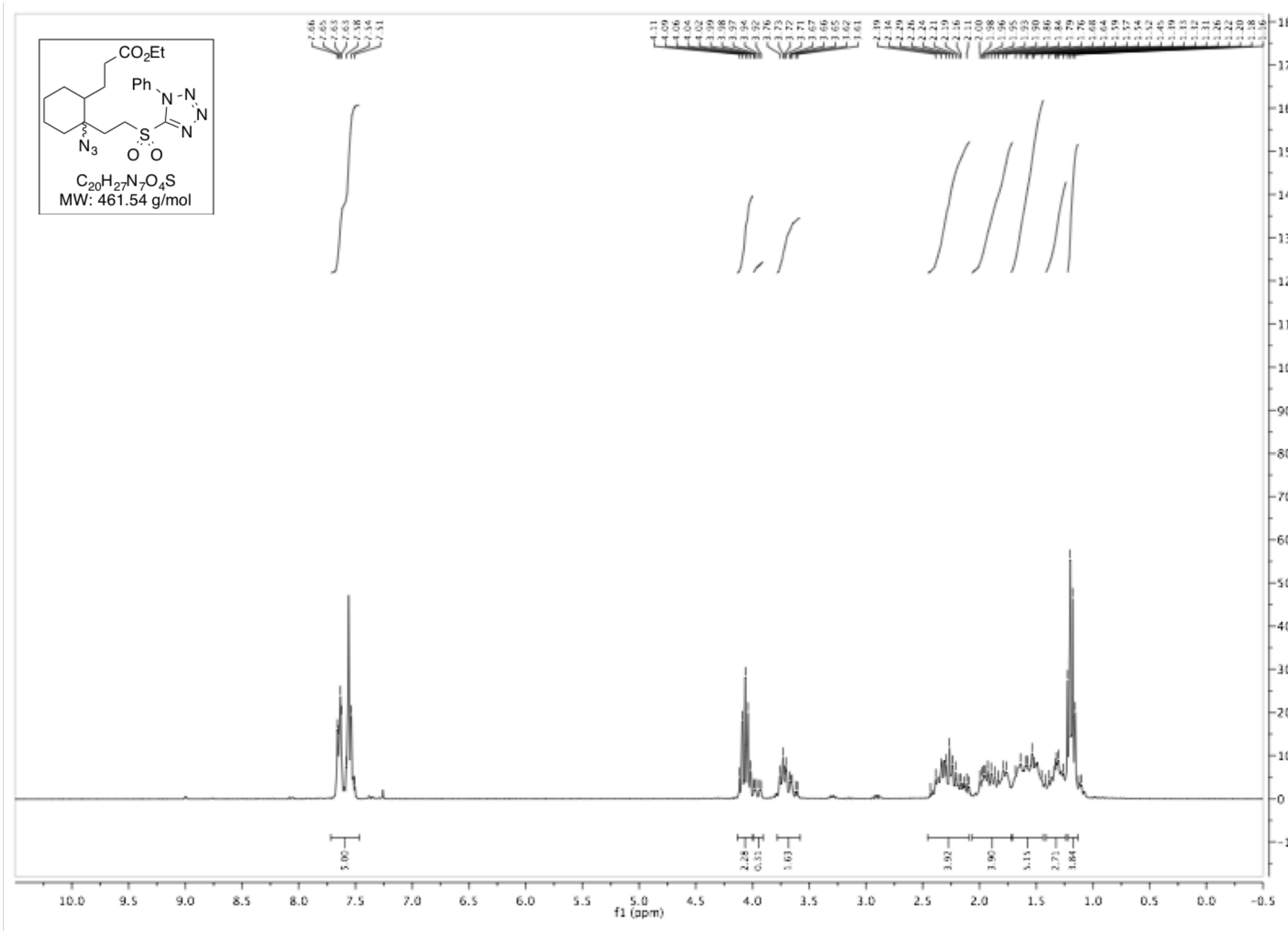

CO2Et
Ph
N
N
N
N
S
O O
N3
C20H27N7O4S
MW: 461.54 g/mol
f1 (ppm)
5.00
2.28
0.51
1.63
3.92
3.90
5.15
2.71
3.84

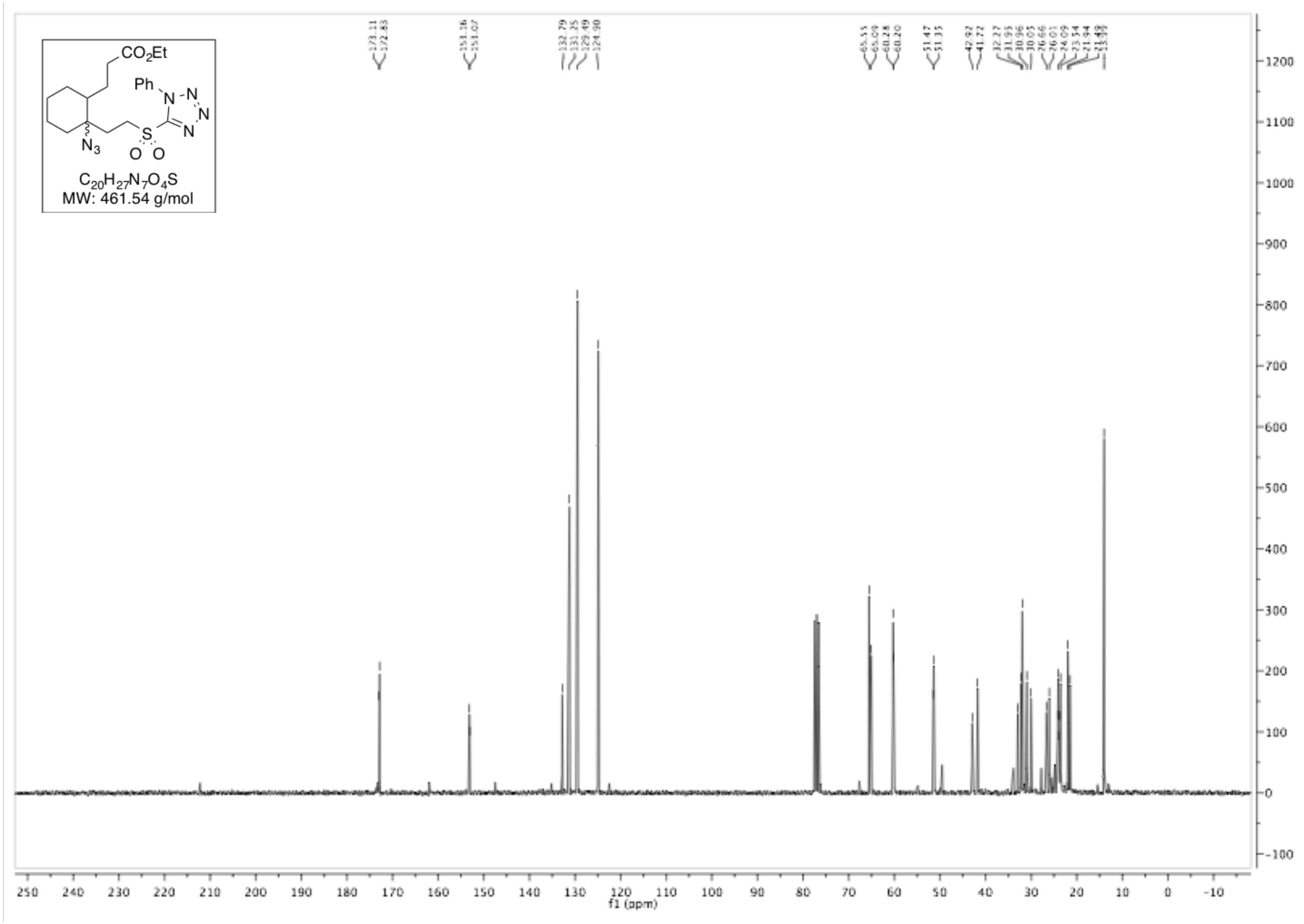

173.11
172.83
153.16
153.07
132.79
131.25
129.49
124.90
65.55
65.09
60.28
60.20
51.47
51.35
42.92
41.72
32.27
31.95
30.96
30.05
26.66
26.01
24.09
23.54
21.94
CO2Et
Ph
N
N3
S
O
C20H27N7O4S
MW: 461.54 g/mol
f1 (ppm)

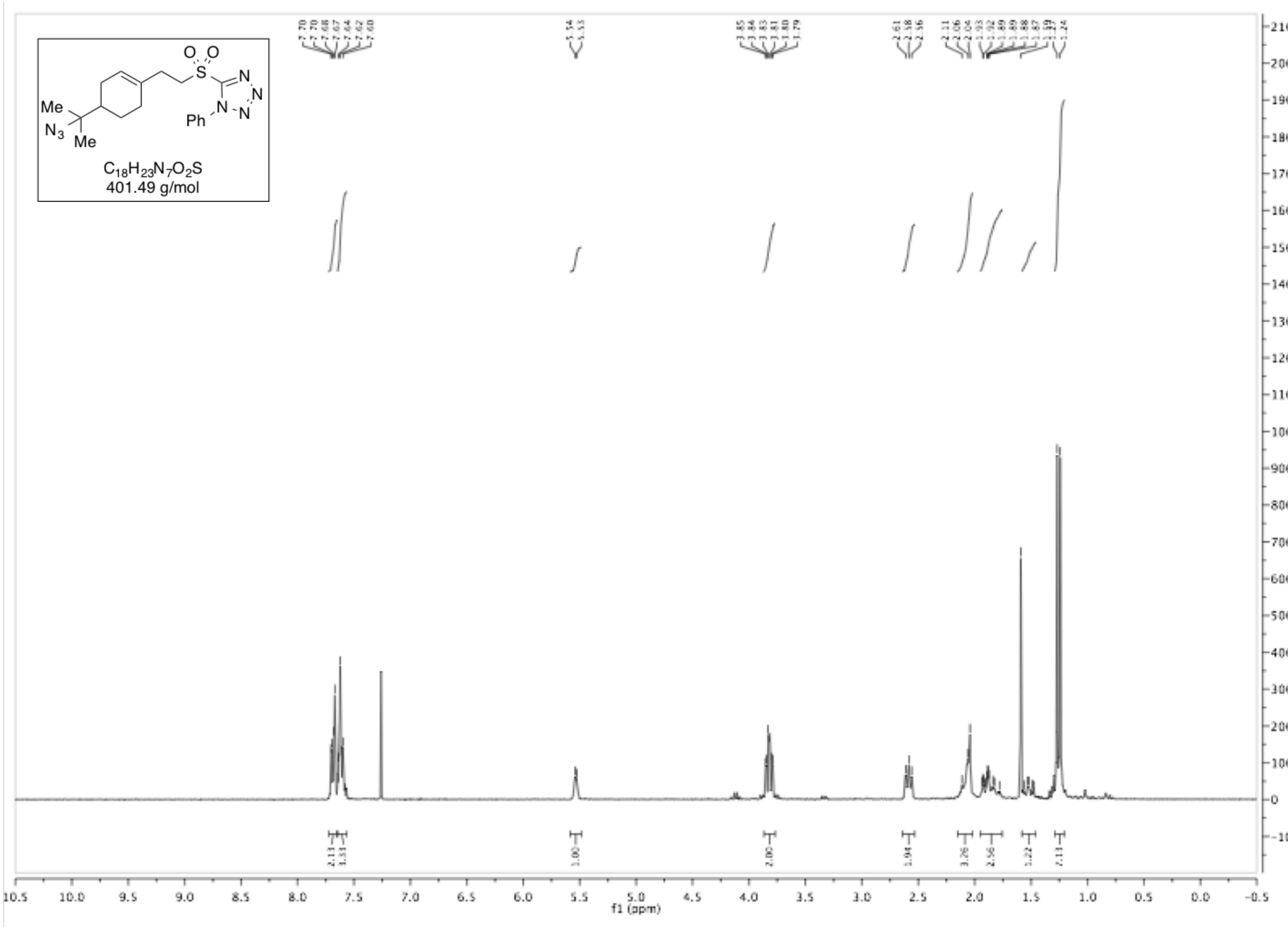

Me
N3
Me
Ph
C18H23N7O2S
401.49 g/mol
7.70 7.70 7.68 7.67 7.64 7.62 7.60
5.54 5.53
3.85 3.84 3.83 3.81 3.80 3.79
2.61 2.58 2.56
2.11 2.06 2.04 1.93 1.92 1.89 1.89 1.88 1.87 1.59 1.27 1.24
2.11 3.31
1.00
2.00
1.94
3.26
2.56
1.22
7.13
f1 (ppm)

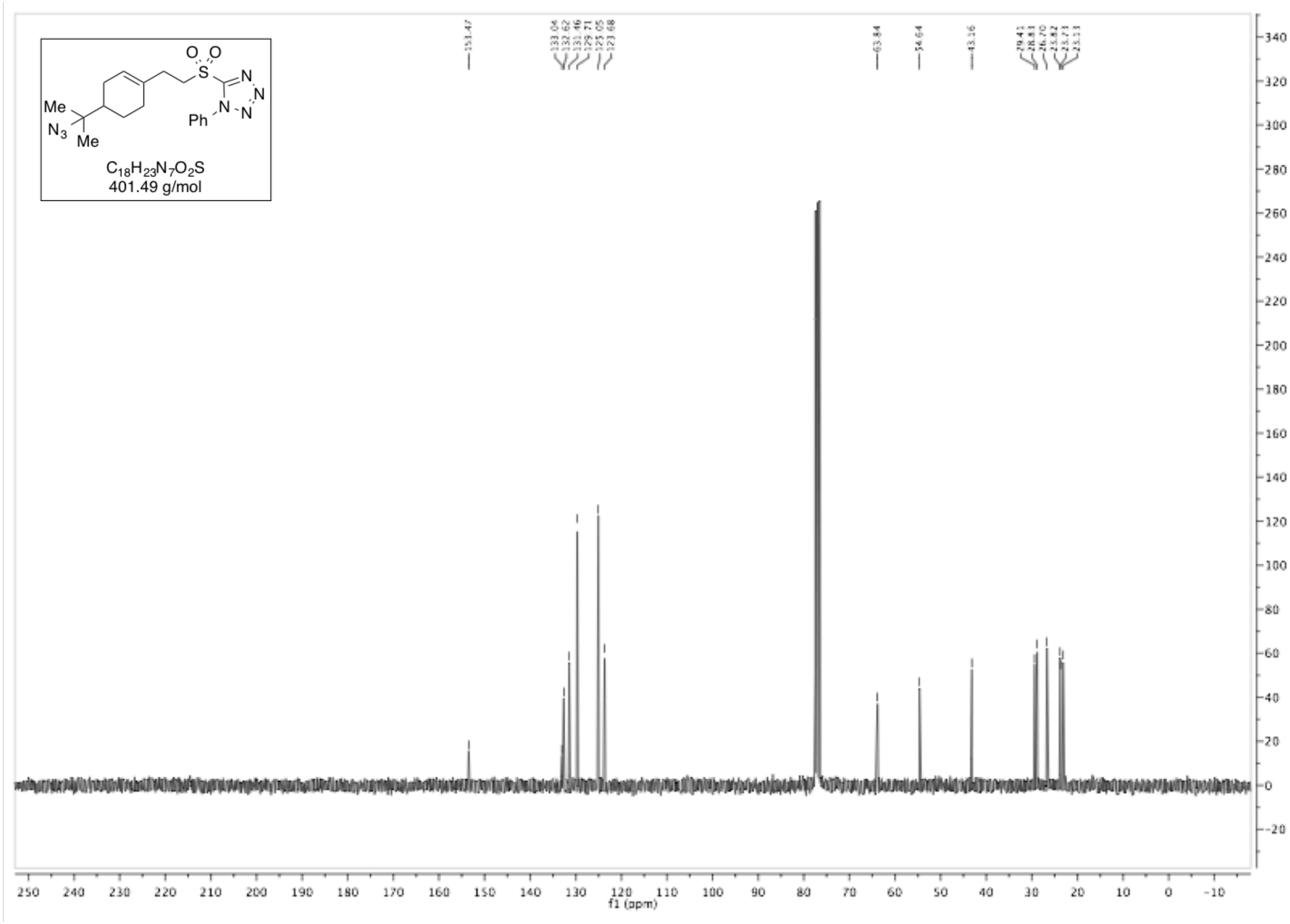

Me
N3
Me
Ph
C18H23N7O2S
401.49 g/mol
153.47
133.04
132.62
131.46
129.71
125.05
123.68
63.84
54.64
43.16
29.41
28.81
26.70
23.82
23.73
23.11
f1 (ppm)

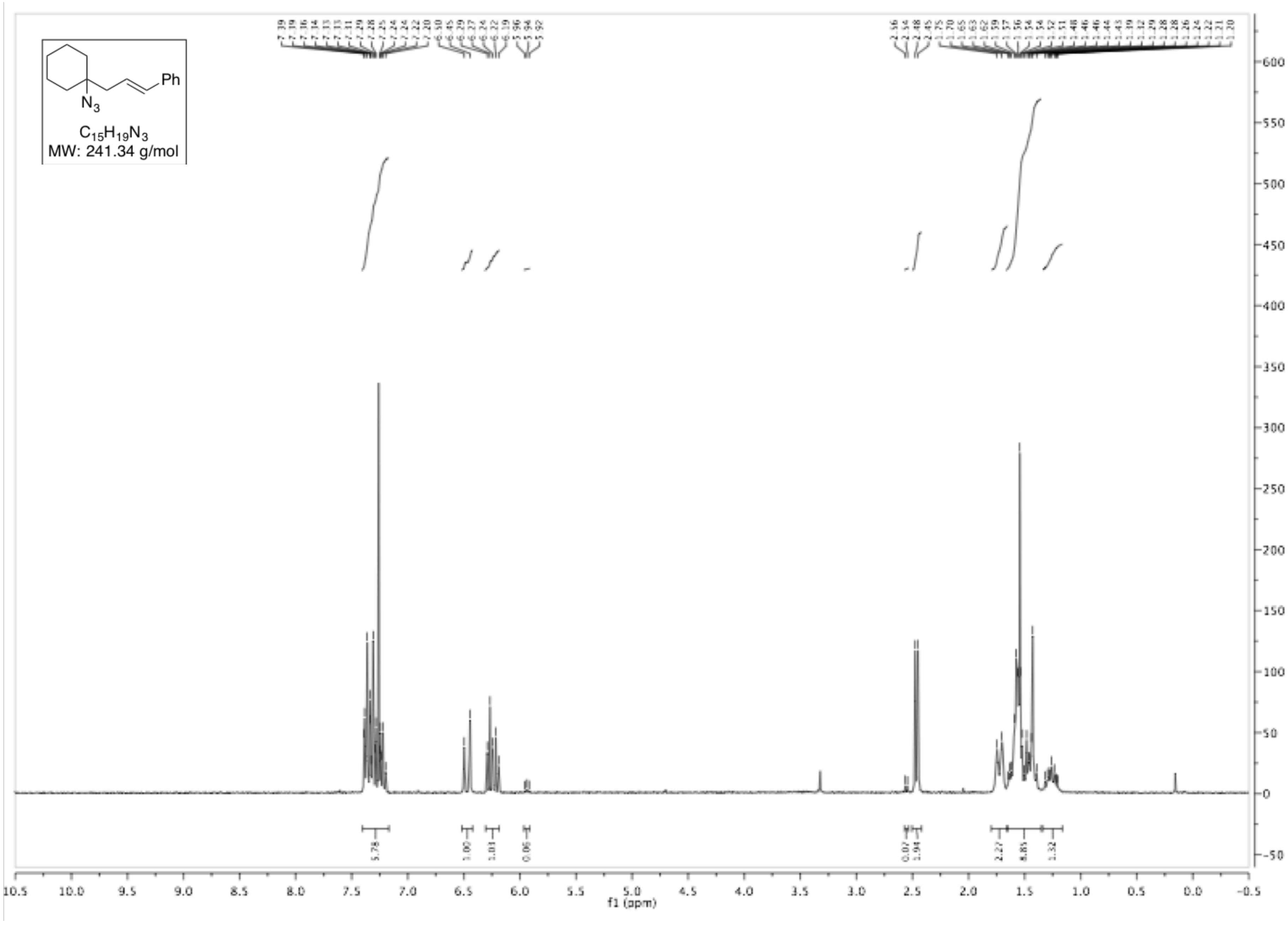

Ph
N_3
$C_{15}H_{19}N_3$
MW: 241.34 g/mol
7.39 7.39 7.36 7.34 7.33 7.31 7.29 7.28 7.25 7.24 7.24 7.22 7.20
6.50 6.45 6.29 6.27 6.24 6.22 6.19
5.96 5.94 5.92
2.56 2.54 2.48 2.45 1.75 1.70 1.65 1.63 1.62 1.59 1.57 1.56 1.56 1.54 1.54 1.52 1.51 1.48 1.46 1.46 1.44 1.43 1.39 1.32 1.29 1.28 1.28 1.26 1.24 1.22 1.21 1.20
5.78
1.00
1.03
0.06
0.07
1.94
2.27
8.85
1.32
f1 (ppm)

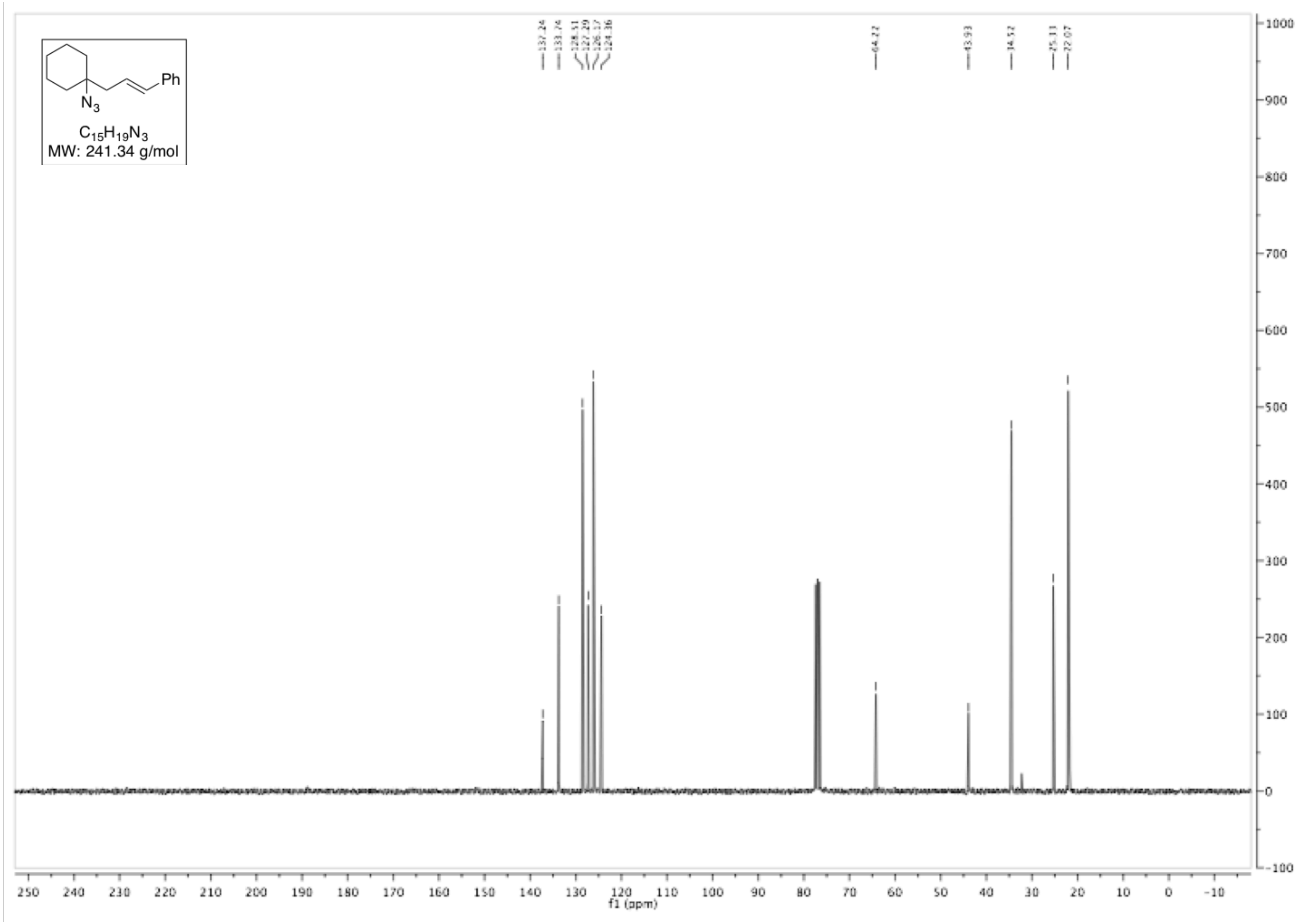

137.24
133.74
128.51
127.29
126.17
124.36
64.22
43.93
34.52
25.13
22.07
Ph
N_3
$C_{15}H_{19}N_3$
MW: 241.34 g/mol
f1 (ppm)

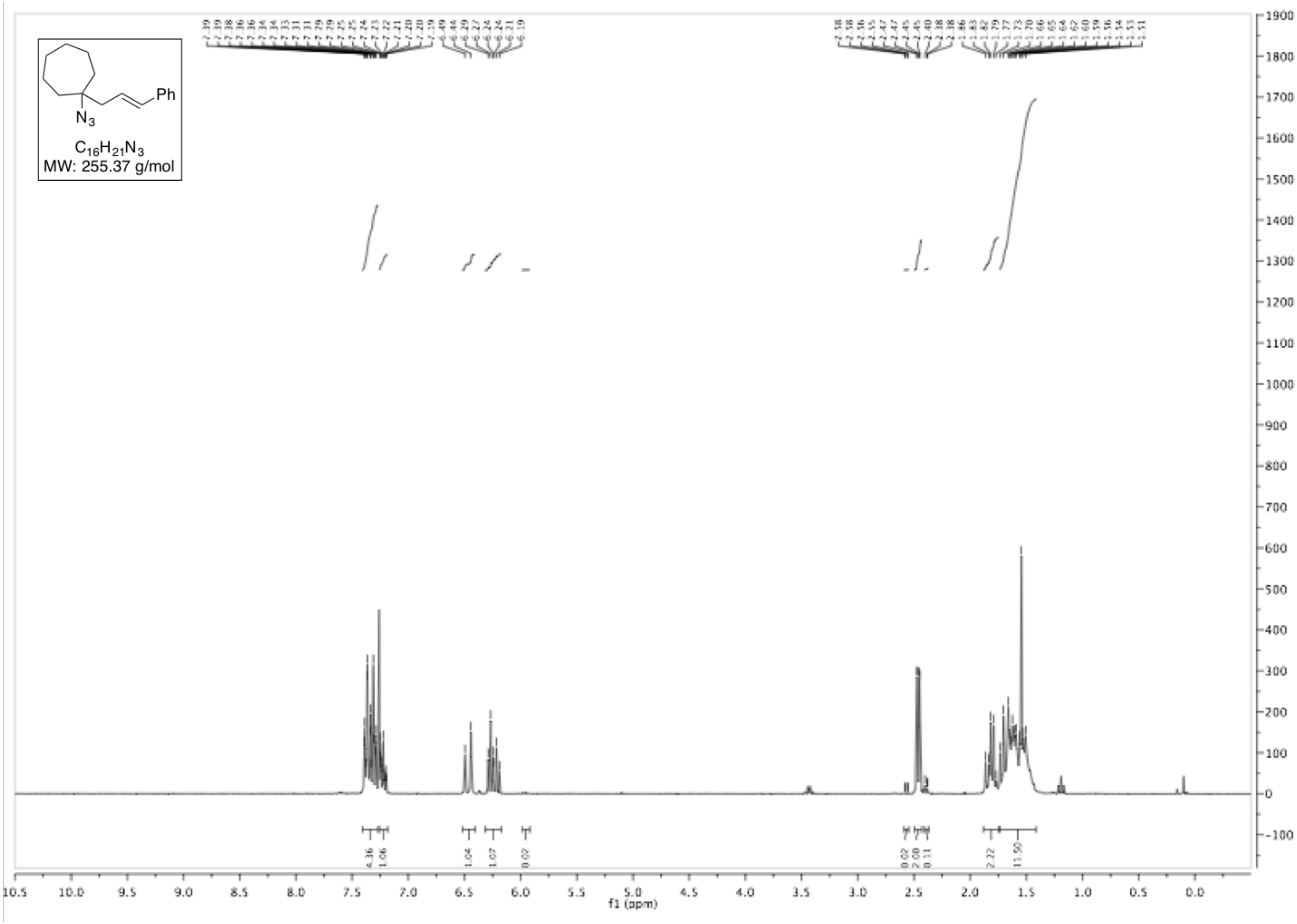

Ph
N3
C16H21N3
MW: 255.37 g/mol
f1 (ppm)

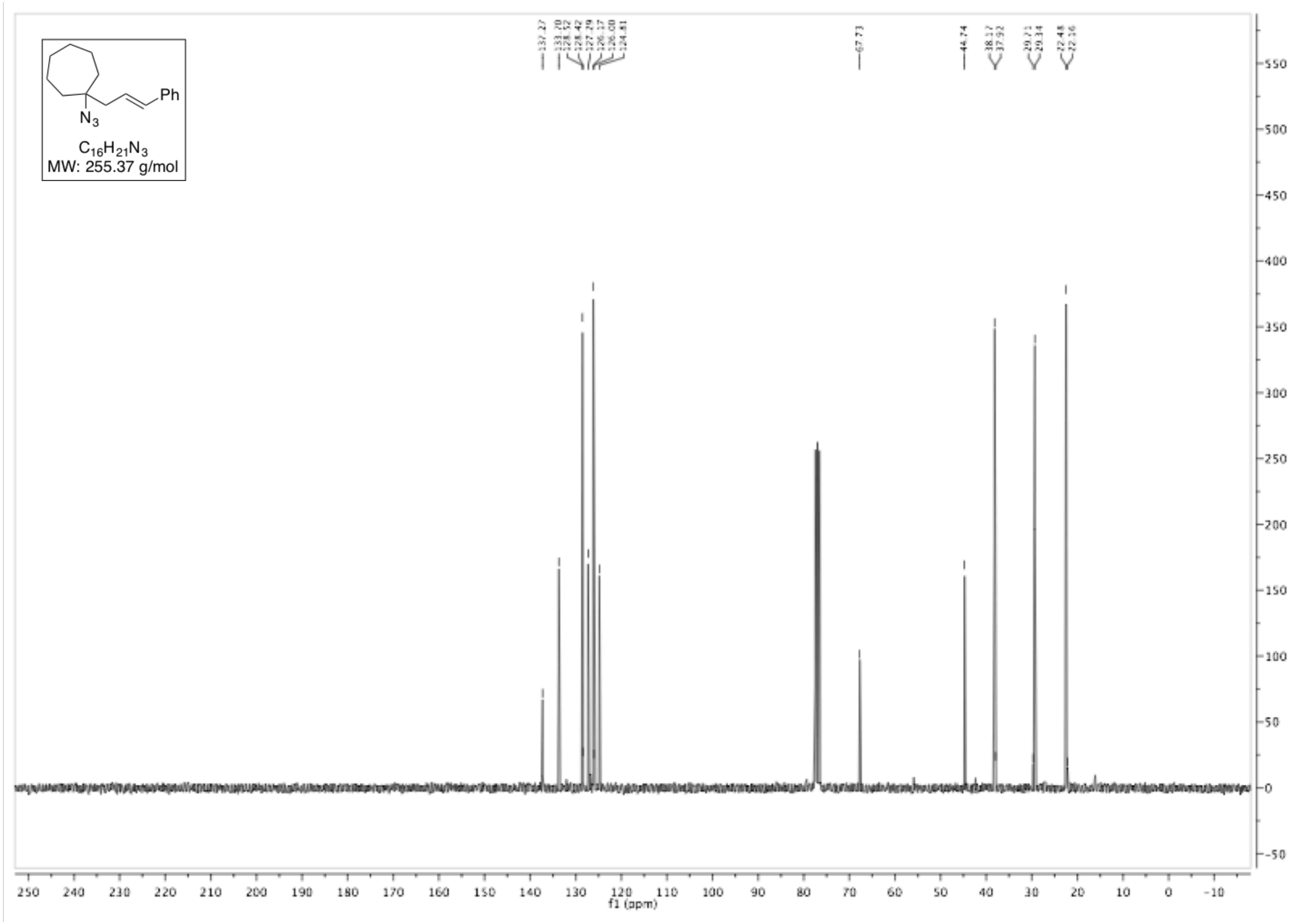

Ph
N_3
$C_{16}H_{21}N_3$
MW: 255.37 g/mol
137.27
133.70
128.52
128.42
127.29
126.17
126.00
124.81
67.73
44.74
38.17
37.92
29.71
29.34
22.48
22.16
f1 (ppm)

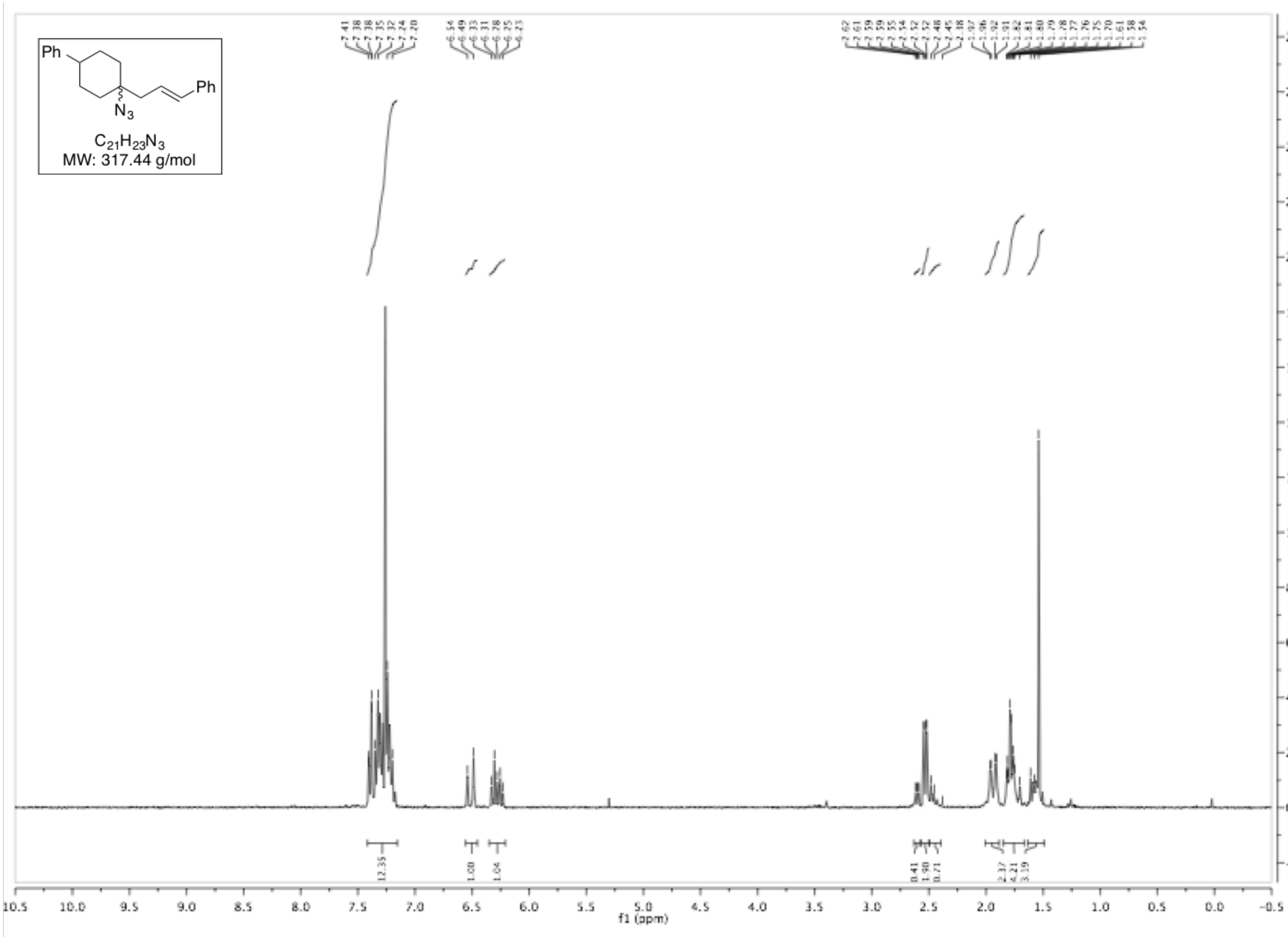
Ph
Ph
N3
$C_{21}H_{23}N_3$
MW: 317.44 g/mol
7.41 7.38 7.38 7.35 7.32 7.24 7.20
6.54 6.49 6.33 6.31 6.28 6.25 6.23
2.62 2.61 2.59 2.59 2.55 2.54 2.52 2.52 2.48 2.45 2.38 1.97 1.96 1.92 1.91 1.82 1.81 1.80 1.79 1.78 1.77 1.76 1.75 1.70 1.61 1.58 1.54
12.35
1.00
1.04
0.41
1.90
0.71
2.37
4.21
3.19
f1 (ppm)

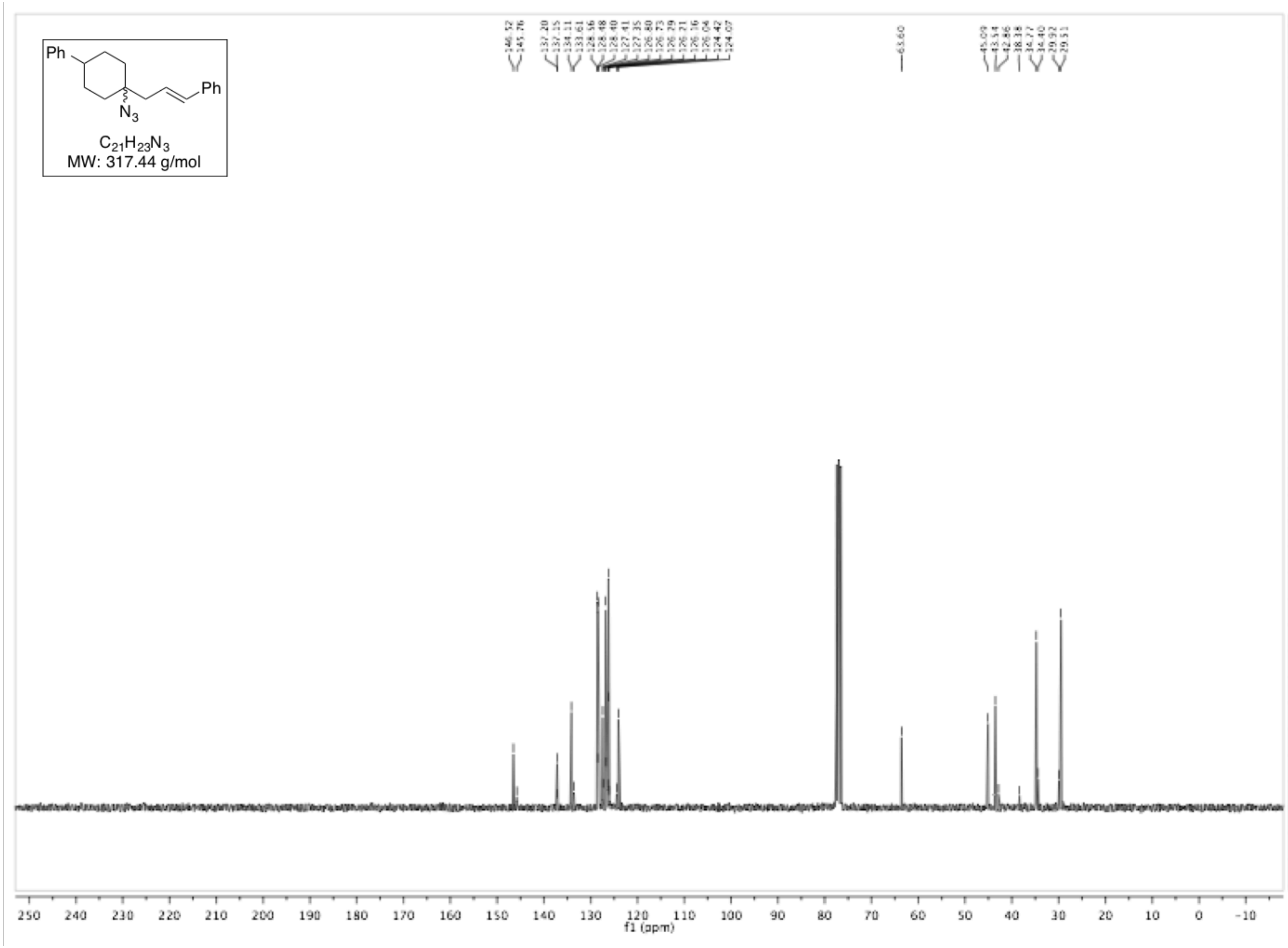
Ph
Ph
N3
C21H23N3
MW: 317.44 g/mol
146.52
145.76
137.20
137.15
134.11
133.61
128.56
128.48
128.40
127.41
127.35
126.80
126.73
126.29
126.21
126.16
126.04
124.42
124.07
63.60
45.09
43.54
42.86
38.38
34.77
34.40
29.92
29.51
f1 (ppm)

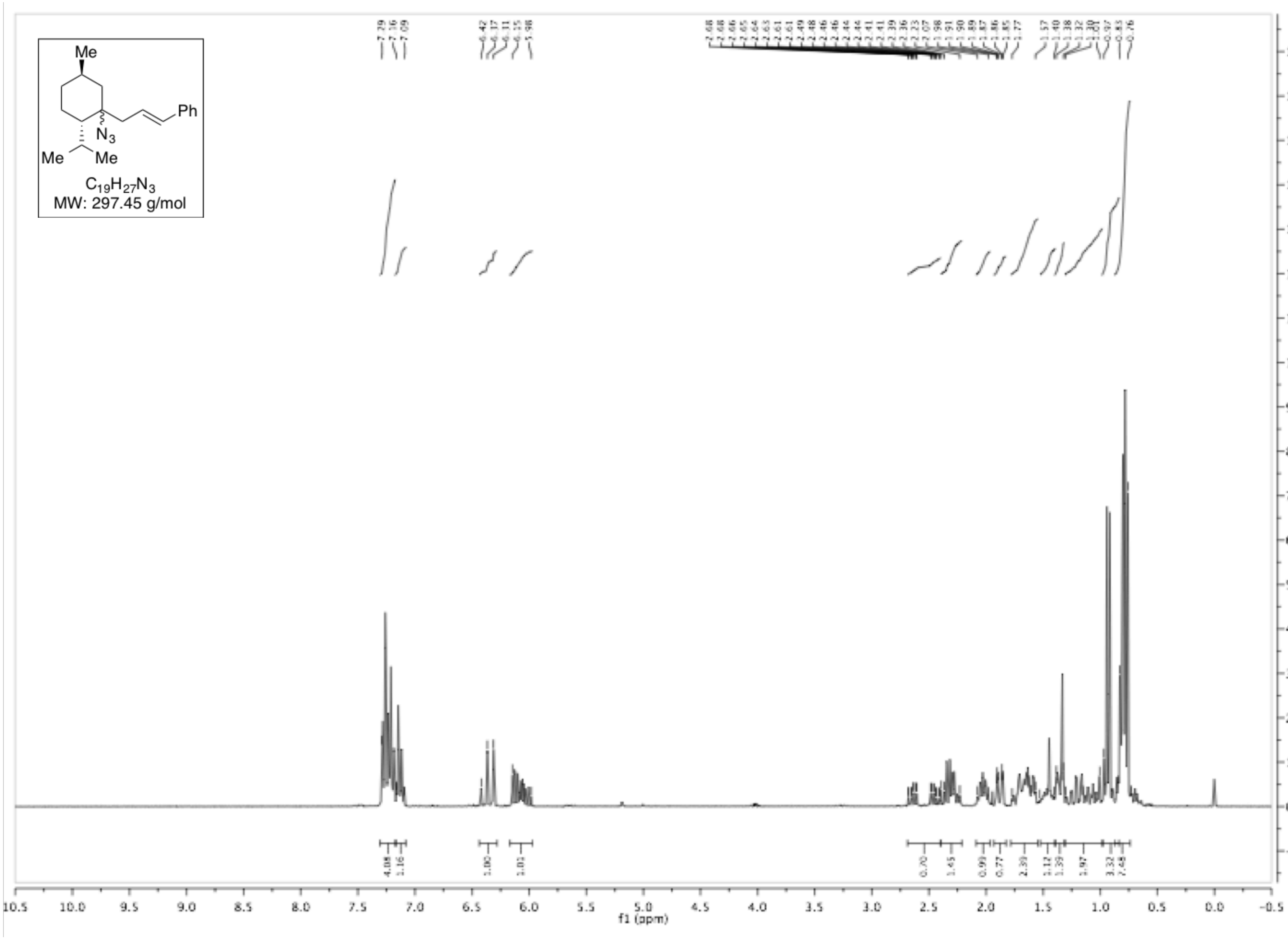
Me
Ph
N3
Me
Me
C19H27N3
MW: 297.45 g/mol
7.29 7.16 7.09
6.42 6.37 6.31 6.15 5.98
2.68 2.68 2.66 2.65 2.64 2.63 2.61 2.61 2.49 2.48 2.46 2.46 2.44 2.44 2.41 2.41 2.39 2.36 2.23 2.07 1.98 1.91 1.90 1.89 1.87 1.86 1.85 1.77 1.57 1.40 1.38 1.32 1.30 1.01 0.97 0.83 0.76
4.08 1.16 1.00 1.01 0.70 1.45 0.99 0.77 2.39 1.12 1.39 1.97 3.32 7.48
f1 (ppm)

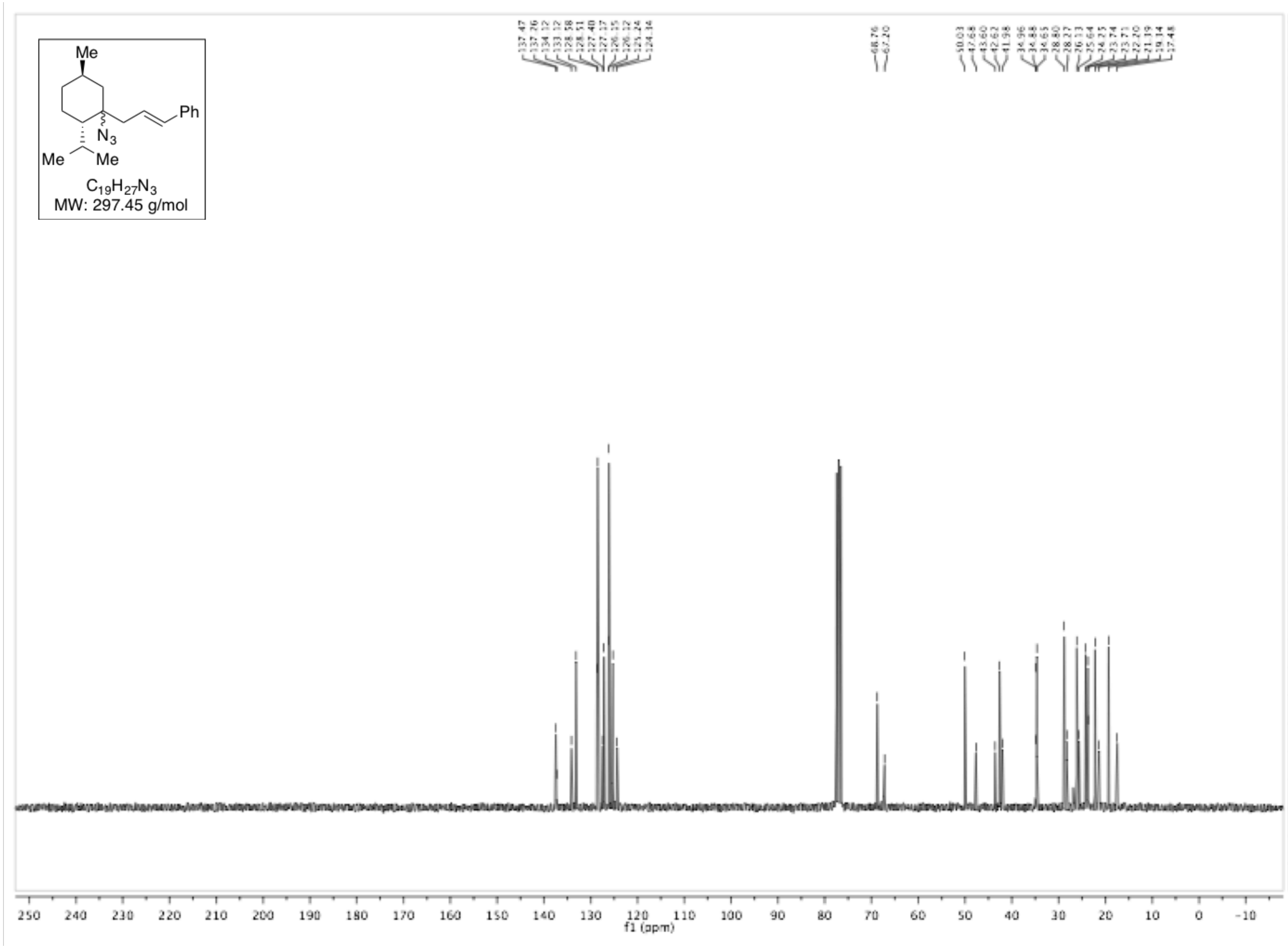
Me
Ph
N3
Me
Me
C19H27N3
MW: 297.45 g/mol
137.47
137.26
134.12
133.12
128.58
128.51
127.40
127.17
126.15
126.12
125.24
124.34
68.76
67.20
50.03
47.68
43.60
42.62
41.98
34.96
34.88
34.65
28.80
28.27
26.13
25.64
24.25
23.74
23.71
22.20
21.19
19.14
17.48
f1 (ppm)

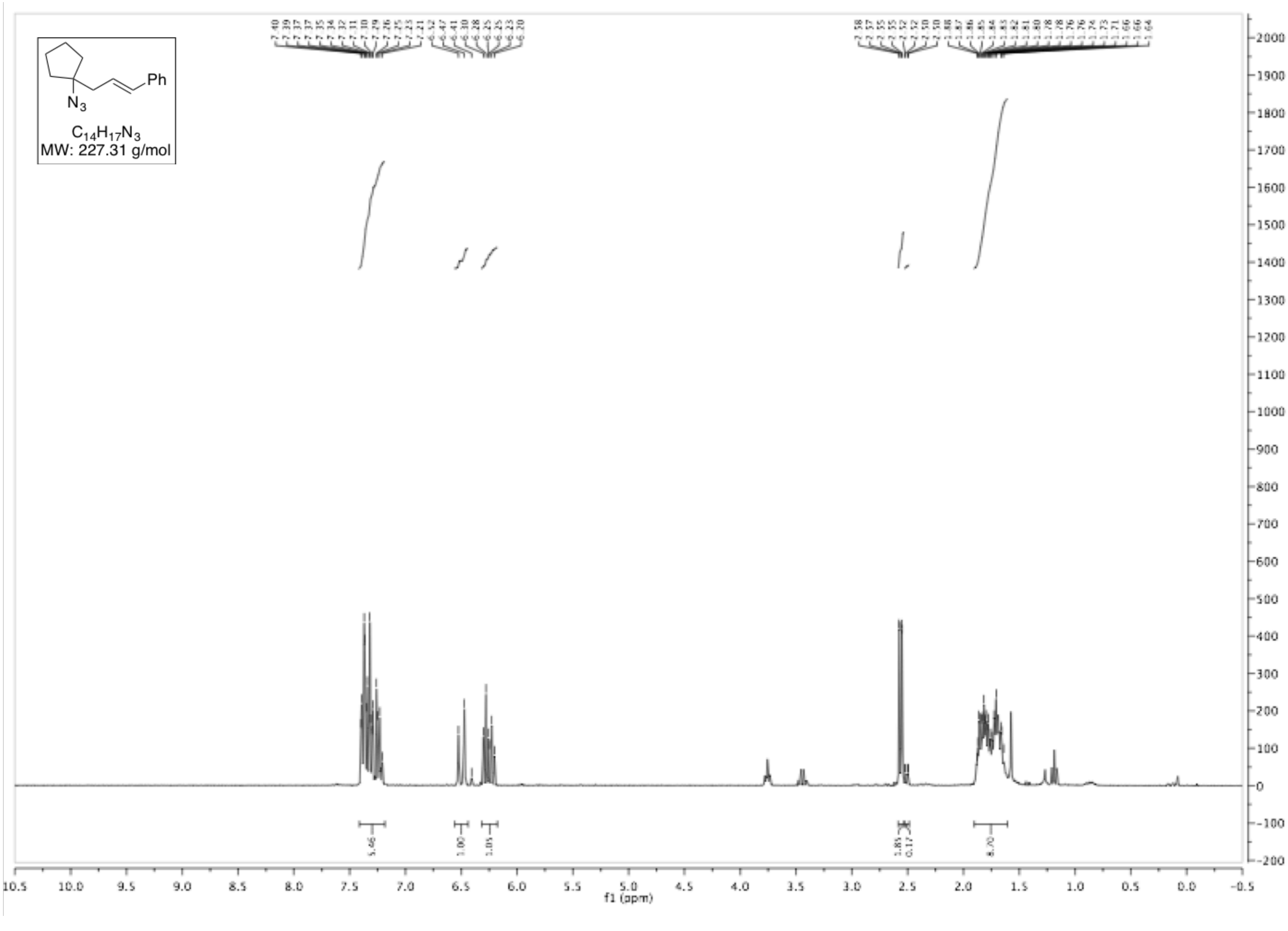

Ph
N_3
$C_{14}H_{17}N_3$
MW: 227.31 g/mol
7.40 7.39 7.37 7.37 7.35 7.34 7.32 7.31 7.30 7.29 7.26 7.25 7.23 7.21
6.52 6.47 6.41 6.30 6.28 6.25 6.25 6.23 6.20
2.58 2.57 2.55 2.55 2.52 2.52 2.50 2.50
1.88 1.87 1.86 1.85 1.84 1.83 1.82 1.81 1.80 1.78 1.78 1.76 1.76 1.74 1.73 1.71 1.66 1.66 1.64
5.46
1.00
1.05
1.85
0.17
8.70
f1 (ppm)

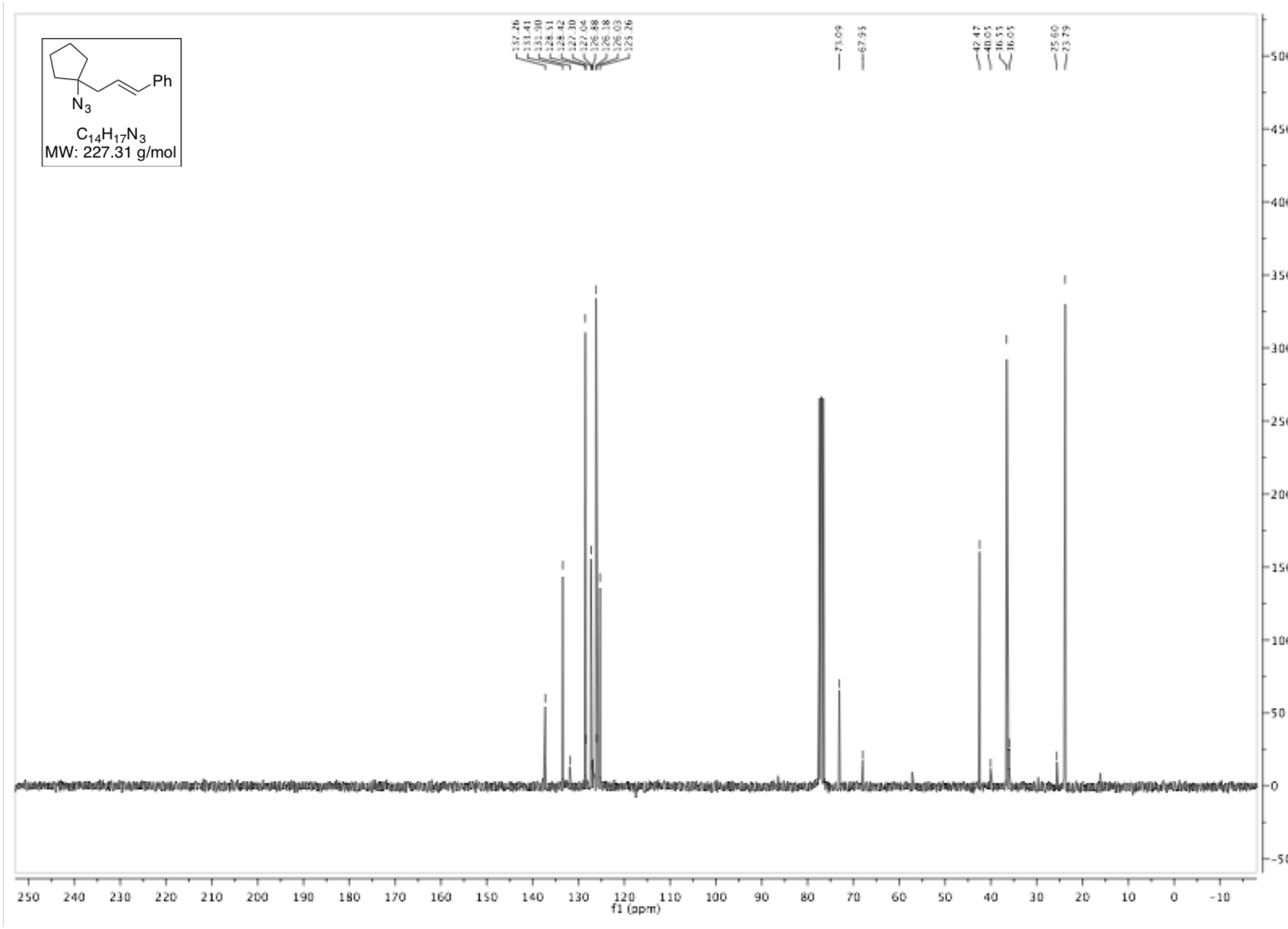
Ph
N3
C14H17N3
MW: 227.31 g/mol
137.26
131.41
131.90
128.51
128.42
127.30
127.04
126.88
126.18
126.03
125.26
73.09
67.95
42.47
40.05
36.55
36.05
25.60
23.79
f1 (ppm)

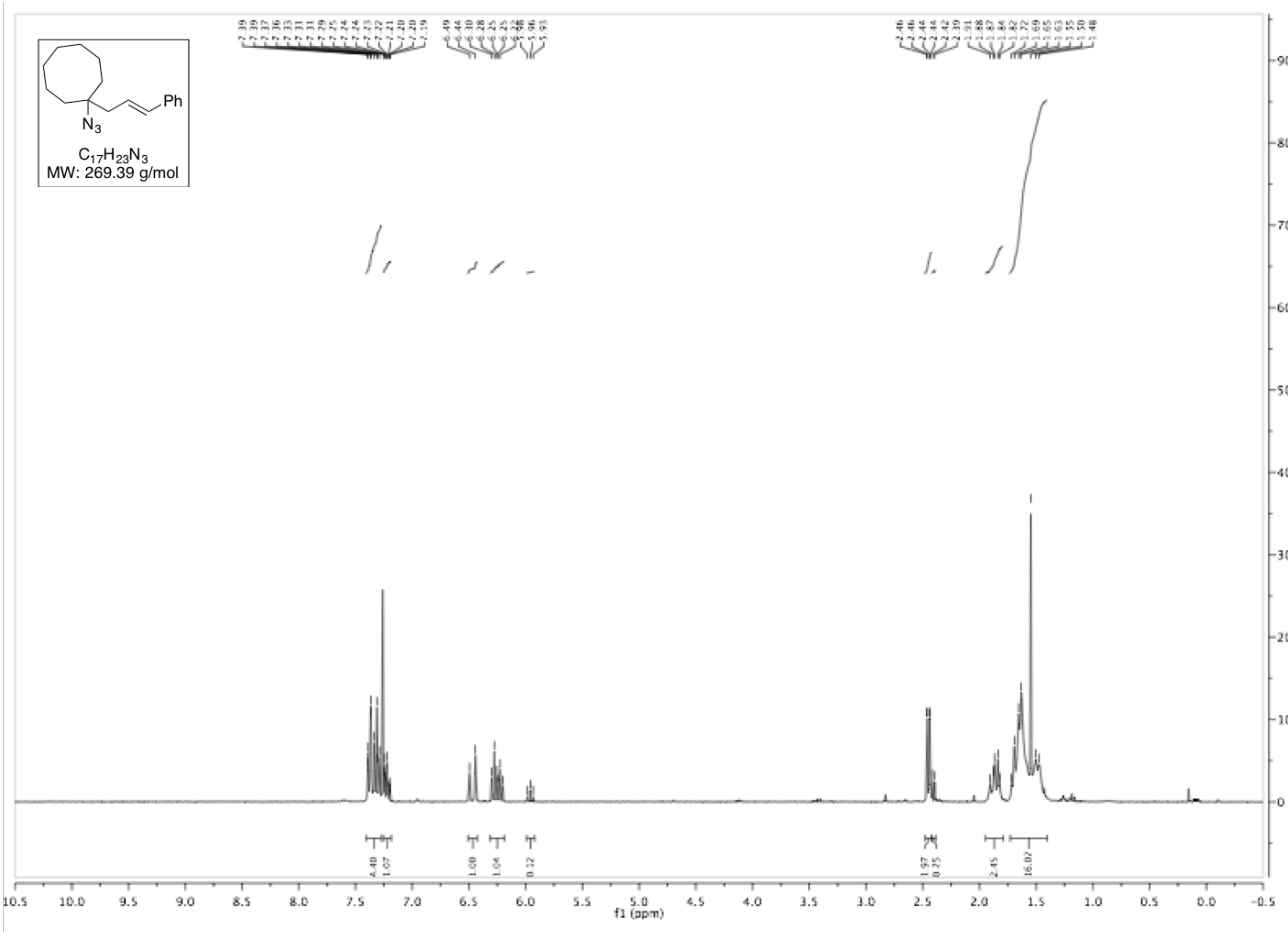
Ph
N_3
$C_{17}H_{23}N_3$
MW: 269.39 g/mol
4.40
1.07
1.00
1.04
0.12
1.97
0.25
2.45
16.07
f1 (ppm)

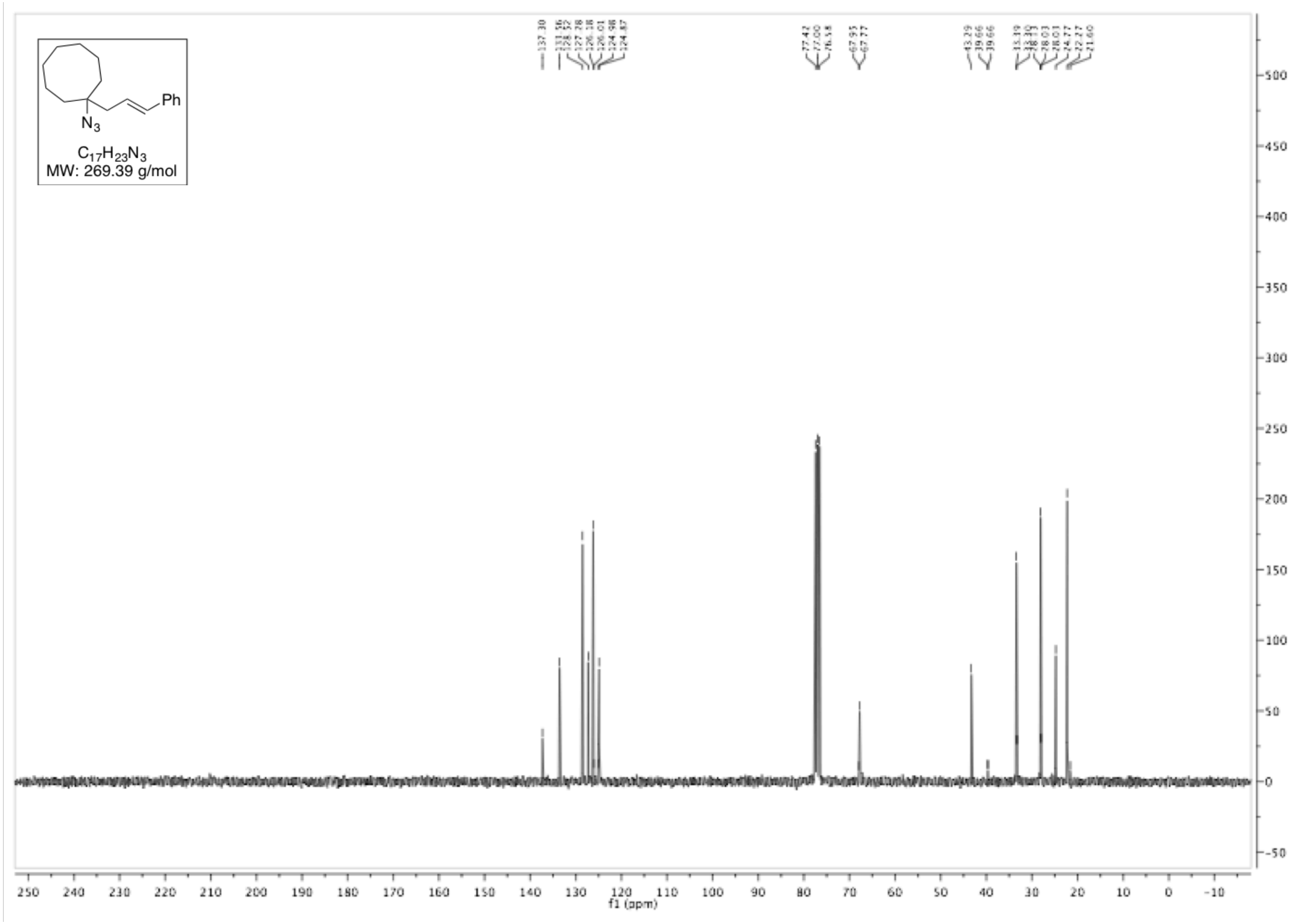

Ph
N3
C17H23N3
MW: 269.39 g/mol
137.30
133.56
128.52
127.28
126.18
126.01
124.98
124.87
77.42
77.00
76.58
67.95
67.77
43.29
39.66
39.66
33.39
33.30
28.13
28.03
28.01
24.77
22.27
21.60
f1 (ppm)

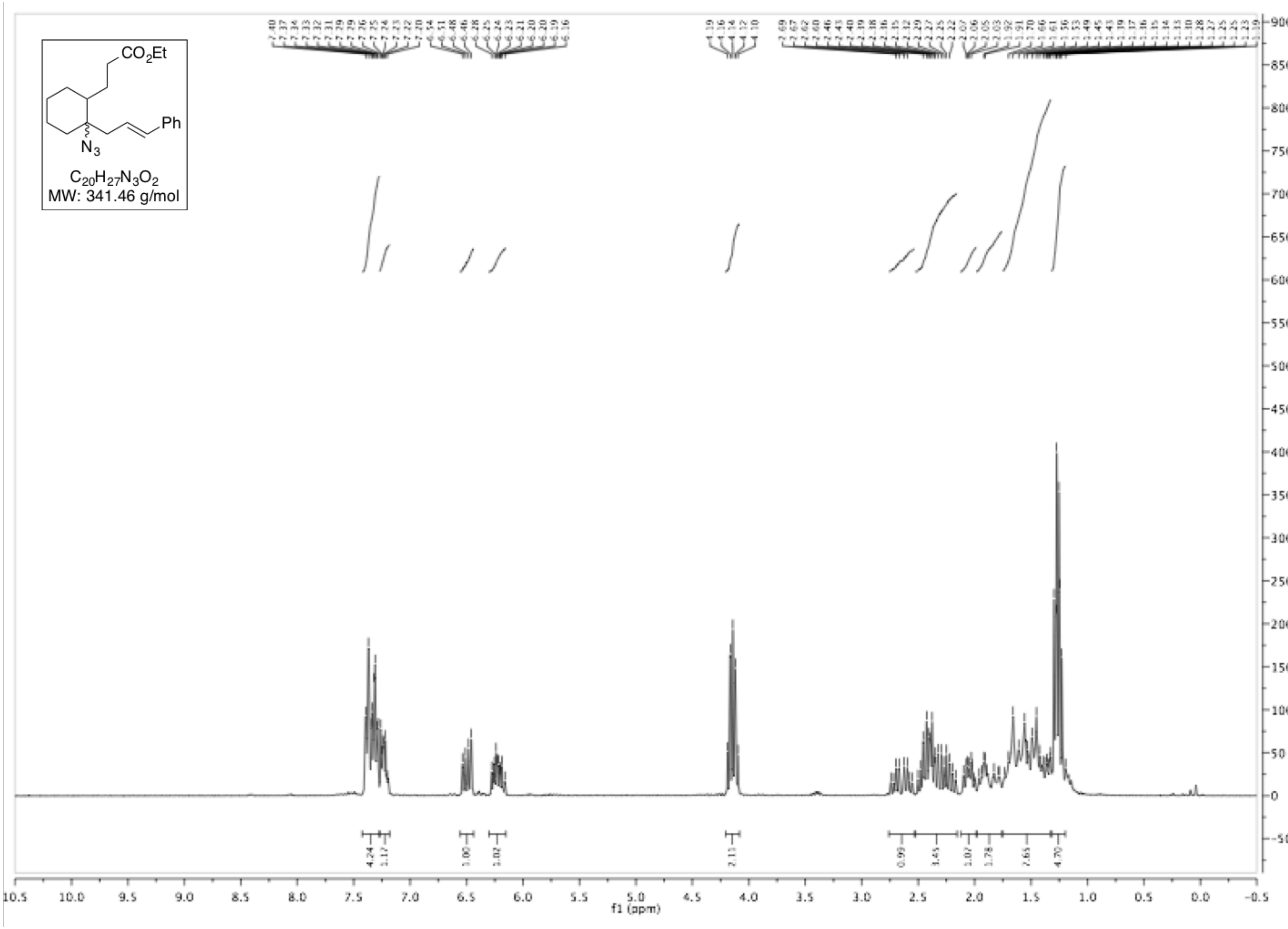
CO2Et
Ph
N3
C20H27N3O2
MW: 341.46 g/mol
f1 (ppm)

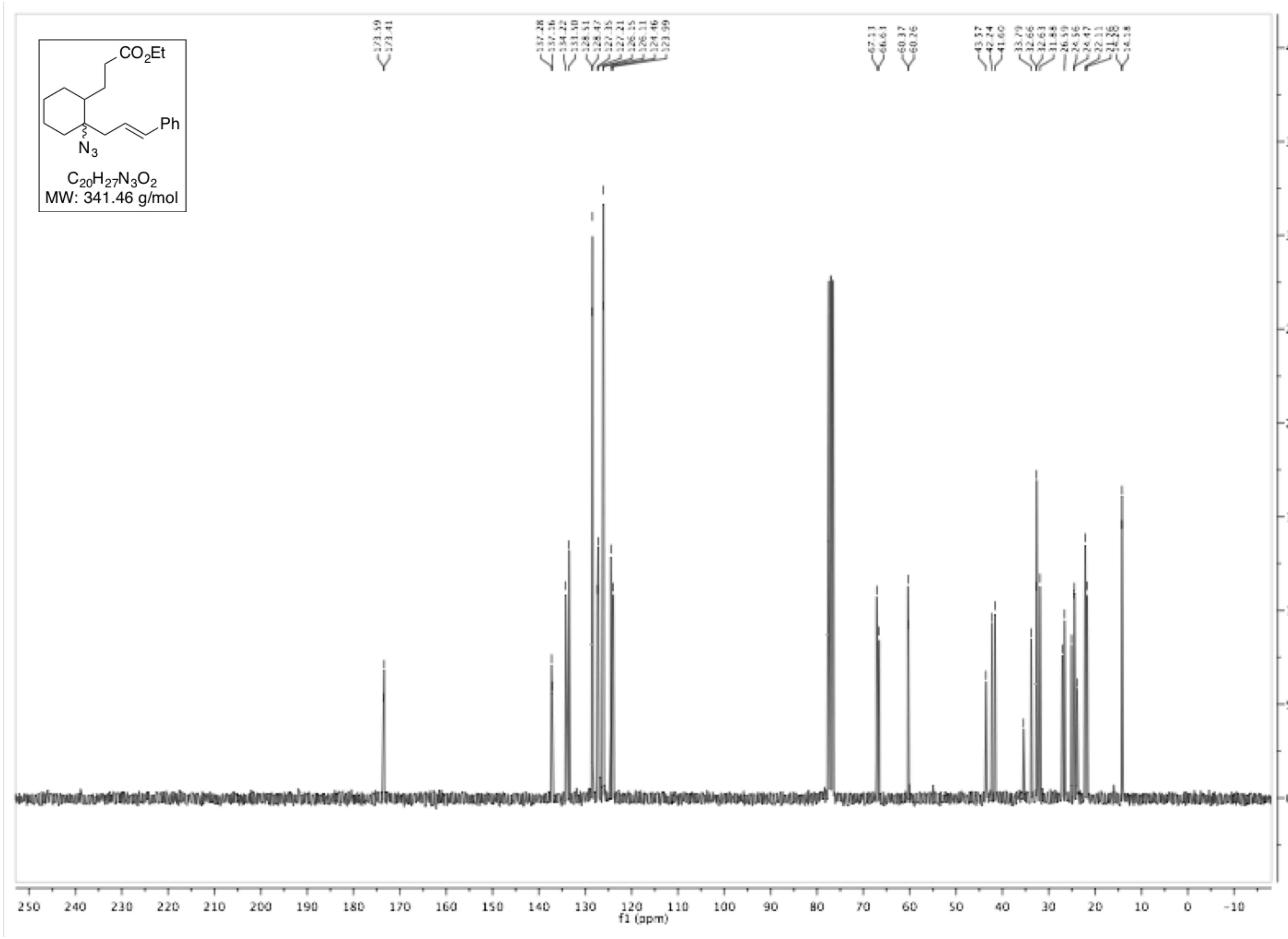
CO2Et
Ph
N3
C20H27N3O2
MW: 341.46 g/mol
173.59
173.41
137.28
137.16
134.22
133.50
128.51
128.47
127.35
127.21
126.15
126.11
124.46
123.99
67.13
66.63
60.37
60.26
43.57
42.24
41.60
33.79
32.66
32.63
31.88
26.59
24.56
24.47
22.11
21.36
14.26
14.18
f1 (ppm)

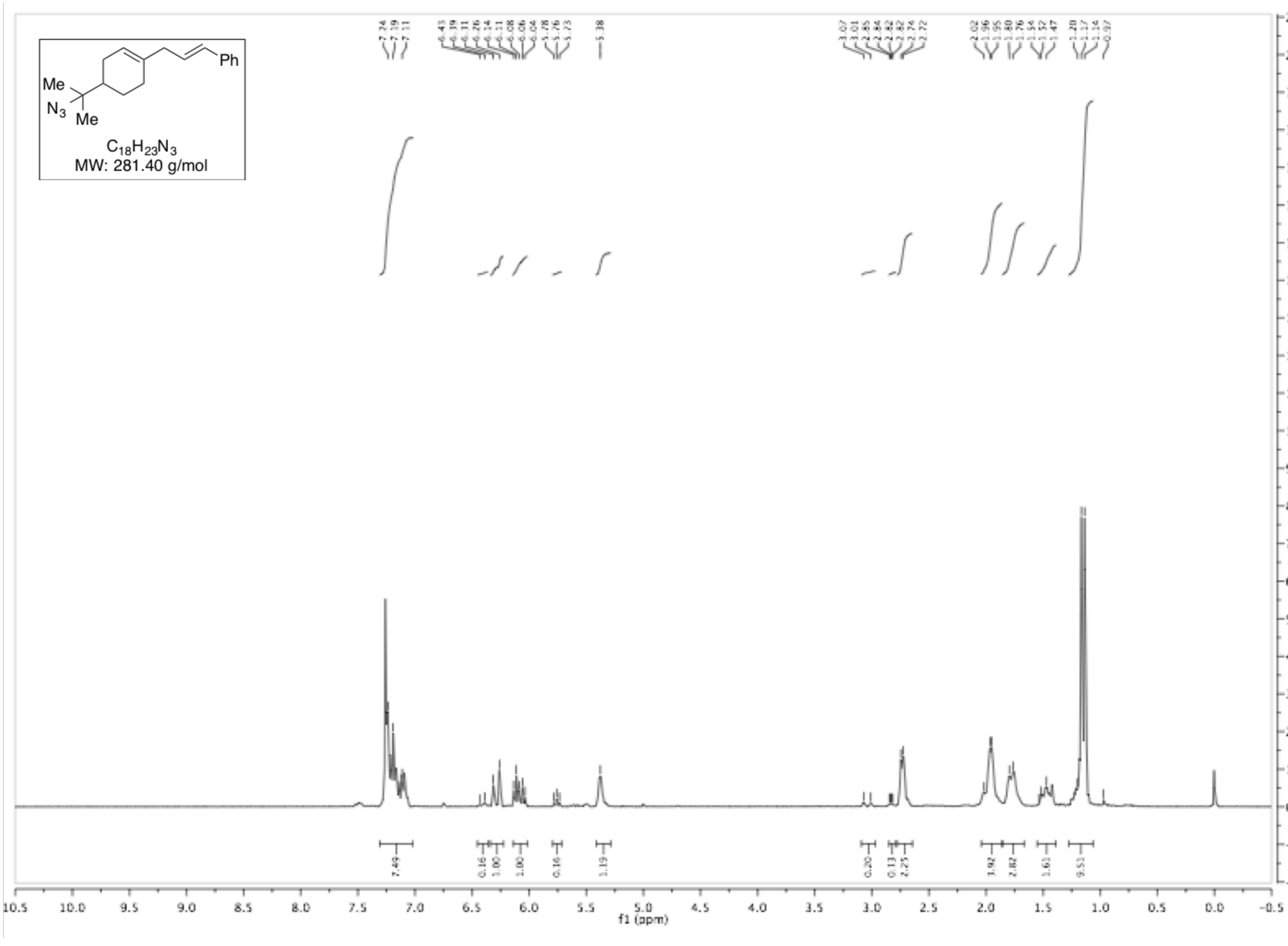
Me
N3
Me
Ph
C18H23N3
MW: 281.40 g/mol
f1 (ppm)

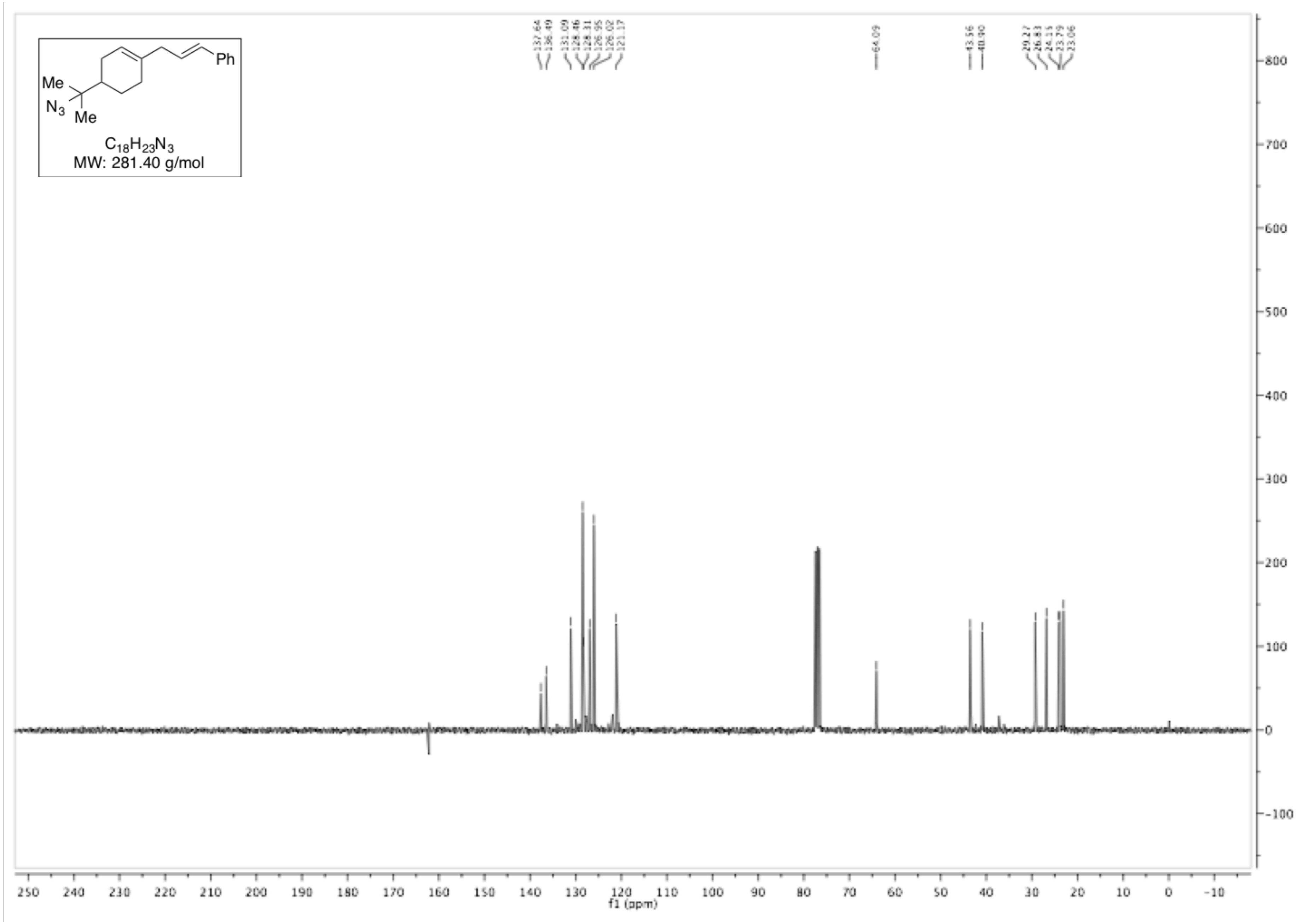
Me
N3
Me
Ph
C18H23N3
MW: 281.40 g/mol
137.64
136.49
131.09
128.46
128.31
126.95
126.02
121.17
64.09
43.56
40.90
29.27
26.83
24.15
23.79
23.06
f1 (ppm)

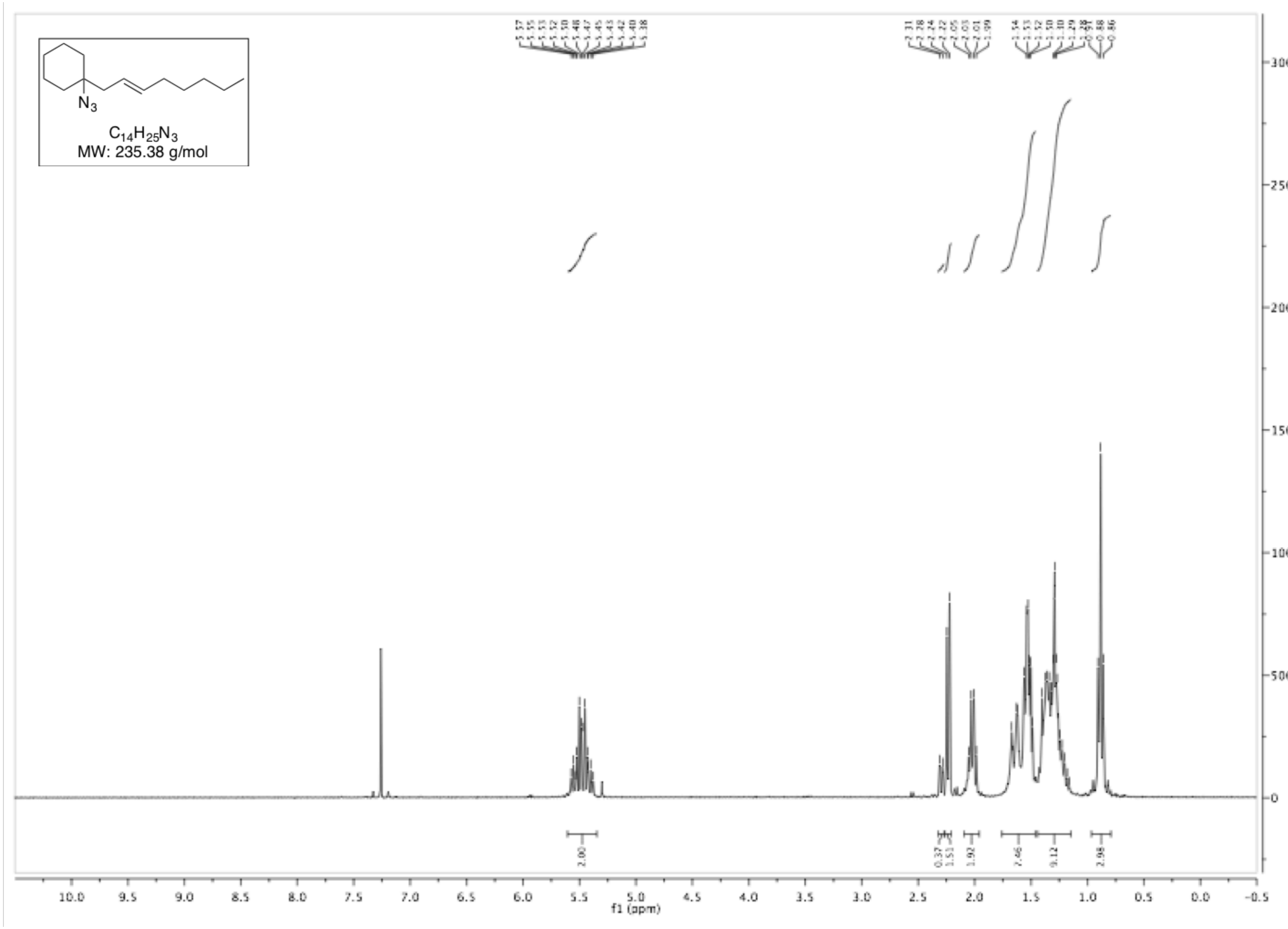

N3
C14H25N3
MW: 235.38 g/mol
5.57 5.55 5.53 5.52 5.50 5.48 5.47 5.45 5.43 5.42 5.40 5.38
2.31 2.28 2.24 2.22 2.05 2.03 2.01 1.99
1.54 1.53 1.52 1.50 1.30 1.29 1.28 0.91 0.88 0.86
2.00
0.37 1.51 1.92 7.46 9.12 2.98
f1 (ppm)

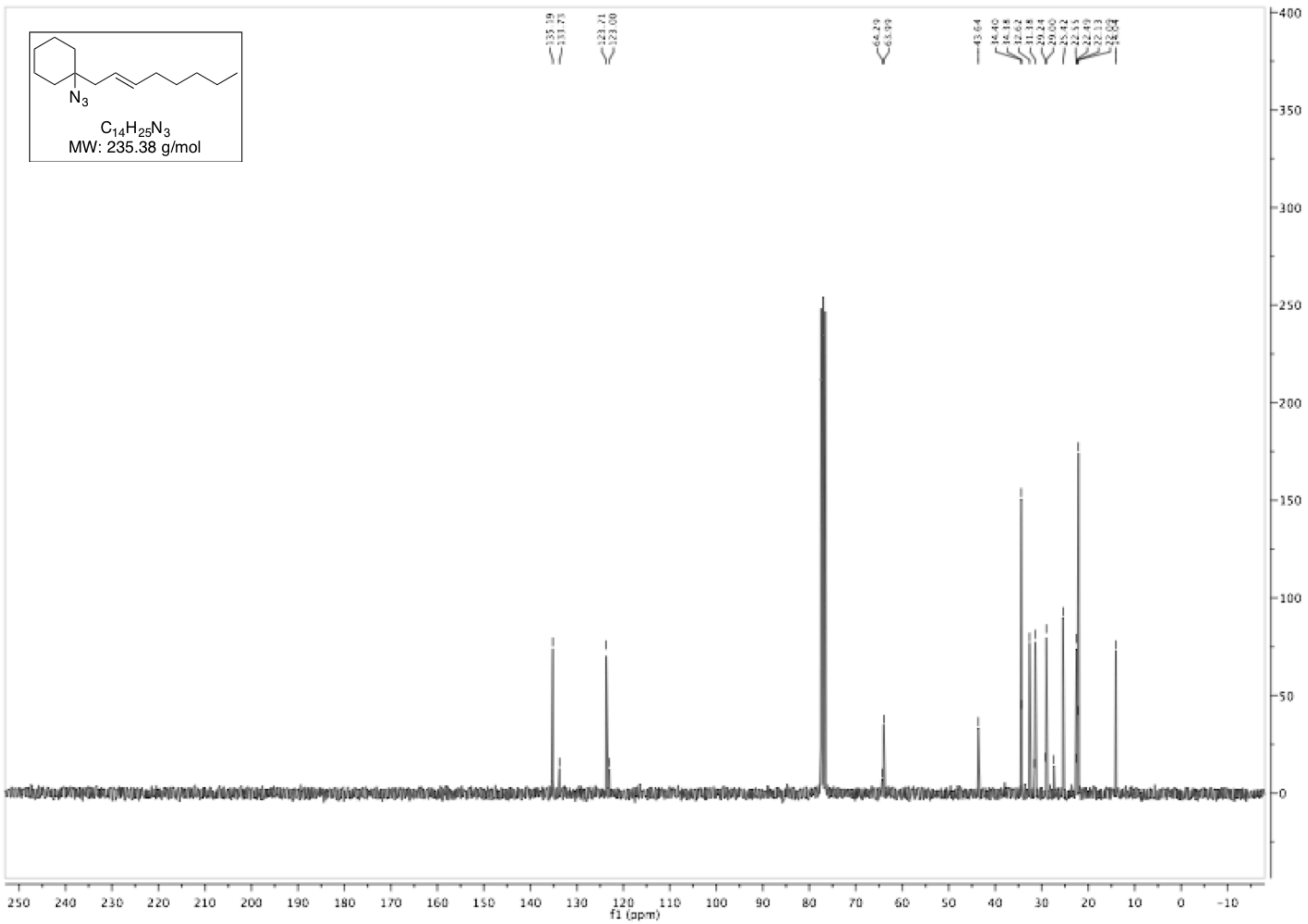

N3
C14H25N3
MW: 235.38 g/mol
135.19
133.75
123.71
123.00
64.29
63.99
43.64
34.40
34.18
32.62
31.18
29.24
29.00
25.42
22.55
22.49
22.13
22.00
14.04
f1 (ppm)

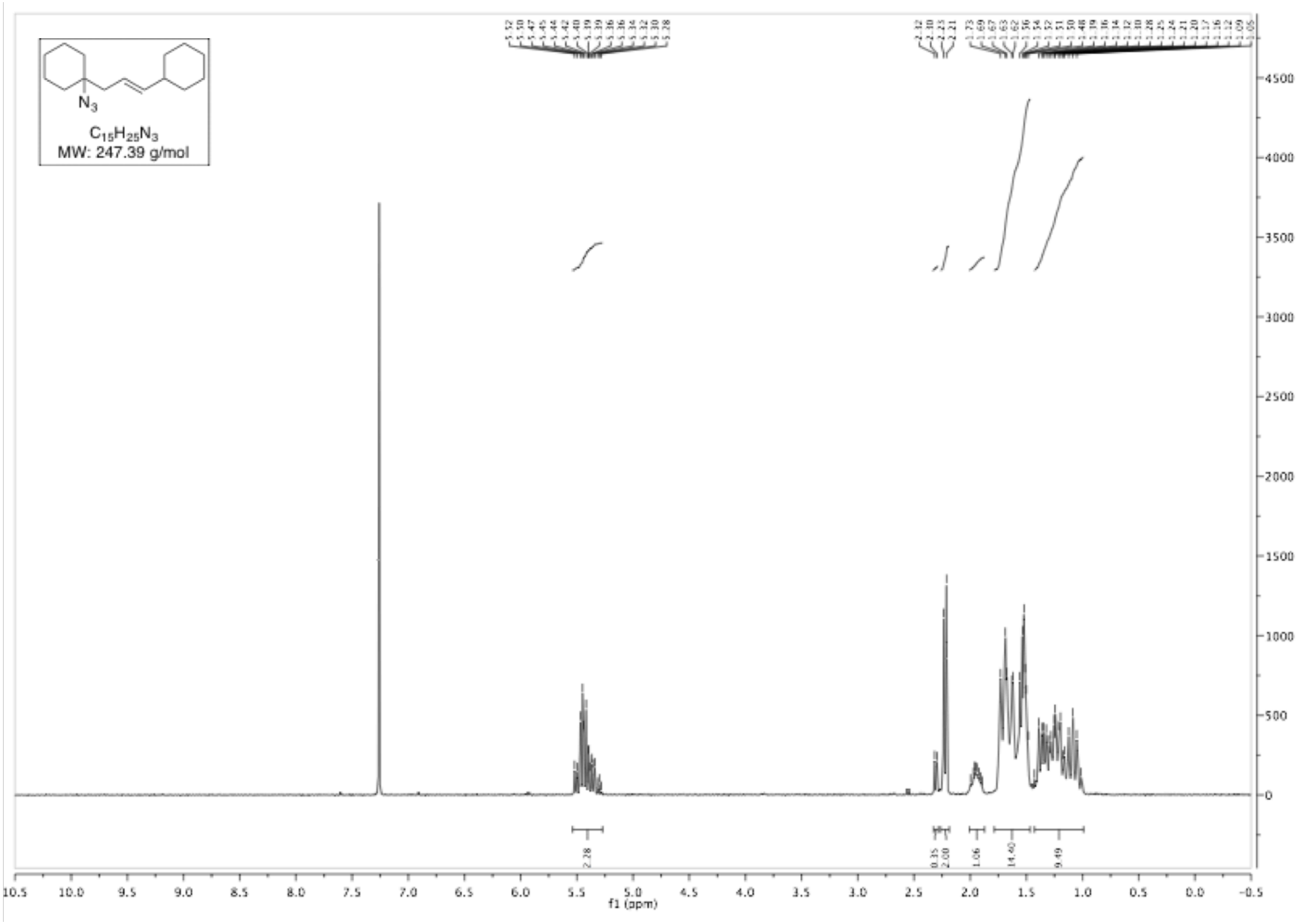
N3
C15H25N3
MW: 247.39 g/mol
f1 (ppm)
2.28
0.35
2.00
1.06
14.40
9.49

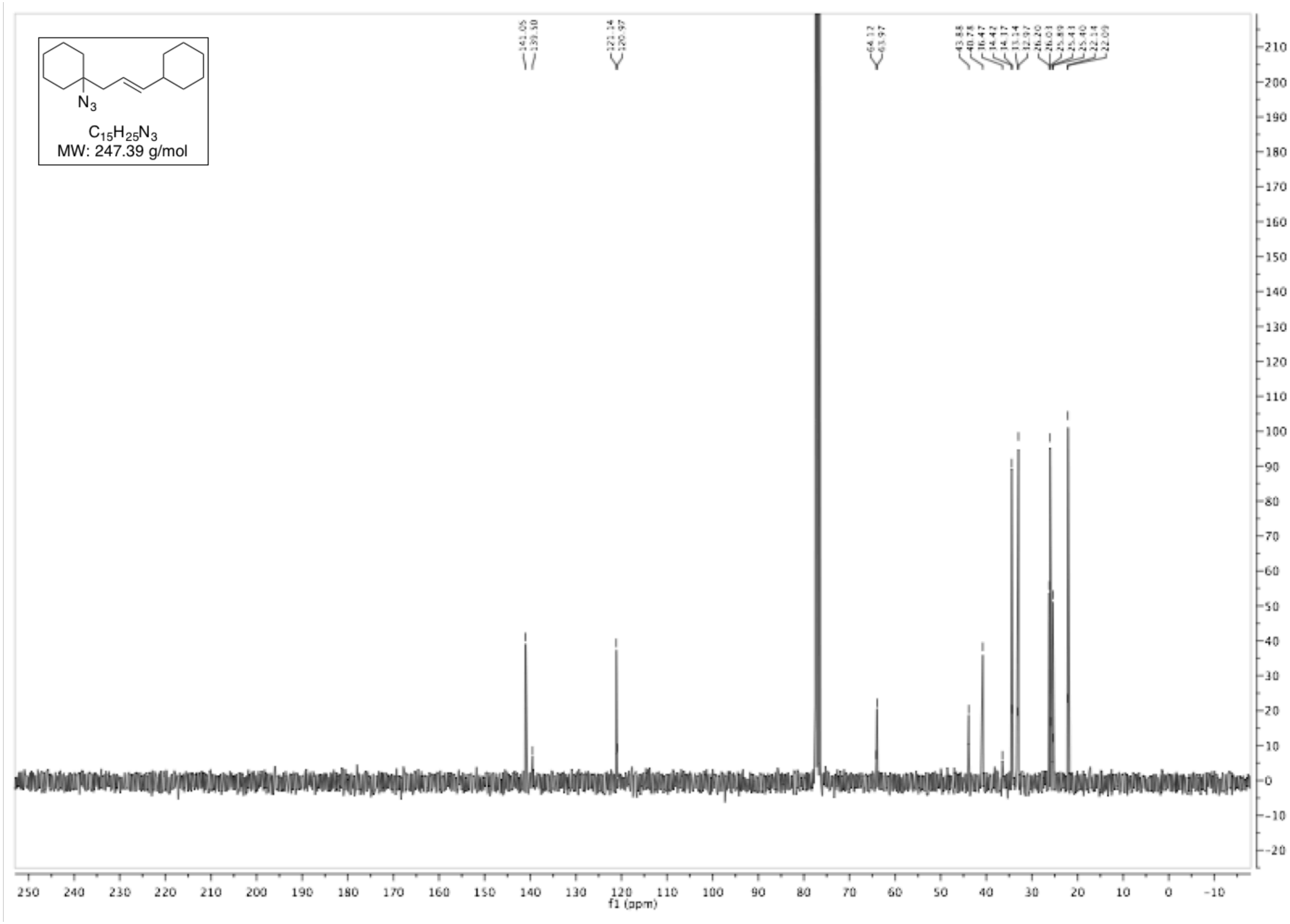
N3
C15H25N3
MW: 247.39 g/mol
141.05
139.50
121.14
120.97
64.12
63.97
43.88
40.78
36.47
34.42
34.17
33.14
32.97
26.20
26.03
25.89
25.43
25.40
22.14
22.09
f1 (ppm)

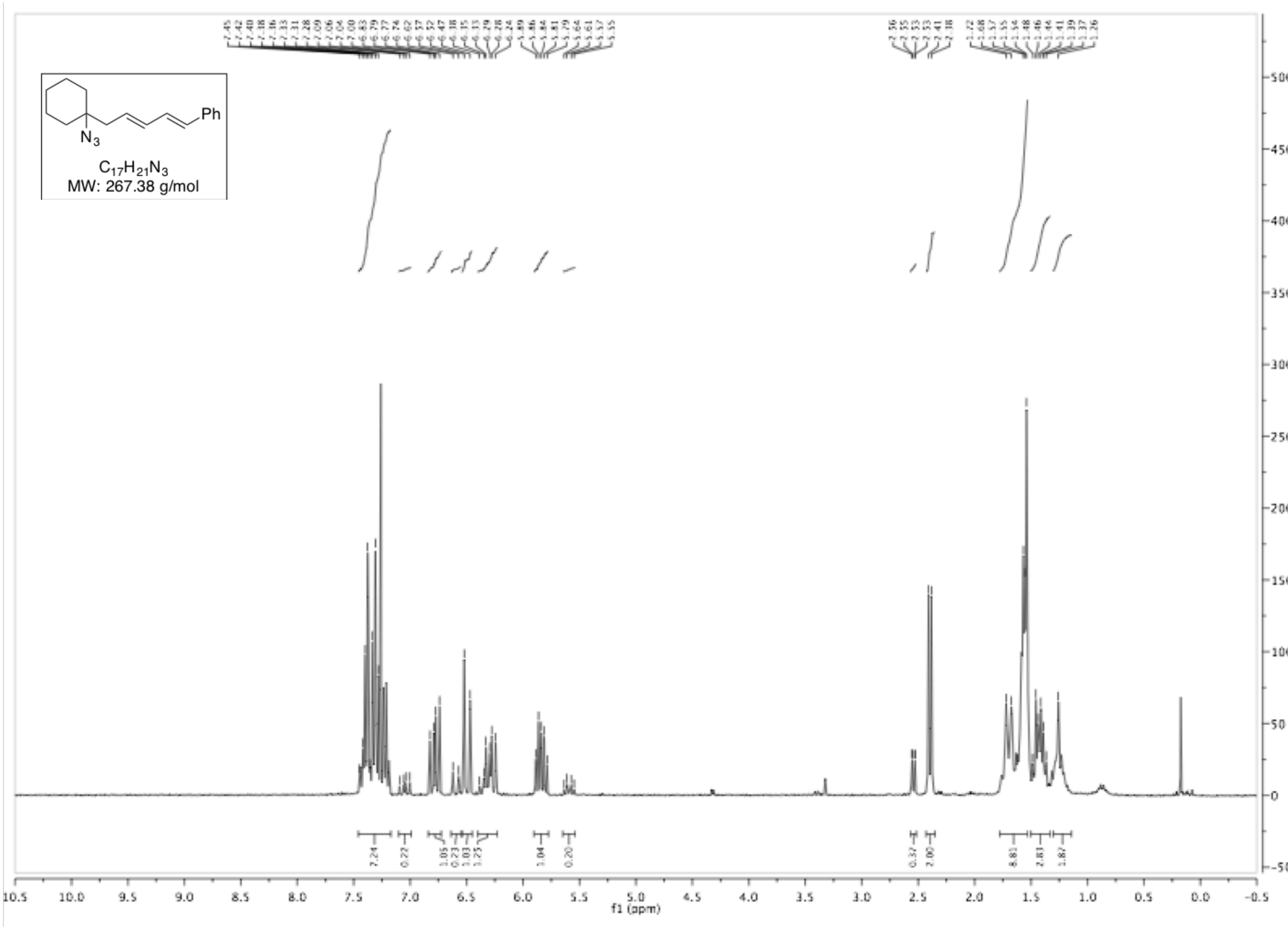
Ph
N_3
$C_{17}H_{21}N_3$
MW: 267.38 g/mol
f1 (ppm)

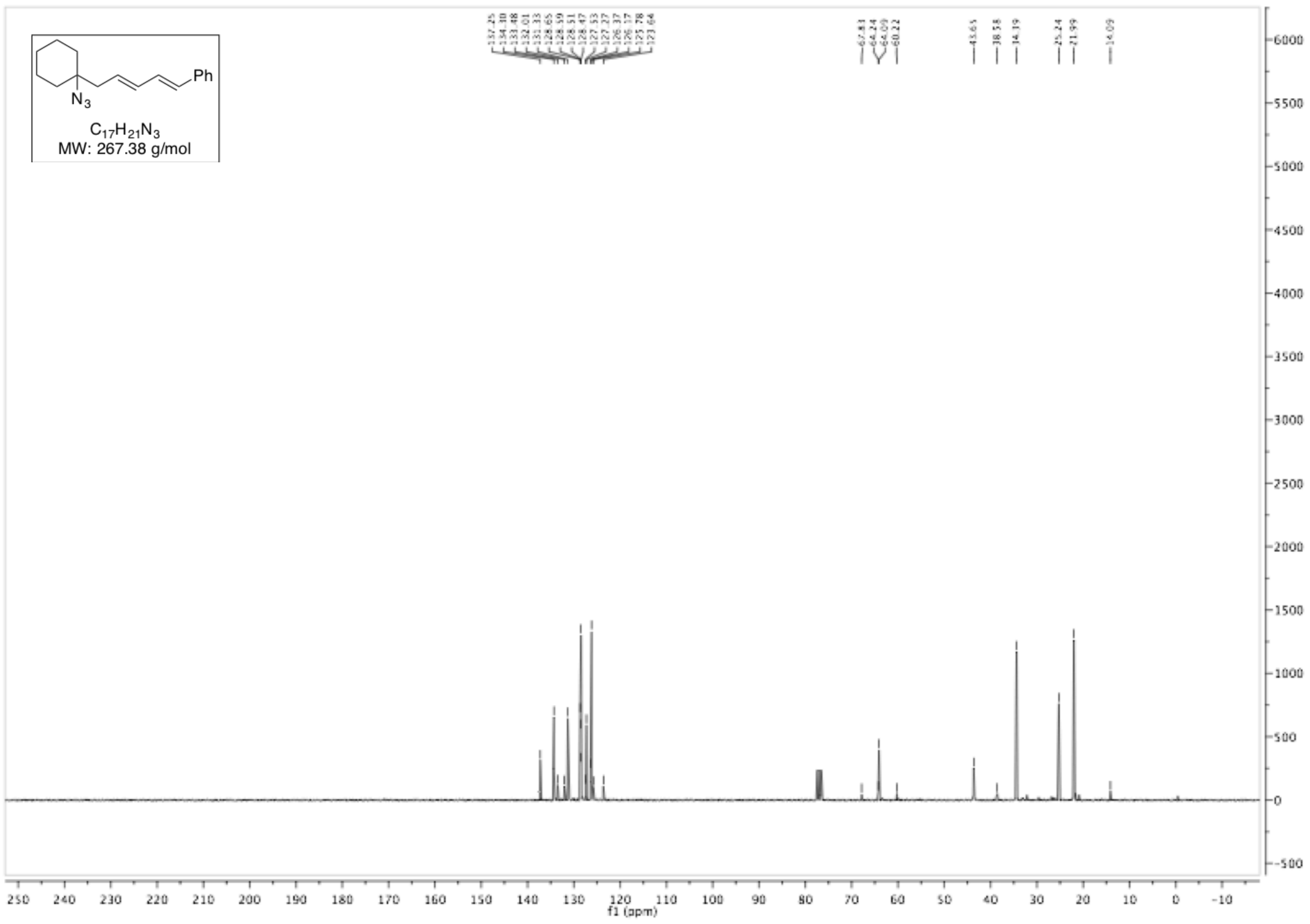
Ph
N3
C17H21N3
MW: 267.38 g/mol
137.25
134.10
133.48
132.01
131.33
128.65
128.59
128.51
128.47
127.53
127.27
126.37
126.17
125.78
123.64
67.83
64.24
64.09
60.22
43.65
38.58
34.39
25.24
21.99
14.09
f1 (ppm)

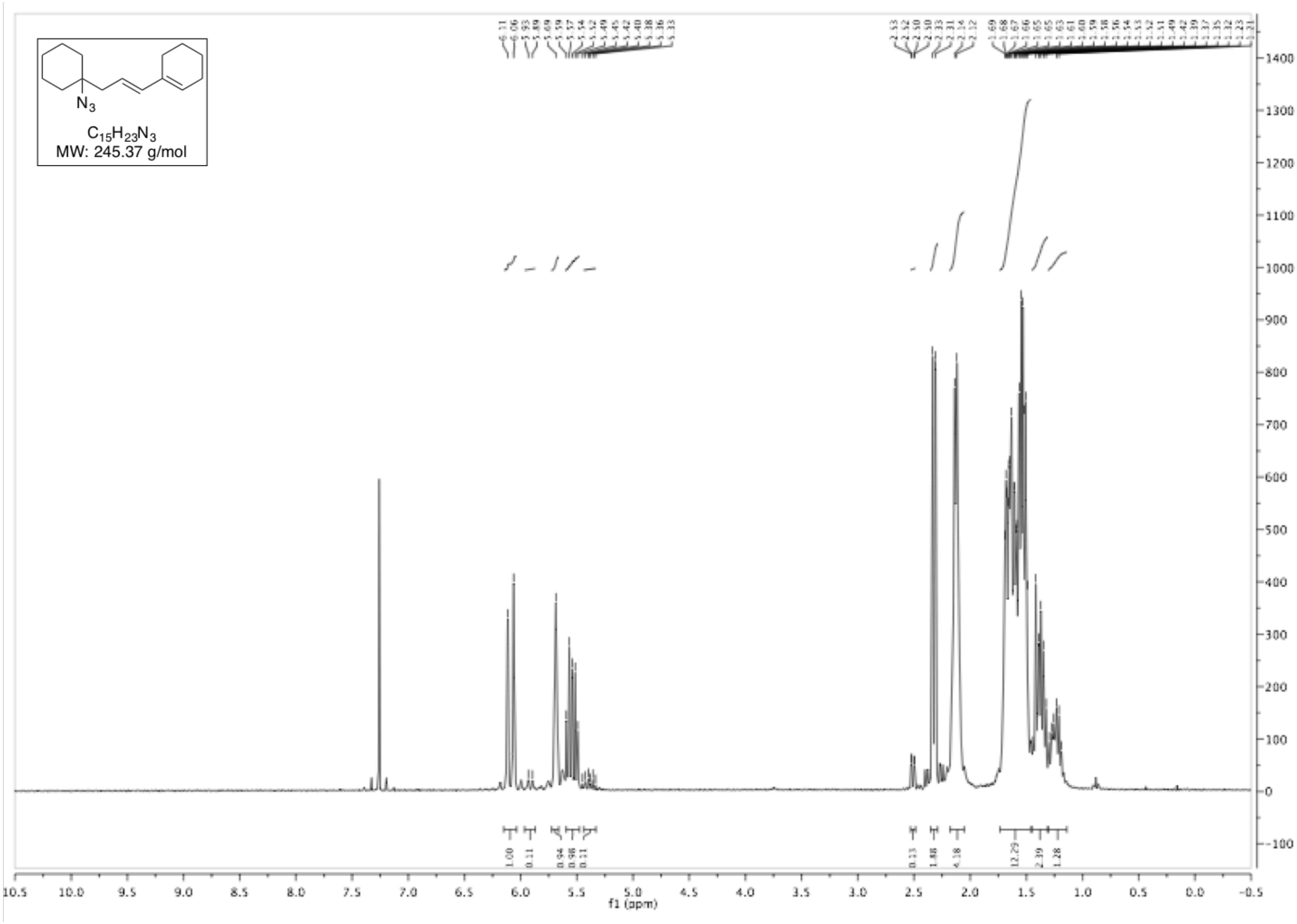

N3
C15H23N3
MW: 245.37 g/mol
6.11 6.06 5.93 5.89 5.69 5.59 5.57 5.54 5.52 5.49 5.45 5.42 5.40 5.38 5.36 5.33
2.53 2.52 2.50 2.50 2.33 2.31 2.14 2.12
1.69 1.68 1.67 1.66 1.65 1.65 1.63 1.61 1.60 1.59 1.58 1.56 1.54 1.53 1.52 1.51 1.49 1.42 1.39 1.37 1.35 1.32 1.23 1.21
1.00 0.11 0.94 0.98 0.11 0.13 1.88 4.18 12.29 2.39 1.28
f1 (ppm)

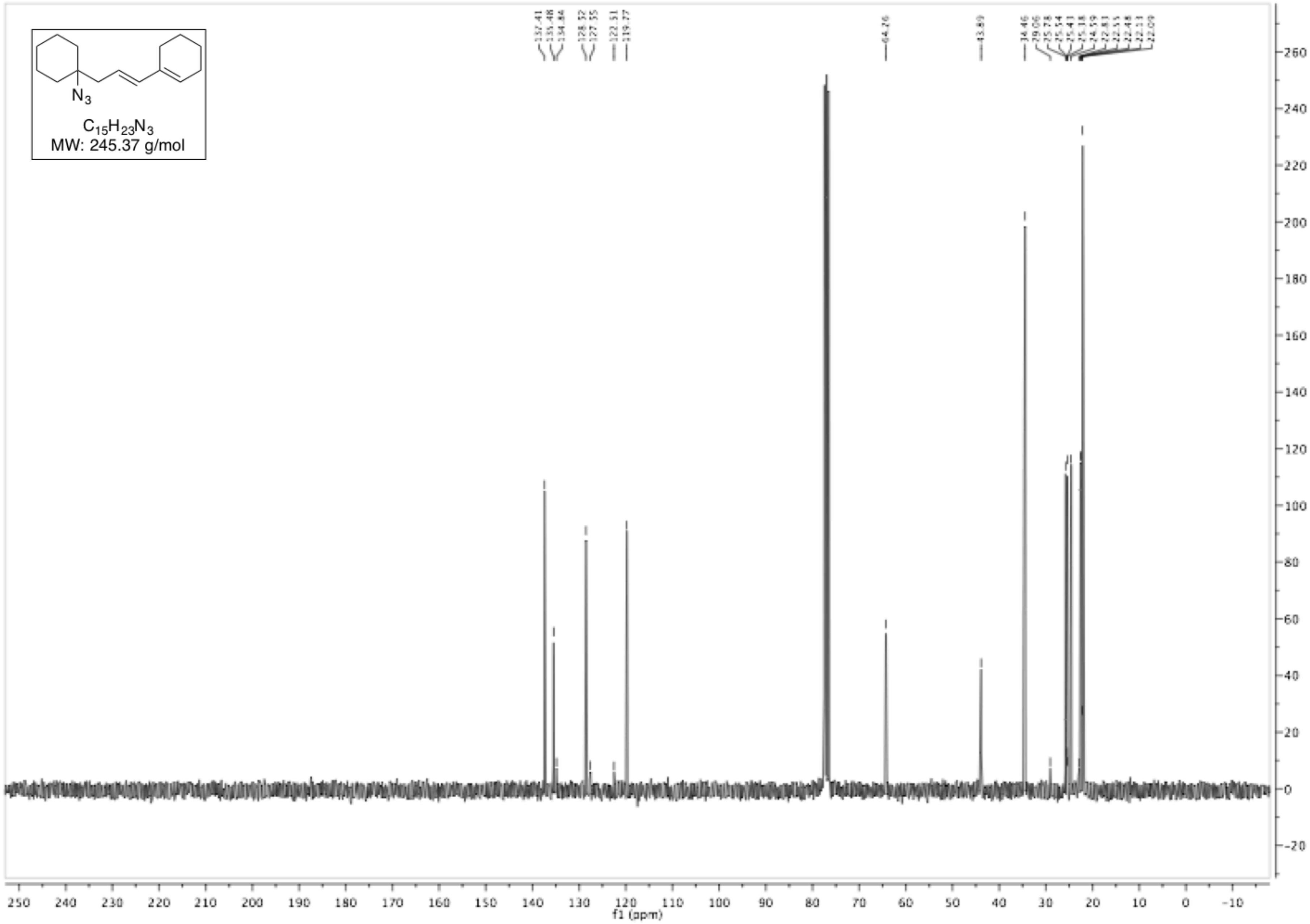
N3
C15H23N3
MW: 245.37 g/mol
137.41
135.48
134.84
128.52
127.55
122.51
119.77
64.26
43.89
34.46
29.06
25.78
25.54
25.41
25.18
24.59
22.81
22.55
22.48
22.13
22.09
f1 (ppm)
